# Asymmetric hydrogenation of 3*H*-azepines *via* catalytic kinetic resolution: access to *anti*-disubstituted azepanes

**DOI:** 10.1039/d6sc01819j

**Published:** 2026-03-27

**Authors:** Linda Bui, Dilara Berna Yildiz, Diego García Matesanz, Esteban Matador, Raquel Sanchez, Iuliana Atodiresei, Giovanni Lonardi, Daniele Leonori

**Affiliations:** a Institute of Organic Chemistry, RWTH Aachen University Landoltweg 1 52056 Aachen Germany giovanni.lonardi@rwth-aachen.de daniele.leonori@rwth-aachen.de; b Department of Chemistry, Faculty of Science, Gazi University Teknikokullar 06500 Ankara Turkey; c Departamento de Química Orgánica, Universidad de Sevilla and Centro de Innovación en Química Avanzada (ORFEO-CINQA) 41012 Sevilla Spain; d Department of Chemistry, University of Manchester Oxford Road Manchester M139PL UK

## Abstract

Azepanes are increasingly valued scaffolds in drug discovery, yet general asymmetric methods for their synthesis remain elusive. This limitation is particularly evident for polysubstituted derivatives bearing stereocenters remote from the nitrogen atom, for which asymmetric access is essentially absent. Here we report the first asymmetric hydrogenation of 3*H*-azepines, enabling direct access to anti-configured azepanes with excellent diastereo- and enantioselectivity (>20 : 1 dr, up to 99 : 1 er). The transformation is mediated by an iridium catalyst featuring a mixed-ligand system composed of a chiral phosphoramidite and an achiral phosphine. Mechanistic studies reveal an unusual sequential pathway in which an initial reduction generates an N-protected intermediate with low enantioselectivity, that subsequently undergoes a highly selective catalytic kinetic resolution. This work provides a catalytic solution to the asymmetric synthesis of disubstituted azepanes and expands the scope of asymmetric hydrogenation to *anti*-selective outcomes beyond classical enantiofacial discrimination.

## Abstract 2

Azepanes are increasingly valued scaffolds in drug discovery, yet general asymmetric methods for their synthesis remain elusive. Herein, asymmetric hydrogenation of 3*H*-azepines affords anti-configured azepanes with excellent diastereo- and enantioselectivity.

## Graphical abstract

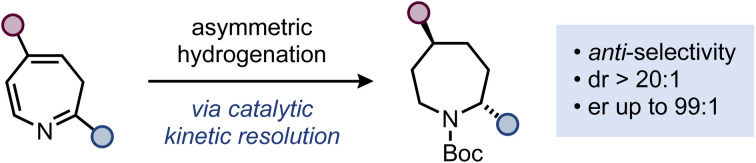

## Introduction

**Scheme 1:**
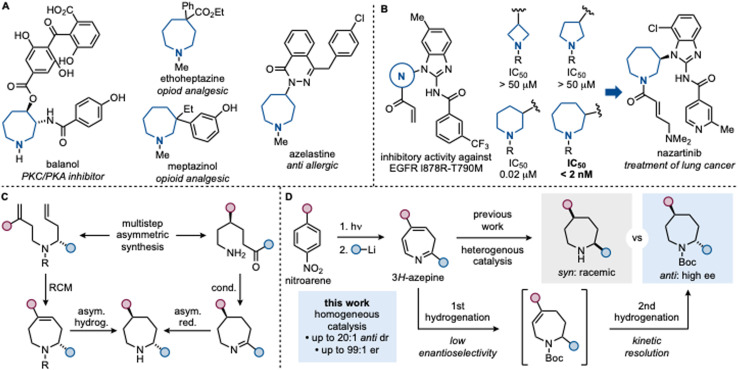
(A) Examples of azepane-containing pharmaceuticals. (B) The azepane core was crucial for the development of nazartinib. (C) Conventional strategies for asymmetric azepane synthesis require the preparation of acyclic precursors. (D) Comparison between our previous photochemical azepane synthesis from nitroarenes leading to racemic *syn*-isomers and the current work where asymmetric hydrogenation conditions provide *anti*-azepanes in high ee.

Stereodefined saturated nitrogen heterocycles are ubiquitous in modern pharmaceuticals.^1–3^ These motifs often impart three-dimensional molecular rigidity, enable precise spatial orientation of substituents, and promote key non-covalent interactions such as H-bonding.^4–7^ Among them, the seven-membered azepane ring has recently emerged as a valuable scaffold for drug discovery and development (Scheme 1A).^8–10^ Its incorporation can markedly improve pharmacokinetic and pharmacodynamic properties, as exemplified by the development of nazartinib, an EGFR inhibitor currently under clinical evaluation by Novartis (Scheme 1B).^11,12^ Structure–activity relationship (SAR) studies revealed that sub-micromolar potency (IC_50_) was dependent on the presence of the azepane unit, which outperformed smaller saturated rings in efficacy.

Despite this promise, azepanes remain markedly underutilized compared to smaller heterocycles. Indeed, while piperidine and pyrrolidine rank among the most common heterocycles in drugs (2nd and 3rd respectively), azepane occupies only the 33rd position.^3^ This underrepresentation arises from fundamental synthetic challenges as azepane construction is limited by a lack of functionalizable precursors and complicated by conformational flexibility, increased ring strain, and the scarcity of reliable ring-forming strategies.^13^ These difficulties are further exacerbated when multiple ring-substituents need to be introduced, particularly under precise stereochemical control.

Although several asymmetric methods exist for the synthesis of C2-monosubstituted azepanes,^14–17^ they generally fail when additional groups must be introduced elsewhere on the ring. In particular, the enantioselective synthesis of polysubstituted azepanes bearing stereocenters remote from nitrogen-atom remains an unresolved challenge. Current strategies typically rely on multistep sequences involving stereodefined acyclic precursors, either with amino ketone functionalities for cyclization and reduction or with two olefins for ring-closing metathesis followed by hydrogenation (Scheme 1C).^18–21^ These strategies suffer from low efficiency, limited modularity, and narrow substrate scope. As a consequence, even industrially relevant azepane-containing compounds are frequently prepared by resolution, which remains the only practical workaround in the absence of general asymmetric methods.^22,23^ A further and often overlooked difficulty lies in diastereochemical control during heterocycle reduction. Hydrogenation of partially unsaturated N-heterocycles overwhelmingly favors *syn*-products, and catalytic access to *anti*-diasteromers remains challenging.^24^ This limitation is well documented for smaller systems such as pyridines, where decades of effort, including recent advances in asymmetric hydrogenation, have started to address stereochemical control.^25^ For larger and more flexible systems such as azepines, no general solution for *anti*-selective, asymmetric reduction has been reported.

Recently, we disclosed a three-step protocol for the synthesis of azepanes from nitroarenes,^26,27^ involving (1) photochemical dearomative N-insertion to generate a 3*H*-azepine intermediate, followed by (2) α-N substitution with organolithium reagents and (3) heterogeneous hydrogenation to afford the *syn*-configured products with high diasteroselectivity (Scheme 1D). This approach demonstrated high fidelity in translating nitroarene substitution patterns into the azepane scaffold, providing a streamlined entry to polysubstituted azepanes. Importantly, however, the stereochemical outcome was intrinsic to the heterogeneous reduction and exclusively favored *syn*-isomers, consistent with precedent in heterocycle hydrogenation. We recently decided to evaluate the possibility of rendering this hydrogenation process asymmetric. However, achieving this would require a complete change in strategy, including the use of homogeneous catalysis conditions with different metal and an appropriate ligand system to imply fundamentally different reactivity and stereochemical control elements.^28–30^ Herein, we report the first asymmetric hydrogenation of 3*H*-azepines, providing direct access to enantioenriched azepanes with high diastereo- and enantioselectivity. The transformation is enabled by a mixed-ligand iridium catalyst system combining a chiral phosphoramidite with an achiral phosphine.^31–34^ In contrast to both our previous azepane synthesis and conventional asymmetric hydrogenations of smaller N-heterocycles, which deliver *syn*-products, this method selectively furnishes *anti*-isomers.^24,26^ Mechanistic investigations reveal that this unexpected stereochemical outcome arises from a sequential hydrogenation process governed by catalytic kinetic resolution rather than classical enantiofacial discrimination. This strategy thus provides a rare catalytic solution to the asymmetric synthesis of disubstituted azepanes and may expand the conceptual scope of asymmetric hydrogenation to *anti*-selective outcomes in saturated N-heterocycles.

## Reaction optimization

**Scheme 2:**
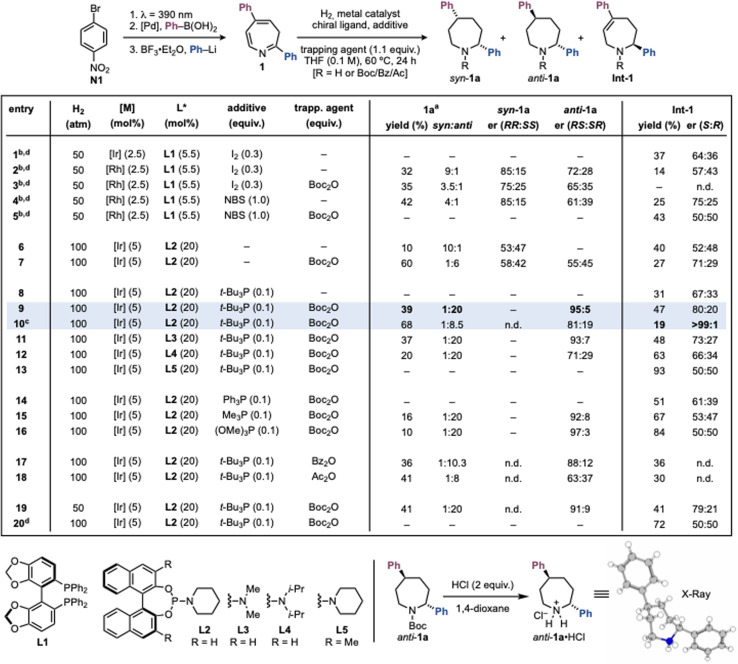
Development of asymmetric hydrogenation of 3*H*-azepine 1. ^*a*^Reported yield for 1a is the yield of the main diasteromer. The dr (*syn* : *anti*) was determined by ^1^H NMR analysis, and er was determined by chiral stationary phase HPLC analysis. ^*b*^Reaction time = 16 h. ^*c*^Reaction time = 48 h. ^*d*^Reaction *T* = 35 °C. [Ir] = [Ir(COD)Cl]_2_; [Rh] = [Rh(COD)Cl]_2_. See SI Section 6 for recovered 1 (rsm). For clarity chlorine anion was removed from the reported crystal structure.

We initiated our studies with the hydrogenation of 2,5-diphenylazepine 1, prepared in three steps from *p*-Br-nitrobenzene N1, to furnish the corresponding 2,5-diphenylazepane 1a (Scheme 2). Our initial working hypothesis was to employ standard iridium- and rhodium-based asymmetric hydrogenation systems with chiral bisphosphine ligands, activated by I_2_.^28,35^ Using [Ir(COD)Cl]_2_ and (*S*)-SEGPHOS (L1) did not produce the desired azepane, and only the partially hydrogenated intermediate Int-1 was isolated in 37% yield and 64 : 36 er (entry 1). Switching to [Rh(COD)Cl]_2_ furnished the *syn*-configured azepane 1a in 32% yield, 9 : 1 dr, and 85 : 15 er (entry 2). However, extensive screening of bisphosphine ligands and reaction parameters failed to improve these results (see SI Section 6.1). Since both product and intermediate feature a free NH group, we suspected that they could strongly coordinate to the [Rh] catalyst and cause deactivation. To address this, we tested the use of trapping agents such as Boc_2_O to protect the amine as soon as generated. However, this did not improve the yield and gave 1a in lower dr and er (entry 3). We also observed that I_2_, while necessary for catalyst activation, promoted decomposition of the starting material 1 (see SI Section 6.1). Alternative activators such as NBS improved efficiency but did not resolve the underlying limitations (entries 4–5).

We then turned to iridium systems with monodentate chiral ligands, which do not require electrophilic activators. In particular, phosphoramidite ligands, which have been established by Feringa and de Vries in asymmetric quinoline and quinoxaline hydrogenations, appeared promising.^36,37^ Initial screens (*e.g.* using L2) gave poor results (entry 6, see SI Section 6.2). Remarkably, however, when Boc_2_O was included as a trapping agent, the azepane 1a was obtained in high yield and 6 : 1 dr favoring the *anti*-isomer (entry 7). This outcome is noteworthy, as the canonical Horiuti–Polanyi hydrogenation pathway typically favors *syn*-products and accessing *anti*-isomers usually requires long synthetic sequences.^24,38^ Interestingly, while the product was racemic, Int-1 was recovered in 71 : 29 er.

**Scheme 3:**
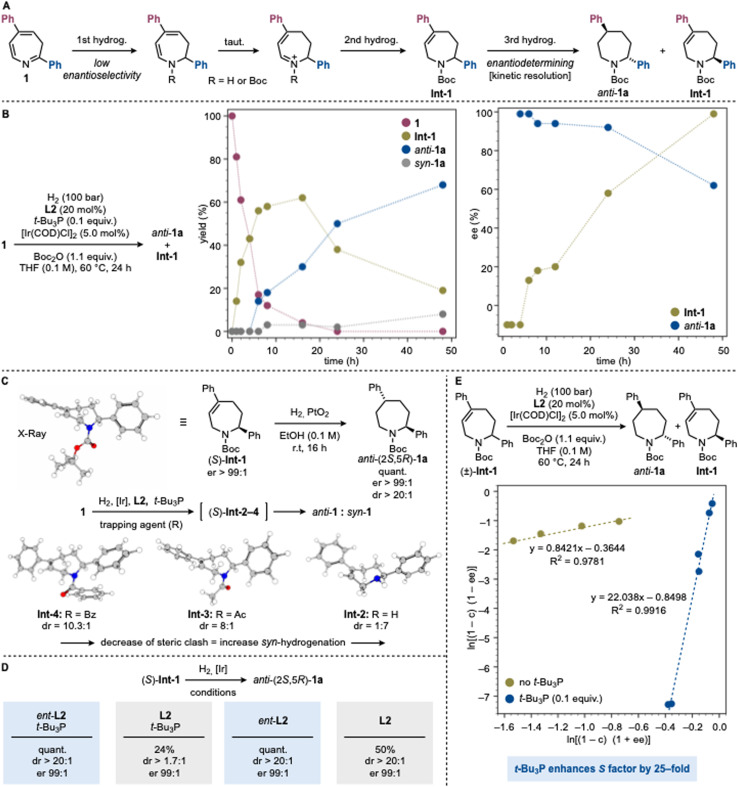
(A) Proposed mechanism for the asymmetric hydrogenation of 1 to *anti*-1a. (B) Reaction time profile for the asymmetric hydrogenation of 1. (C) Diasteroselectivity explained by structural analysis based on the N-protecting group used (UωB97XD/cc-pVTZ//cc-pVDZ(gas)). (D) Match-mismatch effects. (E) Impact of *t*-Bu_3_P on the kinetic resolution. [Ir] = [Ir(COD)Cl]_2_.

Since, the monodentate nature of phosphoramidites enables the use of ancillary ligands, we screened several achiral phosphines to modulate reactivity.^36,39,40^ Pleasingly, the inclusion of *t*-Bu_3_P (0.1 equiv.) greatly enhanced both diasteroselectivity and enantioselectivity, giving *anti*-1a in 39% yield, exclusively as the *anti*-diastereomer, with 95 : 5 er (entry 9). The absolute stereochemistry was established as (2*R*,5*S*) by *N*-Boc deprotection and X-ray analysis of the corresponding azepane·HCl salt. Under these conditions, Int-1 was isolated in 47% yield and 80 : 20 er, with the opposite configuration at C2 (2*S*) (also determined by X-ray analysis, see Scheme 3C). Extending the reaction time increased the yield of *anti*-1a to 61%, but the stereoselectivity eroded (dr 8.5 : 1, er 81 : 19; entry 10), aligned with almost full consumption of (*R*)-Int-1 and the competitive conversion of the opposite (*S*)-Int-1 forming (2*S*,5*R*)-1a. Notably, Int-1 was obtained in 19% yield and >99 : 1 er. This experiment provided the first evidence hinting to an unusual type of kinetic resolution pathway operating under our conditions. Further screening confirmed L2 as the optimum chiral ligand. Phosphoramidites with smaller (L3) or larger (L4) *N*-substituents, or substitution at the BINOL core (L5), gave significantly poorer results (entries 11–13, see SI Section 6.2 for a full ligand screening). Other phosphorus achiral ligands also underperformed relative to *t*-Bu_3_P (entries 14–16, see SI Section 6.2 for full screening of achiral additives). Interestingly, alternative trapping agents led to inferior outcomes, though these experiments yielded insights that guided our mechanistic studies (entries 17–18). Finally, lowering H_2_ pressure (entry 19) or decreasing temperature (entry 20) resulted in reduced selectivity and/or reactivity.

## Mechanistic understanding

While our initial design for the conversion of 1 into 1a was based on a standard asymmetric hydrogenation pathway, two key observations instead pointed to the operation of a kinetic resolution. First, all highly enantioselective experiments gave yields <50%, accompanied by recovery of Int-1 with significant enantioenrichment favoring the opposite C2 configuration (*e.g.*, entry 9). Second, extending the reaction time led to erosion of both dr and er in the product, while the recovered Int-1 became essentially enantiopure (*e.g.*, entry 10).

Together, these results suggest that the hydrogenation proceeds in two sequential phases (Scheme 3A). In the first phase, the chiral Ir complex mediates a hydrogenation at the imine moiety (C2). The resulting enamine undergoes tautomerization and further hydrogenation to provide Int-1 with poor enantioselectivity. *N*-Boc protection likely occurs upon imine hydrogenation or during subsequent transformations. In the second phase, the same catalytic system engages in a kinetic resolution of Int-1, selectively delivering the *anti*-configured product 1a.

To support this mechanistic model, we conducted time-course experiments monitoring intermediate and product formation as well as their enantiomeric composition. As shown in Scheme 3B, substrate consumption initially correlates exclusively with formation of Int-1, while product generation displays a clear induction period (∼5 h). Beyond this point, product formation predominates and is accompanied by a gradual decay of Int-1. In terms of stereochemistry, Int-1 is formed nearly racemic (55 : 45 er), whereas the product appears directly with high enantioselectivity. As the reaction progresses, the er of the product remains initially constant but progressively decreased, while the er of Int-1 steadily increases, ultimately reaching the values observed in the optimized experiments.

To probe the stereodetermining second hydrogenation step, we prepared an authentic sample of (*S*)-Int-1 (>99 : 1 er) and evaluated its reactivity with H_2_ and PtO_2_ as achiral catalyst (Scheme 3C). This species underwent highly diastereoselective hydrogenation to give *anti*-(2*S*,5*R*)-1a in >20 : 1 dr and >99 : 1 er. The strong substrate control can be rationalized by the preferred conformation of Int-1, determined by X-ray crystallography and confirmed by further computational analysis. In this conformation, the C2 phenyl group adopts a pseudo-axial orientation to avoid steric clash with the *N*-Boc group, that folds to the opposite face of the seven-membered ring. This arrangement generates a distinct convex/concave topology that directs the second hydrogenation *anti*.

In addition to its essential role for high reactivity (Scheme 2, entry 8), the Boc group significantly influenced the diastereoselectivity of the process outperforming other protecting groups. Indeed, in the absence of Boc_2_O, compound 1 exhibited drastically reduced reactivity (7%) (*via*Int-2), and delivered the opposite diastereomer, *syn*-1a, with 7 : 1 selectivity. This result underscores the critical role of the Boc substituent in enabling diasteroselection. Alternative protecting groups, like Ac (*via*Int-3) and Bz (*via*Int-4), all led to poorer diastereocontrol (Scheme 2, entries 17–18). Computational analysis of Int-2–4 preferred conformation is consistent with conformational changes that diminish facial differentiation in the seven-membered ring and therefore limits diasteroselectivity (Scheme 3C and SI Section 10.2).

To further probe the interaction of Int-1 with the chiral Ir catalyst, we examined the reactivity of (*S*)-Int-1 with [Ir]/L2/*t*-Bu_3_P and its enantiomeric counterpart *ent*-L2 (Scheme 3D). A pronounced match-mismatch effect was observed.^41^ With *ent*-L2, *anti*-(2*S*,5*R*)-1a was obtained in quantitative yield, >20 : 1 dr, and >99 : 1 er. In contrast, with L2 the yield dropped to 24% with only 1.7 : 1 dr, demonstrating the distinct reactivity of the two enantiomeric catalysts toward (*S*)-Int-1. Interestingly, in the absence of *t*-Bu_3_P the match-mismatch effect was attenuated and with L2, *anti*-(2*S*,5*R*)-1a was obtained in 50% yield and >20 : 1 dr.

These findings suggest that the role of *t*-Bu_3_P is not simply to enhance the kinetic resolution but to modulate the selectivity of the process (Scheme 3E). To probe this, we examined whether the selectivity factor (*S*) remained constant throughout the reaction.^42,43^ Authentic racemic Int-1 was subjected to hydrogenation with [Ir]/L2 both with and without *t*-Bu_3_P. The achiral phosphine increased the stereoselectivity of the kinetic resolution by more than 25-fold, an effect, to the best of our knowledge, not previously reported for mixed-ligand systems (see SI Section 9.2 for full details).

Taken together, these studies highlight the profound impact of the chiral phosphoramidite/*t*-Bu_3_P system on the reactivity of the Ir catalyst. Preliminary evidence supports coordination of both ligands to Ir, but the exact structure of the active species remains elusive (see SI Section 9.4). Our preliminary computational study has provided insights into possible but still speculative ligand complexes, suggesting plausible structural scenarios. We have not yet been able to isolate or definitively characterize such a complex, and further work will be required to unravel the structural and dynamic features of this unusual mixed-ligand catalytic triad. Across all possible mechanisms examined, the conformations leading to the (2*R*,5*S*) stereoisomer consistently emerged as lower in energy than their (2*R*,5*R*)-leading counterparts, providing a rationale for the observed stereochemical outcome. This bias can be attributed to the steric effect of the bulky Boc substituent, which enforces conformational preferences that direct the reaction pathway toward the (2*R*,5*S*) product (see SI Sections 10.2 and 10.3).

## Substrate scope

**Scheme 4:**
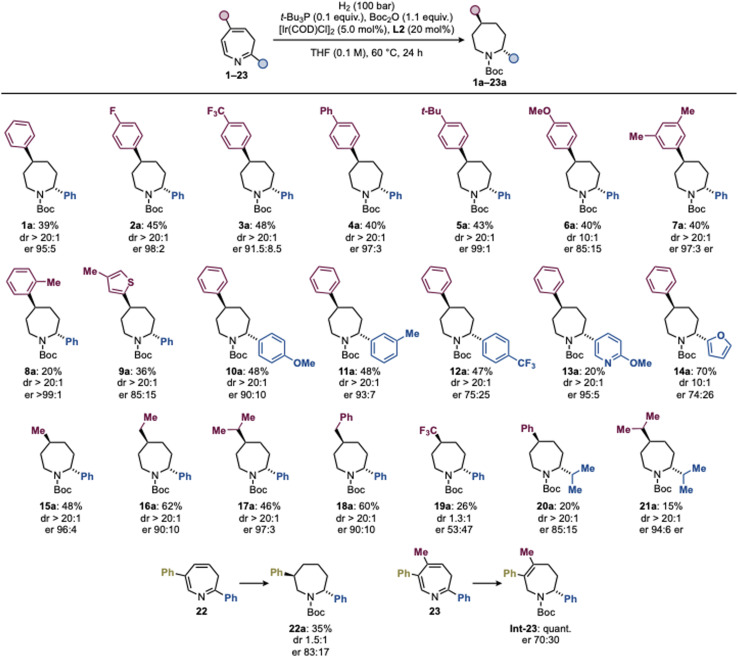
Substrate scope and limitations. The absolute configuration of all the entries was assigned in analogy with 1a.

With the optimized conditions in hand, we evaluated the scope of this asymmetric hydrogenation process platform for azepane synthesis (Scheme 4). We began by targeting *anti*-2,5-diaryl azepanes, a particularly challenging and largely inaccessible azepane subclass. To date, their synthesis has relied on non-modular sequences based on stepwise functional group manipulations, with no general or asymmetric catalytic approach available.^44^ Against this backdrop, we examined a series of 2-phenyl-5-aryl-3*H*-azepines and found that the transformation translated effectively across a broad range of aryl substituents. Both electron-withdrawing groups, including F (2a) and CF_3_ (3a), and electron-donating substituents such as phenyl (4a), *t*-Bu (5a), and OMe (6a) were well tolerated, delivering the corresponding *anti*-azepanes with high levels of stereocontrol. An *ortho*-methyl substituent (8a) led to a less efficient resolution in terms of yield but maintained excellent diastereo- and enantioselectivity. Compatibility was also observed for a *meta*,*meta*-disubstituted aryl system (7a) and for a heteroaryl 2-thienyl substituent (9a), although the latter displayed diminished stereoselectivity.

We next probed the influence of the C2 aryl substituent in the presence of a fixed C5 phenyl group. *para*-OMe (10a) and *meta*-Me (11a) derivatives performed well, whereas a strongly electron-withdrawing *para*-CF_3_ substituent (12a) resulted in a less efficient kinetic resolution. Among heteroaryl substituents, a 3-pyridyl group (13a) afforded good enantioselectivity, while an electron-rich and sterically smaller 2-furyl substituent (14a) led to significantly reduced diastereo- and enantiocontrol, consistent with diminished facial differentiation in the corresponding intermediate.

We then turned to 2-aryl-5-alkyl azepines, a class of compounds for which no asymmetric synthetic approaches have been reported to date. Pleasingly, the catalytic kinetic resolution strategy translated efficiently to substrates bearing 5-Me (15a), Et (16a), *i*-Pr (17a), and Bn (18a) substituents, generally affording the *anti*-azepanes in good yields and with good to excellent enantioselectivities. A limitation was encountered for the 5-CF_3_ derivative (19a), which furnished the product with low diastereoselectivity and as a racemate.

We further examined 2-alkyl-5-aryl azepines, another subclass for which asymmetric access is entirely unprecedented. An *i*-Pr substituent at C2 (20a) delivered the desired azepane in moderate yield and enantioselectivity, while a more sterically demanding 2,5-dialkyl azepine was also successfully converted. In this case, the di-*i*-Pr derivative (21a) was obtained with excellent stereocontrol, demonstrating that even highly substituted and conformationally flexible systems can be accommodated. Collectively, compounds 15a–21a represent the first asymmetric syntheses of 2-aryl-5-alkyl and 2,5-dialkyl azepanes and underscore the ability of this approach to access substitution patterns that have been previously inaccessible by current methods.

Finally, we evaluated the impact of substituent position beyond 2,5-disubstitution. A 2,6-diphenyl-3*H*-azepine (22) was successfully converted to the corresponding *anti*-2,6-diphenyl azepane 22a in good yield and a promising 83 : 17 er, highlighting the potential applicability of the strategy to other systems. In contrast, more highly substituted 3*H*-azepines proved unreactive under the current conditions; for example, trisubstituted 23 underwent only partial reduction to afford the corresponding intermediate Int-23 in quantitative yield.

## Conclusions

The asymmetric synthesis of polysubstituted azepanes remains a long-standing challenge that has limited the broader use of these heterocycles in drug discovery. In this work, we have established the first asymmetric hydrogenation of 3*H*-azepines, enabling direct access to enantioenriched azepanes from readily available nitroarene precursors. Crucially, the transformation selectively furnishes *anti*-configured products, a stereochemical outcome that is intrinsically disfavored in the hydrogenation of unsaturated N-heterocycles. Mechanistic studies reveal that this selectivity does not arise from conventional enantiofacial discrimination, but from a sequential hydrogenation process in which an initial reduction of low enantioselectivity is followed by a highly selective catalytic kinetic resolution. This mechanism contrasts with established approaches to heterocycle reduction and provides a catalytic solution to the asymmetric synthesis of disubstituted azepanes. We anticipate that the generality of the method will facilitate the incorporation of azepane motifs into screening libraries and inspire the development of related hydrogenation strategies for other classes of substituted azepanes.

## Author contributions

G. L. and D. L. designed the project. L. B. ran all synthetic experiments; D. G. M., E. M. M., R. S. B. ran additional experiments; D. B. Y. ran the computational studies. I. A. resolved the X-ray crystal structures. All authors discussed the results and wrote the manuscript.

## Conflicts of interest

There are no conflicts to declare.

## Supplementary Material

SC-OLF-D6SC01819J-s001

SC-OLF-D6SC01819J-s002

## Data Availability

All data is available in the supplementary information (SI). Supplementary information is available. See DOI: https://doi.org/10.1039/d6sc01819j. CCDC 2540109 and 2540110 contain the supplementary crystallographic data for this paper.^45*a*,*b*^
