# Supplementary material for: Asymmetric hydrogenation of 3H-azepines via catalytic kinetic resolution: access to anti-disubstituted azepanes
Source: Chem Sci. 2026 Mar 27;17(20):10007–14. doi: 10.1039/d6sc01819j (PMC13054887; doi:10.1039/d6sc01819j)
Supplement: SC-017-D6SC01819J-s002 [file SC-017-D6SC01819J-s002.pdf]

Supplementary Information (SI) for Chemical Science.
This journal is © The Royal Society of Chemistry 2026

Stereoselective Synthesis of Disubstituted Azepanes via Catalytic Kinetic Resolution of 3*H*-Azepines

Linda Bui,[1] Dilara Berna Yildiz,[1,2] Diego García Matesanz,[1] Esteban Matador,[1,3] Raquel Sanchez,[4] Iuliana Atodiresei,[1] Giovanni Lonardi,[1,*] and Daniele Leonori[1,*]

[1] *Institute of Organic Chemistry, RWTH Aachen University, Landoltweg 1, 52056 Aachen, Germany.*

[2] *Department of Chemistry, Faculty of Science, Gazi University, Teknikokullar, 06500 Ankara, Türkiye.*

[3] *Departamento de Química Orgánica, Universidad de Sevilla and Centro de Innovación en Química Avanzada (ORFEO-CINQA), 41012 Sevilla, Spain.*

[4] *Department of Chemistry, University of Manchester, Oxford Road, Manchester M139PL, UK.*

giovanni.lonardi@rwth-aachen.de & daniele.leonori@rwth-aachen.de

Table of Contents

1 General Experimental Details 3
2 Preparation of Starting Materials and Intermediates 4
2.1 General Procedure for the Ring Expansion of Nitroarenes and Nitrenes – GP1 4
2.2 General Procedure for the Suzuki-Miyaura Cross-coupling of **S1** – GP2 7
2.3 General Procedure for the Organolithium Addition to 3*H*-Azepin-2-amines – GP3 11
3 Chiral Ligands Synthesis 20
4 Overview of Azepine Starting Materials 28
5 Pictures of Reactions Set-up 29
6 Reaction Optimization 30
6.1 General Procedure for the Reaction Optimization of the Asymmetric Reduction of Azepines Using Bisphosphine Ligands – GP5 30
6.2 General Procedure for the Reaction Optimization of the Asymmetric Reduction of Azepines Using Monodentate Ligands – GP6 37
7 Preparation of Azepanes 49
7.1 Preparation of Monosubstituted Azepane 65
8 Reproducibility 66
9 Mechanistic Experiments 67
9.1 Time-profile 67
9.2 Determination of the S–factor 69
9.3 Impact of the [Ir]/L2/*t*-Bu_3P Ratio on Reactivity and Selectivity 73
9.4 ^{31}P NMR Studies of the Ir/L2/*t*-Bu_3P Complexes 75
10 Computational Studies 76
10.1 Computational Details 76
10.2 Conformational Analysis 78
10.3 Mechanistic Studies 90
11 HPLC Traces 99
12 NMR Spectra 112
13 References 179

1 General Experimental Details

All required fine chemicals were used directly without purification unless stated otherwise. All air and moisture sensitive reactions were carried out under nitrogen atmosphere using standard Schlenk manifold technique. All solvents were bought from Acros as 99.8% purity and degassed by N_2 bubbling. N_2 was purchased from Air Products, Ar and H_2 were purchased from Nippon gases. ^{1}H, ^{13}C, ^{19}F and ^{31}P NMR spectra were recorded on Bruker Avance Neo 600 MHz, Varian VNMRS 600 MHz, or on Varian VNMRS 400 MHz. ^{1}H, ^{13}C, ^{19}F and ^{31}P Nuclear Magnetic Resonance (NMR) spectra were acquired at various field strengths as indicated and were referenced to the residual peak solvent (for ^{1}H and ^{13}C) or by the instrument internally after locking and shimming to the deuterated solvent (for ^{19}F and ^{31}P). ^{1}H NMR coupling constants (*J*) are reported in Hertz (Hz) and refer to apparent multiplicities and not true coupling constants. Data is reported as follow: chemical shift (δ), integration, multiplicity (s = singlet, br s = broad singlet, d = doublet, t = triplet, q = quartet, p = quintet, sx = sextet, sp = septet, m = multiplet, dd = doublet of doublets, etc.), proton assignment (determined by 2D NMR experiments: COSY, HSQC, HMBC and NOESY) where possible. High-resolution mass spectra were obtained using a JEOL JMS-700 spectrometer or a Fissions VG Trio 2000 quadrupole mass spectrometer. Spectra were obtained using electron impact ionization (EI) or positive electrospray (ESI) techniques. Enantiomeric excesses were determined on a Shimadzu HPLC LC2050C-3D with a PDA detector, using chiral stationary phase columns (CHIRACEL® OD-H and OJ-H, CHIRALPAK® IA and AD-H, 250 x 4.6 mm, 5μm) from Daisel. Optical rotation data were collected on a digital polarimeter P3000 from A. Krüss Optronic GmbH, using a 100 mm cell with 589 nm polarised light at room temperature. Analytical TLC: aluminium backed plates pre-coated (0.25 mm) with Merck Silica Gel 60 F254. Compounds were visualized by exposure to UV-light or by dipping the plates in ninhydrin and permanganate ($KMnO_4$) stain followed by heating. Flash column chromatography was performed using Merck Silica Gel 60 (40–63 μm) or CombiFlash® Rf+. To enable a clean separation, some azepanes were treated with *m*-CPBA without racemization of the product (1.5 equiv., DCM (0.1 M), r.t., 4-5 h) prior to purification. All mixed solvent eluents are reported as V/V solutions. The LEDs used are Kessil PR 160 427nm (blue LEDs) and 390 nm (purple LEDs). All the reactions were conducted in CEM 9 mL glass microwave tubes. All hydrogenation reactions were conducted in a Berghof high-pressure reactor BHL-800 connected to a temperature controller Berghof BTC-3000.

2 Preparation of Starting Materials and Intermediates

2.1 General Procedure for the Ring Expansion of Nitroarenes and Nitrenes – GP1

General Procedure for the Ring Expansion of Nitroarenes – GP1a

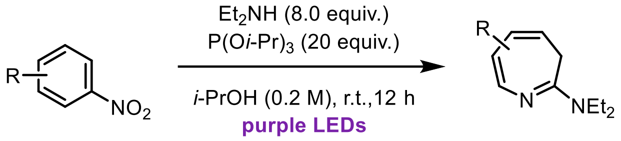

A round bottom flask equipped with a stirring bar was charged with the nitroarene (1.0 equiv.). The flask was capped with a septum (PTFE/butyl), evacuated and refilled with N_2 (x 3). Et_2NH (8.0 equiv.), degassed P(O*i*-Pr)$_3$ (20 equiv.) and degassed *i*-PrOH (0.2 M), were added. The purple LEDs and the fan were switched on and the mixture was stirred under irradiation at room temperature for 12 h. The solvent was evaporated and an excess of HCl (2.0 M in Et_2O) was added until formation of a precipitate. The solvent was evaporated, and the residue was purified by column chromatography on silica gel.

General Procedure for the Ring Expansion of Arylazides – GP1b

Arylazides were synthesized following procedure from the literature.[1]

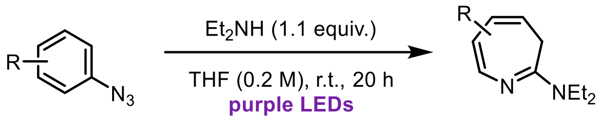

A round bottom flask equipped with a stirring bar was charged with the azide (1.0 equiv.). The flask was capped with a septum (PTFE/butyl), evacuated and refilled with N_2 (x 3). Et_2NH (1.1 equiv.) and degassed THF (0.2 M) were added. The purple LEDs and the fan were switched on and the mixture was stirred under irradiation at room temperature for 20 h. The solvent was evaporated, and the residue was purified by column chromatography on silica gel.

5-Bromo-*N,N*-diethyl-3*H*-azepin-2-amine (S1)

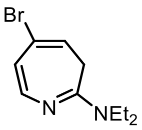

Following **GP1a**, 1-bromo-4-nitrobenzene **N1** (3.0 g, 15 mmol), gave **S1** (1.6 g, 44%) as an oil. ^{1}H NMR (500 MHz, $CDCl_3$) δ 7.00 (1H, d, *J* = 8.0 Hz), 5.79 (1H, d, *J* = 9.3 Hz), 5.26 (1H, t, *J* = 8.1 Hz), 3.37 (4H, br s), 2.58 (2H, br s), 1.16 (6H, br s); ^{13}C NMR (126 MHz, $CDCl_3$) δ 145.8, 142.0, 122.2, 111.3, 111.2, 43.4, 31.7, 13.7. Data in accordance with the literature.[2]

_N,N_-Diethyl-5-methyl-3_H_-azepin-2-amine (S2)

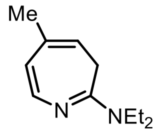

Following **GP1a**, 4-nitrotoluene (1.0 mL, 10 mmol), gave **S2** (2.7 g, 50%) as an oil. ^{1}H NMR (500 MHz, $CDCl_3$) δ 7.01 (1H, d, J = 8.1 Hz), 5.62 (1H, d, J = 8.1 Hz), 4.87 (1H, t, J = 7.3 Hz), 3.37 (4H, q, J = 7.0 Hz), 2.60 (2H, br s), 1.88 (3H, s), 1.15 (6H, t, J = 7.0 Hz); ^{13}C NMR (126 MHz, $CDCl_3$) δ 147.3, 140.1, 137.2, 113.1, 109.1, 43.1, 30.8, 21.2, 13.5. Data in accordance with the literature.[3]

_N,N_-Diethyl-5-ethyl-3_H_-azepin-2-amine (S3)

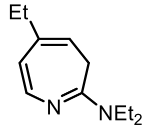

Following **GP1b**, 1-azido-4-ethylbenzene (145 mg, 1,0 mmol), gave **S3** (134 mg, 71%) as an oil. ^{1}H NMR (600 MHz, $CDCl_3$) δ 7.04 (1H, d, J = 8.1 Hz), 5.65 (1H, dd, J = 8.1, 1.2 Hz), 4.87 (1H, t, J = 7.1 Hz), 3.36 (4H, q, J = 7.2 Hz), 2.60 (2H, br s), 2.19 (2H, q, J = 7.5 Hz), 1.13 (6H, t, J = 7.2 Hz), 1.04 (3H, t, J = 7.5 Hz); ^{13}C NMR (151 MHz, $CDCl_3$) δ 147.1, 143.2, 140.3, 112.1, 107.5, 43.0, 30.7, 28.3, 14.2, 13.5 (br s). Data in accordance with the literature.[4]

_N,N_-Diethyl-5-_iso_-propyl-3_H_-azepin-2-amine (S4)

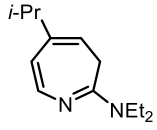

Following **GP1b**, 1-azido-4-*iso*-propyl benzene (1.0 g, 6.5 mmol), gave **S4** (650 mg, 49%) as an oil. R_f 0.45 [hexane:EtOAc (4:1) + Et_3N (2.5%)]; ^{1}H NMR (600 MHz, $CDCl_3$) δ 7.07 (1H, d, J = 8.2 Hz), 5.70 (1H, d, J = 8.2 Hz), 4.88 (1H, t, J = 7.1 Hz), 3.43–3.30 (4H, m), 2.58 (2H, br s), 2.43 (1H, hept, J = 7.1 Hz), 1.13 (6H, br t, J = 7.1 Hz), 1.05 (6H, d, J = 6.9 Hz); ^{13}C NMR (151 MHz, $CDCl_3$) δ 147.8, 147.3, 140.7, 110.8, 106.4, 43.2, 33.6, 30.8, 23.0, 13.7; HRMS (ESI): found MH^+ 207.1857, $C_{13}H_{23}N_2$ requires 207.1856.

5-Benzyl-_N,N_-diethyl-3_H_-azepin-2-amine (S5)

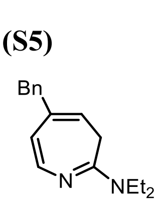

Following **GP1a**, 4-nitrodiphenylmethane (2.8 mL, 15 mmol) gave **S5** (2.3 g, 60%) as an oil. ^{1}H NMR (500 MHz, $CDCl_3$) δ 7.25 (2H, t, J = 7.4 Hz), 7.17 (1H, t, J = 7.4 Hz), 7.12 (2H, d, J = 7.0 Hz), 7.03 (1H, d, J = 8.1 Hz), 5.62 (1H, d, J = 8.1 Hz), 4.91 (1H, t, J = 7.1 Hz), 3.53 (2H, s), 3.39 (4H, br s), 2.65

(2H, br s), 1.15 (6H, t, J = 6.5 Hz); ^{13}C NMR (126 MHz, $CDCl_3$) δ 147.1, 140.8, 140.4, 128.8, 128.2, 128.2, 125.9, 112.2, 110.3, 43.3, 41.4, 30.9, 13.6. Data in accordance with the literature.[5]

***N,N*-Diethyl-5-(trifluoromethyl)-3*H*-azepin-2-amine (S6)**

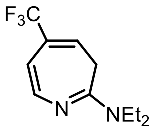

Following **GP1a**, 4-nitrobenzotrifluoride (1.1 mL, 8.0 mmol), gave **S6** (823 mg, 43%) as an oil. ^{1}H NMR (500 MHz, $CDCl_3$) δ 7.25 (1H, d, J = 8.1 Hz), 5.83 (1H, dd, J = 8.1, 1.1 Hz), 5.53 (1H, t, J = 7.5 Hz), 3.38 (4H, br s), 2.71 (2H, br s), 1.16 (6H, br s); ^{13}C NMR (151 MHz, $CDCl_3$) δ 145.1, 143.3, 131.7 (q, J = 30.3 Hz), 123.9 (q, J = 273.3 Hz), 111.8 (q, J = 4.9 Hz), 103.3 (q, J = 2.2 Hz), 43.4, 30.7, 14.2; ^{19}F NMR (471 MHz, $CDCl_3$) δ –65.02 (s). Data in accordance with the literature.[6]

***N,N*-Diethyl-6-phenyl-3*H*-azepin-2-amine (S7)**

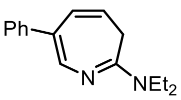

Following **GP1a** but using blue LEDs, 3-nitrobiphenyl (2.5 mL, 10 mmol), gave **S7** (690 mg, 19%) as an oil. ^{1}H NMR (500 MHz, $CDCl_3$) δ 7.58 (1H, br s), 7.46–7.41 (2H, m), 7.34 (2H, t, J = 7.7 Hz), 7.20 (1H, tt, J = 7.0, 1.2 Hz), 6.48 (1H, dd, J = 8.9, 1.4 Hz), 5.32 (1H, app q, J = 7.5 Hz), 3.41 (4H, q, J = 6.8 Hz), 2.75 (2H, br s), 1.19 (6H, t, J = 6.7 Hz); ^{13}C NMR (126 MHz, $CDCl_3$) δ 145.5, 142.0, 139.0, 129.5, 128.3, 126.8, 125.7, 123.0, 113.6, 43.3, 31.9, 13.6. Data in accordance with the literature.[5]

***N,N*-Diethyl-5-methyl-6-phenyl-3*H*-azepin-2-amine (S8)**

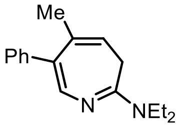

Following **GP1a**, 2-methyl-5-nitro-1,1'-biphenyl (852 mg, 4.0 mmol, 1.0 equiv.), gave **S8** (532 mg, 52%) as an oil. ^{1}H NMR (600 MHz, $CDCl_3$) δ 7.35–7.30 (4H, m), 7.24–7.19 (2H, m), 5.14 (1H, tq, J = 7.4, 1.4 Hz), 3.41 (4H, q, J = 7.4 Hz), 1.84 (2H, br s), 1.78 (3H, s), 1.19 (6H, t, J = 7.4 Hz); ^{13}C NMR (151 MHz, $CDCl_3$) δ 148.4, 141.8, 139.9, 136.0, 129.4, 128.1, 127.2, 125.9, 112.8, 43.1, 31.0, 20.6, 13.8 (br s). Data in accordance with the literature.[5]

2.2 General Procedure for the Suzuki-Miyaura Cross-coupling of S1 – GP2

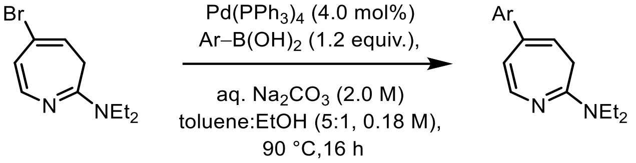

A round bottom flask equipped with a condenser was charged with **S1** (1.0 equiv.), the boronic acid (1.2 equiv.), and $Pd(PPh_3)_4$ (4.0 mol%). The flask was evacuated and refilled with N_2 (x 3). A mixture of toluene–EtOH (5:1, 0.18 M) and an aqueous solution of Na_2CO_3 (2.4 mL/mmol, 2.0 M) were added to the solids. The mixture was stirred at 90 ºC for 16 h. The mixture was cooled to room temperature, diluted with H_2O, and extracted with EtOAc (x 3). The combined organic layers were washed with brine, dried ($MgSO_4$), filtered, and evaporated. The residue was purified by column chromatography on silica gel.

***N,N*-Diethyl-5-phenyl-3*H*-azepin-2-amine (S9)**

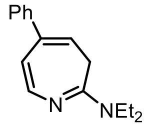

Following **GP2**, **S1** (850 mg, 3.5 mmol) and phenylboronic acid (2.4 g, 19 mmol), gave **S9** (3.8 g, 78%) as a solid. ^{1}H NMR (500 MHz, $CDCl_3$) δ 7.46 (2H, dd, J = 8.3, 1.3 Hz), 7.37–7.31 (2H, m), 7.31–7.26 (2H, m), 5.98 (1H, dd, J = 8.1, 1.0 Hz), 5.34 (1H, t, J = 7.4 Hz), 3.40 (4H, q, J = 7.5 Hz), 2.88 (2H, br s), 1.16 (6H, br s); ^{13}C NMR (126 MHz, $CDCl_3$) δ 146.5, 142.1, 141.3, 140.8, 128.2, 127.7, 127.2, 110.2, 108.4, 43.2, 31.4, 13.6. Data in accordance with the literature.[2]

***N,N*-Diethyl-5-(4-fluorophenyl)-3*H*-azepin-2-amine (S10)**

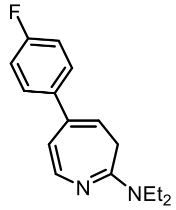

Following **GP2**, **S1** (300 mg, 1.2 mmol) and (4-fluorophenyl)boronic acid (207 mg, 1.5 mmol), gave **S10** (233 mg, 73%) as a solid. ^{1}H NMR (600 MHz, $CDCl_3$) δ 7.41 (2H, dd, J = 8.8, 5.5 Hz), 7.28 (1H, d, J = 8.1 Hz), 7.01 (2H, t, J = 8.8 Hz), 5.92 (1H, dd, J = 8.1, 1.2 Hz), 5.27 (1H, t, J = 7.4 Hz), 3.66 (2H, br s), 3.39 (4H, q, J = 7.8 Hz), 1.16 (6H, br s); ^{13}C NMR (151 MHz, $CDCl_3$) δ 162.4 (d, J = 245.8 Hz), 146.5, 142.3, 140.3, 137.0 (d, J = 3.0 Hz), 129.2 (d, J = 7.9 Hz), 114.9 (d, J = 21.2 Hz), 109.9, 108.0, 43.2, 31.3, 13.5 (br s); ^{19}F NMR (564 MHz, $CDCl_3$) δ –115.93 to –116.01 (m). Data in accordance with the literature.[4]

***N,N*-Diethyl-5-(4-(trifluoromethyl)phenyl)-3*H*-azepin-2-amine (S11)**

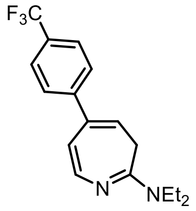

Following **GP2**, **S1** (730 mg, 3.0 mmol) and (4-(trifluoromethyl)phenyl)boronic acid (684 mg, 3.6 mmol), gave **S11** (422 mg, 46%) as a solid. R_f 0.40 [pentane:Et_2O (3:2) + Et_3N (1%)]; ^1H NMR (600 MHz, $CDCl_3$) δ 7.58 (2H, d, *J* = 8.5 Hz), 7.55 (2H, d, *J* = 8.5 Hz), 7.31 (1H, d, *J* = 8.1 Hz), 5.94 (1H, d, *J* = 8.0 Hz), 5.37 (1H, t, *J* = 7.5 Hz), 3.97 (1H, br s), 3.41 (4H, br s), 2.76 (1H, br s), 1.16 (6H, br s); ^{13}C NMR (126 MHz, $CDCl_3$) δ 146.4, 144.5, 142.8, 140.4, 129.3 (q, *J* = 32.4 Hz), 128.0, 125.3 (q, *J* = 3.6 Hz), 124.5 (q, *J* = 271.9 Hz), 109.8, 109.4, 43.4, 31.6, 13.5; ^{19}F NMR (564 MHz, $CDCl_3$) δ –62.35 (s); HRMS (ESI): found MH^+ 309.1572, $C_{17}H_{20}N_2F_3$ requires 309.1573.

5-[(1,1'-Biphenyl)-4-yl]-*N,N*-diethyl-3*H*-azepin-2-amine (S12)

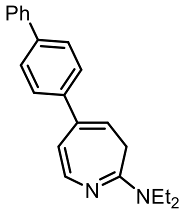

Following **GP2**, **S1** (486 mg, 2.0 mmol) and [1,1'-biphenyl]-4-ylboronic acid (475 mg, 2.4 mmol), gave **S12** (380 mg, 60%) as a solid. R_f 0.45 [pentane:Et_2O (3:2) + Et_3N (1%)]; ^1H NMR (600 MHz, $CDCl_3$) δ 7.61 (2H, d, *J* = 8.1 Hz), 7.58 (2H, d, *J* = 8.0 Hz), 7.54 (2H, d, *J* = 8.0 Hz), 7.44 (2H, t, *J* = 7.7 Hz), 7.36–7.32 (1H, m), 7.31 (1H, d, *J* = 8.0 Hz), 6.02 (1H, d, *J* = 8.1 Hz), 5.40 (1H, t, *J* = 7.4 Hz), 3.91 (1H, br s), 3.40 (4H, br s), 2.72 (1H, br s), 1.17 (6H, br s); ^{13}C NMR (151 MHz, $CDCl_3$) δ 146.6, 142.3, 141.1, 141.0, 140.2, 139.9, 128.9, 128.2, 127.3, 127.2, 127.1, 110.2, 108.6, 43.4, 31.6, 13.6; HRMS (EI): found M^+ 316.1933, $C_{22}H_{24}N_2$ requires 316.1934.

5-(4-(*tert*-Butyl)phenyl)-*N,N*-diethyl-3*H*-azepin-2-amine (S13)

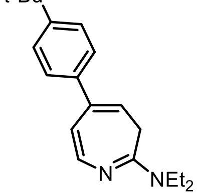

Following **GP2**, **S1** (730 mg, 3.0 mmol) and (4-(*tert*-butyl)phenyl)boronic acid (640 mg, 3.6 mmol), gave **S13** (400 mg, 45%) as a solid. R_f 0.50 [pentane:Et_2O (3:2) + Et_3N (1%)]; ^1H NMR (600 MHz, $CDCl_3$) δ 7.40 (2H, d, *J* = 8.2 Hz), 7.36 (2H, d, *J* = 8.4 Hz), 7.27 (1H, d, *J* = 8.1 Hz), 5.98 (1H, d, *J* = 8.1 Hz), 5.32 (1H, t, *J* = 7.4 Hz), 3.70 (1H, br s), 3.38 (4H, br s), 2.92 (1H, br s), 1.33 (9H, s), 1.14 (6H,

br s); ^{13}C NMR (151 MHz, $CDCl_3$) δ 150.4, 146.6, 142.1, 141.1, 138.1, 127.5, 125.3, 110.4, 108.2, 43.3, 34.6, 31.5, 31.4, 13.5; HRMS (ESI): found MH^+ 297.2318, $C_{20}H_{29}N_2$ requires 297.2325.

***N,N*-Diethyl-5-(4-methoxyphenyl)-3*H*-azepin-2-amine (S14)**

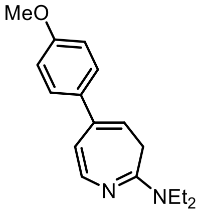

Following **GP2**, **S1** (145 mg, 0.6 mmol) and (4-methoxyphenyl)boronic acid (112 mg, 0.72 mmol), gave **S14** (128 mg, 79%) as a solid. ^{1}H NMR (600 MHz, $CDCl_3$) δ 7.39 (2H, d, J = 8.8 Hz), 7.27 (1H, d, J = 8.3 Hz), 6.88 (2H, d, J = 8.8 Hz), 5.95 (1H, dd, J = 8.1, 1.2 Hz), 5.26 (1H, t, J = 7.4 Hz), 3.81 (3H, s), 3.48–3.29 (4H, m), 1.75 (2H, s), 1.15 (6H, s); ^{13}C NMR (151 MHz, $CDCl_3$) δ 159.2, 146.8, 142.2, 140.8, 133.7, 128.9, 113.7, 110.5, 107.3, 55.5, 43.3, 31.4, 14.0. Data in accordance with the literature.[5]

5-(3,5-Dimethylphenyl)-*N,N*-diethyl-3*H*-azepin-2-amine (S15)

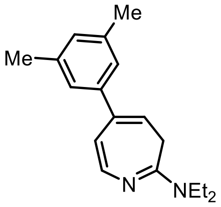

Following **GP2**, **S1** (484 mg, 2.0 mmol) and (3,5-dimethylphenyl)boronic acid (360 mg, 2.4 mmol), gave **S15** (313 mg, 58%) as an oil. R_f 0.35 [hexane:Et_2O (4:1) + Et_3N (2.5%)]; ^{1}H NMR (600 MHz, $CDCl_3$) δ 7.27 (1H, d, J = 8.1 Hz), 7.09 (2H, s), 6.93 (1H, s), 5.97 (1H, dd, J = 8.1, 1.3 Hz), 5.32 (1H, t, J = 7.4 Hz), 4.15 (2H, br s), 3.39 (4H, br s), 2.33 (6H, s), 1.15 (6H, br s); ^{13}C NMR (151 MHz, $CDCl_3$) δ 146.5, 142.0, 141.5, 140.9, 137.8, 129.0, 125.8, 110.5, 108.4, 43.3, 31.5, 21.5, 13.8; HRMS (ESI): found MH^+ 269.2018, $C_{18}H_{25}N_2$ requires 269.2012.

5-(*o*-Tolyl)-*N,N*-diethyl-3*H*-azepin-2-amine (S16)

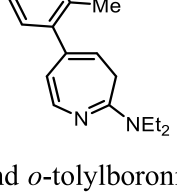

Following **GP2**, **S1** (350 mg, 1.4 mmol) and *o*-tolylboronic acid (235 mg, 1.7 mmol), gave **S16** (271 mg, 74%) as a solid. R_f 0.40 [pentane:EtOAc (8:2) + Et_3N (1%)]; ^{1}H NMR (600 MHz, $CDCl_3$) δ 7.22–7.12 (5H, m), 5.75 (1H, dd, J = 7.9, 1.2 Hz), 5.00 (1H, t, J = 7.3 Hz), 3.39 (4H, br s), 2.19 (3H, s), 1.15 (6H, br s); ^{13}C NMR (151 MHz, $CDCl_3$) δ 146.2, 142.3, 141.8, 140.4, 136.4, 130.0, 129.6, 127.2, 125.5,

112.2, 111.1, 43.4, 31.5, 20.3, 13.7; HRMS (ESI): found M^+ 254.1777, $C_{17}H_{22}N_2$ requires 254.1778. The CH_2 in the azepine core was not observed in the ^1H NMR spectrum but was confirmed by ^{13}C NMR and HRMS analysis.[7]

***N,N*-Diethyl-5-(4-methylthiophen-2-yl)-3*H*-azepin-2-amine (S17)**

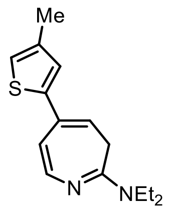

Following **GP2**, **S1** (300 mg, 1.2 mmol) and (4-methylthiophen-2-yl)boronic acid (210 mg, 1.4 mmol), gave **S17** (185 mg, 58%) as a solid. R_f 0.25 [pentane:EtOAc (9:1) + Et_3N (1%)]; ^1H NMR (600 MHz, $CDCl_3$) δ 7.22 (1H, d, J = 8.1 Hz), 6.88 (1H, s), 6.74 (1H, s), 6.00 (1H, d, J = 9.2 Hz), 5.38 (1H, t, J = 7.4 Hz), 3.38 (4H, br s), 2.23 (3H, s), 1.16 (6H, br s); ^{13}C NMR (126 MHz, $CDCl_3$) δ 147.1, 144.5, 142.4, 138.3, 134.8, 126.9, 119.7, 109.2, 107.5, 43.3, 31.4 (2C), 16.0; HRMS (EI): found M^+ 260.1342, $C_{15}H_{20}N_2S$ requires 260.1342. The CH_2 in the azepine core was not observed in the ^1H NMR spectrum but was confirmed by ^{13}C NMR and HRMS analysis.[7]

2.3 General Procedure for the Organolithium Addition to 3*H*-Azepin-2-amines – GP3

Unless commercially available, all organolithium were freshly prepared and titrated prior to use.[8]

Organolithium formation: a solution of the haloarene (1.0 equiv.) in anhydrous THF (0.5 M) was cooled to −78 °C and *n*-BuLi (1.6 M in *n*-hexane, 1.1 equiv.) was added dropwise. The mixture was stirred at −78 ºC for 30 min.

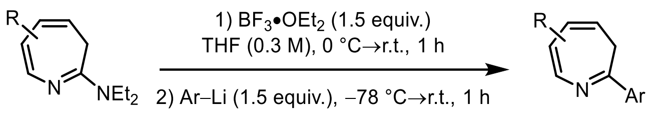

A solution of the 3*H*-azepin-2-amine (1.0 equiv.) in anhydrous THF (0.3 M) was cooled to 0 °C and treated with BF_3•OEt_2 (1.5 equiv.). The reaction was allowed to warm to room temperature and stirred for 1 h. The mixture was cooled to −78 °C and the organolithium solution (1.5 equiv.) was added dropwise. The reaction was stirred at –78 ºC for 30 min and then allowed to warm to room temperature and stirred for 30 min. The mixture was diluted with EtOAc and a saturated aqueous solution of K_2CO_3. The layers were separated, and the aqueous layer was extracted with EtOAc (x 3). The combined organic layers were washed with brine, dried ($MgSO_4$), filtered, and concentrated. The residue was purified by column chromatography on silica gel.

2,5-Diphenyl-3*H*-azepine (1)

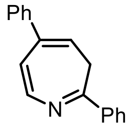

Following **GP3**, **S9** (1.0 g, 4.2 mmol) and PhLi (1.8 M in Bu_2O, 3.5 mL, 6.3 mmol), gave **1** (762 mg, 75%) as a solid. R_f 0.40 [pentane:Et_2O (9:1)]; ^{1}H NMR (600 MHz, $CDCl_3$,) δ 7.91 (2H, dd, *J* = 8.1, 1.2 Hz), 7.73 (1H, d, *J* = 8.1 Hz), 7.45–7.36 (5H, m), 7.34 (2H, t, *J* = 7.6 Hz), 7.29 (1H, t, *J* = 7.2 Hz), 6.51 (1H, dd, *J* = 8.3, 1.0 Hz), 5.57 (1H, t, *J* = 7.3 Hz), 4.44 (1H, br s), 1.72 (1H, br s); ^{13}C NMR (151 MHz, $CDCl_3$) δ 146.8, 142.3, 140.7, 140.2, 137.5, 130.1, 128.9, 128.7, 128.5, 127.9, 127.6, 117.1, 112.8, 34.2; HRMS (ESI): found MH^+ 246.1271, $C_{18}H_{16}N$ requires 246.1277.

5-(4-Fluorophenyl)-2-phenyl-3*H*-azepine (2)

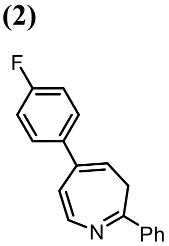

Following **GP3**, **S10** (1.1 g, 3.6 mmol) and PhLi (1.9 M in Bu_2O, 2.81 mL, 5.4 mmol), gave **2** (815 mg, 86%) as a solid. R_f 0.70 [pentane:Et_2O (9:1)]; ^{1}H NMR (600 MHz, $CDCl_3$ δ 7.93–7.90 (2H, m), 7.73 (1H, d, *J* = 8.2 Hz), 7.47–7.33 (5H, m), 7.02 (2H, t, *J* = 8.6 Hz), 6.45 (1H, d, *J* = 8.3 Hz), 5.50 (1H, t, *J* = 7.3 Hz), 4.43 (1H, br s), 1.68 (1H, br s); ^{13}C NMR (151 MHz, $CDCl_3$) δ 163.5, 161.8, 146.8, 142.5,

139.8, 137.4, 136.3 (d, J = 3.6 Hz), 130.2, 129.5 (d, J = 8.5 Hz), 128.9 (d, J = 28.5 Hz), 116.8, 115.2 (d, J = 21.2 Hz), 112.5, 34.0; ^{19}F NMR (564 MHz, $CDCl_3$) δ –115.29 (s); HRMS (ESI): found MH^+ 264.1177, $C_{18}H_{15}NF$ requires 264.1183.

2-Phenyl-5-4-[(trifluoromethyl)phenyl]-3*H*-azepine (3)

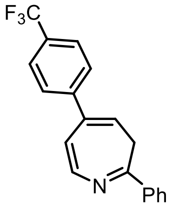

Following **GP3**, **S11** (338 mg, 1.1 mmol) and PhLi (1.9 M in Bu_2O, 0.87 mL, 1.7 mmol), gave **3** (241 mg, 70%) as a solid. R_f 0.65 [pentane:Et_2O (9:1)]; ^{1}H NMR (600 MHz, $CDCl_3$) δ 7.95–7.90 (2H, m), 7.77 (1H, d, J = 8.2 Hz), 7.59 (2H, d, J = 8.2 Hz), 7.52 (2H, d, J = 8.2 Hz), 7.45–7.36 (3H, m), 6.47 (1H, dd, J = 8.2, 1.1 Hz), 5.61 (1H, t, J = 7.4 Hz), 4.48 (1H, br s), 1.73 (1H, br s); ^{13}C NMR (151 MHz, $CDCl_3$) δ 146.9, 143.6, 142.8, 139.6, 137.2, 130.4, 129.8 (q, J = 32.5 Hz), 129.0, 128.8, 128.1, 125.5 (q, J = 3.7 Hz), 124.4 (q, J = 272.0 Hz), 116.2, 114.2, 34.3; ^{19}F NMR (564 MHz, $CDCl_3$) δ –62.45 (s); HRMS (ESI): found MH^+ 314.1140, $C_{19}H_{15}NF_3$ requires 314.1151.

5-([1,1'-Biphenyl]-4-yl)-2-phenyl-3*H*-azepine (4)

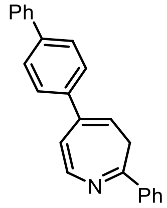

Following **GP3**, **S12** (376 mg, 1.2 mmol) and PhLi (1.9 M in Bu_2O, 0.95 mL, 1.8 mmol), gave **4** (300 mg, 79%) as a solid. R_f 0.50 [pentane:Et_2O (9:1)]; ^{1}H NMR (600 MHz, $CDCl_3$) δ 7.93 (2H, d, J = 6.7 Hz), 7.76 (1H, d, J = 8.2 Hz), 7.63–7.55 (4H, m), 7.50 (2H, d, J = 8.4 Hz), 7.47–7.37 (5H, m), 7.34 (1H, t, J = 7.4 Hz), 6.55 (1H, d, J = 8.2 Hz), 5.63 (1H, t, J = 7.3 Hz), 4.46 (1H, br s), 1.74 (1H, br s); ^{13}C NMR (151 MHz, $CDCl_3$) δ 146.8, 142.4, 140.8, 140.5, 140.2, 139.1, 137.5, 130.2, 129.0, 128.9, 128.7, 128.3, 127.5, 127.2, 127.2, 116.9, 112.9, 34.3; HRMS (ESI): found MH^+ 322.1581, $C_{24}H_{20}N$ requires 322.1590.

5-(4-(*tert*-Butyl)phenyl)-2-phenyl-3*H*-azepine (5)

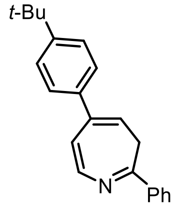

Following **GP3**, **S13** (320 mg, 1.1 mmol) and PhLi (1.9 M in Bu_2O, 0.8 mL, 1.7 mmol), gave **5** (296 mg, 90%) as a solid. R_f 0.65 [pentane:Et_2O (9:1)]; ^{1}H NMR (600 MHz, $CDCl_3$) δ 7.89 (2H, dd, *J* = 8.2, 1.6 Hz), 7.72 (1H, d, *J* = 8.2 Hz), 7.41–7.37 (3H, m), 7.37 (4H, br s), 6.51 (1H, dd, *J* = 8.3, 1.3 Hz), 5.55 (1H, t, *J* = 7.4 Hz), 4.42 (1H, br s), 1.71 (1H, br s), 1.32 (9H, s); ^{13}C NMR (151 MHz, $CDCl_3$) δ 150.7, 146.7, 142.2, 140.4, 137.6, 137.3, 130.1, 128.9, 128.7, 127.5, 125.5, 117.2, 112.4, 34.7, 34.3, 31.5; HRMS (ESI): found MH^+ 302.1902, $C_{22}H_{24}N$ requires 302.1903.

5-(4-Methoxyphenyl)-2-phenyl-3*H*-azepine (6)

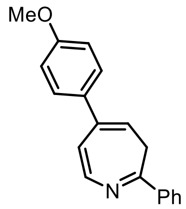

Following **GP3**, **S14** (650 mg, 2.4 mmol) and PhLi (1.9 M in Bu_2O, 1.89 mL, 3.6 mmol), gave **6** (627 mg, 95%) as a solid. R_f 0.70 [pentane:EtOAc (9:1)]; ^{1}H NMR (600 MHz, $CDCl_3$) δ 7.97–7.88 (2H, m), 7.72 (1H, d, *J* = 8.2 Hz), 7.43–7.33 (5H, m), 6.88 (2H, d, *J* = 8.7 Hz), 6.49 (1H, d, *J* = 8.3 Hz), 5.50 (1H, t, *J* = 7.4 Hz), 4.41 (1H, br s), 3.81 (3H, s), 1.69 (1H, br s); ^{13}C NMR (151 MHz, $CDCl_3$) δ 159.4, 146.8, 142.2, 140.1, 137.5, 132.9, 130.1, 129.0, 128.9, 128.7, 117.3, 113.9, 111.6, 55.4, 34.1; HRMS (ESI): found MH^+ 276.1373, $C_{19}H_{18}NO$ requires 276.1383.

5-(3,5-Dimethylphenyl)-2-phenyl-3*H*-azepine (7)

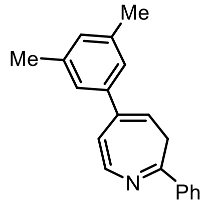

Following **GP3**, **S15** (268 mg, 1.0 mmol) and PhLi (1.9 M in Bu_2O, 0.79 mL, 1.5 mmol), gave **7** (216 mg, 75%) as an oil. R_f 0.70 [pentane:EtOAc (9:1)]; ^{1}H NMR (600 MHz, $CDCl_3$) δ 8.01–7.82 (2H, m), 7.72 (1H, d, *J* = 8.2 Hz), 7.43–7.34 (3H, m), 7.05 (2H, s), 6.94 (1H, s), 6.50 (1H, d, *J* = 8.2 Hz), 5.55 (1H, t, *J* = 7.3 Hz), 4.43 (1H, br s), 2.32 (6H, s), 1.69 (1H, br s); ^{13}C NMR (151 MHz, $CDCl_3$) δ 146.6, 142.1, 140.8, 140.1, 138.0, 137.5, 130.1, 129.2, 128.9, 128.7, 125.8, 117.3, 112.6, 34.2, 21.4; HRMS (ESI): found MH^+ 274.1580, $C_{20}H_{20}N$ requires 274.1590.

2-Phenyl-5-(*o*-tolyl)-3*H*-azepine (8)

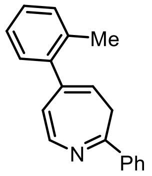

Following **GP3**, **S16** (270 mg, 1.1 mmol) and PhLi (1.9 M in Bu_2O, 0.84 mL, 1.8 mmol), gave **8** (198 mg, 72%) as a solid. R_f 0.65 [pentane:Et_2O (9:1)]; ^{1}H NMR (600 MHz, $CDCl_3$) δ 7.91 (2H, dd, J = 8.1, 1.5 Hz), 7.62 (1H, d, J = 8.1 Hz), 7.41 (3H, m), 7.22–7.12 (4H, m), 6.28 (1H, d, J = 8.1 Hz), 5.25 (1H, t, J = 7.2 Hz), 4.39 (1H, br s), 2.15 (3H, s), 1.74 (1H, br s); ^{13}C NMR (151 MHz, $CDCl_3$) δ 146.7, 141.4, 140.8, 140.7 137.7, 136.3, 130.2, 130.0, 129.8, 128.9, 128.7, 127.6, 125.7, 119.0, 115.3, 34.2, 20.4; HRMS (EI): found M^+ 259.1347, $C_{19}H_{17}N$ requires 259.1357.

5-(4-Methylthiophen-2-yl)-2-phenyl-3*H*-azepine (9)

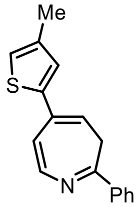

Following **GP3**, **S17** (185 mg, 0.7 mmol) and PhLi (1.9 M in Bu_2O, 0.6 mL, 1.1 mmol), gave **9** (122 mg, 65%) as a solid. R_f 0.42 [pentane:Et_2O (9:1)]; ^{1}H NMR (600 MHz, $CDCl_3$) δ 7.90 (2H, dd, J = 7.9, 1.8 Hz), 7.67 (1H, d, J = 8.2 Hz), 7.43–7.37 (3H, m), 6.87 (1H, d, J = 1.4 Hz), 6.76 (1H, p, J = 1.2 Hz), 6.53 (1H, dd, J = 8.2, 1.3 Hz), 5.60 (1H, t, J = 7.4 Hz), 4.37 (1H, br s), 2.22 (3H, s), 1.75 (1H, br s); ^{13}C NMR (151 MHz, $CDCl_3$) δ 147.5, 143.2, 142.4, 138.4, 137.3, 134.1, 130.3, 128.9, 128.7, 127.4, 120.3, 116.0, 112.0, 34.0, 15.9; HRMS (ESI): found MH^+ 266.0999, $C_{17}H_{16}NS$ requires 266.0998.

2-(4-Methoxyphenyl)-5-phenyl-3*H*-azepine (10)

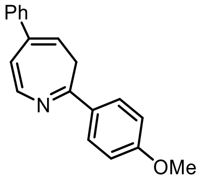

Following **GP3**, **S9** (407 mg, 1.7 mmol) and (4-methoxyphenyl)lithium (0.4 M in THF, 5.5 mL, 2.5 mmol), gave **10** (232 mg, 50%) as a solid. R_f 0.27 [pentane:Et_2O (95:5)]; ^{1}H NMR (400 MHz, $CDCl_3$) δ 7.89 (2H, d, J = 8.9 Hz), 7.70 (1H, d, J = 8.2 Hz), 7.42 (2H, d, J = 7.7 Hz), 7.32 (2H, t, J = 7.3 Hz), 7.29 (1H, d, J = 7.3 Hz), 6.91 (2H, d, J = 8.9 Hz), 6.47 (1H, d, J = 8.2 Hz), 5.53 (1H, t, J = 7.3 Hz) 4.43 (1H, br s), 3.84 (3H, s), 1.74 (1H, br s); ^{13}C NMR (151 MHz, $CDCl_3$) δ 161.3, 146.3, 142.4, 140.6, 140.2, 130.6, 130.0, 128.5, 127.8, 127.5, 116.5, 114.1, 112.9, 55.5, 33.9; HRMS (ESI): found MH^+ 276.1392, $C_{19}H_{18}NO$ requires 276.1383.

5-Phenyl-2-(*m*-tolyl)-3*H*-azepine (11)

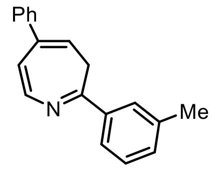

Following **GP3**, **S9** (500 mg, 2.1 mmol) and *m*-tolyllithium (0.4 M in THF, 7.8 mL, 3.1 mmol), gave **11** (381 mg, 70%) as a solid. R_f 0.49 [pentane:Et_2O (9:1)]; ^1H NMR (600 MHz, $CDCl_3$) δ 7.75–7.72 (2H, m), 7.71 (1H, d, *J* = 7.9 Hz), 7.46–7.39 (2H, m), 7.34 (2H, t, *J* = 7.4 Hz), 7.32–7.27 (2H, m), 7.19 (1H, d, *J* = 7.5 Hz), 6.50 (1H, dd, *J* = 8.2, 1.3 Hz), 5.57 (1H, t, *J* = 7.3), 4.45 (1H, s), 2.38 (3H, s), 1.71 (1H, br s); ^{13}C NMR ($CDCl_3$, 151 MHz) δ 147.0, 142.3, 140.7, 140.2, 138.5, 137.4, 131.0, 129.7, 128.6, 128.5, 127.9, 127.6, 126.0, 117.0, 112.9, 34.3, 21.5. HRMS (ESI): found MH^+ 260.1441, $C_{19}H_{18}N$ requires 260.1434.

5-Phenyl-2-(4-(trifluoromethyl)phenyl)-3*H*-azepine (12)

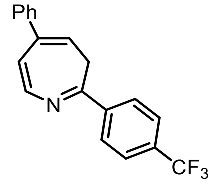

Following **GP3**, **S9** (500 mg, 2.1 mmol) and (4-(trifluoromethyl)phenyl)lithium (0.3 M in THF, 10.3 mL, 3.1 mmol), gave **12** (372 mg, 57%) as a solid. R_f 0.37 [pentane:Et_2O (95:5)]; ^1H NMR (600 MHz, $CDCl_3$) δ 8.02 (2H, d, *J* = 8.2 Hz), 7.76 (1H, d, *J* = 8.2 Hz), 7.65 (2H, d, *J* = 8.3 Hz), 7.42 (2H, d, *J* = 7.3 Hz), 7.36 (2H, t, *J* = 7.4 Hz), 7.31 (1H, t, *J* = 7.3 Hz), 6.57 (1H, d, *J* = 8.2 Hz), 5.59 (1H, t, *J* = 7.3 Hz), 4.41 (1H, s), 1.74 (1H, s); ^{13}C NMR ($CDCl_3$, 151 MHz) δ 144.9, 142.1, 141.1, 140.9, 139.9, 131.6 (q, *J* = 32.6 Hz), 129.2, 128.6, 127.9, 127.8, 125.7 (q, *J* = 3.8 Hz), 124.1 (q, *J* = 272 Hz), 117.8, 112.5, 34.1. ^{19}F NMR ($CDCl_3$, 565 MHz) δ –62.79 (s). HRMS (ESI): found MH^+ 314.1161, $C_{19}H_{15}NF_3$ requires 314.1151.

2-(6-Methoxypyridin-3-yl)-5-phenyl-3*H*-azepine (13)

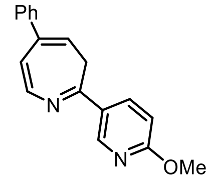

Following **GP3**, **S9** (300 mg, 1.3 mmol) and (6-methoxypyridin-3-yl)lithium (0.25 M in THF, 7.6 mL, 1.9 mmol), gave **13** (223 mg, 65%) as a solid. R_f 0.25 [pentane:EtOAc (95:5)]; ^1H NMR (400 MHz, $CDCl_3$) δ 8.72 (1H, d, *J* = 2.4 Hz), 8.14 (1H, dd, *J* = 8.8, 2.4 Hz), 7.70 (1H, d, *J* = 8.2 Hz), 7.42 (2H, d, *J* = 8.0 Hz), 7.35 (2H, t, *J* = 7.5 Hz), 7.30 (1H, d, *J* = 7.2 Hz), 6.75 (1H, d, *J* = 8.8 Hz), 6.49 (1H, d, *J* = 8.3 Hz), 5.53 (1H, t, *J* = 7.3 Hz), 4.36 (1H, br s), 3.97 (3H, s), 1.75 (1H, br s); ^{13}C NMR (151 MHz,

$CDCl_3$) δ 165.1, 148.0, 144.1, 142.4, 140.9, 140.0, 139.4, 128.5, 127.9, 127.7, 126.8, 117.1, 112.7, 111.2, 54.0, 33.7. HRMS (EI): found M^+ 276.1250, $C_{18}H_{16}N_2O$ requires 276.1257.

2-(Furan-2-yl)-5-phenyl-3*H*-azepine (14)

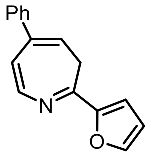

Following **GP3, S9** (288 mg, 1.2 mmol) and furan-2-yllithium (0.45 M in THF, 4.0 mL, 1.8 mmol.), gave **14** (160 mg, 57%) as a solid. R_f 0.20 [pentane:Et_2O (8:2)]; ^1H NMR (600 MHz, $CDCl_3$) δ 7.69 (1H, d, J = 7.2 Hz), 7.51 (1H, s), 7.41 (2H, d, J = 8.3 Hz), 7.34 (2H, t, J = 7.7 Hz), 7.29 (1H, t, J = 7.2 Hz), 6.98 (1H, d, J = 3.6 Hz), 6.51–6.44 (2H, m), 5.52 (1H, t, J = 7.6 Hz), 4.27 (1H, br s), 1.98 (1H, br s); ^{13}C NMR (151 MHz, $CDCl_3$) δ 152.4, 145.1, 142.4, 141.0, 140.1, 137.4, 128.5, 127.9, 127.7, 117.7, 114.0, 113.4, 112.3, 33.2. HRMS (EI): found M^+ 235.0986, $C_{16}H_{13}NO$ requires 235.0992.

5-Methyl-2-phenyl-3*H*-azepine (15)

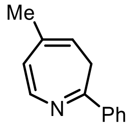

Following **GP3**, **S2** (557 mg, 3.1 mmol) and PhLi (1.7 M in Bu_2O, 2.8 mL, 4.7 mmol), gave **15** (414 mg, 72%) as an oil. R_f 0.50 [pentane:Et_2O (95:5)]; ^1H NMR (600 MHz, $CDCl_3$) δ 7.88 (2H, d, J = 6.4 Hz), 7.48 (1H, d, J = 8.2 Hz), 7.42–7.35 (3H, m), 6.15 (1H, d, J = 8.2 Hz), 5.10 (1H, t, J = 7.1 Hz), 4.10 (1H, br s), 1.95 (3H, s), 1.69 (1H, br s); ^{13}C NMR (151 MHz, $CDCl_3$) δ 146.9, 140.4, 137.7, 136.4, 129.9, 128.8, 128.7, 119.8, 113.4, 33.6, 21.1; HRMS (EI): found M^+ 183.1042, $C_{13}H_{13}N$ requires 183.1043.

5-Ethyl-2-phenyl-3*H*-azepine (16)

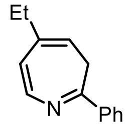

Following **GP3**, **S3** (231 mg, 1.2 mmol) and PhLi (1.9 M in Bu_2O, 0.9 mL, 1.8 mmol), gave **16** (91 mg, 38%) as an oil. R_f 0.54 [pentane:Et_2O (9:1)]; ^1H NMR (400 MHz, $CDCl_3$) δ 7.89 (2H, dd, J = 8.0, 1.8 Hz), 7.51 (1H, d, J = 8.2 Hz), 7.44–7.34 (3H, m), 6.18 (1H, d, J = 8.2 Hz), 5.10 (1H, t, J = 7.0 Hz), 4.11 (2H, br s), 2.26 (2H, q, J = 7.5 Hz), 1.05 (3H, t, J = 7.5 Hz); ^{13}C NMR (101 MHz, $CDCl_3$) δ 147.1, 142.5, 140.8, 137.8, 129.9, 128.8, 128.7, 119.0, 111.9, 33.5, 28.4, 14.5; HRMS (EI): found M^+ 197.1205, $C_{14}H_{15}N$ requires 197.1199.

5-*iso*-Propyl-2-phenyl-3*H*-azepine (17)

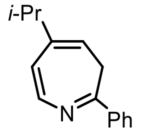

Following **GP3**, **S4** (620 mg, 3.0 mmol) and PhLi (1.9 M in Bu_2O, 2.4 mL, 4.5 mmol), gave **17** (596 mg, 94%) as an oil. R_f 0.60 [pentane:Et_2O (9:1)]; ^1H NMR (600 MHz, $CDCl_3$) δ 7.89 (2H, d, J = 6.7 Hz), 7.54 (1H, d, J = 8.3 Hz), 7.46–7.32 (3H, m), 6.24 (1H, d, J = 9.0 Hz), 5.11 (1H, t, J = 7.0 Hz), 4.15 (1H, br s), 2.52 (1H, hept, J = 6.9 Hz), 1.60 (1H, br s), 1.07 (6H, d, J = 6.9 Hz); ^{13}C NMR (151 MHz, $CDCl_3$) δ 147.4, 146.9, 141.1, 137.8, 129.9, 128.8, 128.7, 117.8, 110.7, 33.6, 33.4, 23.0; HRMS (ESI): found MH^+ 212.1424, $C_{15}H_{18}N$ requires 212.1434.

5-Benzyl-2-phenyl-3*H*-azepine (18)

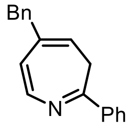

Following **GP3**, **S5** (302 mg, 1.2 mmol) and PhLi (1.6 M in Bu_2O, 1.1 mL, 1.8 mmol), gave **18** (215 mg, 70%) as an oil. ^1H NMR (400 MHz, $CDCl_3$) δ 7.98–7.90 (2H, m), 7.55 (1H, d, J = 8.2 Hz), 7.49–7.39 (3H, m), 7.37–7.30 (2H, m), 7.24 (1H, t, J = 7.3 Hz), 7.16 (2H, d, J = 7.4 Hz), 6.18 (1H, d, J = 8.2 Hz), 5.17 (1H, t, J = 7.1 Hz), 4.12 (2H, br s), 3.63 (2H, s); ^{13}C NMR (101 MHz, $CDCl_3$) δ 147.2, 141.0, 140.1, 139.6, 137.6, 129.9, 128.9, 128.8, 128.6, 128.4, 126.2, 118.8, 114.4, 41.3, 33.6. Data in accordance with the literature.[5]

2-Phenyl-5-(trifluoromethyl)-3*H*-azepine (19)

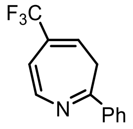

Following **GP3**, **S6** (559 mg, 2.4 mmol) and PhLi (1.8 M in Bu_2O, 2.0 mL, 1.5 mmol), gave **19** (364 mg, 64%) as a solid. ^1H NMR (400 MHz, $CDCl_3$) δ 7.93–7.87 (2H, m), 7.74 (1H, d, J = 8.3 Hz), 7.47–7.41 (3H, m), 6.37 (1H, dd, J = 8.3, 1.3 Hz), 5.82 (1H, tq, J = 7.4, 1.3 Hz), 4.19 (1H, br s), 1.86 (1H, br s); ^{13}C NMR (101 MHz, $CDCl_3$) δ 146.6, 143.4, 136.5, 130.8, 130.7, 129.0, 128.8, 123.4 (q, J = 273.4 Hz), 117.1 (q, J = 4.7 Hz), 110.2, 33.3; ^{19}F NMR (376 MHz, $CDCl_3$) δ −64.60 (s). Data in accordance with the literature.[5]

2-*iso*-Propyl-5-phenyl-3*H*-azepine (20)

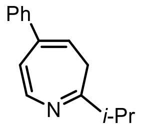

Following **GP3**, **S9** (361 mg, 1.5 mmol) and *i*-PrLi (0.7 M in pentane, 3.2 mL, 2.3 mmol), gave **20** (140 mg, 44%) as an oil. R_f 0.42 [pentane:Et_2O (9:1)]; ^{1}H NMR (400 MHz, $CDCl_3$,) δ 7.49 (1H, d, *J* = 8.3 Hz), 7.41 (2H, d, *J* = 8.3 Hz), 7.36 (2H, t, *J* = 7.1 Hz), 7.29 (1H, t, *J* = 6.7 Hz), 6.39 (1H, d, *J* = 8.5 Hz), 5.44 (1H, t, *J* = 7.4 Hz), 3.68 (1H, br s), 2.65 (1H, hept, *J* = 6.6 Hz), 1.43 (1H, br s), 1.16 (6H, d, *J* = 6.8 Hz); ^{13}C NMR (151 MHz, $CDCl_3$) δ 161.0, 141.5, 140.4, 139.4, 128.5, 127.9, 127.5, 116.5, 112.3, 37.4, 34.8, 21.0; HRMS (ESI): found MH^+ 212.1430, $C_{15}H_{18}N$ requires 212.1434.

2,5-Diisopropyl-3*H*-azepine (21)

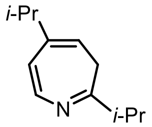

Following **GP3**, **S4** (500 mg, 2.4 mmol) and *i*-PrLi (0.7 M in pentane, 5.2 mL, 3.6 mmol), gave **21** (292 mg, 68%) as an oil. R_f 0.65 [pentane:Et_2O (9:1)]; ^{1}H NMR (600 MHz, $CDCl_3$) δ 7.28 (1H, d, *J* = 8.3 Hz), 6.11 (1H, d, *J* = 8.3 Hz), 4.95 (1H, t, *J* = 6.9 Hz), 3.39 (1H, br s), 2.60 (1H, hept, *J* = 6.9 Hz), 2.46 (1H, hept, *J* = 6.9 Hz), 1.31 (1H, br s), 1.13 (6H, d, *J* = 6.9 Hz), 1.04 (6H, d, *J* = 6.9 Hz); ^{13}C NMR (151 MHz, $CDCl_3$) δ 161.4, 145.6, 140.2, 117.0, 110.2, 37.3, 34.0, 33.6, 23.0, 21.1; HRMS (ESI): found MH^+ 178.1584, $C_{12}H_{20}N$ requires 178.1590.

2,6-Diphenyl-3*H*-azepine (22)

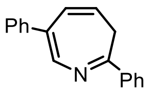

Following **GP3**, **S7** (96 mg, 0.4 mmol) and PhLi (1.9 M in Bu_2O, 0.32 mL, 0.6 mmol), gave **22** (90 mg, 61%) as a solid. ^{1}H NMR (400 MHz, $CDCl_3$) δ 7.98 (1H, s), 7.96–7.91 (2H, m), 7.57–7.52 (2H, m), 7.45–7.39 (5H, m), 7.34 (1H, t, *J* = 7.4 Hz), 6.58 (1H, d, *J* = 8.9 Hz), 5.58–5.48 (1H, m); ^{13}C NMR (101 MHz, $CDCl_3$) δ 145.4, 140.7, 138.9, 137.2, 130.0, 129.5, 128.85, 128.8, 128.7, 128.6, 127.7, 127.2, 117.0, 34.6. The CH_2 in the azepine core was not observed in the ^{1}H NMR spectrum but was confirmed by ^{13}C NMR and HRMS analysis.[7] Data in accordance with the literature.[5]

6-Methyl-2,5-diphenyl-3*H*-azepine (23)

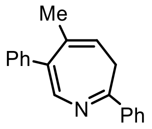

Following **GP3**, **S8** (400 mg, 1.6 mmol) and PhLi (1.9 M in Bu_2O, 1.30 mL, 2.5 mmol), gave **23** (401 mg, 97%) as a solid. R_f 0.70 [pentane:Et_2O (9:1)]; ^1H NMR (600 MHz, $CDCl_3$) δ 7.94 (2H, d, J = 6.7 Hz), 7.62 (1H, s), 7.41–7.36 (7H, m), 7.35–7.30 (1H, m), 5.53 (1H, t, J = 7.2 Hz), 4.25–4.15 (1H, m), 1.87–1.81 (1H, m), 1.80 (3H, s); ^{13}C NMR (151 MHz, $CDCl_3$) δ 140.5, 140.2, 139.7, 139.6, 137.4, 135.2, 133.2, 130.1, 129.6, 128.8, 128.4, 127.2, 115.8, 33.7, 20.6; HRMS (ESI): found MH^+ 260.1429, $C_{19}H_{18}N$ requires 260.1434.

3 Chiral Ligands Synthesis

General Procedure for the Preparation of Chiral Phosphoramidite – GP4

Chiral phosphoramidites **L2-4**, **LS13-16** and **LS18-24** were synthesized following general procedure **GP4** adapted from the literature.[9,10] Chiral phosphoramidites **L5**, **LS17**, **LS25** and **LS26** were repurified from commercially available ligands prior to use.

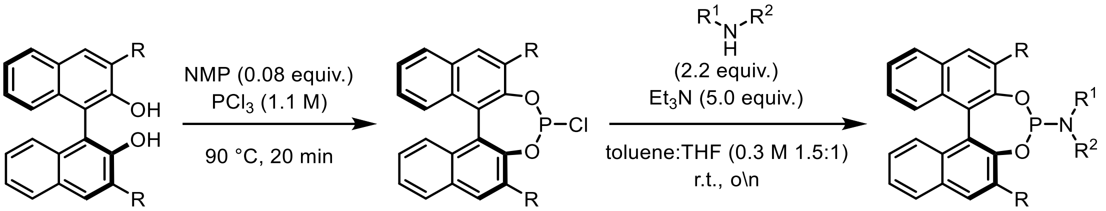

An oven dried two-neck round bottom flask was charged with *(S)*-(−)-1,1′-bi(2-naphthol) (1.0 equiv.). The flask was evacuated and filled with N_2 (x 3). Then PCl_3 (1.1 M) and NMP (0.08 equiv.) were added. The resulting mixture was refluxed under a constant flow of N_2. After 20 minutes, the reaction mixture was cooled to room temperature. The excess of PCl_3 was removed by high vacuum distillation under N_2. Residual amounts of PCl_3 were co-distilled with toluene (x 3) under high vacuum. The remaining solid was redissolved in degassed THF (0.2 M). In parallel, a round bottom flask was charged with the corresponding amine (2.2 equiv.), Et_3N (5.0 equiv.) and toluene (0.8 M). The stock solution of *(S)*-(−)-binol chlorophosphite was added slowly to the reaction mixture and stirred overnight at r.t.. The solids were then filtered and the filtrate concentrated and purified on neutral alumina (pentane:EtOAc 90:10). The residue was triturated with MeOH (x 5) and dried under high vacuum. The purity of the ligands was determined by chiral HPLC and NMR.

***(S)*-(+)-4-Dinaphtho[2,1-d:1′,2′-f][1,3,2]dioxaphosphepin-4-yl-piperidine (L2)**

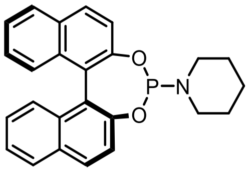

Following **GP4**, *(S)-(−)*-1,1′-Bi(2-naphthol) (10 g, 35 mmol) and piperidine (7.6 mL, 77 mmol.), gave **L2** (3.6 g, 26%) as a solid. ^{1}H NMR (400 MHz, $CDCl_3$) δ 7.97 (1H, d, J = 8.8 Hz), 7.91 (3H, dd, J = 8.4, 3.2 Hz), 7.53 (1H, d, J = 8.8 Hz), 7.46–7.37 (4H, m), 7.34 (1H, d, J = 8.5 Hz), 7.31–7.26 (1H, m), 7.25–7.20 (1H, m), 3.06–2.77 (4H, m), 1.67–1.51 (2H, m), 1.49–1.35 (4H, m); ^{13}C NMR (151 MHz, $CDCl_3$) δ 150.1 (d, J = 5.0 Hz), 149.7, 133.0, 132.8, 131.5, 130.8, 130.3, 129.9, 128.5, 128.4, 127.15, 127.11, 126.15, 126.11, 124.8, 124.6, 124.1 (d, J = 4.9 Hz), 122.8 (d, J = 2.3 Hz), 122.4, 122.2 (d, J = 1.4 Hz), 45.5, 45.4, 27.12, 27.09, 25.1; ^{31}P NMR ($CDCl_3$, 243 MHz) δ 145.60. Data in accordance with the literature.[9]

(11bS)-*N,N*-Dimethyldinaphtho[2,1-d:1',2'-f][1,3,2]dioxaphosphepin-4-amine (L3)

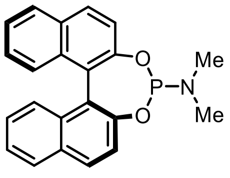

Following **GP4**, *(S)-(−)*-1,1′-Bi(2-naphthol) (3.0 g, 10.4 mmol) and Me_2NH (1.2 mL, 23 mmol), gave **L3** (1.1g, 28%) as a solid. ^{1}H NMR (400 MHz, $CDCl_3$) δ 7.96 (1H, d, *J* = 8.8 Hz), 7.91 (3H, dd, *J* = 8.4, 4.8 Hz), 7.50 (1H, d, *J* = 8.3 Hz), 7.47–7.37 (4H, m), 7.39–7.26 (1H, m), 7.31–7.26 (1H, m), 7.27–7.20 (1H, m), 2.55 (6H, d, *J* = 9.0 Hz); ^{13}C NMR ($CDCl_3$, 151 MHz) δ 150.1 (d, *J* = 4.8 Hz), 149.6, 133.0 (d, *J* = 1.5 Hz), 132.7, 131.5, 130.9, 130.4, 130.1, 128.5, 128.4, 127.13, 127.06, 126.2, 124.9, 124.7, 124.1 (d, *J* = 5.0 Hz), 122.9 (d, *J* = 2.2 Hz), 122.2, 122.1 (d, *J* = 1.8 Hz), 36.1 (d, *J* = 21.0 Hz); ^{31}P NMR (243 MHz, $CDCl_3$) δ 148.71. Data in accordance with the literature.[9]

(11bS)-*N,N*-Diethyldinaphtho[2,1-d:1',2'-f][1,3,2]dioxaphosphepin-4-amine (LS13)

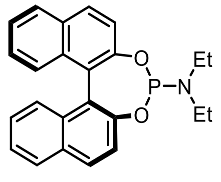

Following **GP4**, *(S)-(−)*-1,1′-Bi(2-naphthol) (3.0 g, 10.4 mmol) and Et_2NH (2.4 mL, 23 mmol) gave **LS13** (1.1g, 29%) as a solid. ^{1}H NMR (400 MHz, $CDCl_3$) δ 7.96 (1H, d, *J* = 8.8 Hz), 7.90 (3H, dd, *J* = 8.4, 6.2 Hz), 7.51 (1H, d, *J* = 8.8 Hz), 7.46–7.38 (3H, m), 7.37–7.31 (2H, m), 7.30–7.27 (1H, m), 7.25–7.18 (1H, m), 3.05 (2H, dp, *J* = 15.5, 7.4 Hz), 2.85 (2H, dp, *J* = 21.1, 7.0 Hz), 1.05 (6H, d, *J* = 14.1 Hz); ^{13}C NMR (151 MHz, $CDCl_3$) δ 150.2, 149.8, 133.0, 132.8, 131.5, 130.8, 130.3, 129.9, 128.44, 128.4, 127.2, 127.1, 126.13, 126.08, 124.8, 124.6, 124.2, 122.6, 122.3, 122.1, 38.4 (d, *J* = 21.6 Hz), 14.8 (d, *J* = 2.6 Hz); ^{31}P NMR (162 MHz, $CDCl_3$) δ 149.73. Data in accordance with the literature.[9]

(11bS)-*N,N*-Diisopropyldinaphtho[2,1-d:1',2'-f][1,3,2]dioxaphosphepin-4-amine (L4)

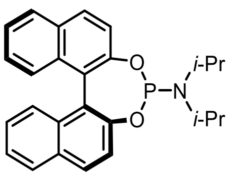

Following **GP4**, *(S)-(−)*-1,1′-Bi(2-naphthol) (3.0 g, 10.4 mmol) and *i*-Pr_2NH (3.2 mL, 23 mmol), gave **L4** (0.67g, 16%) as a solid. ^{1}H NMR (600 MHz, $CDCl_3$) δ 7.96 (1H, d, *J* = 8.7 Hz), 7.90 (3H, dd, *J* = 8.6, 4.6 Hz), 7.51 (1H, d, *J* = 8.8, 2.0 Hz), 7.47–7.36 (4H, m), 7.31 (1H, d, *J* = 8.6 Hz), 7.26–7.18 (2H, m), 3.52–3.32 (2H, m), 1.23 (6H, d, *J* = 6.7 Hz), 1.19 (6H, d, *J* = 6.1 Hz); ^{13}C NMR (151 MHz, $CDCl_3$) δ 150.6 (d, *J* = 6.7 Hz), 150.4, 133.0, 132.9, 131.4, 130.6, 130.3, 129.5, 128.4, 128.3, 127.3, 127.25, 126.0, 125.9, 124.7, 124.4, 124.2 (d, *J* = 5.2 Hz), 122.6, 122.6 (d, *J* = 2.0 Hz), 122.0 (d, *J* = 1.9 Hz),

44.9 (d, J = 12.7 Hz), 24.6 (d, J = 8.5 Hz); ^{31}P NMR ($CDCl_3$, 243 MHz) δ 151.73. Data in accordance with the literature. [9]

(11bS)-*N*-Ethyl-*N*-methyldinaphtho[2,1-d:1',2'-f][1,3,2]dioxaphosphepin-4-amine (LS14)

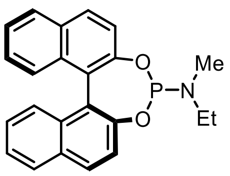

Following **GP4**, *(S)-(−)*-1,1′-Bi(2-naphthol) (3.0 g, 10.4 mmol) and *N*-methylethylamine (2.0 mL, 23 mmol), gave **LS14** (1.3 g, 33%) as a solid. ^{1}H NMR (600 MHz, $CDCl_3$) δ 7.96 (1H, d, J = 8.8 Hz), 7.94–7.86 (3H, m), 7.51 (1H, d, J = 9.2 Hz), 7.45–7.37 (4H, m), 7.35 (1H, d, J = 8.6 Hz), 7.3–7.27 (1H, m), 7.25–7.17 (1H, m), 3.24 – 3.13 (1H, m), 3.05–2.79 (1H, m), 2.39 (3H, d, J = 6.5 Hz), 1.15 (3H, t, J = 7.1 Hz); ^{13}C NMR (151 MHz, $CDCl_3$) δ 150.2 (d, J = 4.8 Hz), 149.7, 133.0, 132.8, 131.5, 130.8, 130.4, 130.1, 128.5, 128.4, 127.2, 127.1, 126.2, 124.9, 124.7, 124.1 (d, J = 5.1 Hz), 122.9 (d, J = 2.3 Hz), 122.2, 122.1, 43.8 (d, J = 34.3 Hz), 31.7 (d, J = 8.5 Hz), 14.7 (d, J = 3.6 Hz); ^{31}P NMR (243 MHz, $CDCl_3$) δ 149.04. Data in accordance with the literature.[11]

(11bS)-*N,N*-Diisopropyldinaphtho[2,1-d:1',2'-f][1,3,2]dioxaphosphepin-4-amine (LS15)

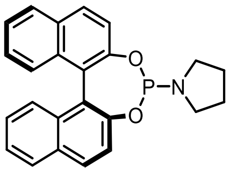

Following **GP4**, *(S)-(−)*-1,1′-Bi(2-naphthol) (3.0 g, 10.4 mmol) and pyrrolidine (1.9 mL, 23 mmol), gave **LS15** (0.98 g, 25%) as a solid. ^{1}H NMR (400 MHz, $CDCl_3$) δ 7.96 (1H, d, J = 8.8 Hz), 7.90 (3H, dd, J = 8.4, 6.2 Hz), 7.50 (1H, d, J = 8.8 Hz), 7.44–7.37 (4H, m), 7.34 (1H, d, J = 8.6 Hz), 7.31–7.27 (1H, m), 7.25–7.21 (1H, m), 3.23–3.10 (2H, m), 3.00–2.86 (2H, m), 1.81–1.62 (4H, m); ^{13}C NMR (101 MHz, $CDCl_3$) δ 150.4 (d, J = 4.9 Hz), 150.0, 133.0, 132.8, 131.5, 130.8, 130.3, 129.9, 128.5, 128.4, 127.13, 127.1, 126.2, 124.8, 124.7, 124.1 (d, J = 4.9 Hz), 123.1, 122.2, 122.1 (d, J = 1.9 Hz), 45.8 (d, J = 15.7 Hz), 26.0 (d, J = 4.2 Hz); ^{31}P NMR (162 MHz, $CDCl_3$) δ 150.19. Data in accordance with the literature.[12]

1-((11bS)-Dinaphtho[2,1-d:1',2'-f][1,3,2]dioxaphosphepin-4-yl)azepane (LS16)

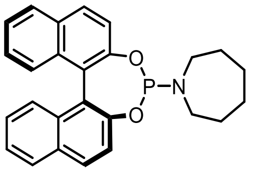

Following **GP4**, *(S)-(−)*-1,1′-Bi(2-naphthol) (3.0 g, 10.4 mmol) and azepane (2.6 mL, 23 mmol), gave **LS16** (0.84 g, 19%) as a solid. ^{1}H NMR (600 MHz, $CDCl_3$) δ 7.96 (1H, d, *J* = 8.8 Hz), 7.93–7.87 (3H, m), 7.52 (1H, d, *J* = 8.7 Hz), 7.46–7.37 (4H, m), 7.33 (1H, d, *J* = 8.6 Hz), 7.29–7.26 (1H, m), 7.26–7.19 (1H, m), 3.15–2.95 (4H, m), 1.61–1.55 (8H, m); ^{13}C NMR (151 MHz, $CDCl_3$) δ 150.4 (d, *J* = 5.4 Hz), 149.9, 133.0, 132.8, 131.5, 130.8, 130.3, 129.9, 128.45, 128.4, 127.2, 127.15, 126.1, 126.1, 124.8, 124.6, 124.2 (d, *J* = 5.0 Hz), 122.6, 122.4, 122.3, 47.1 (d, *J* = 21.1 Hz), 31.0 (d, *J* = 3.6 Hz), 27.2; ^{31}P NMR (243 MHz, $CDCl_3$) δ 150.17. Data in accordance with the literature.[11]

(11bS)-*N,N*-Dicyclohexyldinaphtho[2,1-d:1',2'-f][1,3,2]dioxaphosphepin-4-amine (LS18)

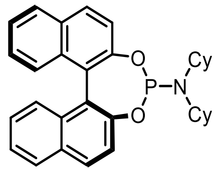

Following **GP4**, *(S)-(−)*-1,1′-Bi(2-naphthol) (2.0 g, 7.0 mmol) and Cy_2NH (3.1 mL, 15 mmol), gave **LS18** (1.1 g, 32%) as a solid. ^{1}H NMR (400 MHz, $CDCl_3$) δ 7.94 (1H, d, *J* = 8.8 Hz), 7.88 (3H, t, *J* = 9.0 Hz), 7.49 (1H, d, *J* = 8.8 Hz), 7.40 (4H, q, *J* = 7.4, 6.2 Hz), 7.30 (1H, d, *J* = 8.5 Hz), 7.25–7.14 (2H, m), 2.79 (2H, q, *J* = 11.7 Hz), 1.93–1.79 (2H, m), 1.76–1.56 (8H, m), 1.45 (4H, d, *J* = 11.2 Hz), 0.95 (6H, dt, *J* = 39.9, 18.5 Hz); ^{13}C NMR (101 MHz) δ 150.6 (d, *J* = 6.7 Hz), 150.3, 133.0, 132.8, 131.4, 130.6, 130.2, 129.4, 128.4, 128.2, 127.2, 127.16, 126.0, 125.9, 124.7, 124.4, 124.2 (d, *J* = 5.4 Hz), 122.6 (d, *J* = 2.1 Hz), 122.3, 122.0 (d, *J* = 2.2 Hz), 54.3 (d, *J* = 10.4 Hz), 51.0, 35.3, 26.5 (d, *J* = 12.3 Hz), 25.6; ^{31}P NMR (162 MHz, $CDCl_3$) δ 151.93. Data in accordance with the literature.[13]

(11bS)-*N,N*-Dibenzyldinaphtho[2,1-d:1',2'-f][1,3,2]dioxaphosphepin-4-amine (LS19)

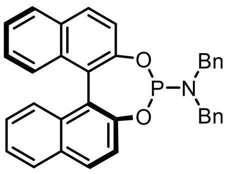

Following **GP4**, *(S)-(−)*-1,1′-Bi(2-naphthol) (2.0 g, 7.0 mmol) gave and Bn_2NH (3.0 mL, 15 mmol), **LS19** (0.72 g, 25%) as a solid. ^{1}H NMR (400 MHz, $CDCl_3$) δ 8.02 (1H, d, *J* = 8.8 Hz), 7.94 (1H, d, *J* = 8.2 Hz), 7.82 (1H, d, *J* = 8.6 Hz), 7.76 (1H, d, *J* = 8.8 Hz), 7.65 (1H, d, *J* = 8.7 Hz), 7.43 (1H, t, *J* = 7.5 Hz), 7.40–7.18 (15H, m), 7.12 (1H, d, *J* = 8.8 Hz), 4.21 (2H, dd, *J* = 15.0, 7.8 Hz), 3.44 (2H, t, *J* = 13.7 Hz); ^{13}C NMR (151 MHz, $CDCl_3$) δ 149.8 (d, *J* = 4.7 Hz), 149.4, 138.0, 133.0, 132.6, 131.6, 130.8,

130.4, 130.3, 129.0, 128.6, 128.5, 128.3, 127.5, 127.2, 127.0, 126.24, 126.22, 125.0, 124.7, 124.2, 122.8, 122.3, 121.6, 48.3 (d, *J* = 21.0 Hz); ^{31}P NMR (243 MHz, $CDCl_3$) δ 144.81. Data in accordance with the literature.[9]

4-((11bS)-Dinaphtho[2,1-d:1',2'-f][1,3,2]dioxaphosphepin-4-yl)morpholine (LS20)

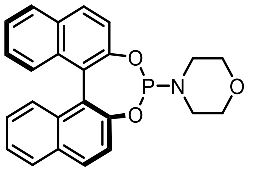

Following **GP4**, *(S)-(−)*-1,1′-Bi(2-naphthol) (3.0 g, 10.4 mmol) and morpholine (2.0 mL, 23 mmol), gave **LS20** (1.4 g, 34%) as a solid. ^{1}H NMR (600 MHz, $CDCl_3$) δ 7.98 (1H, d, *J* = 8.8 Hz), 7.95–7.90 (3H, m), 7.52 (1H, d, *J* = 8.8 Hz), 7.45–7.37 (4H, m), 7.33 (1H, d, *J* = 8.6 Hz), 7.31–7.27 (1H, m), 7.26–7.21 (1H, m), 3.59–3.47 (4H, m), 3.14–3.03 (2H, m), 3.03–2.90 (2H, m); ^{13}C NMR (151 MHz, $CDCl_3$) δ 149.6, 149.4, 132.9, 132.7, 131.6, 130.9, 130.5, 130.2, 128.49, 128.47, 127.1, 127.0, 126.3, 125.0, 124.9, 124.0, 122.9, 122.02, 121.98, 68.0 (d, *J* = 5.0 Hz), 44.7 (d, *J* = 17.9 Hz); ^{31}P NMR ($CDCl_3$, 243 MHz) δ 144.64. Data in accordance with the literature.[9]

4-((11bS)-Dinaphtho[2,1-d:1',2'-f][1,3,2]dioxaphosphepin-4-yl)thiomorpholine (LS21)

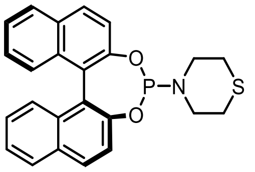

Following **GP4**, *(S)-(−)*-1,1′-Bi(2-naphthol) (2.0 g, 7.0 mmol) and thiomorpholine (1.5 mL, 15 mmol), gave **LS21** (0.72 g, 25%) as a solid. ^{1}H NMR (600 MHz, $CDCl_3$) δ 7.97 (1H, d, *J* = 8.8 Hz), 7.92 (3H, dd, *J* = 8.5, 6.8 Hz), 7.51 (1H, d, *J* = 8.7 Hz), 7.46–7.38 (4H, m), 7.32 (1H, d, *J* = 8.6 Hz), 7.30–7.26 (1H, m), 7.26–7.23 (1H, m), 3.38–3.20 (4H, m), 2.55–2.41 (4H, m); ^{13}C NMR (151 MHz, $CDCl_3$) δ 149.7, 149.3, 132.9, 132.7, 131.6, 130.9, 130.5, 130.2, 128.5, 127.1 (d, *J* = 5.4 Hz), 126.3, 125.0, 124.8, 124.0, 122.8, 122.0, 46.6 (d, *J* = 21.1 Hz), 28.3 (d, *J* = 4.8 Hz); ^{31}P NMR (243 MHz, $CDCl_3$) δ 145.07. Data in accordance with the literature.[9]

1-((11bS)-Dinaphtho[2,1-d:1',2'-f][1,3,2]dioxaphosphepin-4-yl)-2,2,6,6-tetramethylpiperidine (LS22)

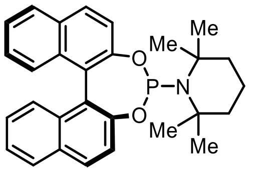

Following **GP4**, *(S)-(−)*-1,1′-Bi(2-naphthol) (3.0 g, 10.5 mmol) and 2,2,6,6-tetramethylpiperidine (2.0 mL, 23 mmol), gave **LS22** (0.97 g, 21%) as a solid. ^{1}H NMR (400 MHz, $CDCl_3$) δ 7.95 (1H, d, *J* = 8.8 Hz), 7.92–7.84 (3H, m), 7.45 (1H, d, *J* = 8.7 Hz), 7.41–7.33 (3H, m), 7.31–7.27 (1H, m), 7.26–7.16 (3H, m), 1.71–1.59 (2H, m), 1.56–1.42 (10H, m), 1.25 (6H, s); ^{13}C NMR (151 MHz, $CDCl_3$) δ 152.0 (d, *J* = 12.0 Hz), 150.9 (d, *J* = 2.1 Hz), 133.3 (d, *J* = 3.6 Hz), 131.4 (d, *J* = 1.5 Hz), 130.5 (d, *J* = 1.5 Hz), 130.1, 129.4 (d, *J* = 1.7 Hz), 128.4, 128.1, 127.5, 127.3, 126.1, 125.8, 124.9, 124.1, 123.7, 122.7 (d, *J* = 2.9 Hz), 121.0 (d, *J* = 2.7 Hz), 56.5, 56.4, 42.5, 32.9 (d, *J* = 9.5 Hz), 17.3; ^{31}P NMR ($CDCl_3$, 162 MHz) δ 162.88. Data in accordance with the literature.[14]

1-((11bS)-Dinaphtho[2,1-d:1',2'-f][1,3,2]dioxaphosphepin-4-yl)-4-methylpiperazine (LS23)

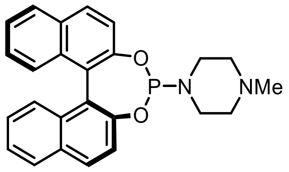

Following **GP4**, *(S)-(−)*-1,1′-Bi(2-naphthol) (3.0 g, 10.4 mmol) and 1-methylpiperazine (2.5 mL, 23 mmol), gave **LS23** (0.99 g, 23%) as a solid. ^{1}H NMR (600 MHz, $CDCl_3$) δ 7.97 (1H, d, *J* = 8.8 Hz), 7.91 (3H, dd, *J* = 8.5, 4.4 Hz), 7.52 (1H, d, *J* = 8.7 Hz), 7.45–7.37 (4H, m), 7.33 (1H, d, *J* = 8.6 Hz), 7.30–7.27 (1H, m), 7.26–7.22 (1H, m), 3.10 (2H, br s), 2.99 (2H, br s), 2.24 (7H, br s); ^{13}C NMR (151 MHz, $CDCl_3$) δ 149.9 (d, *J* = 4.8 Hz), 149.5, 132.9, 132.7, 131.5, 130.9, 130.4, 130.1, 128.5, 127.1, 127.0, 126.24, 126.22, 124.9, 124.8, 124.0 (d, *J* = 5.0 Hz), 122.9 (d, *J* = 2.4 Hz), 122.2, 122.1, 56.1 (d, *J* = 5.4 Hz), 46.6, 44.3 (d, *J* = 18.9 Hz); ^{31}P NMR (243 MHz, $CDCl_3$) δ 145.06. Data in accordance with the literature.[9]

(11bS)-*N*-Ethyl-*N*-phenyldinaphtho[2,1-d:1',2'-f][1,3,2]dioxaphosphepin-4-amine (LS24)

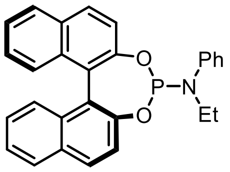

Following **GP4**, *(S)-(−)*-1,1′-Bi(2-naphthol) (2.0 g, 7.0 mmol) and *N*-ethylaniline (1.9 mL, 23 mmol), gave **LS24** (0.9 g, 30%) as a solid. ^{1}H NMR (400 MHz, $CDCl_3$) δ 7.99 (1H, d, *J* = 8.8 Hz), 7.92 (3H, dd, *J* = 8.4, 3.3 Hz), 7.56 (1H, dd, *J* = 8.8, 1.0 Hz), 7.50–7.38 (4H, m), 7.31 (7H, ddt, *J* = 15.1, 9.5, 3.9 Hz), 7.15 (1H, ddt, *J* = 8.5, 5.0, 1.5 Hz), 3.34–3.18 (1H, m), 3.16–2.98 (1H, m), 0.82 (3H, t, *J* = 7.0

Hz); ^{13}C NMR (101 MHz, $CDCl_3$) δ 150.0 (d, J = 5.3 Hz), 149.5, 143.4, 143.1, 133.0, 132.8, 131.6, 130.9, 130.5, 130.1, 129.3, 128.5, 128.45, 127.2, 127.1, 126.3, 126.2, 125.5, 125.4, 125.0, 124.8, 124.6 (d, J = 2.1 Hz), 124.2 (d, J = 5.2 Hz), 122.7, 122.2, 122.1, 41.0, 14.7; ^{31}P NMR (162 MHz, $CDCl_3$) δ 143.25. Data in accordance with the literature.[15]

General procedure for the preparation of Ox-L2

The oxide was prepared following procedure from the literature.[16]

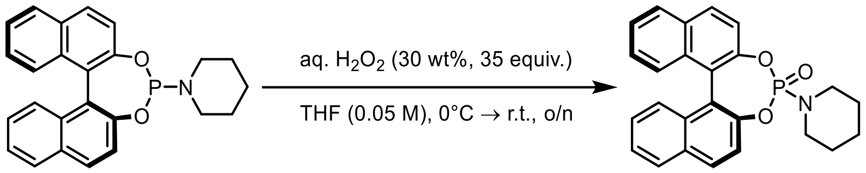

A round bottom flask was charged with **L2** (250 mg, 0.63 mmol, 1.0 equiv.) and dry THF (0.05 M, 12.0 mL). The resulting mixture was cooled down to 0 °C. Then H_2O_2 (30 wt% in H_2O, 2.2 ml, 22 mmol, 35 equiv.) was added dropwise. The reaction mixture was stirred 16 h at room temperature. The solvent was then evaporated, and the residue was purified by flash chromatography to give **Ox-L2** (226 mg, 78%) as a solid. R_f 0.50 [pentane:EtOAc1:1 +1% Et_3N]; $[\alpha]_D$ = + 438.3 (c = 0.6 in $CHCl_3$); ^1H NMR (600 MHz, $CDCl_3$) δ 8.01 (2H, dd, *J* = 14.2, 8.4 Hz), 7.94 (2H, t, *J* = 8.8 Hz), 7.60 (1H, d, *J* = 8.9 Hz), 7.53–7.41 (4H, m), 7.34–7.26 (4H, m), 3.09–1.44 (6H, m); ^{13}C NMR (151 MHz, $CDCl_3$) δ 148.4 (d, *J* = 10.8 Hz), 147.1, 147.0, 132.5, 132.4, 131.9, 131.4, 131.3, 130.8, 128.6, 128.5, 127.4, 127.1, 126.8, 126.6, 125.7, 125.6, 121.41 (d, *J* = 2.3 Hz), 121.37 (d, *J* = 2.6 Hz), 121.1 (d, *J* = 3.2 Hz), 46.5 (d, *J* = 2.2 Hz), 26.2 (d, *J* = 4.2 Hz), 24.3; ^{31}P NMR (243 MHz, $CDCl_3$) δ 12.22; HRMS (ESI): found MH^+ 416.1405, $C_{25}H_{23}NO_3P$ requires 416.1410.

4 Overview of Azepine Starting Materials

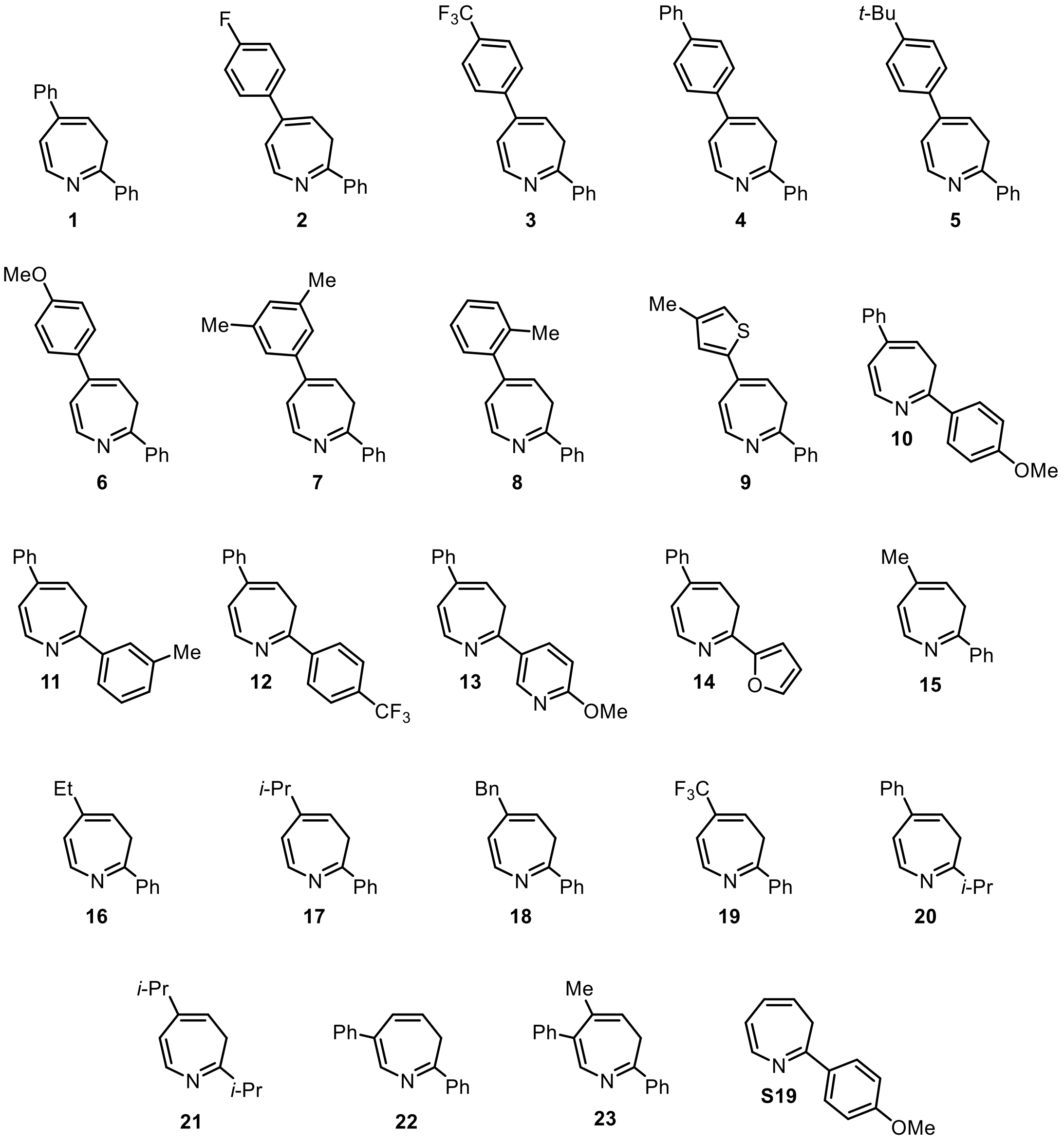

Figure S1.

5 Pictures of Reactions Set-up

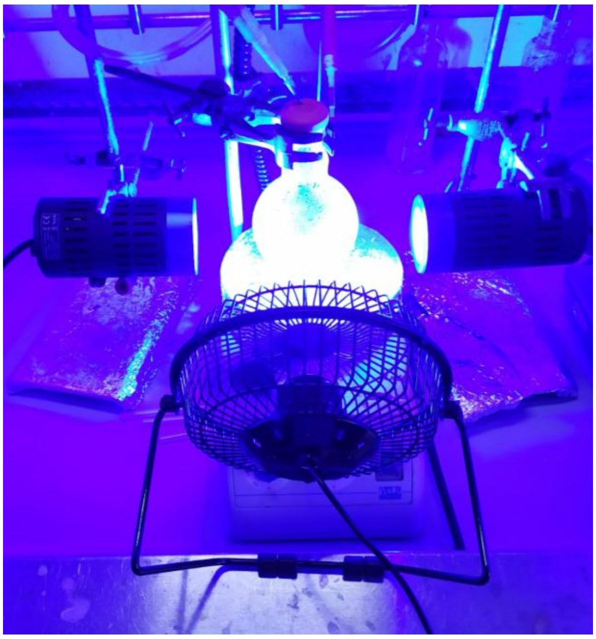

Figure S2. Set-up for gram-scale ring expansion of nitroarenes.

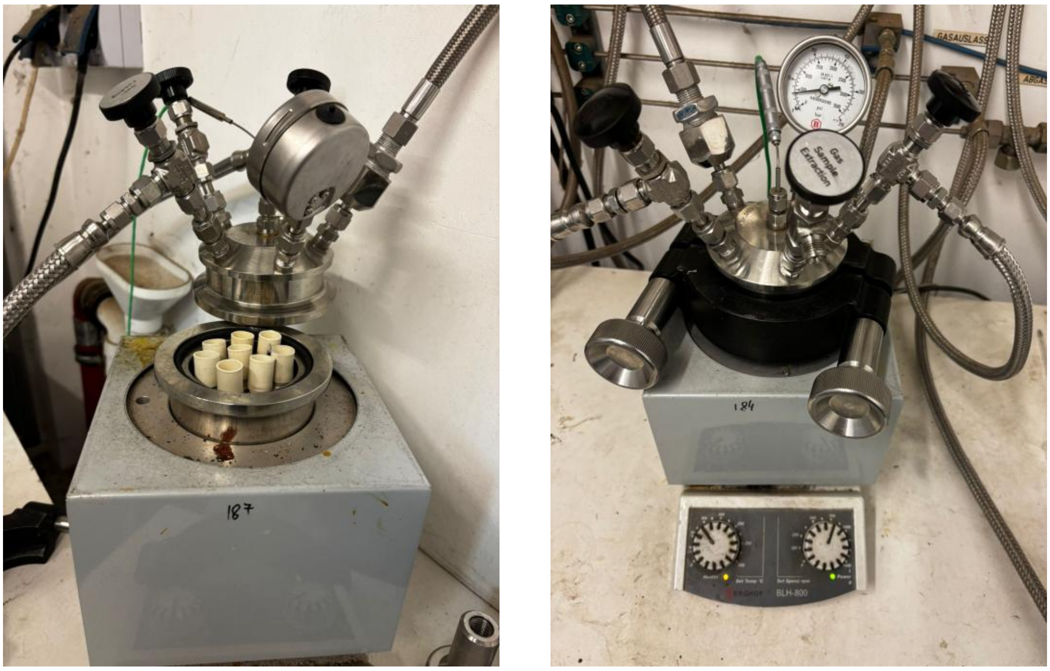

Figure S3. Set-up for 0.1-0.2 mmol scale hydrogenation of azepine.

6 Reaction Optimization

General procedure for the preparation of [M]-bisphosphine pre-catalyst stock solution

In an argon filled glove box, a vial was charged with metal catalyst (1.0 equiv., $[Ir(COD)Cl]_2$ or $[Rh(COD)Cl]_2$), chiral bisphosphine ligand (1.1 equiv.) and degassed THF (0.005 M). The pre-catalyst stock solution was stirred for 10 min at r.t..

6.1 General Procedure for the Reaction Optimization of the Asymmetric Reduction of Azepines Using Bisphosphine Ligands – GP5

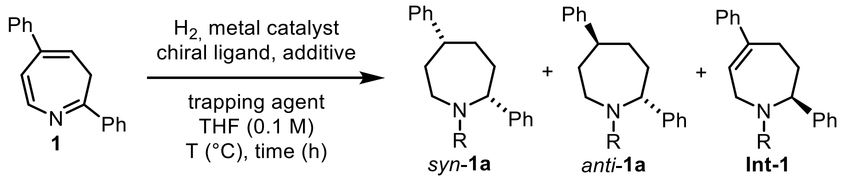

In an Ar filled glovebox, a microwave vial was charged with **1** (0.1 mmol, 1.0 equiv.) and the corresponding additive (0.3-1.0 equiv.). Then 0.5 mL of a [M]-bisphosphine pre-catalyst stock solution (2.5 mol% [M] = $[Ir(COD)Cl]_2$ or $[Rh(COD)Cl]_2$) and 0.5 mL of solvent were added to the solid (0.1 M). The trapping agent (1.1 equiv.) was added, the vial was sealed with a septum and parafilm and then taken out of the glovebox. The reaction mixture was then placed in a stainless steel autoclave. The septum was removed under a flow of nitrogen. The autoclave was flushed with N_2 (3 x 10 bars) followed with H_2 (3 x 10 bars) and then pressurized to the correct pressure of H_2. The reaction mixture was stirred at the corresponding temperature. After the specified time, the autoclave was slowly depressurized, flushed with N_2 (3 x 10 bars), and the reaction vial was cooled down to room temperature. A solution of 1,3-dinitrobenzene in $CDCl_3$ (1.0 equiv., 0.2 M) was added as an internal standard and the reaction analysed by ^{1}H NMR. The diastereomeric ratio was determined by ^{1}H NMR analysis of the reaction crude. The enantiomeric ratio was determined by chiral HPLC (Chiralcel® OD-H, hexane:*i*-PrOH 95:5, flow 0.75 mL/min, oven temperature 30 °C, *syn*-**1a** t_r = 7.15 min and 7.78 min, *anti*-**1a** t_r = 9.03 min and 12.68 min, **Int-1** t_r = 11.14 min and 15.67 min). For all the following table, the er for *syn*-**1a** corresponds to the ratio of (*RR*):(*SS*), for *anti*-**1a** to (*RS*):(*SR*), and for **Int-1** to (*S*):(*R*). The dr is reported as the ratio of *anti*:*syn*.

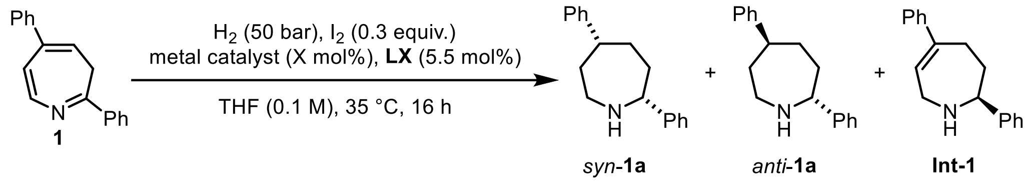

Table S1. Screening of Bidentate Ligands.

entry	metal catalyst (mol%)	L	*syn*-1a		*anti*-1a		dr	Int-1		1 (rsm) (%)
			yield (%)	er	yield (%)	er		yield (%)	er	
**1**	**[Ir]** (2.5)	**L1**	–	–	–	–	–	37	64:36	26
**2**	**[Ir]** (2.5)	**LS2**	21	58:42	–	–	>1:20	38	58:42	24
**2**	**[Ir]** (2.5)	**LS8**	–	–	–	–	–	55	75:25	25
**4**	**[Ir]** (2.5)	**LS4**	11	50:50	–	–	–	40	50:50	31
**5**	**[Rh]** (2.5)	**L1**	32	85:15	4	72:28	1:9	14	57:43	38
**6**	**[Rh]** (2.5)	**LS2**	–	–	–	–	–	–	–	decomp.
**7**	**[Rh]** (2.5)	**LS8**	13	58:42	–	–	–	29	85:15	29
**8**	**[Rh]** (2.5)	**LS4**	52	59:41	9	63:37	1:5.8	–	n.d.	28
**9**	**[Rh]** (2.5)	**LS5**	9	74:26	–	–	1:11	36	–	31
**10**	**[Rh]** (2.5)	**LS6**	28	81:19	–	–	>1:20	30	62:38	51
**11**	**[Rh]** (2.5)	**LS12**	<5	–	–	–	–	15	50:50	26
**12**	**[Rh]** (2.5)	**LS11**	21	81:19	–	–	1:8	15	73:27	30

entry	metal catalyst (mol%)	L	*syn*-1a		*anti*-1a		dr	Int-1		1 (rsm) (%)
			yield (%)	er	yield (%)	er		yield (%)	er	
**13**	**[Rh]** (2.5)	**LS10**	26	81:19	–	–	1:10.6	10	70:30	26
**14**	**[Rh]** (2.5)	**LS9**	44	80:20	<5	–	1:18	0	–	25
**15**	**[Rh]** (2.5)	**LS7**	8	75:25	–	–	–	40	75:25	–
**16**	**[Rh]-S1** (5.0)	**L1**	6	82:18	–	–	–	43	76:24	33
**17**	**[Rh]-S2** (5.0)	**L1**	6	84:16	–	–	–	38	77:23	45

[Rh] = $[Rh(COD)Cl]_2$; **[Ir]** = $[Ir(COD)Cl]_2$, **[Rh]-S1** $Rh(COD)_2BF_4$; **[Rh]-S2** $Rh(COD)_2$BArF

entry	metal catalyst (mol%)	L	*syn*-1a		*anti*-1a		dr	Int-1		1 (rsm) (%)
			yield (%)	er	yield (%)	er		yield (%)	er	

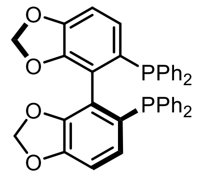

L1

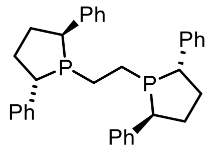

LS2

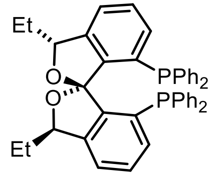

LS3

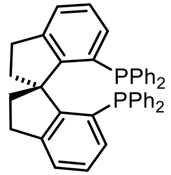

LS4

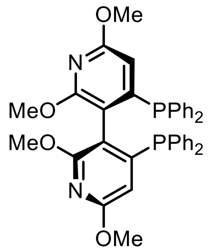

LS5

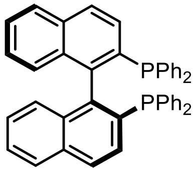

LS6

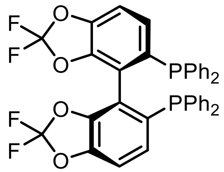

LS7

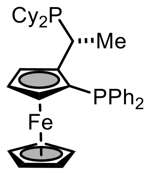

LS8

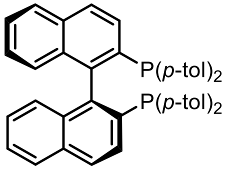

LS9

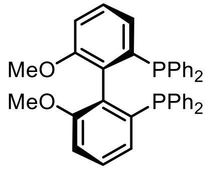

LS10

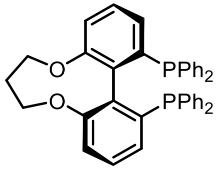

LS11

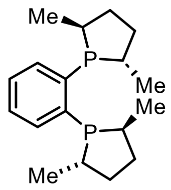

LS12

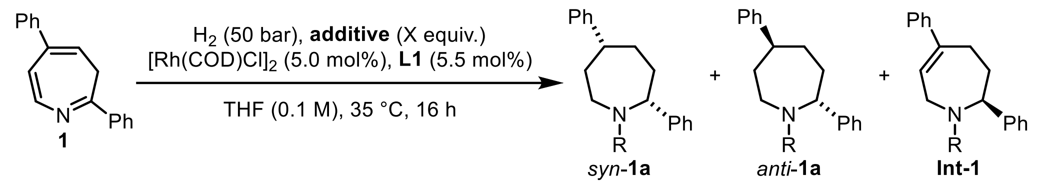

Table S2. Screening of Additive.

entry	additives (equiv.)	*syn*-1a		*anti*-1a		dr	Int-1		1 (rsm) (%)
		yield (%)	er	yield (%)	er		yield (%)	er	
**1**	–	–	–	–	–	–	–	–	87
**2**	I_2 (0.3)	32	85:15	4	72:28	1:9	14	57:43	33
**3**	IBr (0.3)	37	79:21	5	n.d.	1:7.4	6	n.d.	17
**4**	NBS (0.3)	25	75:25	–	–	–	–	–	67
**5**	NIS (0.3)	23	62:38	–	–	–	–	–	–
**6**	NBS (1.0)	42	85:15	10	61:39	1:4	25	75:25	–
**7**	Boc_2O (1.1), NBS (1.0)	–	–	–	–	–	43	50:50	40
**8**	Boc_2O (1.1), I_2 (0.3)	35	75:25	10	65:35	1:3:5	–	–	–

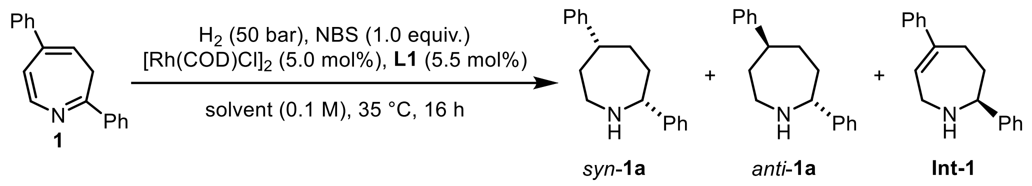

Table S3. Screening of Solvent.

entry	solvent	***syn*-1a**		***anti*-1a**		dr	**Int-1**		1 (rsm) (%)
		**yield (%)**	**er**	**yield (%)**	**er**		**yield (%)**	**er**	
**1**	THF	42	85:15	10	61:39	1:4	25	75:25	–
**2**	1,4-dioxane	36	73:27	11	n.d.	1:3.3	17	–	–
**3**	toluene	7	–	–	–	–	67	54:46	–
**4**	DCE	24	83:17	10	65:35	1:2.4	37	56:44	–
**5**	DCM	37	86:14	15	74:26	1:2.5	33	50:50	–
**6**	HFIP	–	–	–	–	–	45	50:50	12

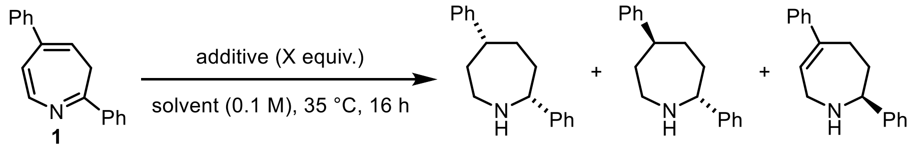

1   *syn*-**1a**   *anti*-**1a**   **Int-1**

Table S4. Decomposition of **1** with Additives.

entry	additive (equiv.)	*syn*-1a		*anti*-1a		dr	Int-1		1 (rsm) (%)
		yield (%)	er	yield (%)	er		yield (%)	er	
**1**	–	–	–	–	–	–	–	–	95
**2**	I_2 (0.3)	–	–	–	–	–	–	–	44
**3**	NBS (1.0)	–	–	–	–	–	–	–	traces

General procedure for the preparation of [Ir]-phosphoramidite pre-catalyst stock solution

In an argon filled glove box, a vial was charged with $[Ir(COD)Cl]_2$ (1.0 equiv.), (*S*)-phosphoramidite (4.0 equiv.) and degassed THF (0.01 M). The pre-catalyst stock solution was stirred for 30 min at r.t..

General procedure for the preparation of additive stock solution

In an argon filled glove box, a vial was charged with additive PR_3 (1.0 equiv.) and degassed THF (0.02M). The mixture was stirred for 5 min at r.t..

6.2 General Procedure for the Reaction Optimization of the Asymmetric Reduction of Azepines Using Monodentate Ligands – GP6

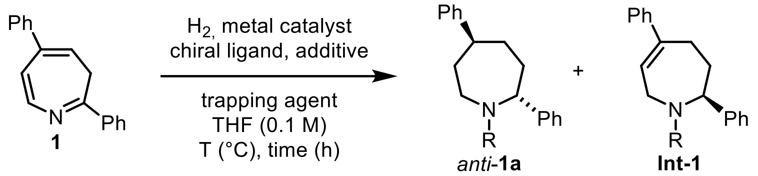

In an Ar filled glovebox, a microwave vial was charged with **1** (1.0 equiv.). Then 0.5 mL of a [Ir]-phosphoramidite pre-catalyst stock solution (5.0 mol%, [Ir] = $[Ir(COD)Cl]_2$) and 0.5 mL of an additive stock solution (0.1 equiv.) were added to the solid (0.1 M). The trapping agent (1.1 equiv.) was added, the vial was sealed with a septum and parafilm and then taken out of the glovebox. The reaction mixture was then placed in a stainless steel autoclave. The septum was removed under a flow of nitrogen. The autoclave was flushed with N_2 (3 x 10 bars) followed with H_2 (3 x 10 bars) and then pressurized to the correct pressure of H_2. The reaction mixture was stirred at the corresponding temperature. After the specified time, the autoclave was slowly depressurized, flushed with N_2 (3 x 10 bars), and the reaction vial was cooled down to room temperature. A solution of 1,3-dinitrobenzene in $CDCl_3$ (1.0 equiv., 0.2 M) was added as an internal standard and the reaction analysed by ^{1}H NMR. The diastereomeric ratio was determined by ^{1}H NMR analysis of the reaction crude. The enantiomeric ratio was determined by chiral HPLC (for *anti*-**1a**: Chiralpak® AD-H, hexane:*i*-PrOH 99:1, flow 1.0 mL/min, oven temperature 30 °C; for **Int-1**: Chiralcel® IA, hexane:*i*-PrOH 98:2, flow 0.75 mL/min, oven temperature 30 °C.). For all the following table, the er for *syn*-**1a** corresponds to the ratio of (*RR*):(*SS*), for *anti*-**1a** to (*RS*):(*SR*), and for **Int-1** to (*S*):(*R*). The dr is reported as the ratio of *anti*:*syn*.

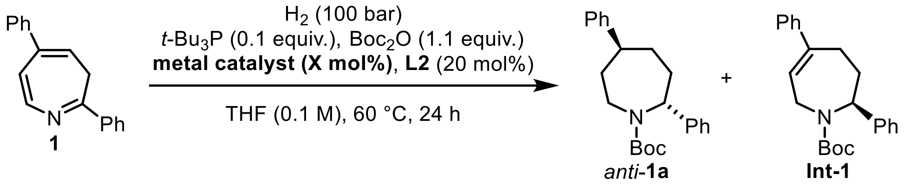

Table S5. Screening of Metal Catalyst.

entry	metal catalyst (mol%)	***anti*-1a**			**Int-1**		**1 (rsm) (%)**
		**yield (%)**	**er**	**dr**	**yield (%)**	**er**	
**1**	$[Ir(COD)Cl]_2$ (2.5)	–	–	–	80	50:50	–
**2**	$[Ir(COD)Cl]_2$ (5.0)	39	95:5	>20:1	47	80:20	–
**2**	$Ir(COD)_2BF_4$ (10)	63	81:19	–	18	n.d.	–
**4**	$Ir(COD)_2BARF$ (10)	36	75:25	7:1	48	76:24	–
**5**	$[Rh(COD)Cl]_2$ (5.0)	–	–	–	–	–	100

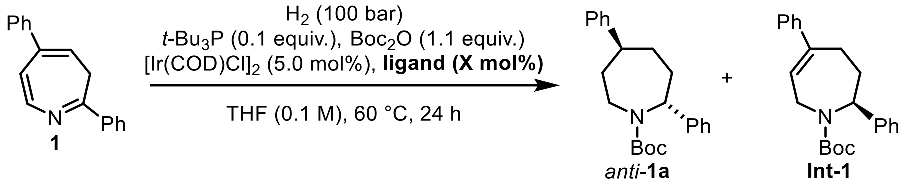

Table S6. Screening of Chiral Phosphoramidite.

entry	ligand (20 mol%)	*anti*-1a			Int-1		1 (rsm) (%)
		yield (%)	er	dr	yield (%)	er	
**1**	**L2**	39	95:5	>20:1	47	80:20	–
**2**	**L3**	37	93:7	>20:1	48	73:27	–
**3**	**LS13**	24	92:8	>20:1	71	61:39	–
**4**	**L4**	20	71:29	>20:1	63	66:34	–
**5**	**LS14**	27	97:3	>20:1	60	66:34	–
**6**	**LS15**	10	99:1	>20:1	70	55:45	–
**7**	**LS16**	46	90:10	>20:1	42	87:13	–
**8**	**LS17**	53	79:21	>20:1	36	84:16	–
**9**	**LS18**	25	68:32	5:1	60	73:27	–
**10**	**LS19**	30	80:20	>20:1	55	73:27	–
**11**	**LS20**	40	94:6	>20:1	42	74:26	–
**12**	**LS21**	–	–	–	30	59:41	55

entry	ligand (20 mol%)	***anti*-1a**			**Int-1**		**1 (rsm) (%)**
		**yield (%)**	**er**	**dr**	**yield (%)**	**er**	
**14**	**LS22**	–	–	–	94	36:64	–
**15**	**LS23**	<5	93:7	>20:1	87	65:35	–
**16**	**LS24**	32	91:9	>20:1	49	80:20	–
**17**	**L5**	–	–	–	93	50:50	–
**18**	**LS25**	–	–	–	83	36:64	–
**19**	**LS26**	10	93:7	>20:1	70	58:42	–

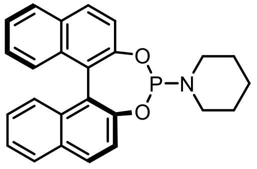

L2

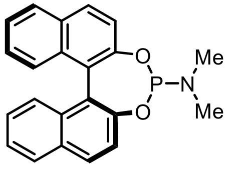

L3

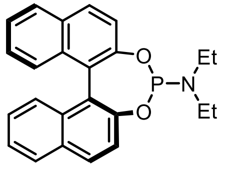

LS13

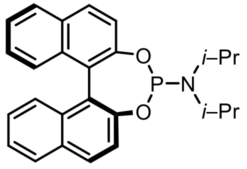

L4

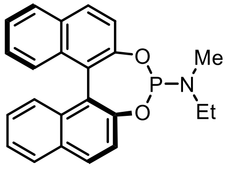

LS14

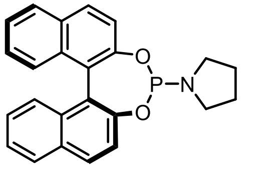

LS15

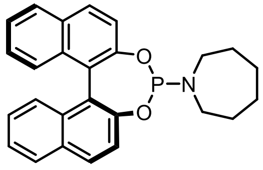

LS16

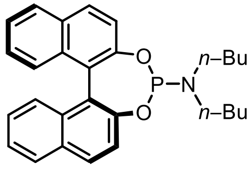

LS17

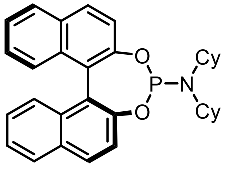

LS18

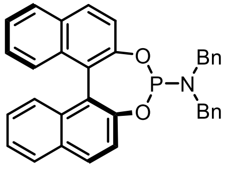

LS19

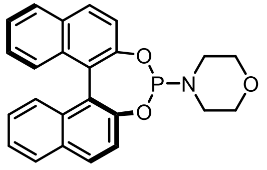

LS20

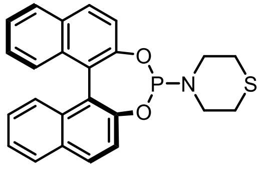

LS21

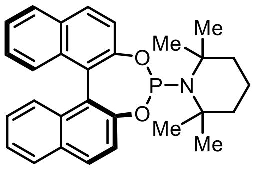

LS22

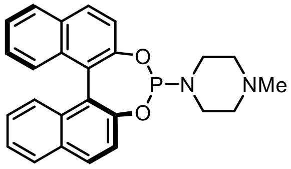

LS23

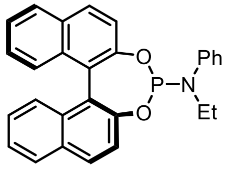

LS24

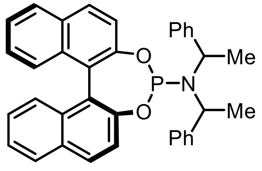

LS25

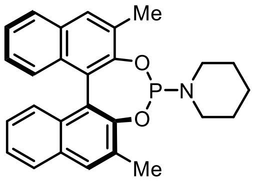

L5

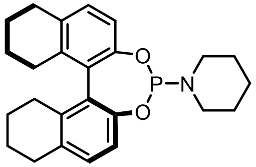

LS26

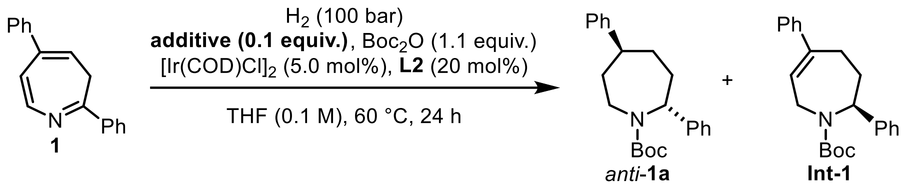

Table S7. Screening of Additives.

entry	additives (0.1 equiv.)	***anti*-1a**			**Int-1**		**1 (rsm) (%)**
		**yield (%)**	**er**	**dr**	**yield (%)**	**er**	
**1**	*t*-Bu_3P	39	95:5	>20:1	47	80:20	–
**2**	Me_3P	16	92:8	>20:1	67	53:47	4
**3**	Et_3P	30	93:7	>20:1	64	45:55	–
**4**	*i*-Pr_3P	15	67:33	>20:1	72	43:57	–
**5**	*n*-Bu_3P	26	67:33	>20:1	72	57:43	–
**6**	Bn_3P	–	–	–	35	44:56	43
**7**	Cy_3P	–	–	–	10	28:72	70
**8**	*1*-Ad_3P	56	92:8	>20:1	33	97:3	–
**9**	Ph_3P	–	–	–	51	39:61	24
**10**	(*o*-tolyl)$_3$P	34	85:15	>20:1	49	75:25	–
**11**	(*m*-tolyl)$_3$P	–	–	–	81	50:50	–

entry	additives (0.1 equiv.)	***anti*-1a**			**Int-1**		**1 (rsm) (%)**
		**yield (%)**	**er**	**dr**	**yield (%)**	**er**	
**12**	(*p*-tolyl)$_3$P	12	83:17	>20:1	71	50:50	–
**13**	(*p*-F-Ph)$_3$P	–	–	–	20	44:56	45
**14**	(*p*-anisole)$_3$P	<5	–	–	67	48:52	–
**15**	(*o*-anisole)$_3$P	15	92:8	>20:1	72	52:48	–
**16**	$(C_6F_5)_3P$	30	91:9	>20:1	52	69:31	–
**17**	Mes_3P	11	50:50	2:1	44	85:15	–
**18**	(*2*-furan)$_3$P	–	–	–	74	46:54	–
**19**	(*1*-naphthalene)$_3$P	–	–	–	–	–	100
**20**	*rac*-binap	56	73:27	>20:1	21	98:2	–
**21**	JohnPhos	48	64:36	2.4:1	14	86:14	–
**22**	PPh_2Me	–	–	–	82	65:35	–
**23**	$PPh_2(CH_2)PPh_2$	50	82:18	>20:1	34	79:21	–
**24**	$(OMe)_3P$	10	97:3	>20:1	84	50:50	–
**26**	$(OEt)_3P$	17	93:7	>20:1	57	63:37	–
**27**	(O*i*-Pr)$_3$P	–	–	–	78	42:58	8
**28**	$(OPh)_3P$	–	–	–	29	50:50	51
**29**	$(NMe_2)_3P$	10	82:18	>20:1	78	54:46	–

entry	additives (0.1 equiv.)	*anti*-1a			Int-1		1 (rsm) (%)
		yield (%)	er	dr	yield (%)	er	
**30**	$(NEt_2)_3P$	<5	96:4	>20:1	84	53:47	–
**31**	Cl_3P	–	–	–	–	–	decomp.

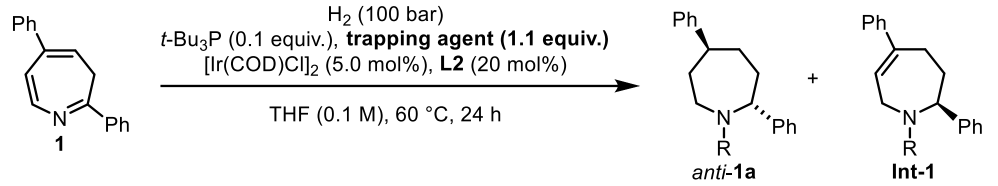

Table S8. Screening of Trapping Agent.

entry	trapping agent	*anti*-1a			Int-1		1 (rsm) (%)
		yield (%)	er	dr	yield (%)	er	
**1**	Boc_2O	39	95:5	>20:1	47	80:20	-
**2**	MeI	–	–	–	–	–	decomp.
**3**	MeOTf	–	–	–	24	n.d.	decomp.
**4**	BnBr	–	–	–	–	–	decomp.
**5**	TFAA	–	–	–	18	n.d.	decomp.
**6**	Ac_2O	41	63:37	8:1	30	n.d.	–

entry	trapping agent	*anti*-1a			Int-1		1 (rsm) (%)
		yield (%)	er	dr	yield (%)	er	
**7**	BzCl	–	–	–	–	–	decomp.
**8**	Bz_2O	36	88:12	10.3:1	36	n.d.	–
**9**[a]	Bz_2O	23	97:3	11.5:1	56	n.d.	–
**10**	Ts_2O	–	–	–	24	n.d.	–
**11**	FmocOsu	67	84:16	13:1	13	n.d.	–
**12**	–	–	–	–	40	50:50	51

[a] 16 h

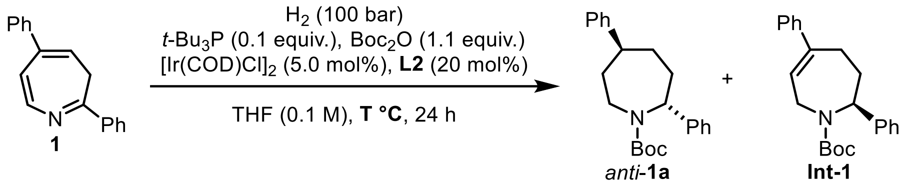

Table S9. Screening of Temperature.

entry	temperature (°C)	*anti-1a*			Int-1		1 (rsm) (%)
		yield (%)	er	dr	yield (%)	er	
**1**	35	–	–	–	72	50:50	20
**2**	60	39	95:5	>20:1	47	80:20	–

entry	temperature (°C)	*anti-1a*			Int-1		1 (rsm) (%)
		yield (%)	er	dr	yield (%)	er	
**3**	80	72	64:36	4.5:1	–	–	–

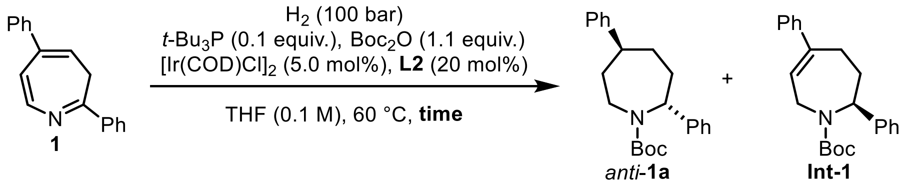

Table S10. Screening of Reaction Time.

entry	time (h)	*anti*-1a			Int-1		1 (rsm) (%)
		yield (%)	er	dr	yield (%)	er	
**1**	16	30	95:5	>20:1	52	70:30	–
**2**	24	39	95:5	>20:1	47	80:20	–
**3**	48	68	81:19	8.5:1	19	99:1	–

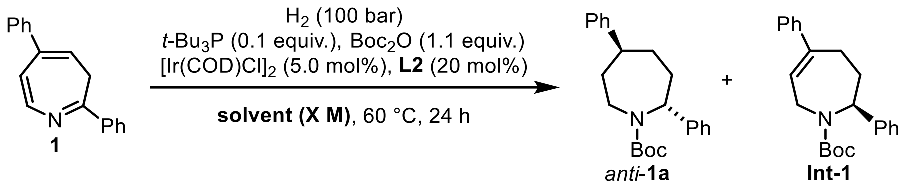

Table S11. Screening of Solvent.

entry	solvent (0.1 M)	*anti*-1a			Int-1		1 (rsm) (%)
		yield (%)	*er*	dr	yield (%)	*er*	
**1**	THF	39	95:5	>20:1	47	80:20	–
**2**	THF (0.2 M)	60	80:20	8:1	26	98:2	–
**3**	THF (0.05 M)	40	90:10	10:1	42	80:20	–
**4**	1,4-dioxane	46	92:8	>20:1	52	69:31	–
**5**	DCE	20	94:6	>20:1	76	50:50	–
**6**	DCM	20	93:7	>20:1	70	65:35	–
**7**	EtOAc	40	94:6	>20:1	57	62:38	–
**8**	*i*-PrOH	30	87:13	15:1	70	65:35	–
**9**	toluene	59	92:8	>20:1	31	98:2	–
**10**	MTBE	58	91:9	>20:1	32	95:5	–

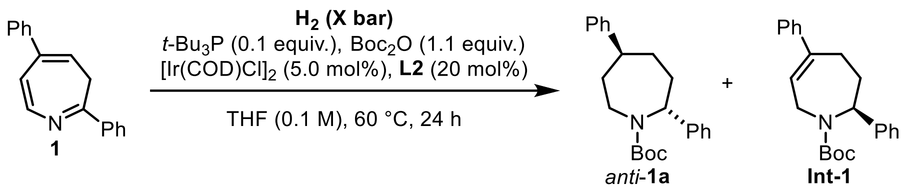

Table S12. Screening of H_2 Pressure.

entry	H_2 pressure (bar)	*anti*-1a			Int-1		1 (rsm) (%)
		yield (%)	er	dr	yield (%)	er	
**1**	120	38	96:4	>20:1	52	62:38	–
**2**	100	39	95:5	>20:1	47	80:20	–
**3**	50	41	91:9	>20:1	41	79:21	–
**4**	30	15	93:7	>20:1	58	53:47	18

7 Preparation of Azepanes

General Procedure for the Asymmetric Reduction of Azepines – GP7

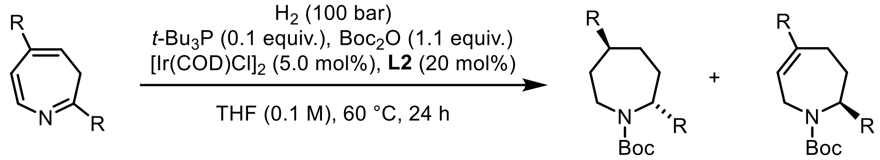

In an Ar filled glove box, a microwave vial was charged with the azepine (0.1 mmol, 1.0 equiv.). Then 0.5 mL of a [Ir]-**L2** pre-catalyst stock solution (5.0 mol%, [Ir] = $[Ir(COD)Cl]_2$) and 0.5 mL of *t*-Bu_3P stock solution (0.1 equiv.) were added to the solid (0.1 M). Boc_2O (1.1 equiv.) was added, the vial was sealed with a septum and parafilm and then taken out of the glovebox. The reaction mixture was then placed in a stainless steel autoclave and the septum was removed under a flow of N_2. The autoclave was flushed with N_2 (3 x 10 bars) followed with H_2 (3 x 10 bars) and then pressurized to 100 bars of H_2. The reaction mixture was stirred for 24 h at 60 °C. After the specified time, the autoclave was slowly depressurized, flushed with N_2 (3 x 10 bars), and the reaction vial was cooled down to room temperature. The crude mixture was then purified with CombiFlash® Rf+ to give the desired product with a dr >20:1 unless otherwise stated. The enantiomeric excess was determined by chiral HPLC after purification. The racemic samples were prepared using the same procedure but using *rac*–**L2**.

tert-Butyl (2*R*,5*S*)-2,5-Diphenylazepane-1-carboxylate (1a)

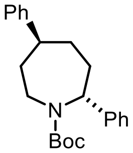

Following **GP7**, **1** (25 mg, 0.1 mmol), gave **1a** (39%, 95:5 er) as a solid. R_f 0.75 [hexane:EtOAc (9:1)]; HPLC analysis of **1a**: Chiralpak® AD-H, hexane:*i*-PrOH 99:1, flow 1.0 mL/min, oven temperature 30 °C, t_r = 10.64 min (major) and t_r = 14.01 min (minor); $[\alpha]_D$ = +14.2 (c = 0.15, $CHCl_3$, determined on (2*R*,5*S*)-**1a** after purification by chiral HPLC >99:1 er); ^1H NMR (600 MHz, $CDCl_3$, rotamers) δ 7.35–7.26 (4H, m), 7.26–7.17 (6H, m), 5.31 (0.5H, dd, *J* = 11.6, 6.5 Hz), 5.01 (0.5H, dd, *J* = 12.4, 5.9 Hz), 4.27 (0.5H, d, *J* = 14.6 Hz), 3.95 (0.5H, d, *J* = 13.3 Hz), 3.17 (0.5H, dd, *J* = 14.5, 11.6 Hz), 3.09 (0.5H, dd, *J* = 14.3, 12.5 Hz), 2.69–2.58 (1H, m), 2.53–2.44 (0.5H, m), 2.38–2.30 (0.5H, m), 2.09–1.92 (2H, m), 1.92–1.75 (3H, m), 1.49 (4.5H, s), 1.33 (4.5H, s); ^{13}C NMR (151 MHz, $CDCl_3$, rotamers) δ 156.2, 156.1, 147.5, 147.2, 144.9, 143.6, 128.66, 128.64, 128.6, 128.5, 127.0, 126.9, 126.8, 126.7, 126.3, 126.27, 125.8, 125.6, 79.8, 79.7, 60.9, 58.7, 48.55, 48.5, 42.5, 42.2, 38.6, 38.1, 35.9, 34.8, 34.5, 33.0, 28.7, 28.5; HRMS (ESI): found MNa^+ 374.2079, $C_{23}H_{29}NO_2Na$ requires 374.2091.

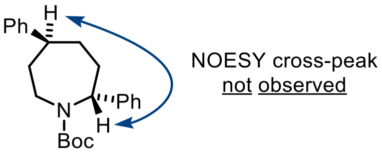

The main enantiomer of **1a** was isolated by preparative chiral HPLC (Chiralpak® IA, 250 x 20 mm, hexane:*i*-PrOH 99.2:0.8, 12 ml /min 15 bar, UV detector 230 nm.) for crystallization. Suitable single crystal of enantiopure **1a** for XRD analysis (see **Figure S4**) was obtained by slow evaporation of aqueous HCl (30 wt%)/1,4-dioxane at room temperature:

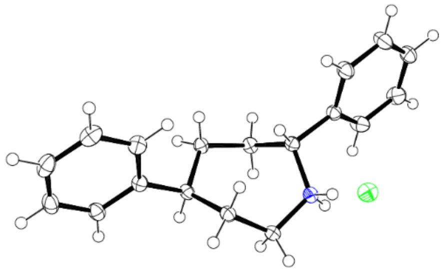

Figure S4. ORTEP plot of (2*R*,5*S*)-**1a** with thermal ellipsoids set at the 50% probability level.[17]

Crystallization of compound (2*R*,5*S*)-**1a·HCl** ($C_{18}H_{22}NCl$) from aqueous HCl (30 wt%)/1,4-dioxane at room temperature resulted in crystals of good quality for X-ray analysis. Crystallographic data were collected on a Bruker D8 Adventure diffractometer[18] with monochromatic Mo–Kα radiation (λ = 0.71073 Å) and a PHOTONIII detector. The structure was solved by intrinsic phasing using the ShelXT 2018/2 structure solution program[19] and refined against F^2 on all data by full-matrix least-squares methods using ShelXL-2018/3[20] and Olex2[21]. All non-hydrogen atoms were refined anisotropically. The carbon bound hydrogen atoms were placed at idealised positions and refined isotropically using the riding model. The NH hydrogens were allowed to freely refine isotropically. Flack x parameter was determined using 1577 quotients [(I+)-(I-)]/[(I+)+(I-)].[22] The Hooft Parameter was determined as 0.022(9).[23]

CCDC	2540110	
Empirical formula	$C_{23}H_{27}NO_2$	
Formula weight	349.45	
Temperature	100.0 K	
Wavelength	0.71073 Å	
Crystal system	Orthorhombic	
Space group	P 21 21 21	
Unit cell dimensions	a = 5.69650(10) Å	α = 90
	b = 11.8449(3) Å	β = 90
	c = 23.1004(6) Å	γ = 90
Volume	1558.69(6) Å^3	
Z	4	
Density (calculated)	1.226 Mg/m^3	

Absorption coefficient	0.236 mm^{-1}
F(000)	616
Crystal shape	bar
Crystal size	0.08 x 0.08 x 0.20
Theta range for data collection	1.932 to 28.302°.
Index ranges	-7<=h<=7, -15<=k<=15, -30<=l<=30
Reflections collected	109815
Independent reflections	3878 [R(int) = 0.0438]
Completeness to $theta_{max}$	99.7 %
Absorption correction	Multi-scan, SADABS[24,25]
Max. and min. transmission	0.8621 and 0.8329
Refinement method	Full-matrix least-squares on F^2
Data / restraints / parameters	3878 / 0 / 187
Goodness-of-fit on F^2	1.073
Final R indices [I>2sigma(I)]	R1 = 0.0275, wR2 = 0.0735
R indices (all data)	R1 = 0.0283, wR2 = 0.0743
Largest diff. peak and hole	0.323 and -0.139 e.$Å^{-3}$
Absolute structure parameters:	
Flack x	0.004 (9)
Hooft y	0.022 (9)

***tert*-Butyl (*S*)-2,5-Diphenyl-2,3,4,7-tetrahydro-1*H*-azepine-1-carboxylate (Int-1)**

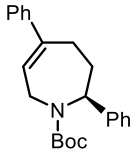

Following **GP7**, **1** (25 mg, 0.1 mmol), gave **Int-1** (47%, 80:20 er) as a solid. R_f 0.75 [pentane:EtOAc (9:1)]; HPLC analysis of **Int-1**: Chiralpak® IA, hexane: *i*-PrOH 98:2, flow 0.75 mL/min, oven temperature 30 °C, t_r = 14.14 min (major) and t_r = 16.49 min (minor); $[\alpha]_D$ = –4.7 (c = 0.57, $CHCl_3$, determined on (*S*)-**Int-1** after purification by chiral HPLC >99:1 er); ^1H NMR (600 MHz, $CDCl_3$, rotamers) δ 7.40–7.28 (9H, m), 7.28–7.22 (1H, m), 6.02 (0.5H, br d, *J* = 7.1 Hz), 5.93 (0.5H, br d, *J* = 6.6 Hz), 5.55 (0.6H, dd, *J* = 12.3, 5.8 Hz), 5.24 (0.4H, dd, *J* = 12.3, 5.5 Hz), 4.58 (0.4H, dd, *J* = 17.6, 7.0 Hz), 4.26 (0.6H, dd, *J* = 17.5, 6.7 Hz), 3.82 (0.4H, d, *J* = 17.3 Hz), 3.69 (0.6H, d, *J* = 17.5 Hz), 2.89–2.67 (2H, m), 2.56–2.37 (1H, m), 2.33 (1H, q, *J* = 13.1 Hz), 1.47 (5H, s), 1.39 (4H, s); ^{13}C NMR (151 MHz, $CDCl_3$, rotamers) δ 156.7, 155.9, 144.1, 143.7, 143.0, 142.8, 142.2, 142.1, 128.6, 128.4, 128.3, 127.0, 126.4, 126.0, 125.9, 80.0, 79.8, 60.3, 58.3, 41.2, 40.6, 32.7, 31.8, 31.1, 30.4, 28.6, 28.5; HRMS (ESI): found MNa^+ 372.1948, $C_{23}H_{27}NO_2Na$ requires 372.1934.

The main enantiomer of **Int-1** was isolated by preparative chiral HPLC (Chiralpak® IA, 250 x 20 mm, hexane:*i*-PrOH 99.2:0.8, 12 ml /min 15 bar, UV detector 230 nm.) for crystallization. Suitable single crystal of (*S*)-**Int-1** for XRD analysis (see **Figure S5**) was obtained by slow evaporation of toluene at room temperature:

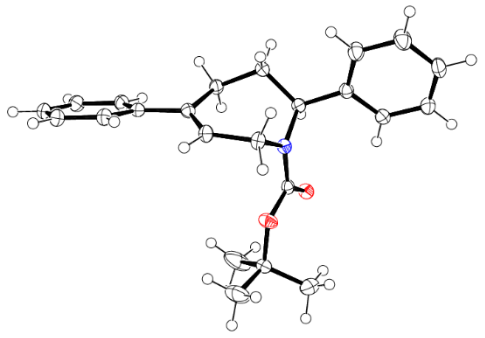

Figure S5. ORTEP plot of (*S*)-**Int-1** with thermal ellipsoids set at the 50% probability level.[17]

Crystallization of compound (*S*)-**Int-1** ($C_{23}H_{27}NO_2$) from toluene at room temperature resulted in crystals of good quality for X-ray analysis. Crystallographic data were collected on a Bruker D8 Adventure diffractometer[18] with monochromatic Cu–Kα radiation (λ=1.54178 Å) and a PHOTONIII detector. The structure was solved by intrinsic phasing using the ShelXT 2018/2 structure solution program[19] and refined against F^2 on all data by full-matrix least-squares methods using ShelXL-2018/3[20] and Olex2[21]. All non-hydrogen atoms were refined anisotropically. The carbon bound hydrogen atoms were placed at idealised positions and refined isotropically using the riding model. Flack x parameter was determined using 1741 quotients [(I+)-(I-)]/[(I+)+(I-)].[22] The Hooft Parameter was determined as 0.01(2).[23]

CCDC	2540109	
Empirical formula	$C_{23}H_{27}NO_2$	
Formula weight	349.45	
Temperature	100.0 K	
Wavelength	1.54178 Å	
Crystal system	Orthorhombic	
Space group	P 21 21 21	
Unit cell dimensions	a = 6.37180(10) Å	α = 90
	b = 9.9032(2) Å	β = 90
	c = 31.2048(7) Å	γ = 90
Volume	1969.06(7) $Å^3$	
Z	4	

Density (calculated)	1.179 Mg/m^3
Absorption coefficient	0.582 mm^{-1}
F(000)	752
Crystal shape	irregular block
Theta range for data collection	4.684 to 79.090°.
Index ranges	-8<=h<=7, -12<=k<=11, -39<=l<=39
Reflections collected	43668
Independent reflections	4215 [R(int) = 0.0232]
Completeness to theta$_{max}$	99.1 %
Absorption correction	Multi-scan, SADABS[24,25]
Max. and min. transmission	0.7542 and 0.608
Refinement method	Full-matrix least-squares on F^2
Data / restraints / parameters	4215 / 0 / 239
Goodness-of-fit on F^2	1.084
Final R indices [I>2sigma(I)]	R1 = 0.0285, wR2 = 0.074
R indices (all data)	R1 = 0.0285, wR2 = 0.074
Extinction coefficient	0.0053(5)
Largest diff. peak and hole	0.235 and -0.162 e.Å^{-3}
Absolute structure parameters:	
Flack x	-0.02 (3)
Hooft y	0.01 (2)

***tert*-Butyl (2*R*,5*S*)-5-(4-Fluorophenyl)-2-phenylazepane-1-carboxylate (2a)**

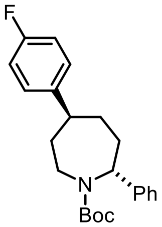

Following **GP7**, **2** (26 mg, 0.1 mmol), gave **2a** (45%, 98:2 er) as an oil. R_f 0.43 [pentane:Et_2O (9:1)]; HPLC analysis of **2a**: Chiralcel® OD-H, hexane: *i*-PrOH 99.5:0.5, flow 0.60 mL/min, oven temperature 30 °C, t_r = 13.16 min (minor) and t_r = 18.40 min (major); $[\alpha]_D$ = +10.8 (c = 0.28, $CHCl_3$); ^{1}H NMR (600 MHz, $CDCl_3$, rotamers) δ 7.34–7.29 (2H, m), 7.26–7.19 (3H, m), 7.18–7.12 (2H, m), 6.98 (2H, q, *J* = 8.9 Hz), 5.29 (0.5H, dd, *J* = 11.6, 6.5 Hz), 5.00 (0.5H, dd, *J* = 12.4, 5.9 Hz), 4.32–4.23 (0.5H, m), 4.01–3.82 (0.5H, m), 3.15 (0.5H, dd, *J* = 14.3, 11.8 Hz), 3.08 (0.5H, dd, *J* = 14.3, 12.1 Hz), 2.68–2.57 (1H, m), 2.56–2.43 (0.5H, m), 2.42–2.28 (0.5H, m), 2.08–1.95 (2H, m), 1.95–1.70 (3H, m), 1.49 (4.5H, s), 1.33 (4.5H, s); ^{13}C NMR (151 MHz, $CDCl_3$, rotamers) δ 162.2, 160.6, 156.2, 156.1, 144.7, 143.5, 143.1 (d, *J* = 3.3 Hz), 142.9 (d, *J* = 3.1 Hz), 128.7, 128.5, 128.3 (d, *J* = 7.7 Hz), 128.2 (d, *J* = 7.6 Hz), 126.9,

126.8, 125.8, 125.6, 115.4 (d, *J* = 14.2 Hz), 115.2 (d, *J* = 14.1 Hz), 79.9, 79.7, 60.9, 58.7, 47.73, 47.7, 42.4, 42.1, 38.7, 38.3, 35.8, 34.7, 34.6, 33.2, 28.7, 28.5; ^{19}F NMR (564 MHz, $CDCl_3$, rotamers) δ –117.22 to –117.29 (m), –117.32 to –117.40 (m); HRMS (ESI): found MNa^+ 392.1995, $C_{23}H_{28}NO_2FNa$ requires 392.1996.

***tert*-Butyl-(2*R*,5*S*)-2-Phenyl-5-(4-(trifluoromethyl)phenyl)azepane-1-carboxylate (3a)**

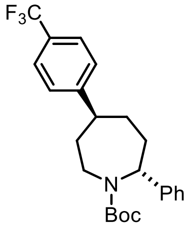

Following **GP7**, **3** (31 mg, 0.1 mmol), gave **3a** (48%, 91.5:8.5 er) as an oil. R_f 0.47 [pentane:Et_2O (9:1)]; HPLC analysis of **3a:** Chiralpak® AD-H, hexane: *i*-PrOH 99:1, flow 0.75 mL/min, oven temperature 30 °C, t_r = 32.05 min (major) and t_r = 35.99 min (minor); $[\alpha]_D$ = +10.4 (c = 0.23, $CHCl_3$); ^{1}H NMR (600 MHz, $CDCl_3$, rotamers) δ 7.58–7.53 (2H, m), 7.35–7.28 (4H, m), 7.26–7.18 (3H, m), 5.31 (0.5H, dd, *J* = 11.3, 6.7 Hz), 5.01 (0.5H, dd, *J* = 12.2, 5.9 Hz), 4.29 (0.5H, ddd, *J* = 14.4, 4.4, 1.6 Hz), 4.00–3.93 (0.5H, m), 3.17 (0.5H, dd, *J* = 14.3, 11.7 Hz), 3.10 (0.5H, dd, *J* = 14.3, 12.0 Hz), 2.71 (1H, m), 2.55–2.46 (0.5H, m), 2.36 (0.5H, ddd, *J* = 14.4, 8.5, 6.0 Hz), 2.08–1.90 (2H, m), 1.90–1.71 (3H, m), 1.49 (4.5H, s), 1.33 (4.5H, s); ^{13}C NMR (151 MHz, $CDCl_3$, rotamers) δ 156.1, 156.0, 151.4, 151.1, 144.6, 143.3, 128.8–128,6 (m), 128.7, 128.5, 127.3, 127.2, 126.9, 126.8, 125.8, 125.7–125.5 (m), 125.5, 124.4 (q, *J* = 271.8 Hz), 80.0, 79.9, 60.8, 58.6, 48.4, 48.3, 42.3, 42.0, 38.4, 37.9, 35.7, 34.6, 34.2, 32.6, 28.7, 28.5; ^{19}F NMR (564 MHz, $CDCl_3$, rotamers) δ –62.3 (s), –62.4 (s); HRMS (ESI): found MNa^+ 442.1963, $C_{24}H_{28}O_2NF_3Na$ requires 442.1964.

***tert*-Butyl (2*R*,5*S*)-5-([1,1'-Biphenyl]-4-yl)-2-phenylazepane-1-carboxylate (4a)**

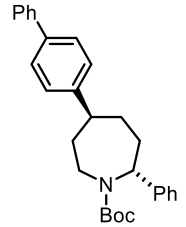

Following **GP7**, **4** (32 mg, 0.1 mmol), gave **4a** (40%, 97:3 er) as an oil. R_f 0.63 [hexane:EtOAc (9:1)]; HPLC analysis of **4a**: Chiralpak® AD-H, hexane: *i*-PrOH 99:1, flow 0.75 mL/min, oven temperature 30 °C, t_r = 30.65 min (major) and t_r = 36.69 min (minor); $[\alpha]_D$ = –7.4 (c = 0.14, $CHCl_3$); ^{1}H NMR (600 MHz, $CDCl_3$, rotamers) δ 7.59 (2H, d, *J* = 7 .9 Hz), 7.54 (2H, t, *J* = 9.1 Hz), 7.45–7.41 (2H, m), 7.36–7.31 (3H, m), 7.30–7.27 (3H, m), 7.25–7.20 (2H, m), 5.32 (0.5H, dd, *J* = 10.9, 7.0 Hz), 5.02 (0.5H, dd, *J* = 12.2, 5.8 Hz), 4.30 (0.5H, dd, *J* = 14.4, 2.8 Hz), 3.98 (0.5H, d, *J* = 14.6), 3.19 (0.5H, dd, *J* = 14.4, 11.6 Hz), 3.16–3.09 (0.5H, m), 2.74–2.65 (1H, m), 2.55–2.47 (0.5H, m), 2.39–2.33 (0.5H,m), 2.14–

1.95 (2H, m), 1.95–1.72 (3H, m), 1.51 (4.5H, s), 1.34 (4.5H, s); ^{13}C NMR (151 MHz, $CDCl_3$, rotamers) δ 156.2, 156.1, 146.6, 146.3, 144.8, 143.6, 141.2, 141.1, 139.3, 139.2, 128.87, 128.85, 128.7, 128.5, 127.42, 127.4, 127.32, 127.31, 127.23, 127.2, 127.1, 126.8, 126.7, 125.8, 125.6, 79.8, 79.75, 60.9, 58.7, 48.2, 48.1, 42.5, 42.2, 38.6, 38.1, 35.9, 34.8, 34.6, 32.9, 28.7, 28.5; HRMS (ESI): found MNa^+ 450.2402, $C_{29}H_{33}NO_2Na$ requires 450.2404.

***tert*-Butyl (2*R*,5*S*)-5-(4-(*tert*-Butyl)phenyl)-2-phenylazepane-1-carboxylate (5a)**

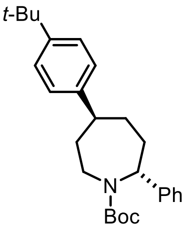

Following **GP7**, **5** (30 mg, 0.1 mmol), gave **5a** (43%, >99:1 er) as a solid. R_f 0.75 [hexane:EtOAc (9:1)]; HPLC analysis of **5a**: Chiralcel® OD-H, hexane: *i*-PrOH 99.5:0.5, flow 0.60 mL/min, oven temperature 30 °C, t_r = 23.47 min (major) and t_r = 24.96 min (minor); $[\alpha]_D$ = +7.9 (c = 0.21, $CHCl_3$); ^{1}H NMR (600 MHz, $CDCl_3$, rotamers) δ 7.36–7.29 (4H, m), 7.25–7.19 (3H, m), 7.16–7.08 (2H, m), 5.29 (0.5H, dd, *J* = 11.5, 6.4 Hz), 5.00 (0.5H, dd, *J* = 12.3, 5.8 Hz), 4.26 (0.5H, ddd, *J* = 14.4, 4.5, 1.6 Hz), 3.98–3.92 (0.5H, m), 3.16 (0.5H, dd, *J* = 14.2, 11.8 Hz), 3.08 (0.5H, dd, *J* = 14.2, 12.1 Hz), 2.67–2.56 (1H, m), 2.51–2.43 (0.5H, m), 2.35–2.29 (0.5H, m), 2.09–1.92 (2H, m), 1.90–1.73 (3H, m), 1.49 (4.5H, s), 1.33 (4.5H, s), 1.32 (4.5H, s), 1.31 (4.5H, s); ^{13}C NMR (151 MHz, $CDCl_3$, rotamers) δ 156.2, 156.1, 149.03, 149.0, 144.9, 144.4, 144.1, 143.7, 128.6, 128.5, 126.8, 126.7, 126.6, 126.5, 125.8, 125.6, 125.5, 125.4, 79.74, 79.7, 60.9, 58.8, 47.9, 42.5, 42.2, 38.6, 38.0, 35.9, 34.9, 34.6, 33.0, 31.5, 30.5, 29.9, 28.7, 28.5; HRMS (ESI): found MNa^+ 430.2714, $C_{27}H_{37}NO_2Na$ requires 430.2717.

***tert*-Butyl (2*R*,5*S*)-5-(4-Methoxyphenyl)-2-phenylazepane-1-carboxylate (6a)**

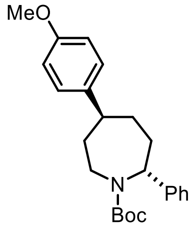

Following **GP7**, **6** (28 mg, 0.1 mmol), gave **6a** (40%, dr 10:1, 85:15 er) as a solid. R_f 0.37 [pentane:Et_2O (9:1)]; HPLC analysis of **6a**: Chiralpak® IA, hexane: *i*-PrOH 99.3:0.7, flow 0.65 mL/min, oven temperature 30 °C, t_r = 31.25 min (major) and t_r = 34.56 min (minor); $[\alpha]_D$ = +11.5 (c = 0.87, $CHCl_3$); ^{1}H NMR (600 MHz, $CDCl_3$, rotamers) δ 7.36–7.18 (5H, m), 7.13 (2H,m), 6.89–6.80 (2H, m), 5.30 (0.5H, q, *J* = 6.0 Hz), 5.00 (0.5H, dd, *J* = 1 2.4, 5.8 Hz), 4.26 (0.5H, dd, *J* = 14.5, 2.9 Hz), 4.00–3.91 (0.5H, m), 3.80 (1.5H, s), 3.79 (1.5H, s), 3.16 (0.5H, dd, *J* = 14.2, 11.8 Hz), 3.08 (0.5H, dd, *J* = 14.2, 12.1 Hz), 2.69–2.54 (1H, m), 2.47 (0.5H, m), 2.32 (0.5H, ddd, m), 2.08–1.96 (2H, m), 1.90–1.72 (3H,

m), 1.50 (4.5H, s), 1.33 (4.5H, s); ^{13}C NMR (151 MHz, $CDCl_3$, rotamers) δ 158.05, 158.0, 156.2, 156.1, 144.9, 143.6, 139.7, 139.5, 128.6, 128.4, 127.8, 127.7, 126.8, 126.7, 125.8, 125.6, 114.0, 113.9, 79.75, 79.7, 60.9, 58.7, 55.4, 47.6, 47.5, 42.4, 42.1, 38.7, 38.3, 35.9, 34.85, 34.8, 33.2, 28.7, 28.5; HRMS (ESI): found MNa^+ 404.2196, $C_{24}H_{31}NO_3Na$ requires 404.2196.

***tert*-Butyl (2*R*,5*S*)-5-(3,5-Dimethylphenyl)-2-phenylazepane-1-carboxylate (7a)**

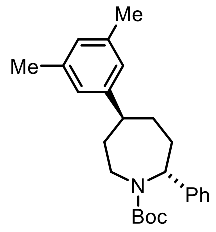

Following **GP7**, **7** (27 mg, 0.1 mmol), gave **7a** (40%, 97:3 er) as a solid. R_f 0.46 [pentane:Et_2O (9:1)]; HPLC analysis of **7a**: Chiralpak® AD-H, hexane: *i*-PrOH 99:1, flow 0.75 mL/min, oven temperature 30 °C, t_r = 11.69 min (minor) and t_r = 15.21 min (major); $[\alpha]_D$ = +13.7 (c = 0.37, $CHCl_3$); ^{1}H NMR (600 MHz, $CDCl_3$, rotamers) δ 7.32 (2H, m), 7.26–7.19 (3H, m), 6.87–6.78 (3H, m), 5.29 (0.5H, dd, *J* = 11.3, 6.7 Hz), 4.99 (0.5H, dd, *J* = 12.3, 5.8 Hz), 4.27 (0.5H, dd, *J* = 14.4, 5.0 Hz), 3.95 (0.5H, dd, *J* = 14.7, 3.4 Hz), 3.15 (0.5H, dd, *J* = 14.5, 11.6 Hz), 3.08 (0.5H, dd, *J* = 14.3, 12.2 Hz), 2.61–2.51 (1H, m), 2.50–2.43 (0.5H, m), 2.35–2.32 (0.5H, m), 2.30 (3H, s), 2.29 (3H, s), 2.06–1.89 (2H, m), 1.89–1.74 (3H, m), 1.50 (4.5H, s), 1.32 (4.5H, s); ^{13}C NMR (151 MHz, $CDCl_3$, rotamers) δ 156.2, 156.1, 147.5, 147.2, 145.0, 143.7, 138.1, 138.0, 128.6, 128.5, 127.95, 127.92, 126.8, 126.7, 125.8, 125.6, 124.9, 124.8, 79.8, 79.7, 61.0, 58.8, 48.5, 48.3, 42.5, 42.3, 38.6, 38.1, 36.0, 34.8, 34.7, 33.0, 28.7, 28.5, 21.5, 21.48; HRMS (ESI): found MNa^+ 402.2404, $C_{25}H_{33}NO_2Na$ requires 402.2404.

***tert*-Butyl (2*R*,5*S*)-2-Phenyl-5-(o-tolyl)azepane-1-carboxylate (8a)**

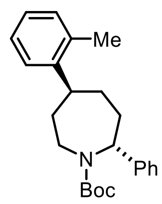

Following **GP7**, **8** (26 mg, 0.1 mmol), gave **8a** (20%, 99:1 er) as an oil. R_f 0.30 [pentane:Et_2O (9:1)]; HPLC analysis of **8a**: Chiralpak® AD-H, hexane: *i*-PrOH 99.2:0.8, flow 1.0 mL/min, oven temperature 30 °C, t_r = 15.18 min (major) and t_r = 21.03 min (minor); $[\alpha]_D$ = +9.8 (c = 0.38, $CHCl_3$); ^{1}H NMR (600 MHz, $CDCl_3$, rotamers) δ 7.35–7.31 (2H, m), 7.28–7.26 (1H, m), 7.25–7.06 (6H, m), 5.33 (0.5H, dd, *J* = 10.9, 7.1 Hz), 5.03 (0.5H, dd, *J* = 12.3, 5.9 Hz), 4.34–4.26 (0.5H, m), 4.01–3.93 (0.5H, m), 3.18 (0.5H, dd, *J* = 14.2, 11.7 Hz), 3.10 (0.5H, dd, *J* = 14.2, 12.1 Hz), 2.92–2.81 (1H, m), 2.55–2.48 (0.5H, m), 2.40–2.34 (0.5H, m), 2.33 (3H, s), 2.00–1.70 (5H, m), 1.50 (4.5H, s), 1.34 (4.5H, s); ^{13}C NMR (151 MHz, $CDCl_3$, rotamers) δ 156.2, 156.1, 145.4, 145.1, 144.8, 143.6, 134.7, 134.68, 130.5, 130.4, 128.6,

128.5, 126.8, 126.7, 126.5, 126.4, 126.0, 125.92, 125.9, 125.8, 125.7, 125.6, 79.8, 79.7, 60.9, 58.6, 43.6, 43.58, 42.8, 42.5, 37.1, 37.0, 36.2, 35.2, 33.7, 32.4, 28.7, 28.5, 19.6, 19.5; HRMS (ESI): found MNa^+ 388.2245, $C_{24}H_{31}NO_2Na$ requires 388.2247.

***tert*-Butyl (2*R*,5*S*)-5-(4-Methylthiophen-2-yl)-2-phenylazepane-1-carboxylate (9a)**

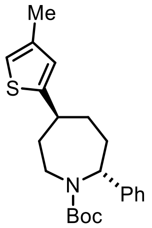

Following **GP7**, **9** (27 mg, 0.1 mmol), gave **9a** (36%. 85:15 er) as a solid. R_f 0.26 [pentane:Et_2O (9:1)]; HPLC analysis of **9a**: Chiralpak® AD-H, hexane: *i*-PrOH 98:02, flow 0.75 mL/min, oven temperature 30 °C, t_r = 12.56 min (major) and t_r = 17.30 min (minor); $[\alpha]_D$ = +15.4 (c = 0.24, $CHCl_3$); ^1H NMR (600 MHz, $CDCl_3$, rotamers) δ 7.34–7.28 (2H, m), 7.26–7.19 (3H, m), 6.69 (1H, d, *J* = 5.6 Hz), 6.63 (1H, d, *J* = 12.3 Hz), 5.26 (0.5H, dd, *J* = 11.9, 6.5 Hz), 4.97 (0.5H, dd, *J* = 12.3, 5.8 Hz), 4.26 (0.5H, ddd, *J* = 14.6, 4.9, 2.1 Hz), 3.94 (0.5H, ddd, *J* = 14.9, 4.6, 2.2 Hz), 3.15 (0.5H, dd, *J* = 14.6, 11.7 Hz), 3.11–3.03 (0.5H, m), 2.94–2.85 (1H, m), 2.46 (0.5H, ddd, *J* = 14.3, 8.6, 6.4 Hz), 2.32 (0.5H, ddd, *J* = 14.7, 8.8, 5.8 Hz), 2.27–2.22 (1H, m) 2.22 (1.5H, s), 2.21 (1.5H, s), 2.21–2.07 (1H, m), 1.91–1.71 (3H, m), 1.49 (4.5H, s), 1.31 (4.5H, s), ^1H NMR contains impurities from the partially reduced product; ^{13}C NMR (151 MHz, $CDCl_3$, rotamers) δ 156.1, 156.0, 151.1, 150.8, 144.7, 143.5, 137.32, 137.26, 128.7, 128.5, 126.9, 126.8, 125.8, 125.6, 125.0, 124.9, 117.7, 79.84, 79.81, 60.7, 58.6, 43.1, 43.0, 42.1, 41.7, 38.9, 38.6, 35.7, 35.4, 34.4, 34.3, 28.7, 28.5, 15.9; HRMS (ESI): found MNa^+ 394.1802, $C_{22}H_{29}NO_2SNa$ requires 394.1811.

***tert*-Butyl (2*R*,5*S*)-2-(4-Methoxyphenyl)-5-phenylazepane-1-carboxylate (10a)**

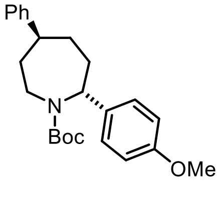

Following **GP7**, **10** (28 mg, 0.1 mmol), gave **10a** (48%, 90:10 er) as an oil. R_f 0.14 [pentane:Et_2O (9:1)]; HPLC analysis of **10a**: Chiralpak® AD-H, hexane: *i*-PrOH 99:1, flow 0.75 mL/min, oven temperature 30 °C, t_r = 26.23 min (major) and t_r = 36.83 min (minor); $[\alpha]_D$ = +7.0 (c = 0.24, $CHCl_3$); ^1H NMR (600 MHz, $CDCl_3$, rotamers) δ 7.33–7.27 (3H, m), 7.23–7.17 (4H, m), 7.14 (1H, d, *J* = 8.5 Hz), 6.89–6.82 (1H, m), 5.25 (0.5H, dd, *J* = 11.5, 6.5 Hz), 4.97 (0.5H, dd, *J* = 12.2, 5.9 Hz), 4.20 (0.5H, ddd, *J* = 14.4, 4.8, 2.1 Hz), 3.90 (0.5H, ddd, *J* = 14.2, 5.3, 2.7 Hz), 3.81 (1.5H, s), 3.79 (1.5H, s), 3.13 (0.5H, dd, *J* = 14.1, 11.8 Hz), 3.06 (0.5H, dd, *J* = 14.1, 12.1 Hz), 2.68–2.59 (1H, m), 2.43 (0.5H, ddd, *J* = 14.5, 8.6, 6.6 Hz), 2.31 (0.5H, ddd, *J* = 14.6, 8.7, 6.0 Hz), 2.07–1.90 (2H, m), 1.91–1.72 (3H, m), 1.49 (4.5H, s),

1.36 (4.5H, s); ^{13}C NMR (151 MHz, $CDCl_3$, rotamers) δ 158.5, 158.4, 156.2, 156.1, 147.5, 147.3, 136.9, 135.7, 128.7, 128.6, 127.1, 127.0, 126.9, 126.8, 126.3, 126.2, 114.0, 113.8, 79.7, 79.6, 60.1, 58.0, 55.4, 48.6, 48.5, 42.2, 42.0, 38.5, 38.0, 35.9, 34.7, 34.3, 32.9, 28.7, 28.5; HRMS (ESI): found MNa^+ 404.2195, $C_{24}H_{31}NO_3Na$ requires 404.2196.

***tert*-Butyl (2*R*,5*S*)-5-Phenyl-2-(*m*-tolyl)azepane-1-carboxylate (11a)**

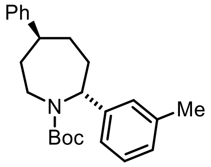

Following **GP7**, **11** (26 mg, 0.1 mmol), gave **11a** (48%, 93:7 er) as an oil. The enantiomeric excess of **11a** was determined after Boc deprotection followed by benzoylation of the free amine. R_f 0.53 [pentane:EtOAc (9:1)]; HPLC analysis of *N*-Bz-**11a**: Chiralpak® IA, hexane: *i*-PrOH 90:10, flow 0.75 mL/min, oven temperature 30 °C, t_r = 14.25 min (major) and t_r = 26.74 min (minor); $[\alpha]_D$ = +19.8 (c = 0.27, $CHCl_3$); ^{1}H NMR (600 MHz, $CDCl_3$, rotamers) δ 7.33–7.27 (2H, m), 7.23–7.17 (4H, m), 7.08–6.98 (3H, m), 5.28–5.23 (0.5H, m), 4.97 (0.5H, dd, *J* = 12.4, 5.8 Hz), 4.25 (0.5H, ddd, *J* = 14.6, 4.8, 2.4 Hz), 3.98–3.92 (0.5H, m), 3.16 (0.5H, dd, *J* = 14.5, 10.7 Hz), 3.10 (0.5H, dd, *J* = 14.8, 11.8 Hz), 2.69–2.58 (1H, m), 2.49–2.42 (0.5H, m), 2.34 (3H, s), 2.32 (0.5H, dd, *J* = 7.7, 5.2 Hz), 2.07–1.74 (5H, m), 1.49 (4.5H, s), 1.34 (4.5H, s); ^{13}C NMR (151 MHz, $CDCl_3$, rotamers) δ 156.2, 156.1, 147.5, 147.3, 144.7, 143.6, 138.1, 138.0, 128.65, 128.6, 128.4, 127.6, 127.4, 127.0, 126.9, 126.7, 126.4, 126.3, 126.2, 122.74, 122.7, 79.8, 79.7, 60.8, 58.8, 48.6, 48.5, 42.5, 42.2, 38.6, 38.1, 35.8, 34.9, 34.5, 33.0, 28.7, 28.5, 21.8, 21.7; HRMS (ESI): found MNa^+ 388.2245, $C_{24}H_{31}N_2O_2Na$ requires 388.2247.

***tert*-Butyl (2*R*,5*S*)-5-Phenyl-2-(4-(trifluoromethyl)phenyl)azepane-1-carboxylate (12a)**

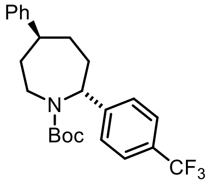

Following **GP7**, **12** (31 mg, 0.1 mmol), gave **12a** (47%, 75:25 er) as an oil. R_f 0.67 [pentane:EtOAc (9:1)]; HPLC analysis of **12a**: Chiralpak® AD-H, hexane: *i*-PrOH 99:1, flow 0.60 mL/min, oven temperature 30 °C, t_r = 13.72 min (major) and t_r = 24.20 min (minor); $[\alpha]_D$ = +4.6 (c = 0.65, $CHCl_3$); ^{1}H NMR (600 MHz, $CDCl_3$, rotamers) δ 7.58 (2H, d, *J* = 8.1 Hz), 7.36 (1H, d, *J* = 8.0 Hz), 7.34–7.28 (3H, m), 7.23–7.17 (3H, m), 5.33 (0.5H, dd, *J* = 11.7, 6.4 Hz), 5.05 (0.5H, dd, *J* = 12.0, 5.7 Hz), 4.31 (0.5H, ddd, *J* = 14.7, 5.0, 2.1 Hz), 4.01 (0.5H, ddd, *J* = 14.7, 4.6, 2.2 Hz), 3.15 (0.5H, dd, *J* = 14.6, 11.1 Hz), 3.09 (0.5H, dd, *J* = 14.8, 11.0 Hz), 2.69–2.61 (1H, m), 2.52–2.44 (0.5H, m), 2.37–2.30 (0.5H, m), 2.13–1.95 (2H, m), 1.92–1.74 (3H, m), 1.50 (4.5H, s), 1.33 (4.5H, s); ^{13}C NMR (151 MHz, $CDCl_3$, rotamers) δ 156.1, 155.8, 149.0, 147.8, 147.1, 146.9, 129.2 (q, *J* = 32.1 Hz), 128.7, 128.6, 127.0, 126.9,

126.41, 126.38, 126.1, 125.9, 125.7 (q, *J* = 3.9 Hz), 125.5 (q, *J* = 3.8 Hz), 124.4 (q, *J* = 272.4 Hz), 80.1, 60.9, 58.9, 48.4, 48.35, 42.6, 42.3, 38.6, 38.2, 35.8, 34.8, 34.6, 33.1, 28.7, 28.4; ^{19}F NMR (565 MHz, $CDCl_3$, rotamers) δ –62.33(s), –62.41(s); HRMS (ESI): found MNa^+ 442.1966, $C_{24}H_{28}NO_2F_3Na$ requires 442.1964.

***tert*-Butyl (2*R*,5*S*)-2-(6-Methoxypyridin-3-yl)-5-phenylazepane-1-carboxylate (13a)**

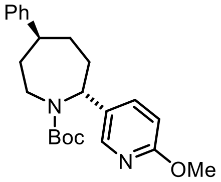

Following **GP7**, **13** (28 mg, 0.1 mmol), gave **13a** (20%, 95:5 er) as a solid. R_f 0.13 [pentane:EtOAc (9:1)]; HPLC analysis of **13a**: Chiralpak® AD-H, hexane: *i*-PrOH 98:2, flow 0.75 mL/min, oven temperature 30 °C, t_r = 20.14 min (major) and t_r = 29.52 min (minor); $[\alpha]_D$ = +23.6 (c = 0.33, $CHCl_3$); ^{1}H NMR (600 MHz, $CDCl_3$, rotamers) δ 8.08 (0.5H, d, *J* = 2.5 Hz), 8.05 (0.5H, d, *J* = 2.6 Hz), 7.50 (0.5H, dd, *J* = 8.6, 2.6 Hz), 7.45 (0.5H, dd, *J* = 8.5, 2.6 Hz), 7.34–7.26 (2H, m), 7.25–7.16 (3H, m), 6.72 (0.5H, s), 6.70 (0.5H, s), 5.25 (0.5H, dd, *J* = 11.5, 6.4 Hz), 4.97 (0.5H, dd, *J* = 12.4, 5.9 Hz), 4.19 (0.5H, ddd, *J* = 14.5, 4.8, 2.1 Hz), 3.93 (1.5H, s), 3.92 (1.5H, s), 3.91–3.87 (0.5H, m), 3.11 (0.5H, dd, *J* = 14.5, 11.6 Hz), 3.03 (0.5H, dd, *J* = 14.3, 12.1 Hz), 2.70–2.58 (1H, m), 2.43 (0.5H, ddd, *J* = 14.6, 8.7, 6.6 Hz), 2.27 (0.5H, ddd, *J* = 14.7, 8.6, 5.9 Hz), 2.09–1.90 (2H, m), 1.91–1.72 (3H, m), 1.49 (4.5H, s), 1.39 (4.5H, s); ^{13}C NMR (151 MHz, $CDCl_3$, rotamers) δ 163.34, 163.3, 156.1, 155.7, 147.3, 147.0, 144.4, 144.1, 137.2, 136.4, 132.7, 131.6, 128.7, 128.6, 126.9, 126.8, 126.34, 126.3, 110.8, 110.6, 80.1, 79.9, 58.1, 56.2, 53.54, 53.5, 48.5, 48.4, 42.2, 42.0, 38.4, 37.9, 35.6, 34.4, 34.3, 32.8, 28.7, 28.6; HRMS (ESI): found MNa^+ 405.2148, $C_{23}H_{30}N_2O_3Na$ requires 405.2149.

***tert*-Butyl (2*R*,5*S*)-2-(Furan-2-yl)-5-phenylazepane-1-carboxylate (14a)**

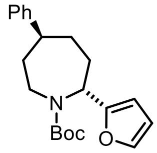

Following **GP7**, **14** (24 mg, 0.1 mmol), gave **14a** (70%, 74:26 er) as a solid. R_f 0.33 [pentane:EtOAc (9:1)]; HPLC analysis of **14a**: Chiralpak® AD-H, hexane: *i*-PrOH 99.2:0.8, flow 0.75 mL/min, oven temperature 30 °C, t_r = 18.65 min (major) and t_r = 22.94 min (minor); $[\alpha]_D$ = +5.8 (c = 0.99, $CHCl_3$); ^{1}H NMR (600 MHz, $CDCl_3$, rotamers) δ 7.38–7.32 (1H, m), 7.33–7.26 (2H, m), 7.24–7.15 (3H, m), 6.33–6.29 (1H, m), 6.17 (0.6H, d, *J* = 3.2 Hz), 6.10 (0.4H, d, *J* = 3.2 Hz), 5.37 (0.5H, dd, *J* = 12.1, 6.9 Hz), 5.12 (0.5H, dd, *J* = 12.2, 6.6 Hz), 4.00 (0.5H, ddd, *J* = 14.6, 4.5, 2.4 Hz), 3.77 (0.5H, dt, *J* = 14.8, 3.3 Hz), 3.06 (1H, *J* = 13.4 Hz), 2.69–2.61 (1H, m), 2.42 (0.5H, dt, *J* = 15.2, 7.7 Hz), 2.40–2.32 (0.5H, m), 2.05–1.87 (3H, m), 1.87–1.70 (2H, m), 1.50 (4.5H, s), 1.49 (4.5H, s); ^{13}C NMR (151 MHz, $CDCl_3$,

rotamers) δ 156.4, 156.0, 155.95, 155.6, 147.5, 147.3, 141.8, 141.6, 128.7, 128.6, 127.0, 126.9, 126.3, 126.2, 110.0, 105.5, 105.1, 79.9, 79.8, 53.7, 52.3, 48.8, 48.7, 41.8, 41.3, 38.2, 37.5, 33.1, 32.1, 32.0, 31.5, 28.7, 28.6; HRMS (ESI): found MNa$^+$ 364.1884, $C_{21}H_{27}NO_3Na$ requires 364.1883.

***tert*-Butyl (2*R*,5*S*)-5-Methyl-2-phenylazepane-1-carboxylate (15a)**

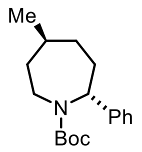

Following **GP7**, **15** (18 mg, 0.1 mmol), gave **15a** (48%, 96:4 er) as an oil. The enantiomeric excess of **15a** was determined after Boc deprotection followed by benzoylation of the free amine. R_f 0.36 [pentane:Et_2O (9:1)]; HPLC analysis of *N*-Bz-**15a**: Chiralcel® OJ-H, hexane: *i*-PrOH 99.2:0.8, flow 1.0 mL/min, oven temperature 30 °C, t_r = 18.72 min (major) and t_r = 31.94 min (minor); $[\alpha]_D$ = +41 (c = 0.14, $CHCl_3$); ^{1}H NMR (600 MHz, $CDCl_3$, rotamers) δ 7.32–7.26 (2H, m), 7.23–7.14 (3H, m), 5.19 (0.5H, dd, *J* = 12.4, 6.5 Hz), 4.91 (0.5H, dd, *J* = 12.5, 5.9 Hz), 4.13 (0.5H, ddd, *J* = 14.3, 4.8, 2.1 Hz), 3.88–3.81 (0.5H, m), 3.03 (0.5H, dd, *J* = 14.4, 11.8 Hz), 2.96 (0.5H, dd, *J* = 14.6, 12.0 Hz), 2.33 (0.5H, ddd, *J* = 15.0, 8.6, 6.6 Hz), 2.20 (0.5H, ddd, *J* = 14.9, 8.8, 6.0 Hz), 1.87–1.65 (3H, m), 1.52–1.49 (1H, m), 1.48 (4.5H, s), 1.30 (4.5H, s), 1.28–1.23 (2H, m), 0.95 (3H, d, *J* = 6.5Hz); ^{13}C NMR (151 MHz, $CDCl_3$, rotamers) δ 156.2, 156.1, 145.1, 144.0, 128.6, 128.4, 126.7, 126.6, 125.8, 125.6, 79.6, 79.4, 60.9, 58.8, 42.4, 42.1, 38.6, 38.4, 36.63, 36.6, 35.4, 35.1, 34.5, 34.3, 28.7, 28.5, 23.3, 23.1; HRMS (ESI): found MNa$^+$ 312.1935, $C_{18}H_{27}NO_2Na$ requires 312.1934.

***tert*-Butyl (2*R*,5*S*)-5-Ethyl-2-phenylazepane-1-carboxylate (16a)**

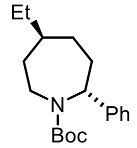

Following **GP7**, **16** (20 mg, 0.1 mmol), gave **16a** (62%, 90:10 er) as a solid. The enantiomeric excess of **16a** was determined after Boc deprotection followed by benzoylation of the free amine. R_f 0.45 [pentane:Et_2O (9:1)]; HPLC analysis of *N*-Bz-**16a**: Chiralcel® OJ-H, hexane: *i*-PrOH 98.5:1.5, flow 1.0 mL/min, oven temperature 30 °C, t_r = 17.17 min (major) and t_r = 27.93 (minor); $[\alpha]_D$ = +40.5 (c = 0.78, $CHCl_3$); ^{1}H NMR (600 MHz, $CDCl_3$, rotamers) δ 7.32–7.26 (2H, m), 7.24–7.15 (3H, m), 5.18 (0.5H, dd, *J* = 12.4, 6.5 Hz), 4.90 (0.5H, dd, *J* = 12.5, 5.9 Hz), 4.16 (0.5H, dd, *J* = 14.1, 4.4 Hz), 3.88 (0.5H, dd, *J* = 14.2, 3.9 Hz), 3.02 (0.5H, dd, *J* = 14.5, 10.6 Hz), 2.95 (0.5H, dd, *J* = 14.7, 10.8 Hz), 2.36 (0.5H, ddd, *J* = 15.0, 8.7, 6.5 Hz), 2.23 (0.5H, ddd, *J* = 14.8, 8.7, 5.9 Hz), 1.93–1.80 (2H, m), 1.81–1.61 (2H, m), 1.48 (4.5H, s), 1.30 (4.5H, s), 1.30–1.24 (4H, m), 0.89 (3H, t, *J* = 7.1 Hz); ^{13}C NMR (151 MHz, $CDCl_3$, rotamers) δ 156.2, 156.1, 145.1, 143.9, 128.5, 128.4, 126.7, 126.6, 125.8, 125.6, 79.5, 79.4,

60.8, 58.7, 43.2, 43.1, 42.4, 42.0, 36.0, 35.7, 35.4, 34.4, 32.7, 31.7, 30.3, 30.2, 28.7, 28.5, 11.9, 11.86; HRMS (ESI): found MNa+ 326.2092, $C_{19}H_{29}NO_2Na$ requires 326.2091.

***tert*-Butyl (2*R*,5*S*)-5-*iso*-Propyl-2-phenylazepane-1-carboxylate (17a)**

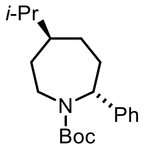

Following **GP7**, **17** (21 mg, 0.1 mmol), gave **17a** (46%, 97:3 er) as an oil. The enantiomeric excess of **17a** was determined after Boc deprotection followed by benzoylation of the free amine. R_f 0.32 [pentane:Et_2O (9:1)]; HPLC analysis of *N*-Bz-**17a**: Chiralpak® IA, hexane: *i*-PrOH 90:10, flow 0.75 mL/min, oven temperature 30 °C, t_r = 12.98 min (major) and t_r = 21.50 min (minor); $[\alpha]_D$ = +57.4 (c = 0.20, $CHCl_3$ measured on *N*-Bz-**17a**); ^{1}H NMR (600 MHz, $CDCl_3$, rotamers) δ 7.32–7.26 (2H, m), 7.24–7.14 (3H, m), 5.18 (0.5H, dd, *J* = 12.3, 6.6 Hz), 4.89 (0.5H, dd, *J* = 12.5, 5.9 Hz), 4.18 (0.5H, ddd, *J* = 14.4, 5.0, 2.2 Hz), 3.88 (0.5H, ddd, *J* = 14.7, 4.4, 2.3 Hz), 3.00 (0.5H, dd, *J* = 14.4, 11.7 Hz), 2.92 (0.5H, dd, *J* = 13.2, 12.3 Hz), 2.39 (0.5H, ddd, *J* = 15.1, 8.8, 6.6 Hz), 2.25 (0.5H, ddd, *J* = 14.8, 8.8, 5.9 Hz), 1.87–1.71 (2H, m), 1.71–1.60 (3H, m), 1.48 (4.5H, s), 1.45–1.36 (2H, m), 1.30 (4.5H, s), 0.87 (6H, dd, *J* = 6.8, 3.7 Hz); ^{13}C NMR (151 MHz, $CDCl_3$, rotamers) δ 156.3, 156.1, 145.1, 143.8, 128.5, 128.4, 126.7, 126.6, 125.8, 125.6, 79.6, 79.4, 60.8, 58.6, 47.6, 47.5, 42.4, 42.1, 35.6, 34.5, 33.2, 32.4, 31.8, 29.7, 28.7, 28.6, 28.5, 20.0, 19.8, 19.4, 19.3; HRMS (ESI): found MNa+ 340.2245, $C_{20}H_{31}NO_2Na$ requires 340.2247.

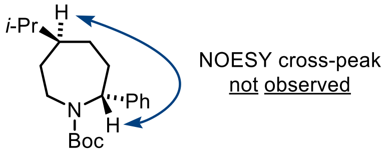

***tert*-Butyl (2*R*,5*S*)-5-Benzyl-2-phenylazepane-1-carboxylate (18a)**

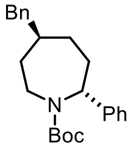

Following **GP7**, **18** (26 mg, 0.1 mmol), gave **18a** (60%, 90:10 er) as a solid. R_f 0.65 [pentane:EtOAc (9:1)]; HPLC analysis of **18a**: Chiralpak® AD-H, hexane: *i*-PrOH 99.6:0.4, flow 0.75 mL/min, oven temperature 30 °C, t_r = 25.23 min (minor) and t_r = 29.48 min (major); $[\alpha]_D$ = +28.8 (c = 1.46, $CHCl_3$); ^{1}H NMR (600 MHz, $CDCl_3$, rotamers) δ 7.31–7.25 (4H, m), 7.23–7.16 (3H, m), 7.17–7.12 (3H, m), 5.18 (0.4H, dd, *J* = 12.4, 6.5 Hz), 4.90 (0.6H, dd, *J* = 12.5, 5.8 Hz), 4.12 (0.6H, ddd, *J* = 14.7, 5.0, 2.1 Hz), 3.87–3.81 (0.4H, m), 2.98 (0.6H, dd, *J* = 14.5, 11.8 Hz), 2.92 (0.4H, dd, *J* = 14.4, 12.2 Hz), 2.54 (2H, d, *J* = 7.1 Hz), 2.32 (0.4H, ddd, *J* = 15.1, 8.7, 6.6 Hz), 2.19 (0.6H, ddd, *J* = 14.8, 8.7, 5.8 Hz),

1.95–1.84 (1H, m), 1.75 (1H, m), 1.69–1.63 (2H, m), 1.47 (4.5H, s), 1.42–1.32 (1H, m), 1.30 (4.5H, s), 1.29–1.23 (1H, m); ^{13}C NMR (151 MHz, $CDCl_3$, rotamers) δ 156.2, 156.1, 144.9, 143.8, 141.2, 141.1, 129.3, 129.28, 128.5, 128.4, 128.37, 128.34, 126.7, 126.6, 126.0, 125.98, 125.8, 125.6, 79.7, 79.5, 60.8, 58.7, 44.2, 44.1, 43.6, 43.4, 42.2, 41.8, 36.4, 36.2, 35.2, 34.3, 32.7, 31.6, 28.7, 28.5; HRMS (ESI): found MNa^+ 388.2246, $C_{24}H_{31}NO_2Na$ requires 388.2247.

***tert*-Butyl (2*R*,5*S*)-2-Phenyl-5-(trifluoromethyl)azepane-1-carboxylate (19a)**

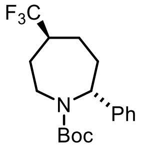

Following **GP7**, **19** (24 mg, 0.1 mmol), gave **19a** (26%, dr 1.3:1, 53:47 er*)* as an oil. R_f 0.41 [pentane:Et_2O (9:1)]; HPLC analysis of **19a**: Chiralcel® OD-H, hexane: *i*-PrOH 99.2:0.8, flow 0.75 mL/min, oven temperature 30 °C, t_r = 17.88 min and t_r = 19.18 min; ^{1}H NMR (600 MHz, $CDCl_3$, rotamers) δ 7.34–7.28 (2H, m), 7.26–7.13 (3H, m), 5.25 (0.5H, dd, *J* = 12.2, 6.7 Hz), 4.96 (0.5H, dd, *J* = 12.4, 6.0 Hz), 4.28 (0.5H, dd, *J* = 16.1, 4.1 Hz), 3.97 (0.5H, d, *J* = 15.0 Hz), 3.02 (0.5H, dd, *J* = 14.7, 11.9 Hz), 2.95 (0.5H, dd, *J* = 15.0, 12.1 Hz), 2.51 (0.5H, ddd, *J* = 15.2, 8.4, 6.5 Hz), 2.37 (0.5H, ddd, *J* = 15.1, 8.9, 6.1 Hz), 2.23–2.07 (2H, m), 2.07–1.93 (1H, m), 1.78–1.63 (2H, m), 1.59–1.57 (0.5H, m), 1.56–1.53 (0.5H, m), 1.49 (4.5H, s), 1.31 (4.5H, s); ^{13}C NMR (151 MHz, $CDCl_3$, rotamers) δ 155.9, 155.8, 143.9, 142.7, 128.7, 128.6, 127.1, 127.0, 125.7, 125.5, 80.2, 60.2, 58.0, 45.5 (q, *J* = 25.2 Hz), 40.8, 40.4, 33.8, 32.7, 29.9, 28.9, 28.6, 28.4, 25.0, 24.2; ^{19}F NMR (564 MHz, $CDCl_3$, rotamers) δ –73.16 (d, *J* = 8.4 Hz), –73.25 (d, *J* = 8.4 Hz); HRMS (ESI): found MNa^+ 366.1655, $C_{18}H_{24}NO_2F_3Na$ requires 366.1651.

***tert*-Butyl (2*R*,5*S*)-2-*iso*-propyl-5-phenylazepane-1-carboxylate (20a)**

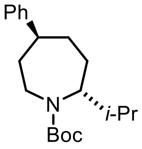

Following **GP7**, **20** (21 mg, 0.1 mmol), gave **20a** (20%, 85:15 er) as an oil. The enantiomeric excess of **20a** was determined after Boc deprotection followed by benzoylation of the free amine. R_f 0.53 [pentane:Et_2O (9:1)]; HPLC analysis of *N*-Bz-**20a:** Chiralpak® IA, hexane: *i*-PrOH 90:10, flow 0.75 mL/min, oven temperature 30 °C, t_r = 14.13 min (major) and t_r = 16.22 min (minor); $[\alpha]_D$ = +24.2 (c = 0.14, $CHCl_3$ measured on *N*-Bz-**20a**); ^{1}H NMR (600 MHz, $CDCl_3$, rotamers) δ 7.31–7.24 (2H, m), 7.21–7.13 (3H, m), 3.95–3.89 (0.5H, m), 3.89–3.84 (0.5H, m), 3.76–3.70 (0.5H, m), 3.70 (0.5H, app t, *J* = 3.2 Hz), 2.82 (1H, app q, *J* = 12.2 Hz), 2.59–2.49 (1H, m), 2.22 (0.5H, dt, *J* = 14.9, 7.6 Hz), 2.16 (0.5H, ddd, *J* = 14.9, 8.6, 6.7 Hz), 1.92–1.77 (2H, m), 1.77–1.53 (3H, m), 1.50 (4.5H, s), 1.48 (4.5H, s), 1.45–1.34 (1H, m), 0.93 (3H, dd, *J* = 7.8, 6.7 Hz), 0.90 (3H, d, *J* = 6.8 Hz); ^{13}C NMR (151 MHz, $CDCl_3$,

rotamers) δ 156.5, 156.2, 148.0, 147.8, 128.6, 128.5, 127.0, 126.9, 126.15, 126.1, 79.3, 79.0, 61.2, 60.1, 49.1, 49.0, 41.6, 41.3, 38.0, 36.4, 33.6, 33.4, 33.2, 32.3, 31.4, 31.1, 28.75, 28.5, 20.2, 19.9, 19.3, 19.2; HRMS (ESI): found MNa$^+$ 340.2237, $C_{20}H_{31}NO_2Na$ requires 340.2247.

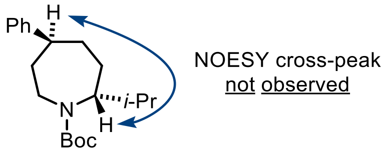

***tert*-Butyl (2*R*,5*S*)-2,5-Diisopropylazepane-1-carboxylate (21a)**

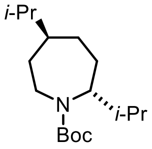

Following **GP7**, **21** (18 mg, 0.1 mmol), gave **21a** (15%, 94:6 er) as an oil. The enantiomeric excess of **21a** was determined after Boc deprotection followed by benzoylation of the free amine. R_f 0.59 [pentane:Et_2O (9:1)]; HPLC analysis of *N*-Bz-**21a**: Chiralcel® OD-H, hexane: *i*-PrOH 99:1, flow 0.75 mL/min, oven temperature 30°C, t_r = 22.07 min (major) and t_r = 22.07 min (minor); $[\alpha]_D$ = +11.7 (c = 0.23, $CHCl_3$ measured on *N*-Bz-**21a**); ^{1}H NMR (600 MHz, $CDCl_3$, rotamers) δ 3.82–3.78 (0.5H, m), 3.80–3.74 (0.5H, m), 3.64 (0.5H, dt, *J* = 14.4, 3.3 Hz), 3.63–3.57 (0.5H, m), 2.70–2.65 (0.5H, m), 2.66–2.62 (0.5H, m), 2.12 (0.5H, ddd, *J* = 15.2, 8.9, 7.1 Hz), 2.11–2.02 (0.5H, m), 1.69–1.57 (2H, m), 1.57–1.48 (2H, m), 1.46 (4.5H, s), 1.45 (4.5H, s), 1.45–1.32 (1H, m), 1.25–1.11 (2H, m), 1.07–0.96 (1H, m), 0.90–0.84 (6H, m), 0.84–0.80 (6H, m); ^{13}C NMR (151 MHz, $CDCl_3$, rotamers) δ 156.4, 156.1, 78.9, 78.6, 61.1, 59.9, 47.9, 47.8, 41.3, 41.0, 33.3, 33.2, 33.15, 33.1, 30.8, 30.6, 30.4, 28.6, 28.5, 28.1, 27.7, 20.0, 19.85, 19.8, 19.6, 19.2, 19.15, 19.1, 19.0; HRMS (ESI): found MNa$^+$ 306.2400, $C_{24}H_{31}O_3NNa$ requires 306.2404.

***tert*-Butyl (2*R*,6*R*)-2,6-Diphenylazepane-1-carboxylate (22a)**

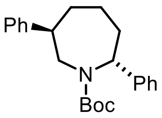

Following **GP7**, **22** (25 mg, 0.1 mmol), gave **22a** (35%, dr 1.5:1, 83:17 er) as an oil. The enantiomeric excess of **22a** was determined after Boc deprotection. R_f 0.51 [hexane:EtOAc (9:1)]; HPLC analysis of *NH*-**22a**: Chiralcel® OJ-H, hexane: *i*-PrOH 99:1, flow 0.75 mL/min, oven temperature 30 °C, t_r = 23.81 min (minor) and t_r = 25.99 min (major); $[\alpha]_D$ = +8.7 (c = 0.23, $CHCl_3$); ^{1}H NMR (600 MHz, $CDCl_3$, rotamers) δ 7.33–7.28 (5H, m), 7.25–7.17 (5H, m), 5.29 (0.6H, dd, *J* = 12.1, 6.9 Hz), 5.04 (0.4H, dd, *J* = 12.3, 6.4 Hz), 4.10 (0.4H, d, *J* = 13.9 Hz), 3.84 (0.6H, d, *J* = 14.2 Hz), 3.16 (0.4H, dd, *J* = 14.1, 10.7 Hz), 3.11 (0.6H, dd, *J* = 14.3, 10.8 Hz), 3.00–2.88 (1H, m), 2.54 (0.6H, dt, *J* = 15.0, 7.7 Hz), 2.42 (0.4H, ddd, *J* = 14.9, 8.7, 6.8 Hz), 2.13–1.98 (2H, m), 1.84–1.76 (1H, m), 1.64–1.56 (2H, m), 1.52 (4.5H, s),

1.36 (4.5H, s); ^{13}C NMR (151 MHz, $CDCl_3$, rotamers) δ 156.3, 156.1, 144.7, 144.6, 144.4, 143.5, 128.7, 128.6, 128.52, 128.5, 127.3, 127.1, 126.8, 126.7, 126.6, 126.4, 125.8, 125.7, 79.9, 79.8, 60.1, 58.2, 49.6, 49.3, 47.3, 46.7, 37.5, 37.3, 36.4, 35.7, 28.8, 28.5, 25.6, 25.0; HRMS (ESI): found MNa^+ 374.2090, $C_{23}H_{29}O_2NNa$ requires 374.2091.

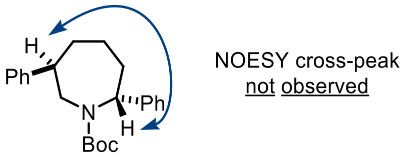

***tert*-Butyl (*R*)-5-Methyl-2,6-diphenyl-2,3,4,7-tetrahydro-1*H*-azepine-1-carboxylate (23a)**

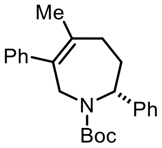

Following **GP7**, **23** (26 mg, 0.1 mmol), gave **23a** (quant., 70:30 er) as an oil. R_f 0.10 [pentane:Et_2O (95:5)]; HPLC analysis of **23a**: Chiralpak® IA, hexane: *i*-PrOH 98:2, flow 0.75 mL/min, oven temperature 30 °C, t_r = 7.77 min (minor) and t_r = 9.09 min (major); ^{1}H NMR (600 MHz, $CDCl_3$, rotamers) δ 7.40–7.27 (7H, m), 7.22 (3H, q, *J* = 10.2, 7.5 Hz), 5.45 (0.6H, dd, *J* = 12.2, 5.9 Hz), 5.19 (0.4H, dd, *J* = 12.1, 5.7 Hz), 4.51 (0.4H, d, *J* = 17.3 Hz), 4.27 (0.6H, d, *J* = 17.4 Hz), 3.92 (1H, dd, *J* = 30.4, 17.5 Hz), 2.53 (1H, q, *J* = 16.5 Hz), 2.45–2.30 (2H, m), 2.25 (1H, q, *J* = 12.7 Hz), 1.60 (2H, s), 1.59 (1H, s), 1.52 (5H, s), 1.43 (4H, s); ^{13}C NMR (151 MHz, $CDCl_3$, rotamers) δ 156.7, 155.9, 143.0, 143.0, 142.9, 142.3, 135.0, 134.5, 133.8, 132.9, 129.5, 129.1, 128.6, 128.5, 128.2, 128.1, 126.9, 126.5, 126.4, 126.2, 125.9, 80.2, 79.8, 59.8, 58.0, 47.1, 46.8, 34.1, 33.2, 32.4, 31.7, 28.8, 28.6, 23.0, 22.9. HRMS (ESI): found MNa^+ 386.2091, $C_{24}H_{29}O_2NNa$ requires 386.2090.

7.1 Preparation of Monosubstituted Azepane

N,N-Diethyl-3*H*-azepin-2-amine (S18)

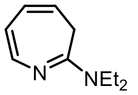

Following **GP1a**, nitrobenzene (1.0 mL, 10 mmol) gave **S18** (618 mg, 38%) as an oil. ^{1}H NMR (500 MHz, $CDCl_3$) δ 7.11 (1H, d, *J* = 7.9 Hz), 6.31 (1H, dd, *J* = 8.7, 5.6 Hz), 5.79–5.68 (1H, m), 5.10 (1H, q, *J* = 7.5 Hz), 3.37 (4H, q, *J* = 7.0 Hz), 2.66 (2H, br s), 1.14 (6H, t, *J* = 6.9 Hz); ^{13}C NMR (126 MHz, $CDCl_3$) δ 144.8, 140.9, 128.7, 112.0, 109.8, 43.2, 31.5, 13.5. Data in accordance with the literature.[26]

2-(4-Methoxyphenyl)-3*H*-azepine (S19)

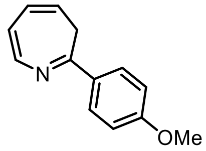

Following **GP3**, **S18** (300 mg, 1.8 mmol) and freshly prepared (4-methoxyphenyl)lithium (0.8 M in THF, 4.0 mL, 3.1 mmol), gave **S19** (69%) as a solid. R_f 0.56 [pentane:Et_2O (9:1)]; ^{1}H NMR (600 MHz, $CDCl_3$) δ 7.85 (2H, d, *J*=8.4 Hz), 7.55 (1H, d, *J*=7.9 Hz), 6.91 (2H, d, *J*=8.4 Hz), 6.42 (1H, t, *J*=7.1 Hz), 6.21 (1H, d, *J*=7.3 Hz), 5.28 (1H, q, *J*=7.3 Hz), 3.84 (3H, s), 2,88 (2H,br s); ^{13}C NMR (151 MHz, $CDCl_3$) δ 161.1, 143.7, 141.4, 130.6, 130.2, 127.8, 116.3, 116.2, 114.0, 55.5, 34.1; HRMS (EI): found M^+ 199.0990, $C_{13}H_{13}ON$ requires 199.0992.

tert-Butyl *(R)*-2-(4-methoxyphenyl)azepane-1-carboxylate (S19a)

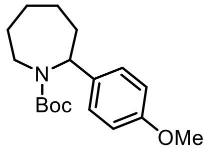

Following **GP6**, **S19** (20 mg, 0.1 mmol), gave **S19a** (quant., 56:44 er) as an oil. R_f 0.47 [hexane:EtOAc (9:1)]; HPLC analysis of **S19a**: Chiralcel® OD-H, hexane: *i*–PrOH 99:1, flow 1.0 mL/min, oven temperature 30 °C, t_r = 6.44 min and t_r = 7.79 min; ^{1}H NMR (600 MHz, $CDCl_3$, rotamers) δ 7.17 (1H, d, *J* = 8.4 Hz), 7.11 (1H, d, *J* = 8.6 Hz), 6.83 (2H, m), 5.15 (0.5H, dd, *J* = 11.9, 6.7 Hz), 4.88 (0.5H, dd, *J* = 12.1, 5.9 Hz), 4.07 (0.5H, d, *J* = 14.4 Hz), 3.83 (0.5H, m), 3.79 (1.5H, s), 3.79 (1.5H, s), 2.94 (0.5H, t, *J* = 12.4 Hz), 2.86 (0.5H, t, *J* = 12.9 Hz), 2.39–2.29 (0.5H, m), 2.28–2.20 (0.5H, m), 1.96–1.82 (2H, m), 1.81–1.58 (3H, m), 1.47 (4.5H, s), 1.41 (2H, m), 1.33 (4.5H, s); ^{13}C NMR (151 MHz, $CDCl_3$, rotamers) δ 158.4, 158.3, 156.2, 156.0, 137.0, 136.0, 127.1, 126.8, 113.9, 113.7, 79.5, 79.3, 59.7, 57.7, 55.4, 43.2, 43.0, 36.5, 35.5, 29.9, 29.8 29.7, 29.5, 28.7, 28.5, 26.4, 25.6; HRMS (ESI): found MNa^+ 328.1887, $C_{18}H_{27}NO_3Na$ requires 328.1883.

8 Reproducibility

We tested if oxidation of the phopshoramidite **L2** could be an issue for reproducibility. We therefore synthetized **Ox-L2** and tested its effect in the reaction conditions. The presence of **Ox-L2** proved to decrease yield, er and dr (**Table S13**). To guarantee the reproducibility of each reaction, all phosphoramidites were systematically synthesized following **GP4** or repurified.

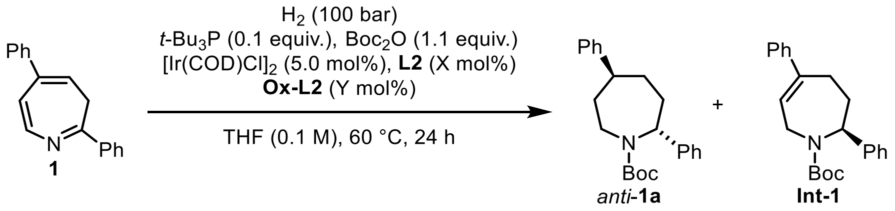

Table S13. Ligand Poisoning.

entry	X/Y (mol%)	*anti*-1a			Int-1		rsm 1 (%)
		yield (%)	er	dr	yield (%)	er	
**1**	20/0	39	95:5	>20:1	47	80:20	–
**2**	10/10	42	87:13	5:1	31	46:54	–
**3**	0/20	20	50:50	2:3	5	n.d.	–

The quality of the iridium metal catalyst has a huge impact on the outcome of the reaction (**Table S14**). $[Ir(COD)Cl]_2$ was constantly purchased from supplier–3 (Thermo-Fisher).

Table S14. [Ir] Suppliers.

entry	Ir	Batch 1		Batch 2	
		anti-1a		*anti*-1a	
		yield (%)	er	yield (%)	er
**1**	supplier–1	–	–	–	–
**2**	supplier–2	56	85:15	14	96:4
**3**	supplier–3	41	95:5	39	95:5

Supplier–1 Supplier–2 Supplier–3

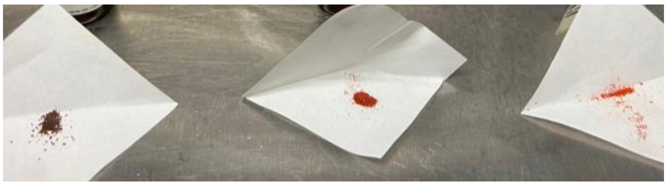

9 Mechanistic Experiments

9.1 Time-profile

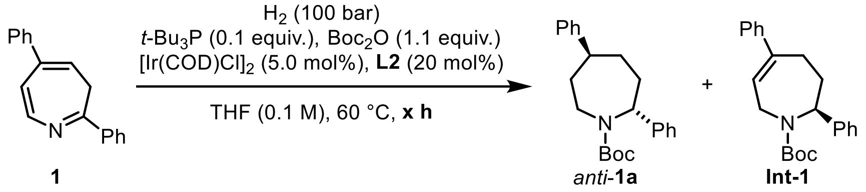

Table S15. Reaction Time Screening with *t*-Bu_3P.

entry	time (h)	*anti*-1a			Int-1		1 (rsm) (%)
		yield (%)	er	dr	yield (%)	er	
**1**	0	–	–	–	–	–	100
**2**	0.5	–	–	–	<2	45:55	97
**3**	1	–	–	–	14	45:55	81
**4**	1.5	–	–	–	20	45:55	75
**5**	2	–	–	–	32	45:55	61
**6**	4	–	–	–	48	45:55	46
**7**	6	14	>99:1	>20:1	57	56:44	15
**8**	8	18	97:3	>20:1	58	59:41	12
**9**	12	26	96:4	>20:1	60	64:36	–
**10**	16	30	95:5	>20:1	52	70:30	–
**11**	24	39	95:5	>20:1	47	80:20	–
**12**	48	68	81:19	8.5:1	19	>99:1	–

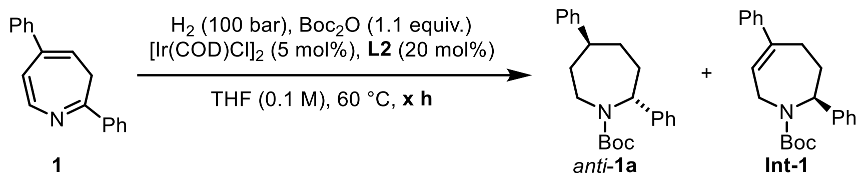

Table S16. Reaction Time Screening without *t*-Bu_3P.

entry	Time (h)	*anti*-1a			Int-1		1 (rsm) (%)
		yield (%)	er	dr	yield (%)	er	
**1**	0	–	–	–	–	–	100
**2**	0.5	–	–	–	8	46:54	88
**3**	1	–	–	–	30	46:54	56
**4**	1.5	–	–	–	36	45:55	50
**5**	2	–	–	–	50	45:55	29
**6**	4	<2	–	–	85	45:55	–
**7**	6	25	62:38	4:1	58	65:35	–
**8**	8	32	67:33	4:1	50	59:41	–
**9**	16	56	60:40	8:1	21	67:33	–
**10**	24	65	55:45	6:1	16	71:29	–

9.2 Determination of the S–factor

The S factor was determined using the following formula:[27]

$$S = \frac{\ln\,[(1-c)(1-ee_{\text{Int-1}})]}{\ln\,[(1-c)(1+ee_{\text{Int-1}})]}$$

where c is the conversion determined using the enantiomeric excess of recovered **Int-1** ($ee_{\text{Int-1}}$) and **1a** ($ee_{anti\text{-}1a}$) following the formula:

$$c = \frac{ee_{\text{Int-1}}}{ee_{\text{Int-1}}+ee_{anti\text{-}1a}}$$

In all cases, *syn*-**1a** is formed in very negligeable amount.

S–Factor in presence of *t*-Bu$_3$P

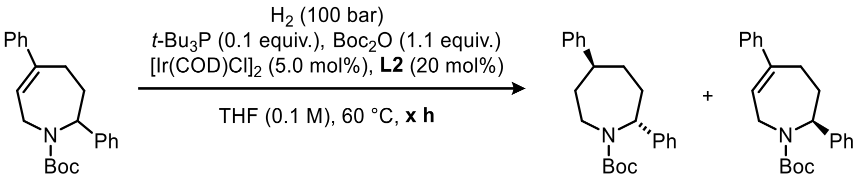

rac-**Int-1** *anti*-**1a** **Int-1**

Table S17. Determination of the S–factor in presence of *t*-Bu$_3$P.

entry	time (h)	*anti*-1a		Int-1 (rsm)		c	ln[(1-c)(1-ee$_{\text{Int-1}}$)]	ln[(1-c)(1+ee$_{\text{Int-1}}$)]
		yield	er	yield	er			
**1**	1	20	87:13	79	59:41	0.20	-0.416174423	-0.052209045
**2**	2	33	88:12	73	66:34	0.30	-0.737060368	-0.07376615
**3**	4	52	86:14	38	93:7	0.51	-2.14766251	-0.155232346

entry	time (h)	*anti*-1a		Int-1 (rsm)		c	ln[(1-c)(1-ee$_{Int-1}$)]	ln[(1-c)(1+ee$_{Int-1}$)]
		yield	er	yield	er			
**4**	6	55	87:13	39	93:7	0.54	-2.737221578	-0.150532234
**5**	8	59	76:24	21	>99:1	0.66	-7.285928245	-0.379173466
**6**	16	60	77:23	17	>99:1	0.65	-7.261277109	-0.35452233

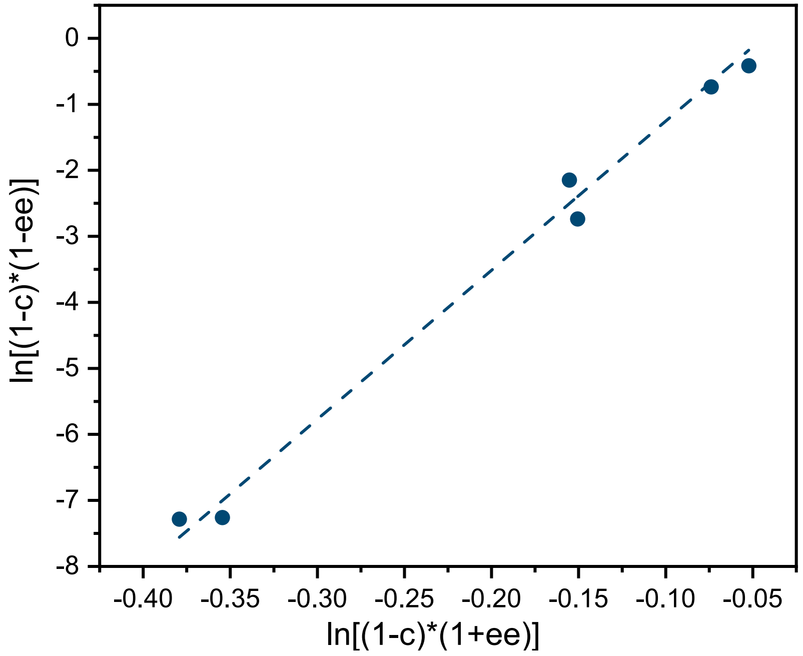

Figure S6. Linear regression analysis to determine S–factor in presence of *t*-Bu_3P. From the linear regression analysis: intercept = 0.8498, slope = S–factor = 22.038, R^2 = 0.9916.

S–Factor in absence of *t*-Bu$_3$P

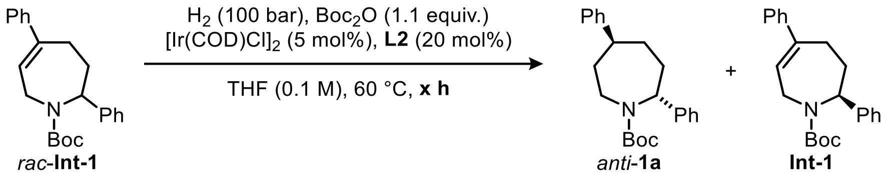

Table S18. Determination of the S–factor without *t*-Bu$_3$P.

entry	time (h)	*anti*-1a		Int-1 (rsm)		c	ln[(1-c)(1-ee$_{Int-1}$)]	ln[(1-c)(1+ee$_{Int-1}$)]
		yield	er	yield	er			
**1**	0.3	12	49:51	85	47:53	0,75	-1.448169765	-1.328025453
**2**	1	16	52:48	64	46:54	0,67	-1.181993898	-1.021651248
**3**	2	18	49:51	68	46:54	0,80	-1.692819521	-1.532476871
**4**	6	56	56.44	37	57:43	0,58	-1.026291627	-0.744440475

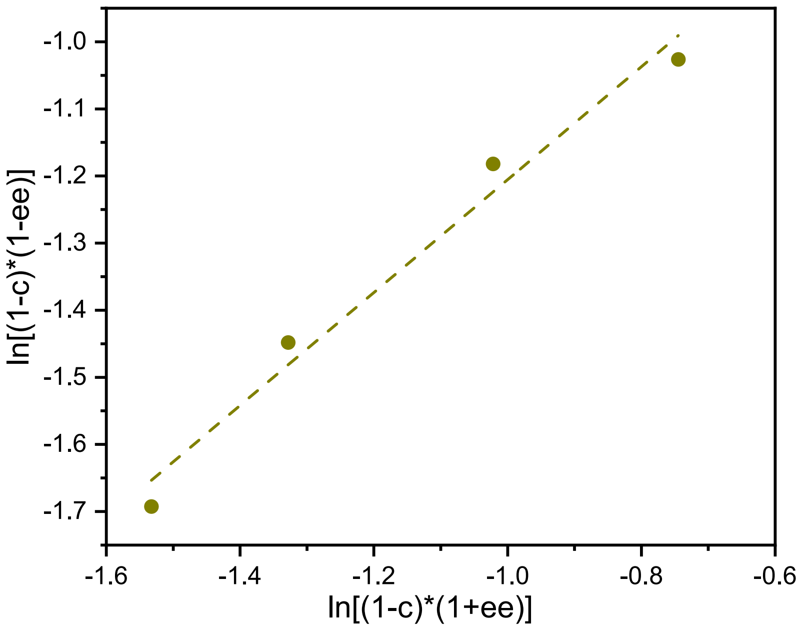

Figure S7. Linear regression analysis to determine S–factor in absence of *t*-Bu_3P. From the linear regression analysis: intercept = 0.3644, slope = S–factor = 0.8421, R^2 = 0.9781.

9.3 Impact of the [Ir]/L2/*t*-Bu$_3$P Ratio on Reactivity and Selectivity

To test the impact of the phosphine additive we screened different Ir/**L2**/*t*-Bu$_3$P ratios with an Ir/**L2**/*t*-Bu$_3$P stoichiometry = 1/2/1 being optimal (**Table S19**). However, this does not necessarily reflect the nature of the active catalyst but it is the best experimental conditions we have identified to maximize yield and ee.

Table S19. Screening of Ir/**L2**/*t*-Bu$_3$P Ratio.

entry	[Ir]/L2/ *t*-Bu$_3$P (mol%)	*anti*-1a			Int-1		rsm 1 (%)
		yield (%)	er	dr	yield (%)	er	
**1**	10/20/10	39	95:5	>20:1	47	80:20	–
**2**	10/10/10	56	87:13	>20:1	34	55:45	–
**3**	10/10/20	50	91:9	>20:1	40	75:25	–
**4**	10/20/5	63	88:12	>20:1	18	95:5	–
**5**	10/20/20	40	96:4	>20:1	55	62:38	–
**6**	10/20/50	28	97:3	>15:1	61	55:45	–
**7**	10/20/100	12	97:3	>15:1	76	45:55	–
**8**	10/0/20	–	–	–	7	50:50	80
**9**	10/20/0	60	54:46	6:1	27	71:29	–
**10**	10/0/0	–	–	–	–	–	100

We propose that there are three complexes which might be in equilibrium: one hetero-complex (**A**) and two homo-complexes (**B** and **C**) (**Figure S8**).

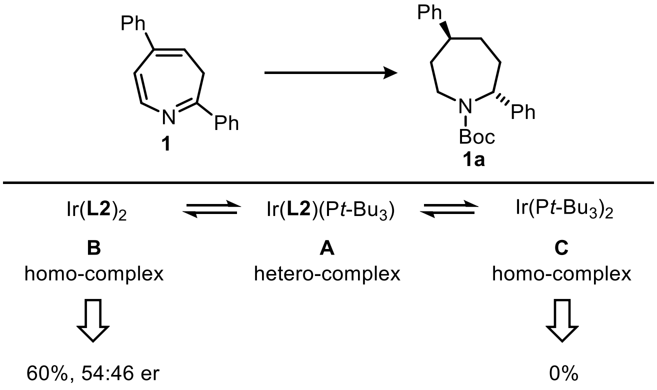

Figure S8

The homo-complex with only *t*-Bu$_3$P (**C**) is completely inactive (Table S19, entry 8) while the homo complex with two **L2** (**B**) provides the desired product in good yield but very low er (Table S19, entry 9). Based on previous literature on mixed ligands approach using achiral phosphine in combination to

phosphoramidite, we believe the active catalyst to be the 1/1/1 Ir/**L2**/*t*-Bu_3P complex.[28–31] However, as phosphines are stronger donor ligands than phosphonamidites, an excess of phosphoramite is required to shift the equilibrium toward the formation of the heterocomplex (**A**). A larger excess of **L2** compared to *t*-Bu_3P however will favour the formation of heterocomplex (**B**) leading to higher yield but decreased er (entries 2,4), while a larger excess of *t*-Bu_3P or a ratio of 1:1 L2: *t*-Bu_3P will favour the heterocomplex (**C**), leading to decreased yield but higher er (entries 5, 6 and 7). Globally, it has been observed that a ratio of **L2**: *t*-Bu_3P ~ 2:1 is necessary to maximize both yield and er.

9.4 ^{31}P NMR Studies of the Ir/L2/*t*-Bu$_3$P Complexes

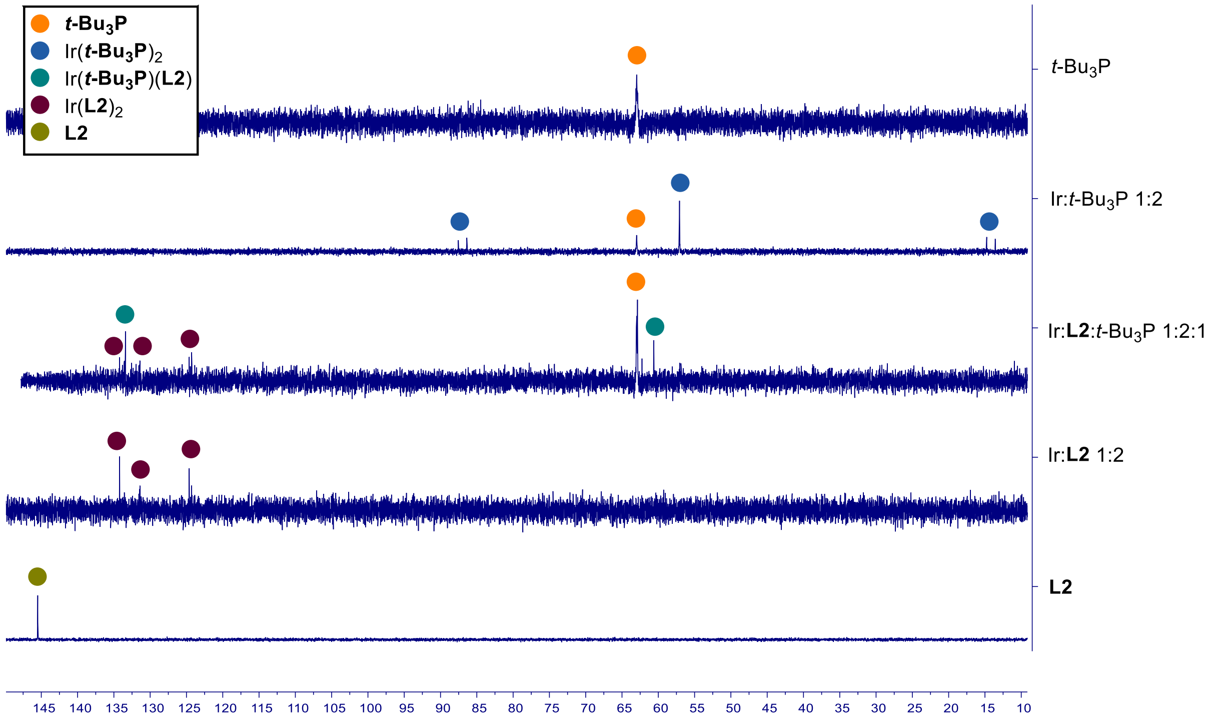

Figure S9. ^{31}P{^{1}H} NMR of different ratios of Ir(COD)$_2$BARF (Ir), *t*-Bu$_3$P and **L2** in THF-d$_8$.

10 Computational Studies

10.1 Computational Details

A comprehensive conformational analysis was carried out for **Int-1-4** to identify the lowest-energy conformers and establish a reliable energetic ordering among the accessible structures. Although these compounds share a common molecular scaffold, they differ in their protecting groups, offering an opportunity to evaluate how such substituents influence the overall geometry and conformational preferences. Our aim was to examine whether these variations primarily affect the steric profile, torsional flexibility, or intramolecular interactions of the scaffold, with the ultimate goal of predicting the most likely region of the molecule for coordination to the iridium metal center.

The conformational search was initially carried out using the GOAT (Global Optimization of Algorithmic Torsions)[32,33] module as implemented in the ORCA 6.0.1 program package.[34–36] In this automated approach, the torsional degrees of freedom are systematically sampled in order to generate a broad and representative set of conformers distributed over the potential energy surface. The search was performed employing the GOAT-ENTROPY keyword. For computational efficiency, the underlying electronic structure calculations were conducted at the xTB (extended tight-binding) level,[37] which allows for a reliable yet rapid evaluation of the potential energy hypersurface during the automated search. This combination ensures a comprehensive and efficient identification of relevant conformers for further high-level quantum chemical investigations.

For this conformational ensemble, all unique structures lying within 3.0 kcal mol^{-1} relative to the global minimum were selected for further refinement. These conformers were subsequently reoptimized at the Density Functional Theory (DFT)[38–41] level using the long-range corrected ωB97XD functional[42] in combination with the correlation consistent cc-pVDZ basis set,[43–47] as implemented in Gaussian 16, Revision C.02 program package.[48] The same level of theory was employed for vibrational frequency analyses to confirm that no negative eigenvalues in the Hessian matrix. No symmetry restrictions were applied. Single-point energy calculations with the cc-pVTZ basis set[43–47] were conducted on the DFT-optimized geometries to refine the energetic ordering, employing the ωB97XD functional.

Thermodynamic data were obtained via quasi-harmonic corrections to entropy using the rigid rotor/harmonic oscillator (RRHO) approximation,[49] in which vibrational modes below 100 cm^{-1} were treated with a free rotor approximation interpolated with a damping function. These corrections were applied with the *Goodvibes.py* script.[50]

Finally, the relative energies of the conformers were calculated on the cc-pVTZ single-point results, and these values were used to establish the energetic hierarchy of the conformational landscape. The lowest-energy structures and their corresponding relative energies are summarized in Table **S20-S23.**

In addition to the conformational study described above, we also investigated larger catalytic systems comprising the Ir catalyst, the chiral ligand (**L2**) in a combination with an achiral phosphine ligand (*t*-

Bu_3P), together with hydrogen and the substrate ((*R*)-**Int-1**). These model systems were constructed based on reported literature precedents to ensure structural relevance. The geometry optimizations of these complexes were performed at the density functional theory (DFT) level using the Gaussian 16, Revision C.02 program package. The long-range corrected ωB97XD functional was employed throughout, as it incorporates empirical dispersion corrections that are essential for accurately describing weak noncovalent interactions within the catalyst-substrate assembly.

For the geometry optimizations, a mixed basis set was applied: 6-31G(d,p)[51–60] for **H**, **C**, **N**, **O** and **P** atoms, while the LANL2DZ[61–64] effective core potential and associated basis set were used for **Ir**. To improve the description of the metal center, an additional f-type polarization function (exponent 0.938)[65] was included. The same level of theory was employed for vibrational frequency analyses to confirm that no negative eigenvalues in the Hessian matrix. No symmetry restrictions were applied. Subsequent single-point energy refinements were carried out on the optimized geometries employing larger basis sets: def2-TZVP[66] for **H**, **C**, **N**, **O** and **P** atoms, and def2-QZVP[66] basis set for **Ir**. To account for solvation effects, the SMD implicit solvent model[67] was applied with THF as the solvent.

Thermodynamic data were obtained via quasi-harmonic corrections to entropy using the rigid rotor/harmonic oscillator (RRHO) approximation, in which vibrational modes below 100 cm^{-1} were treated with a free rotor approximation interpolated with a damping function. These corrections were applied with the *Goodvibes.py* script, assuming a solution-phase standard state (c = 1 mol^{-1}).

Through this computational setup, our objective was to identify which catalyst-substrate assembly is energetically the most favourable and, consequently, to predict the stereochemical outcome of the hydrogenation reaction. Specifically, the analysis aims to determine whether the reaction pathway preferentially leads to the (2*R*,5*S*) or the (2*R*,5*R*) product.

10.2 Conformational Analysis

Conformational preferences play a decisive role in determining the steric environment, torsional flexibility, and intramolecular interactions of organic molecules, all of which directly impact their reactivity and selectivity. In particular, for systems incorporating seven membered rings, subtle conformational changes can significantly influence the degree of facial differentiation, thereby affecting the stereochemical outcome of catalytic transformations. In this study, a detailed conformational analysis of the investigated intermediates (*S*)**-Int-1-4** (**Figure S10**) was undertaken in order to identify their lowest-energy structures and to evaluate how substituent effects shape the conformational landscape. These insights are especially critical for rationalizing the extent to which conformational adjustment may reduce facial bias and thus limit the achievable diastereoselectivity. The optimized geometries and relative energies of these intermediates are summarized below (**Table S20-S23**).

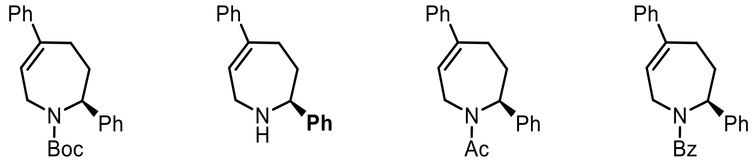

(*S*)-Int-1 **(*S*)-Int-2** **(*S*)-Int-3** **(*S*)-Int-4**

Figure S10. Structures of the investigated intermediates **(*S*)-Int-1-4**.

Table S20. Optimized geometries and relative energies of the lowest-energy conformers of **(*S*)-Int-1**. The blue values correspond to relative energies obtained from the initial GOAT conformational search (xTB level), whereas the red values represent the corresponding Gibbs free energies calculated at UωB97XD/cc-pVTZ//cc-pVDZ level of theory.

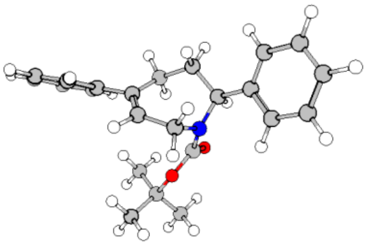<br>**Conf-1**<br>**0.0**<br>**0.0**	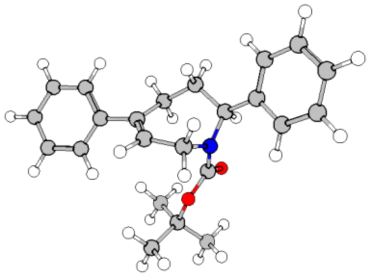<br>Conf-2<br>0.2<br>0.4	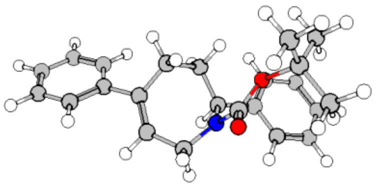<br>Conf-3<br>0.5<br>1.5

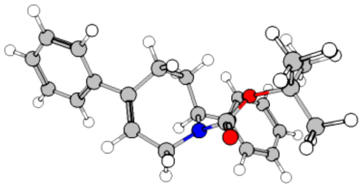<br>Conf-4<br>0.7<br>1.8	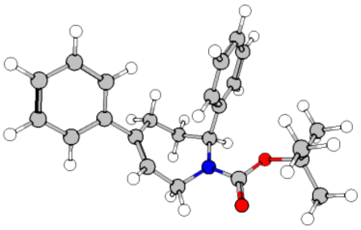<br>Conf-5<br>1.0<br>0.5	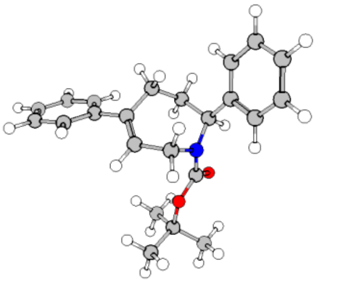<br>Conf-6<br>1.1<br>1.3
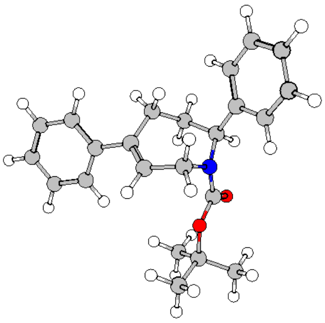<br>Conf-7<br>1.1<br>1.2	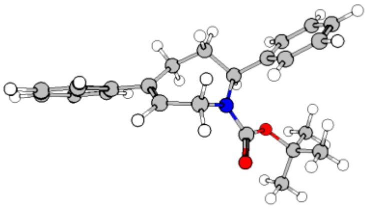<br>Conf-8<br>1.2<br>0.5	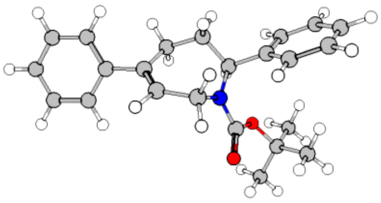<br>Conf-9<br>1.3<br>0.0
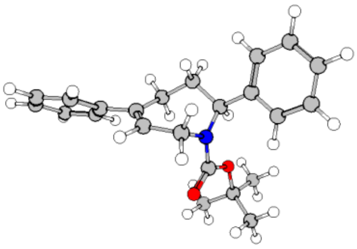<br>Conf-10<br>1.4<br>0.4	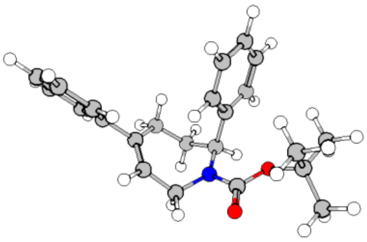<br>Conf-11<br>1.4<br>0.8	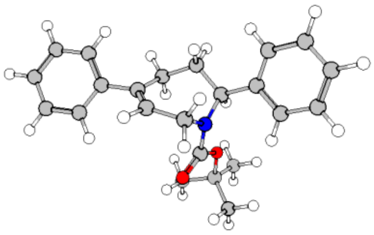<br>Conf-12<br>1.4<br>1.0

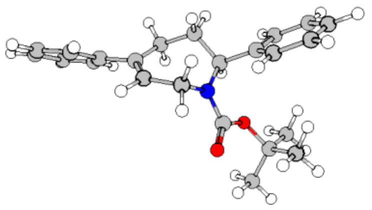<br>Conf-13<br>1.5<br>0.5	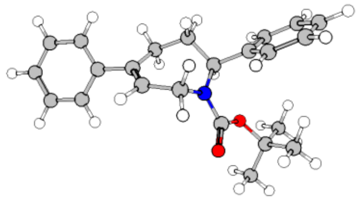<br>Conf-14<br>1.6<br>0.0	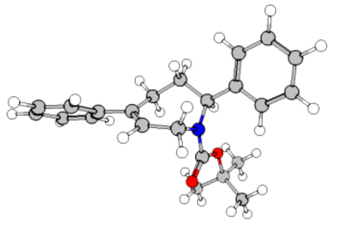<br>Conf-15<br>1.8<br>0.4
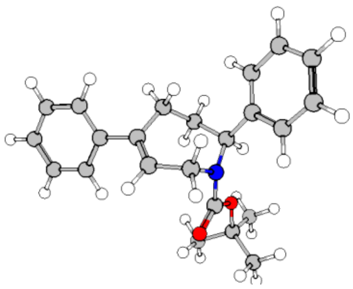<br>Conf-16<br>2.0<br>2.2	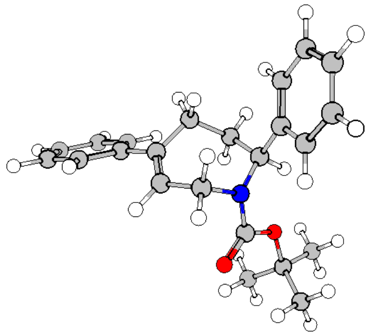<br>Conf-17<br>2.0<br>2.2	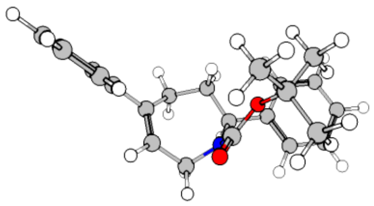<br>Conf-18<br>2.1<br>3.3
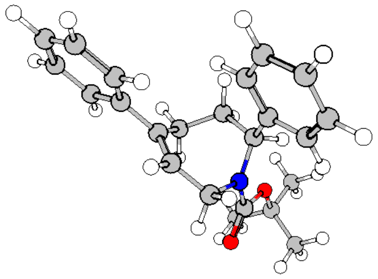<br>Conf-19<br>2.1<br>3.3	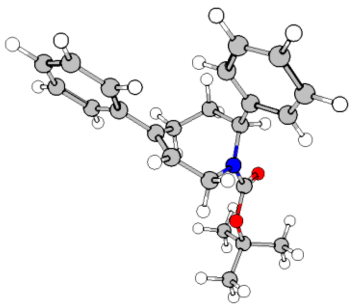<br>Conf-20<br>2.1<br>3.1	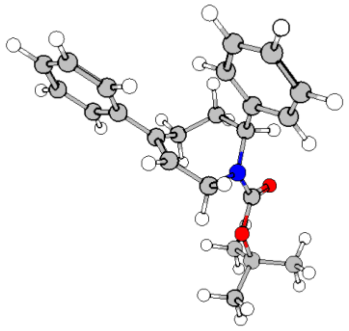<br>Conf-21<br>2.1<br>3.1

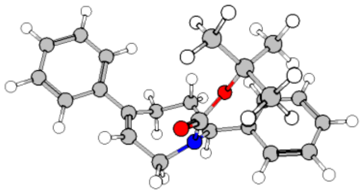 Conf-22<br>2.2<br>3.6	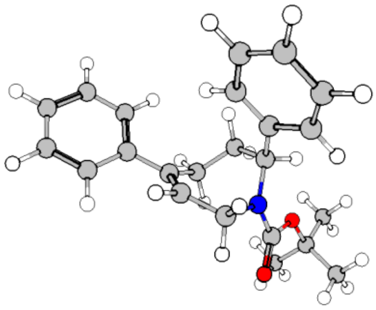 Conf-23<br>2.3<br>3.1	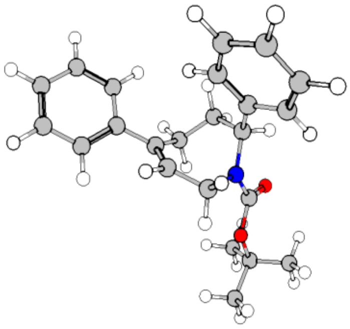 Conf-24<br>2.4<br>3.0
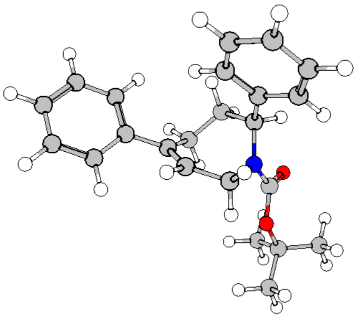 Conf-25<br>2.4<br>3.0	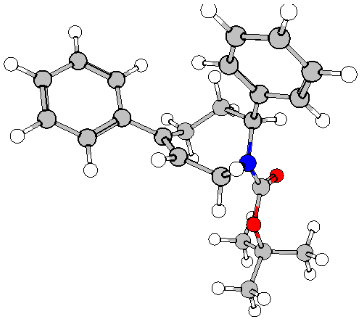 Conf-26<br>2.4<br>3.0	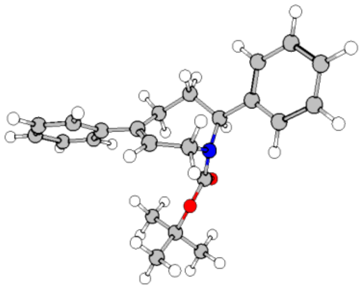 Conf-27<br>2.5<br>0.0
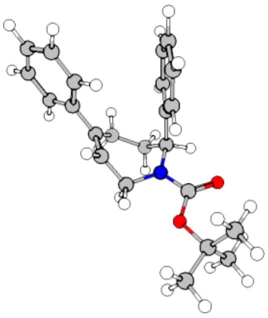 Conf-28<br>2.7<br>4.2	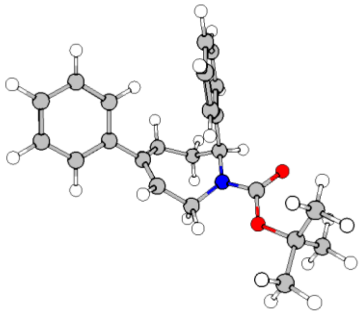 Conf-29<br>2.9<br>3.2	

Table S21. Optimized geometries and relative energies of the lowest-energy conformers of ***(S)*-Int-2**. The blue values correspond to relative energies obtained from the initial GOAT conformational search (xTB level), whereas the red values represent the corresponding Gibbs free energies calculated at UωB97XD/cc-pVTZ//cc-pVDZ level of theory.

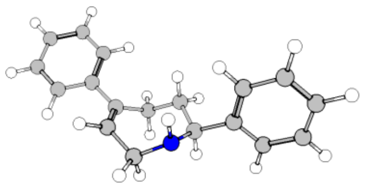<br>Conf-1<br>0.0<br>1.2	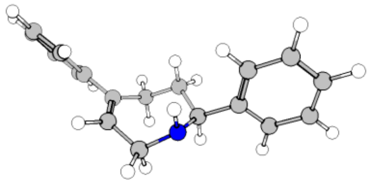<br>Conf-2<br>0.2<br>1.2	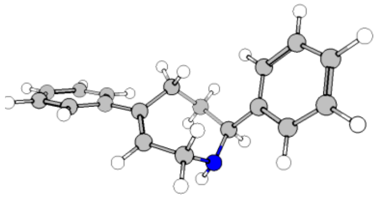<br>Conf-3<br>1.3<br>2.5
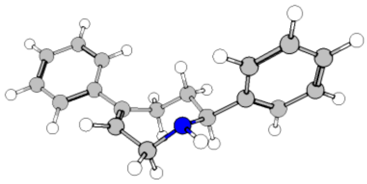<br>**Conf-4**<br>**1.3**<br>**0.0**	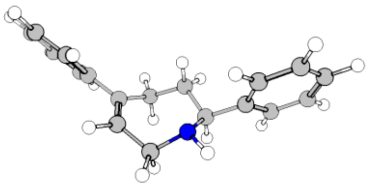<br>Conf-5<br>1.4<br>0.2	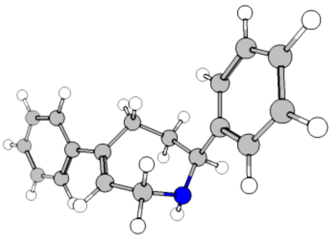<br>Conf-6<br>1.4<br>2.4
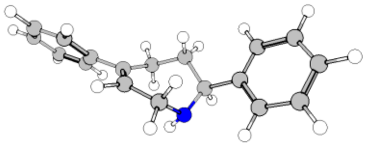<br>Conf-7<br>1.7<br>1.5	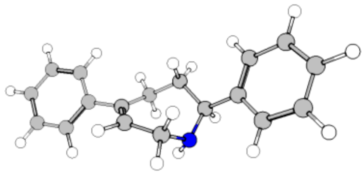<br>Conf-8<br>2.0<br>1.5	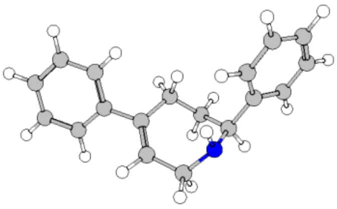<br>Conf-9<br>2.2<br>2.6
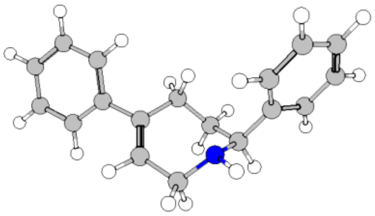<br>Conf-10<br>2.2<br>0.4	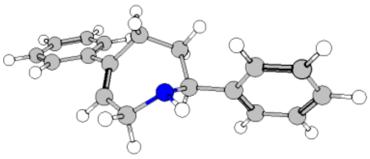<br>Conf-11<br>2.3<br>2.2	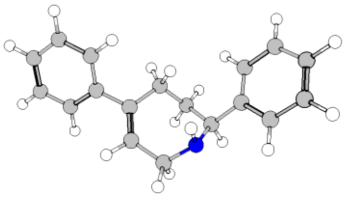<br>Conf-12<br>2.4<br>2.1

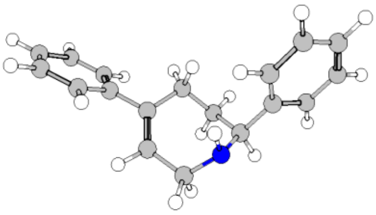<br>Conf-13<br>2.4<br>2.5	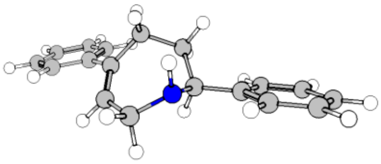<br>Conf-14<br>2.4<br>3.0	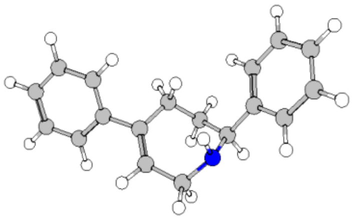<br>Conf-15<br>2.6<br>2.1
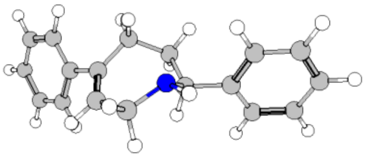<br>Conf-16<br>2.6<br>2.4	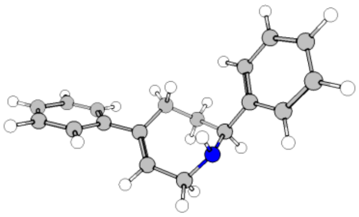<br>Conf-17<br>2.6<br>2.3	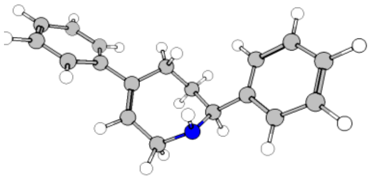<br>Conf-18<br>2.6<br>2.3
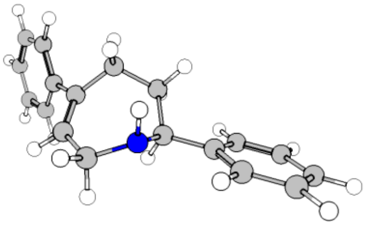<br>Conf-19<br>2.7<br>3.2	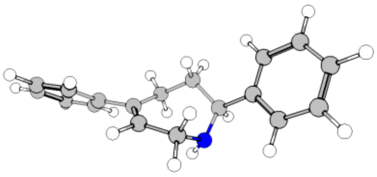<br>Conf-20<br>2.9<br>1.5	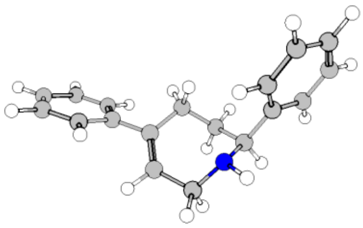<br>Conf-21<br>3.0<br>0.8

Table S22. Optimized geometries and relative energies of the lowest-energy conformers of ***(S)*-Int-3**. The blue values correspond to relative energies obtained from the initial GOAT conformational search (xTB level), whereas the red values represent the corresponding Gibbs free energies calculated at UωB97XD/cc-pVTZ//cc-pVDZ level of theory.

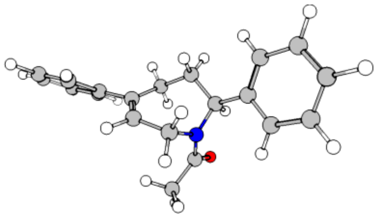<br>**Conf-1**<br>**0.0**<br>**0.0**	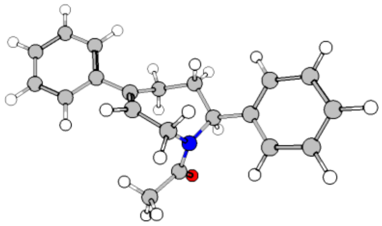<br>Conf-2<br>0.3<br>0.3	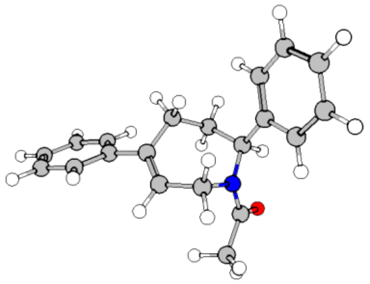<br>Conf-3<br>1.1<br>2.0
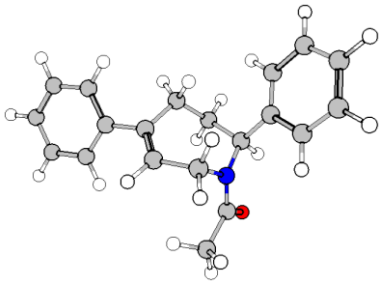<br>Conf-4<br>1.2<br>2.0	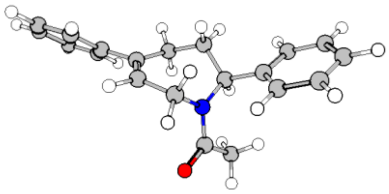<br>Conf-5<br>1.8<br>0.6	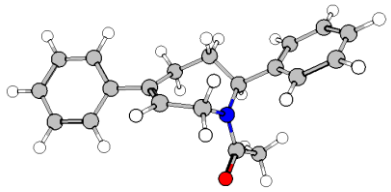<br>Conf-6<br>1.9<br>0.3
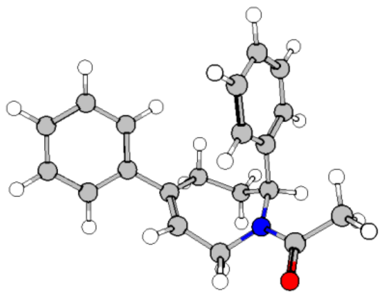<br>Conf-7<br>2.3<br>1.6	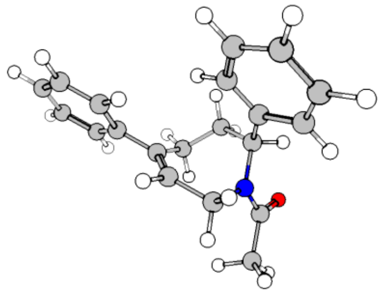<br>Conf-8<br>2.3<br>3.0	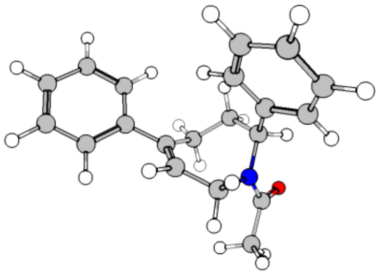<br>Conf-9<br>2.6<br>3.2

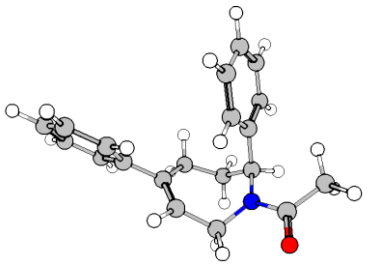<br>Conf-10<br>2.6<br>2.5	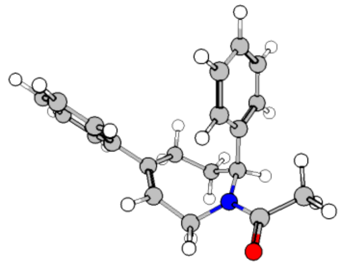<br>Conf-11<br>2.6<br>2.5	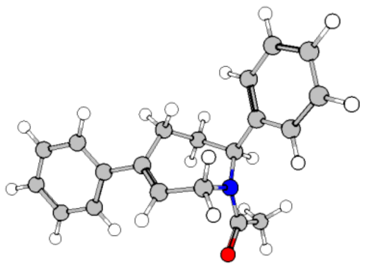<br>Conf-12<br>2.7<br>2.4
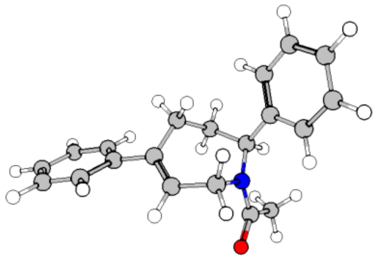<br>Conf-13<br>2.8<br>2.6	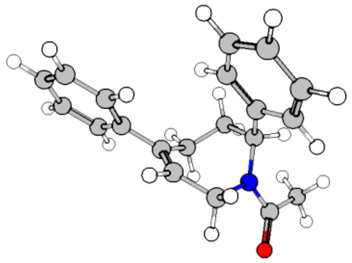<br>Conf-14<br>2.9<br>3.9	

Table S23. Optimized geometries and relative energies of the lowest-energy conformers of ***(S)*-Int-4**. The blue values correspond to relative energies obtained from the initial GOAT conformational search (xTB level), whereas the red values represent the corresponding Gibbs free energies calculated at UωB97XD/cc-pVTZ//cc-pVDZ level of theory.

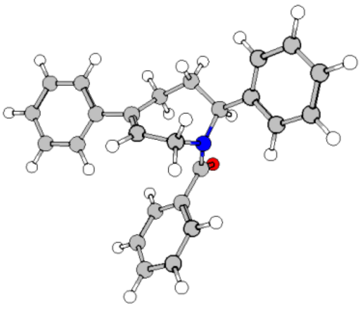<br>Conf-1<br>0.0<br>1.0	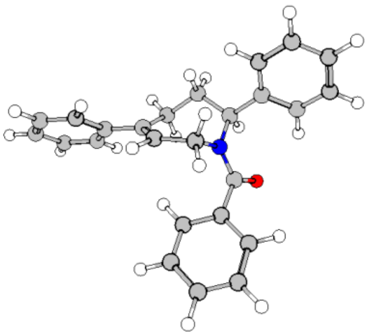<br>Conf-2<br>0.0<br>0.3	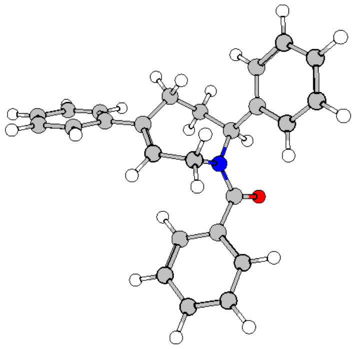<br>Conf-3<br>0.9<br>2.4
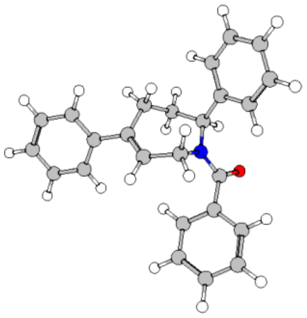<br>Conf-4<br>1.0<br>2.5	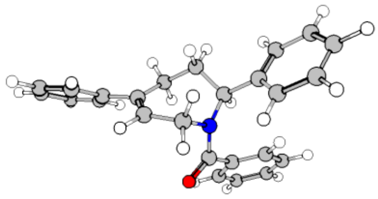<br>Conf-5<br>1.4<br>0.5	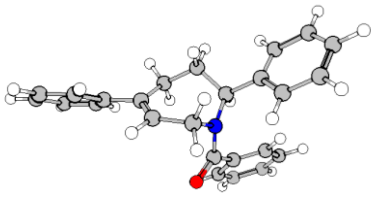<br>Conf-6<br>1.4<br>0.5
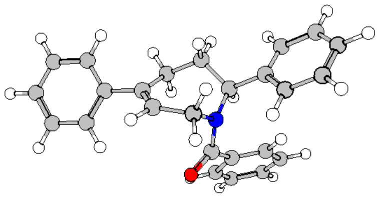<br>**Conf-7**<br>**1.4**<br>**0.0**	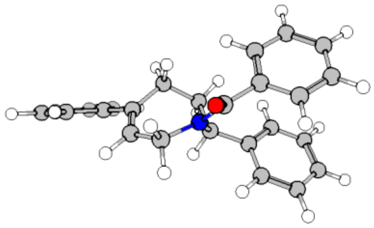<br>Conf-8<br>1.5<br>4.3	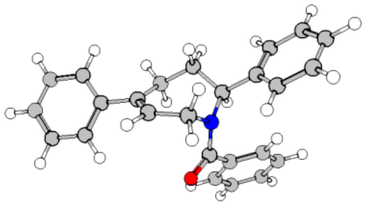<br>Conf-9<br>1.6<br>0.0

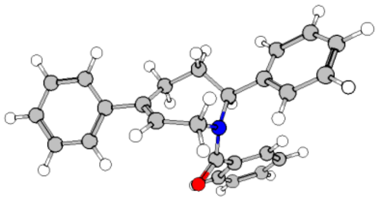<br>Conf-10<br>1.6<br>0.0	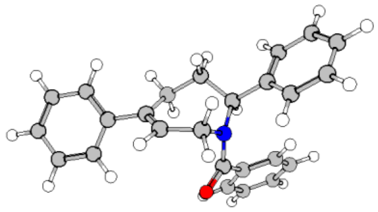<br>Conf-11<br>1.6<br>0.0	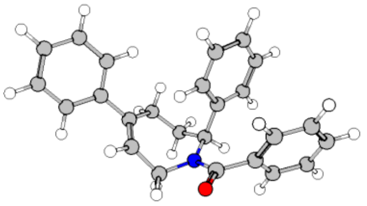<br>Conf-12<br>1.7<br>2.5
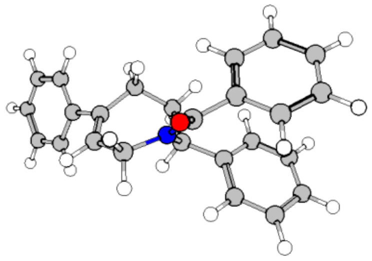<br>Conf-13<br>1.8<br>4.3	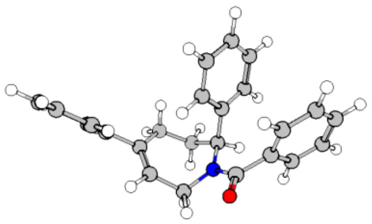<br>Conf-14<br>2.1<br>3.0	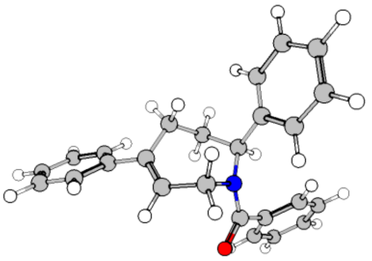<br>Conf-15<br>2.3<br>2.7
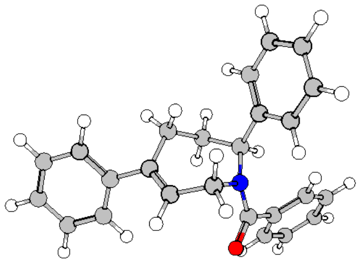<br>Conf-16<br>2.4<br>2.4	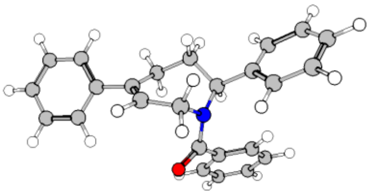<br>Conf-17<br>2.5<br>0.0	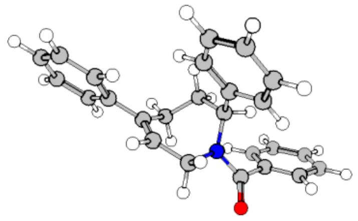<br>Conf-18<br>2.5<br>3.6
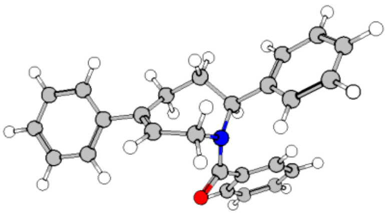<br>Conf-19<br>2.5<br>0.0	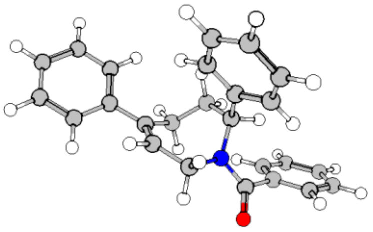<br>Conf-20<br>2.6<br>3.7	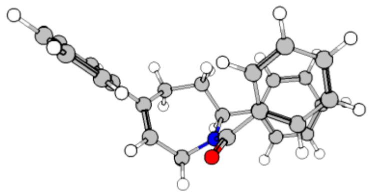<br>Conf-21<br>2.6<br>5.4

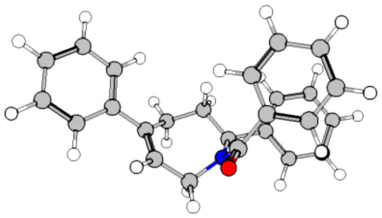	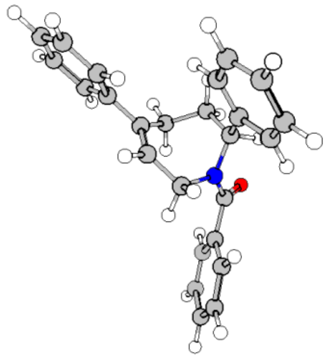	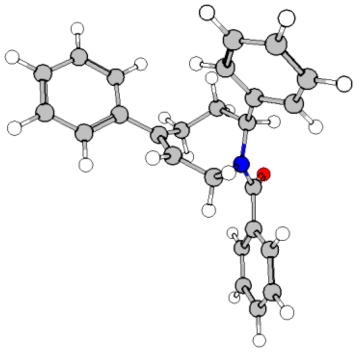
Conf-22	Conf-23	Conf-24
2.7	2.8	3.0
5.7	3.2	3.3

The conformational analysis of intermediates (*S*)-**Int-1-4**, bearing Boc, H, Ac, or Bz as a substituent on the nitrogen atom, highlights how the protecting group governs the steric environment of the seven-membered ring (**Table S24**). In the lowest-energy conformations, the bulky Boc group in (*S*)-**Int-1** imposes significant steric interactions that enforce a well-defined spatial arrangement, thereby maintaining pronounced facial differentiation. By comparison, the Bz group (**Int-4**) also generates a pronounced steric clash with the adjacent substituents, yet the resulting facial differentiation is less effective than that enforced by Boc. Replacement of Boc/Bz with the smaller Ac group (**Int-3**) alleviates this steric congestion, creating a more balanced environment that reduces the distinction between the two faces of the ring. Finally, the absence of any protecting group (**Int-2**, NH) yields the most conformationally flexible structure, in which steric interactions are minimized, and facial bias is largely lost. This trend underscores how subtle conformational changes induced by the protecting group can strongly influence facial differentiation and, ultimately, the stereochemical outcome of the system.

	→ increase of steric clash →			
protecting group	H (**Int-2**)	Ac (**Int-3**)	Bz (**Int-4**)	Boc (**Int-1**)
dr *anti*:*syn*	1:7	8:1	10.3:1	> 20:1

Table S24. Comparison of protecting groups in intermediates (*S*)-**Int-1-4**. Shown are the molecular structures and their corresponding lowest-energy geometries optimized at the DFT (UωB97XD/cc-pVDZ) level of theory.

Protecting Group	Structure	Optimized Geometry
**Boc**	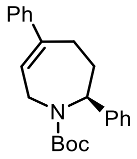 **(*S*)-Int-1**	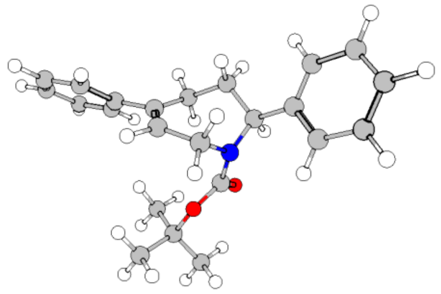
**H**	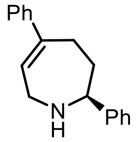 **(*S*)-Int-2**	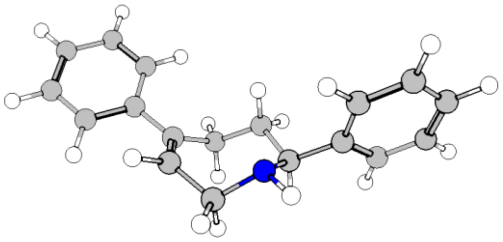
**Ac**	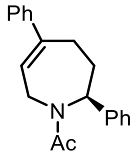 **(*S*)-Int-3**	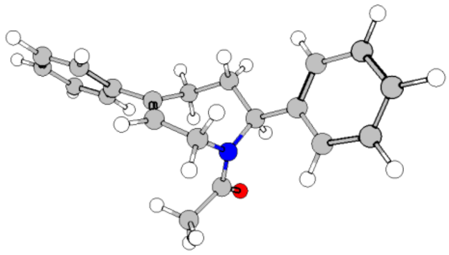
**Bz**	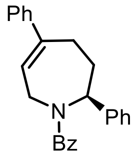 **(*S*)-Int-4**	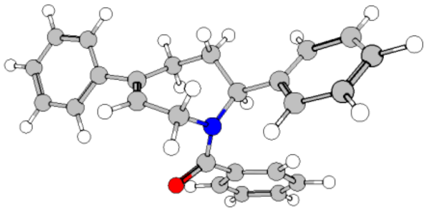

10.3 Mechanistic Studies

To gain deeper insight into the origin of diastereoselectivity in the Ir-catalyzed process, we next turned our attention to the mechanistic investigation. Based on precedents from the literature,[68] three possible mechanistic pathways (**Figure S11**) were identified and used as the basis for our computational analysis. For each mechanism, the corresponding reactant structures were constructed starting from (*R*)-**Int-1**, which is the precursor of product (*R,S*)-**1a** obtained in the reaction conditions. In the case of systems lacking a *t*-Bu_3P ligand, the equatorial coordination sphere of Ir was modelled with two **L2**, whereas in the *t*-Bu_3P-containing cases, one **L2** and one *t*-Bu_3P ligand were placed equatorially. We note that this ligand arrangement is partly speculative but was chosen to provide a reasonable starting point for comparison.

Proposed Mechanism 1

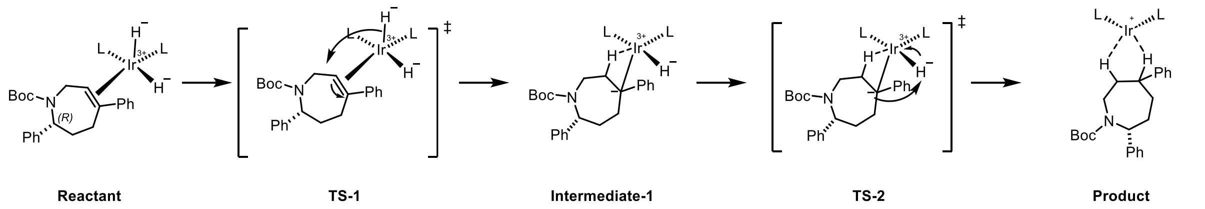

Proposed Mechanism 2

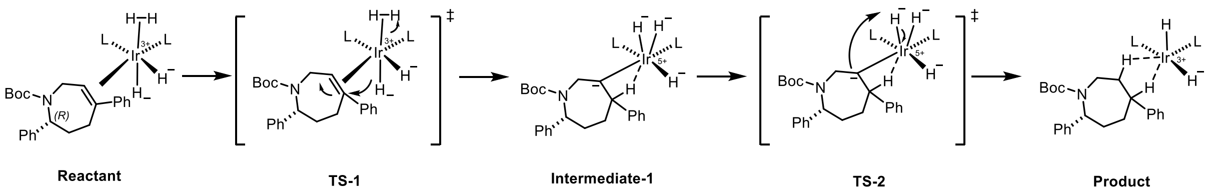

Proposed Mechanism 3

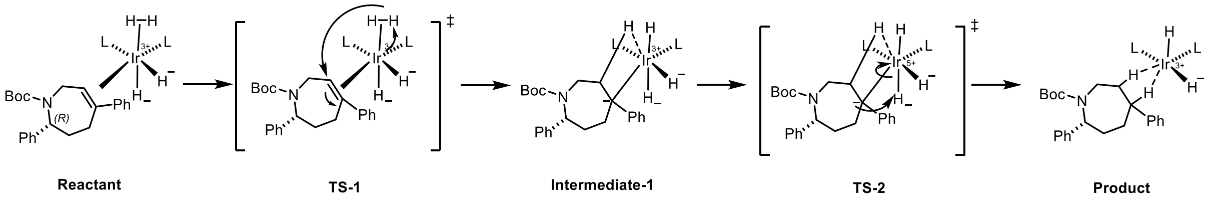

Figure S11. Proposed mechanistic pathways for the Ir-catalyzed alkene hydrogenation, highlighting the key intermediates and elementary steps considered in this study.

For Mechanisms 2 and 3, the same reactant structures were employed, differing only in the subsequent elementary steps that define the hydrogenation. All calculated structures were analyzed with respect to their potential to yield either the (2*R*,5*S*) or (2*R*,5*R*) diasteroisomer. Optimized structures of the complexes together with their relative Gibbs free energies are provided in **Table S25-S32**.

Table 25. Optimized geometries and relative Gibbs free energies of the reactant structures considered for Mechanism 1, in which the Ir complex contains no *t*-Bu_3P ligand, leading to formation of the (2*R*,5*S*) stereoisomer in the hydrogenation reaction.

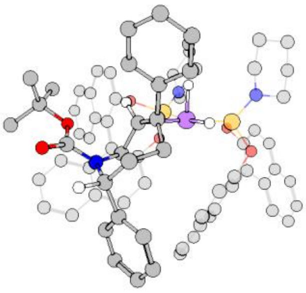<br>**Conf-1**<br>**0.0**	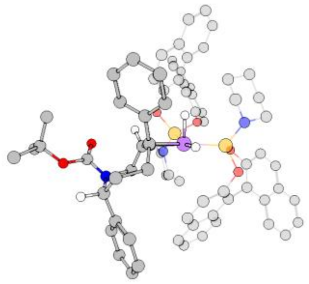<br>Conf-2<br>2.4
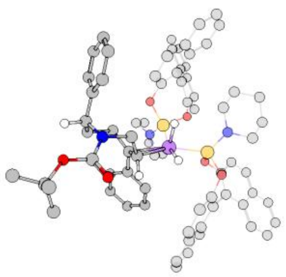<br>Conf-3<br>6.0	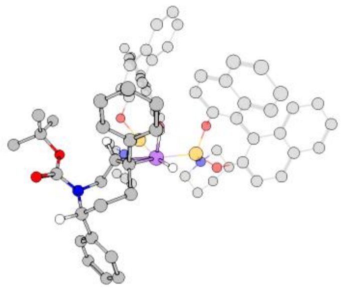<br>Conf-4<br>6.3
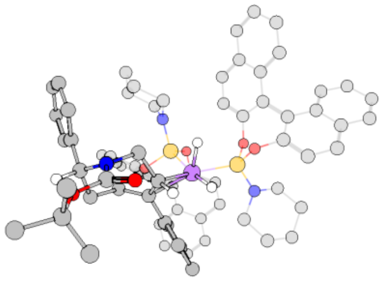<br>Conf-5<br>7.1	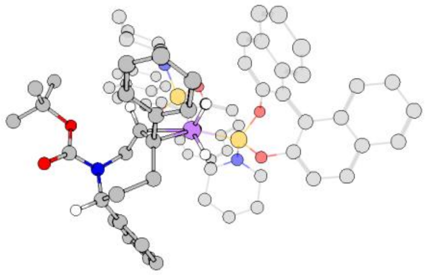<br>Conf-6<br>7.3
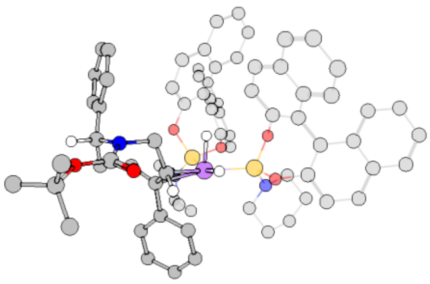<br>Conf-7<br>9.2	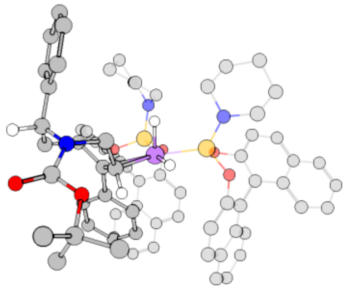<br>Conf-8<br>9.4

Table S26. Optimized geometries and relative Gibbs free energies of the reactant structures considered for Mechanism 1, in which the Ir complex contains no *t*-Bu_3P ligand, leading to formation of the (2*R*,5*R*) stereoisomer in the hydrogenation reaction.

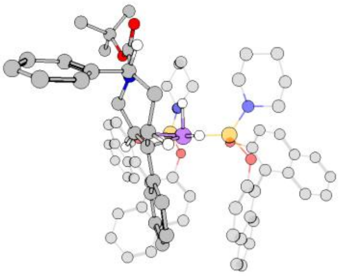<br>**Conf-9**<br>**9.9**	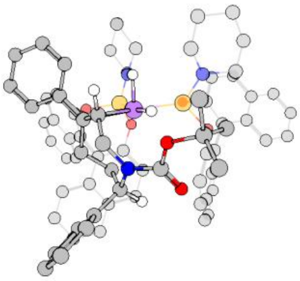<br>Conf-10<br>10.7
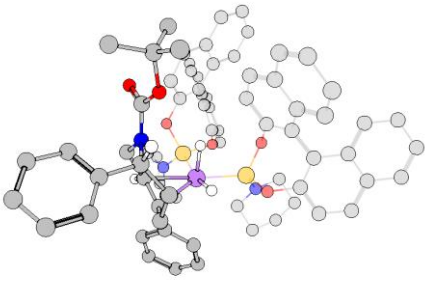<br>Conf-11<br>11.0	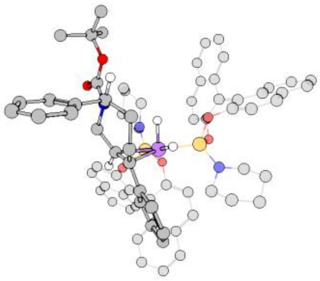<br>Conf-12<br>11.7
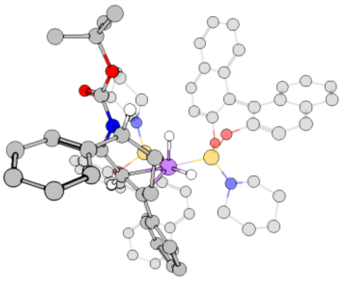<br>Conf-13<br>12.1	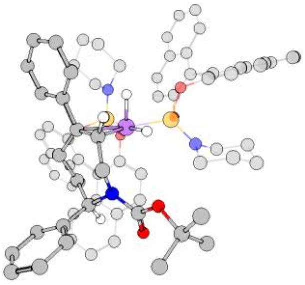<br>Conf-14<br>14.2
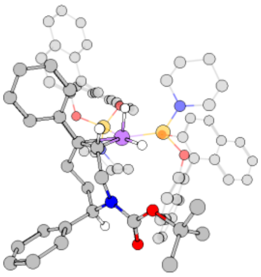<br>Conf-15<br>18.0	

Table S27. Optimized geometries and relative Gibbs free energies of the reactant structures considered for Mechanism 1, in which the Ir complex contains *t*-Bu_3P ligand, leading to formation of the (2*R*,5*S*) stereoisomer in the hydrogenation reaction.

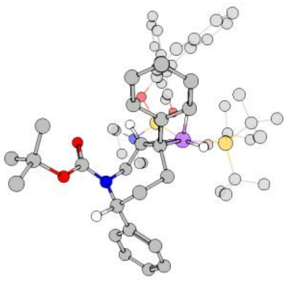<br>**Conf-1**<br>**0.0**	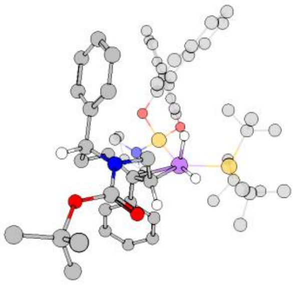<br>Conf-2<br>4.0
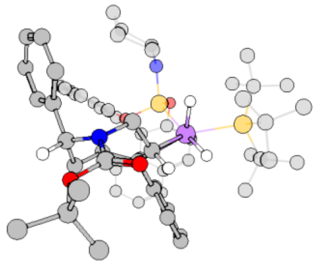<br>Conf-3<br>9.1	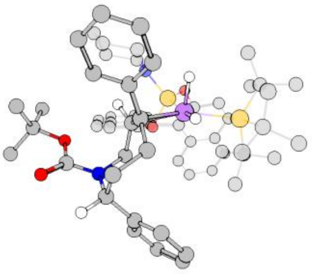<br>Conf-4<br>11.3

Table S28. Optimized geometries and relative Gibbs free energies of the reactant structures considered for Mechanism 1, in which the Ir complex contains *t*-Bu_3P ligand, leading to formation of the (2*R*,5*R*) stereoisomer in the hydrogenation reaction.

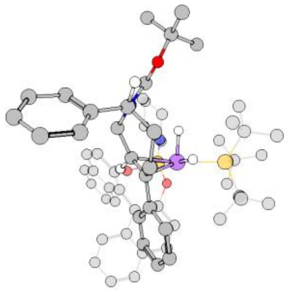 **Conf-5** **4.3**	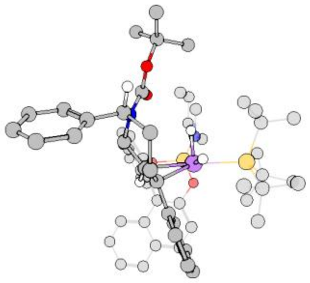 Conf-6 8.1
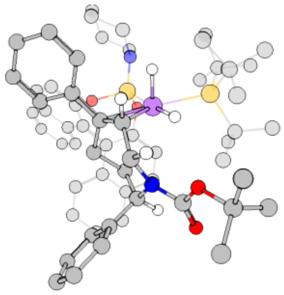 Conf-7 10.8	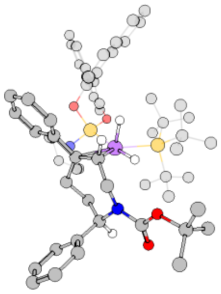 Conf-8 11.1

Table S29. Optimized geometries and relative Gibbs free energies of the reactant structures considered for Mechanism 2 and 3, in which the Ir complex contains no *t*-Bu_3P ligand, leading to formation of the (2*R*,5*S*) stereoisomer in the hydrogenation reaction.

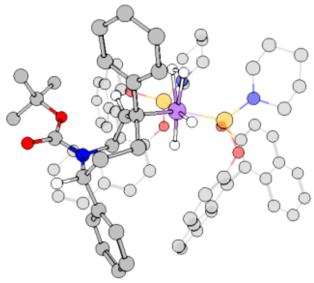<br>**Conf-1**<br>**0.0**	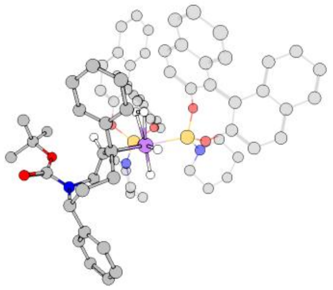<br>Conf-2<br>4.4
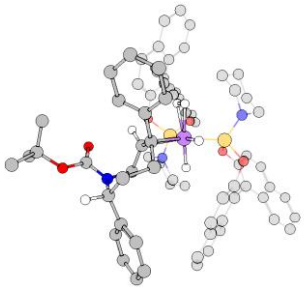<br>Conf-3<br>5.3	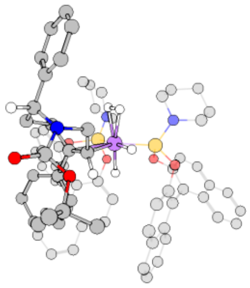<br>Conf-4<br>9.0
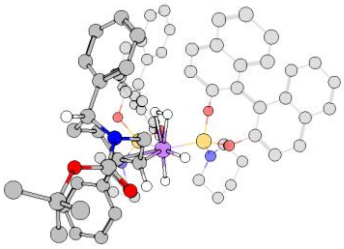<br>Conf-5<br>10.5	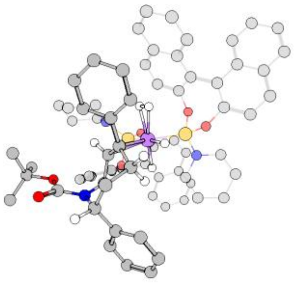<br>Conf-6<br>11.3

Table S30. Optimized geometries and relative Gibbs free energies of the reactant structures considered for Mechanism 2 and 3, in which the Ir complex contains no *t*-Bu_3P ligand, leading to formation of the (2*R*,5*R*) stereoisomer in the hydrogenation reaction.

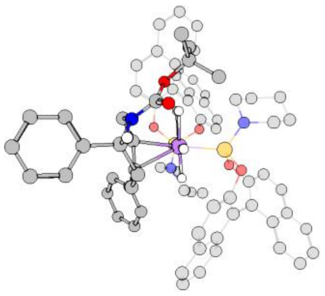<br>**Conf-7**<br>**6.1**	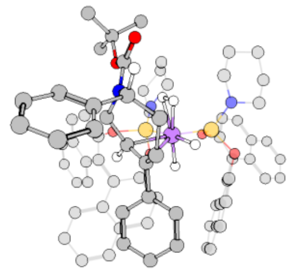<br>Conf-8<br>7.8
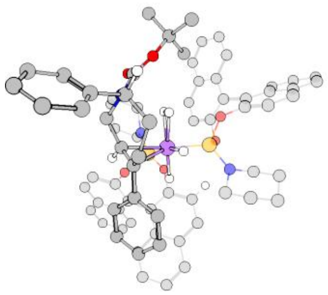<br>Conf-9<br>8.8	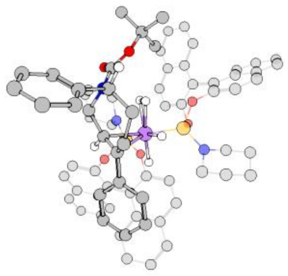<br>Conf-10<br>8.9
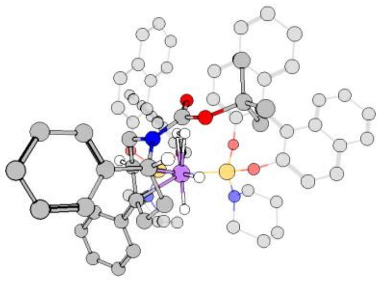<br>Conf-11<br>10.1	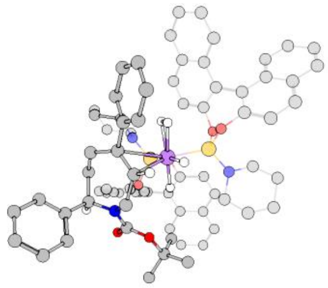<br>Conf-12<br>17.6

Table S31. Optimized geometries and relative Gibbs free energies of the reactant structures considered for Mechanism 2 and 3, in which the Ir complex contains *t*-Bu_3P ligand, leading to formation of the (2*R*,5*S*) stereoisomer in the hydrogenation reaction.

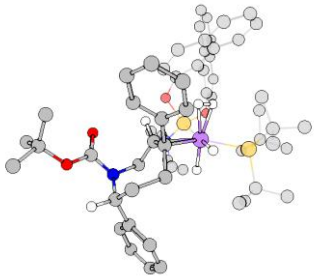<br>**Conf-1**<br>**0.0**	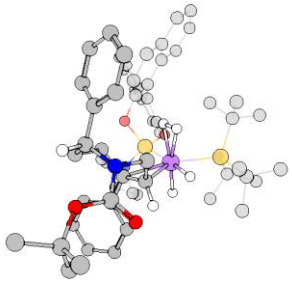<br>Conf-2<br>2.6
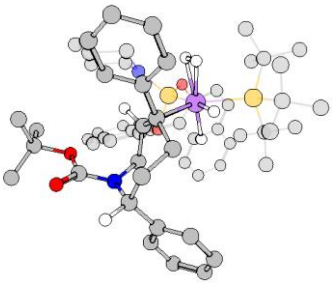<br>Conf-3<br>8.4	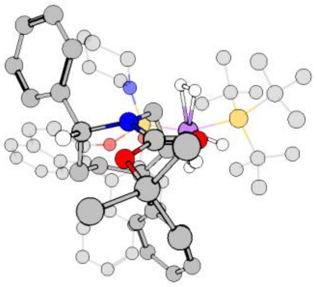<br>Conf-4<br>9.3

Table S32. Optimized geometries and relative Gibbs free energies of the reactant structures considered for Mechanism 2 and 3, in which the **Ir** complex contains *t*-Bu_3P ligand, leading to formation of the (2*R*,5*R*) stereoisomer in the hydrogenation reaction.

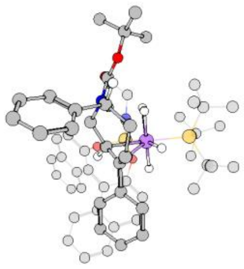<br>**Conf-5**<br>**0.9**	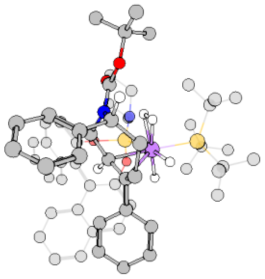<br>Conf-6<br>1.8
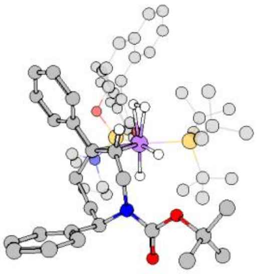<br>Conf-7<br>7.7	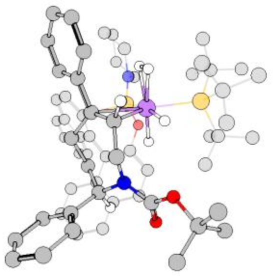<br>Conf-8<br>8.5

Across all conformational ensemble examined, including Mechanism 1 as well as Mechanism 2 and 3, both with and without a *t*-Bu_3P ligand, the reactant structures leading to the (2*R*,5*S*) stereoisomer consistently emerged as lower in energy compared to their (2*R*,5*R*)-leading counterparts. This energetic preference provides a coherent rationale for the observed stereochemical outcome. The origin of this bias can be traced to the steric influence of the Boc substituent, whose bulky nature enforces conformational arrangements that favour facial differentiation within the seven-membered ring. As a result, the Boc protecting group plays a decisive role in directing the reaction pathway toward the (2*R*,5*S*) product through sterically driven diastereoselection.

11 HPLC Traces

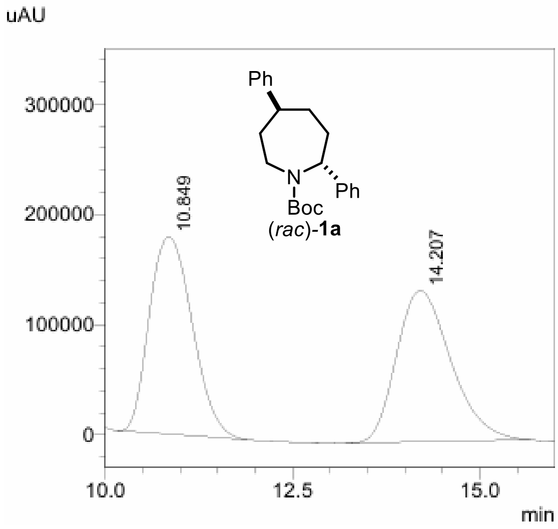

PDA Ch1 208nm

Peak#	Ret. Time	Area	Area%
1	10.849	7134376	50.698
2	14.207	6937822	49.302
Total		14072198	100.000

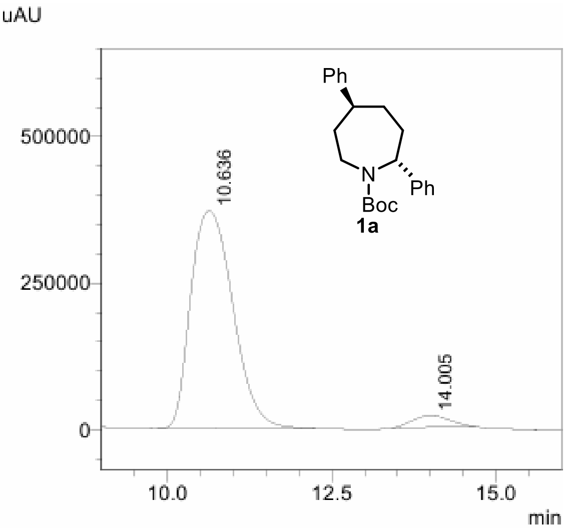

PDA Ch1 208nm

Peak#	Ret. Time	Area	Area%
1	10.636	16646205	95.527
2	14.005	779515	4.473
Total		17425720	100.000

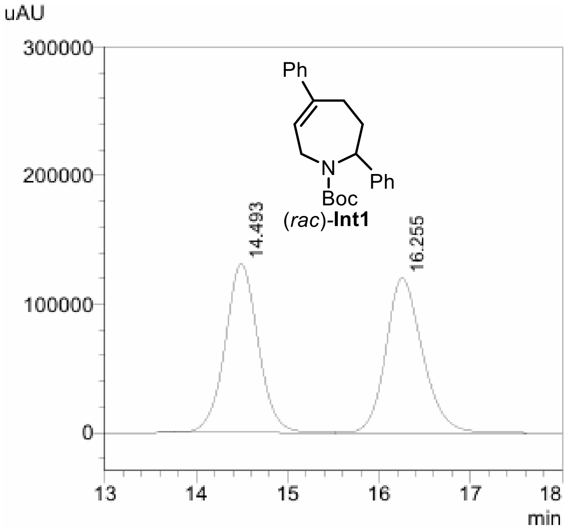

PDA Ch1 246nm

Peak#	Ret. Time	Area	Area%
1	14.493	3307517	49.654
2	16.255	3353601	50.346
Total		6661118	100.000

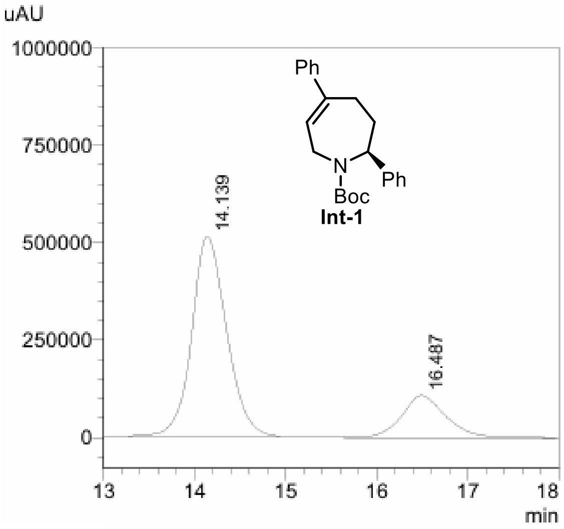

PDA Ch1 246nm

Peak#	Ret. Time	Area	Area%
1	14.139	13593995	79.088
2	16.487	3594420	20.912
Total		17188415	100.000

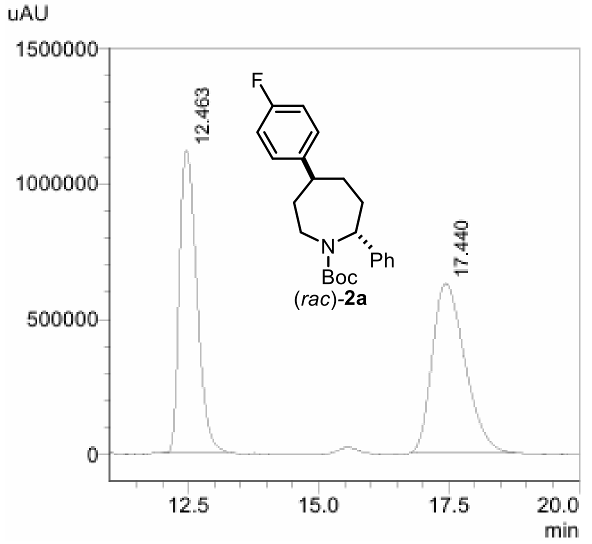

PDA Ch1 208nm

Peak#	Ret. Time	Area	Area%
1	12.463	25789535	48.585
2	17.440	27291446	51.415
Total		53080982	100.000

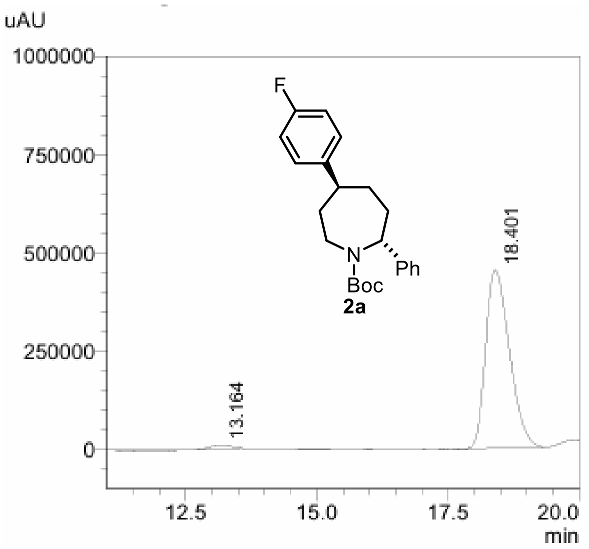

PDA Ch1 208nm

Peak#	Ret. Time	Area	Area%
1	13.164	284285	1.944
2	18.401	14338039	98.056
Total		14622324	100.000

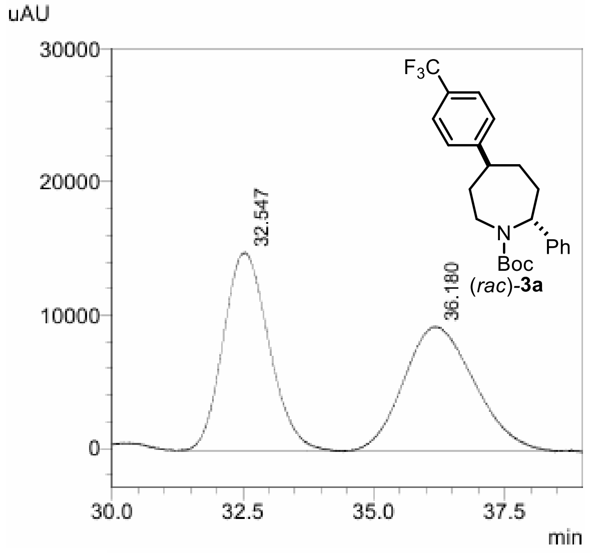

PDA Ch1 214nm

Peak#	Ret. Time	Area	Area%
1	32.547	902604	50.701
2	36.180	877632	49.299
Total		1780235	100.000

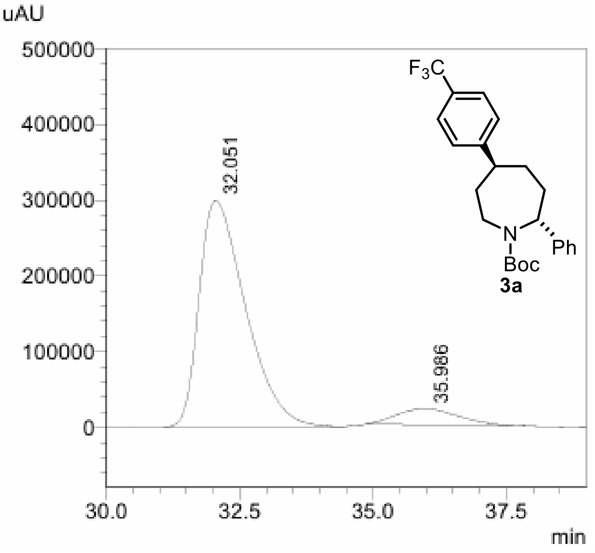

PDA Ch1 208nm

Peak#	Ret. Time	Area	Area%
1	32.051	18160054	91.404
2	35.986	1707831	8.596
Total		19867886	100.000

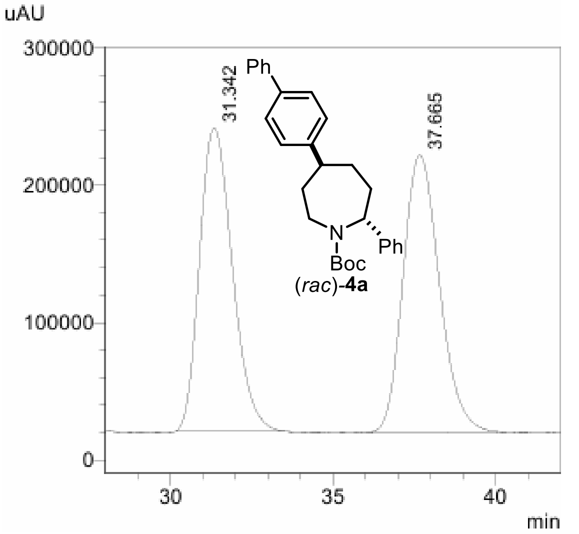

PDA Ch1 206nm

Peak#	Ret. Time	Area	Area%
1	31.342	15433401	49.653
2	37.665	15649275	50.347
Total		31082676	100.000

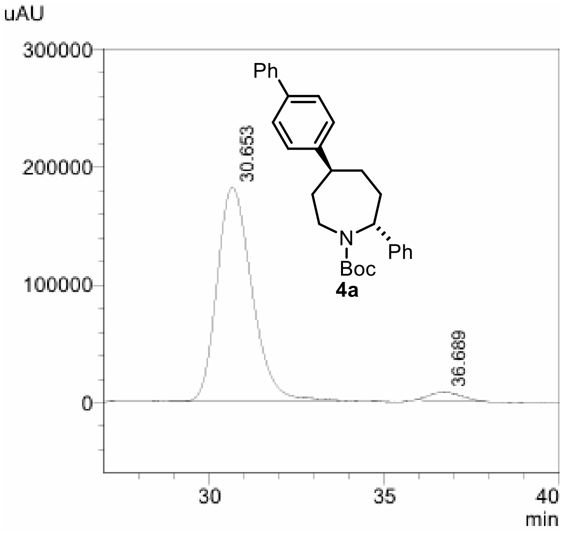

PDA Ch1 206nm

Peak#	Ret. Time	Area	Area%
1	30.653	12574249	96.612
2	36.689	440949	3.388
Total		13015199	100.000

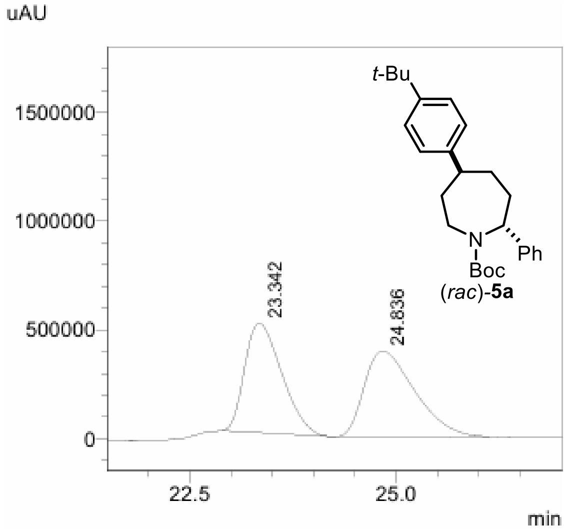

PDA Ch1 208nm

Peak#	Ret. Time	Area	Area%
1	23.342	15708146	48.312
2	24.836	16805494	51.688
Total		32513641	100.000

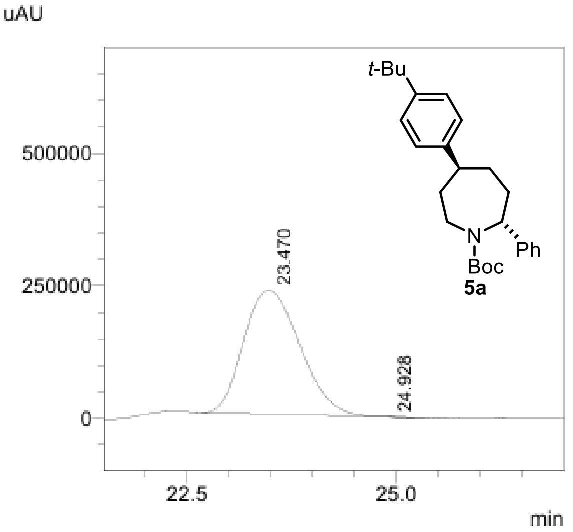

PDA Ch1 222nm

Peak#	Ret. Time	Area	Area%
1	23.470	10962172	100.004
2	24.928	-480	-0.004
Total		10961692	100.000

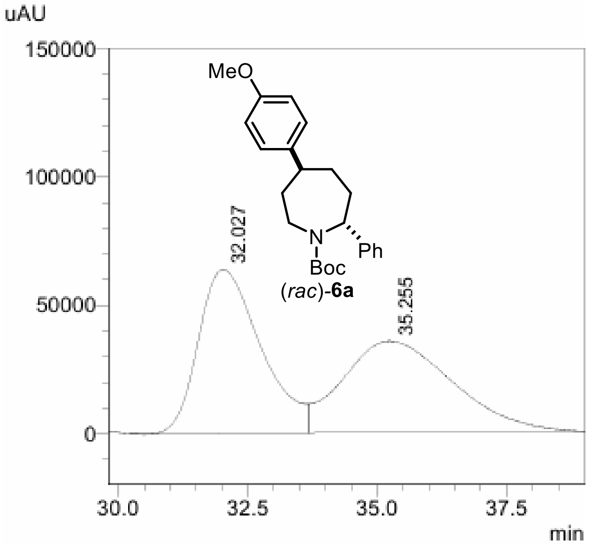

PDA Ch1 203nm

Peak#	Ret. Time	Area	Area%
1	32.027	5367983	50.415
2	35.255	5279550	49.585
Total		10647533	100.000

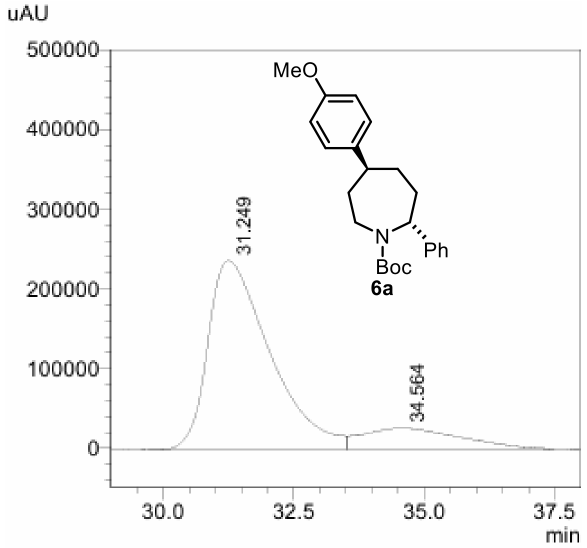

PDA Ch1 203nm

Peak#	Ret. Time	Area	Area%
1	31.249	19743128	85.000
2	34.564	3484216	15.000
Total		23227344	100.000

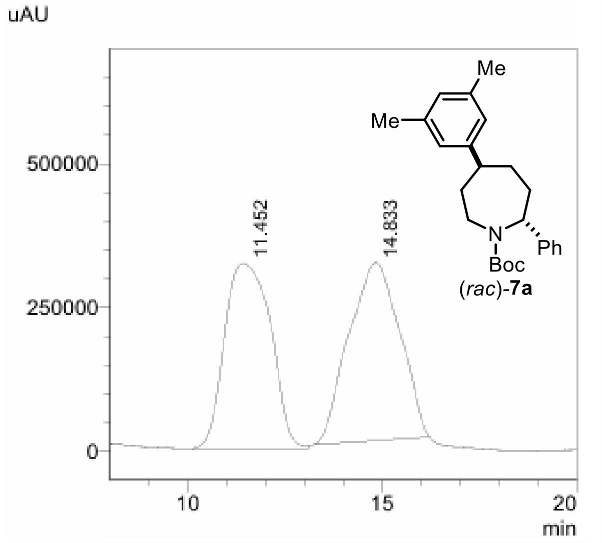

PDA Ch1 204nm

Peak#	Ret. Time	Area	Area%
1	11.452	26585500	48.519
2	14.833	28208025	51.481
Total		54793526	100.000

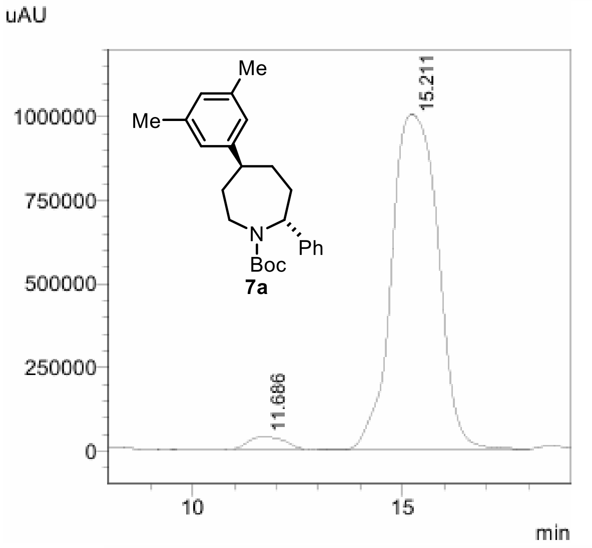

PDA Ch1 204nm

Peak#	Ret. Time	Area	Area%
1	11.686	2100835	2.652
2	15.211	77117310	97.348
Total		79218145	100.000

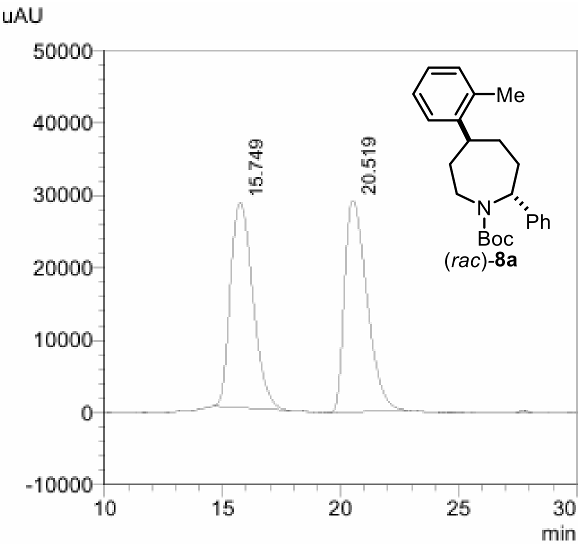

PDA Ch1 244nm

Peak#	Ret. Time	Area	Area%
1	15.749	1890658	49.403
2	20.519	1936364	50.597
Total		3827022	100.000

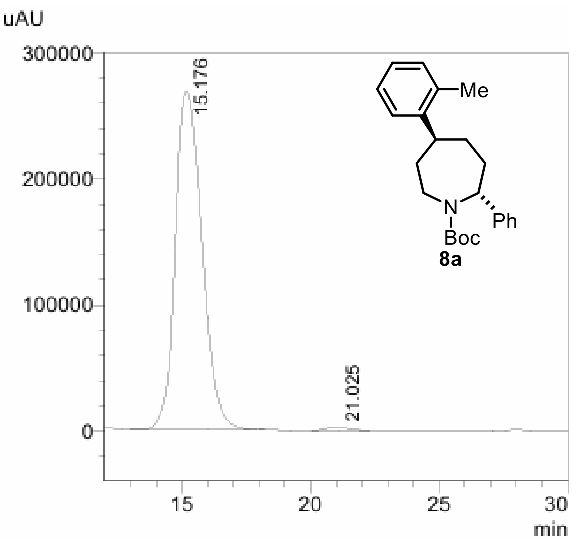

PDA Ch1 211nm

Peak#	Ret. Time	Area	Area%
1	15.176	19511289	99.176
2	21.025	162165	0.824
Total		19673454	100.000

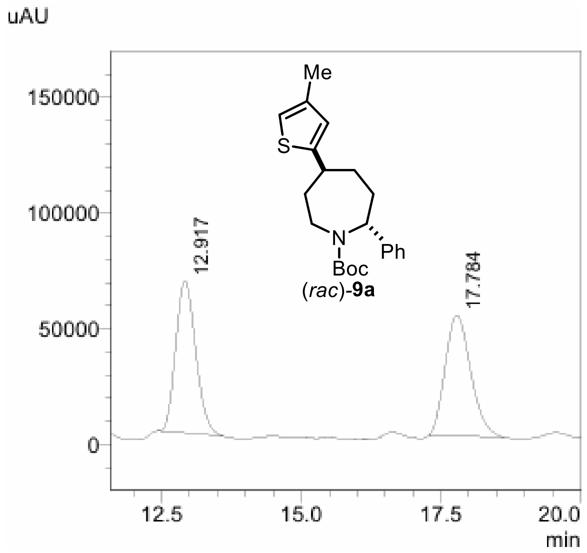

PDA Ch1 212nm

Peak#	Ret. Time	Area	Area%
1	12.917	1642377	49.692
2	17.784	1662746	50.308
Total		3305123	100.000

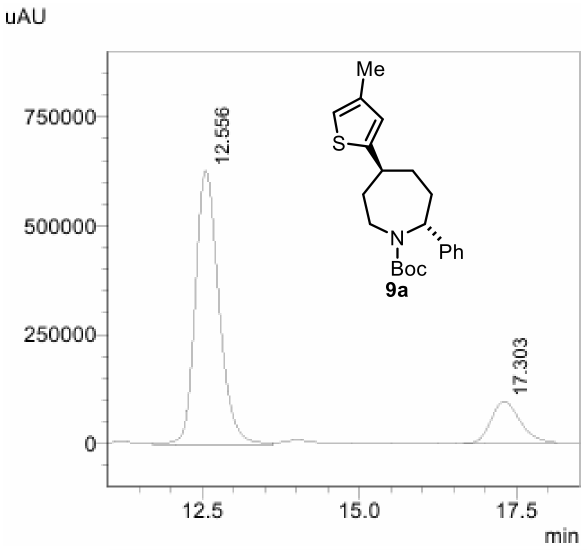

PDA Ch1 212nm

Peak#	Ret. Time	Area	Area%
1	12.556	16566284	84.469
2	17.303	3046079	15.531
Total		19612363	100.000

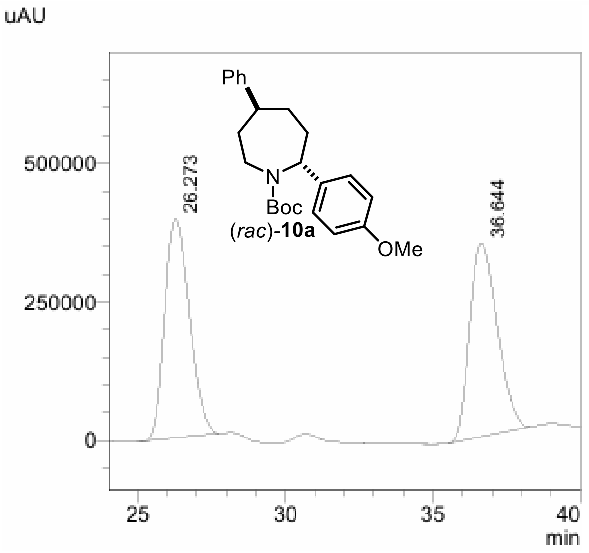

PDA Ch1 211nm

Peak#	Ret. Time	Area	Area%
1	26.273	23116994	50.440
2	36.644	22713500	49.560
Total		45830494	100.000

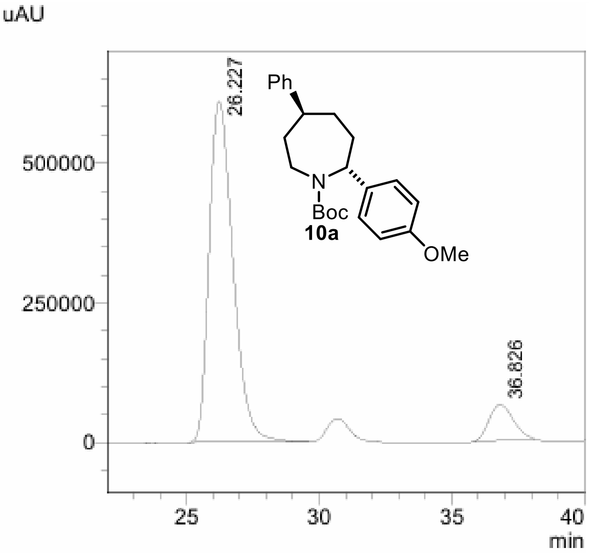

PDA Ch1 211nm

Peak#	Ret. Time	Area	Area%
1	26.227	38199303	90.397
2	36.826	4057808	9.603
Total		42257110	100.000

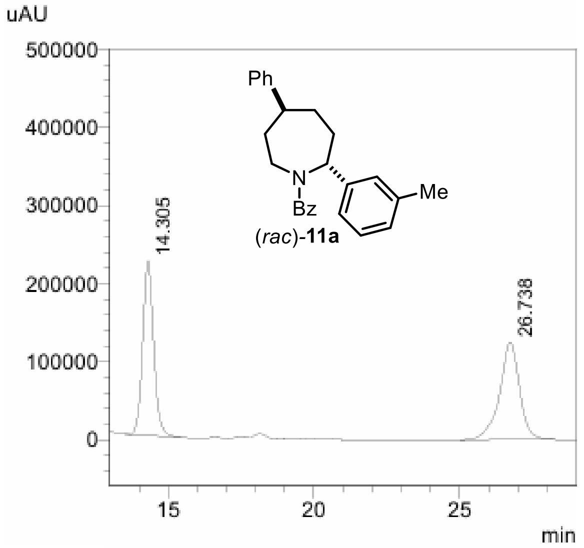

PDA Ch1 215nm

Peak#	Ret. Time	Area	Area%
1	14.305	5826345	49.460
2	26.738	5953657	50.540
Total		11780002	100.000

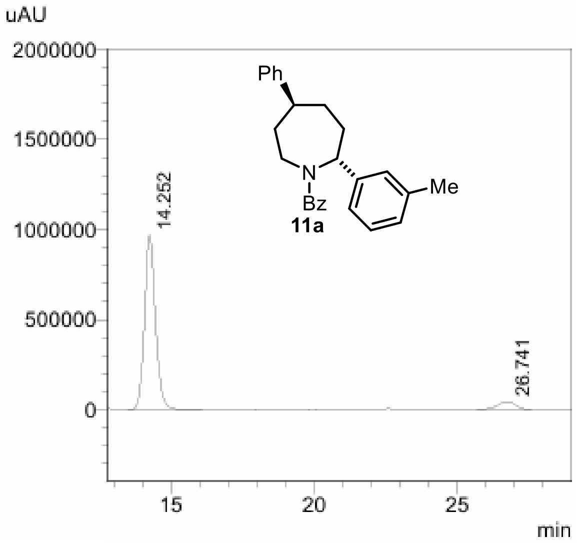

PDA Ch1 215nm

Peak#	Ret. Time	Area	Area%
1	14.252	25864467	92.844
2	26.741	1993598	7.156
Total		27858064	100.000

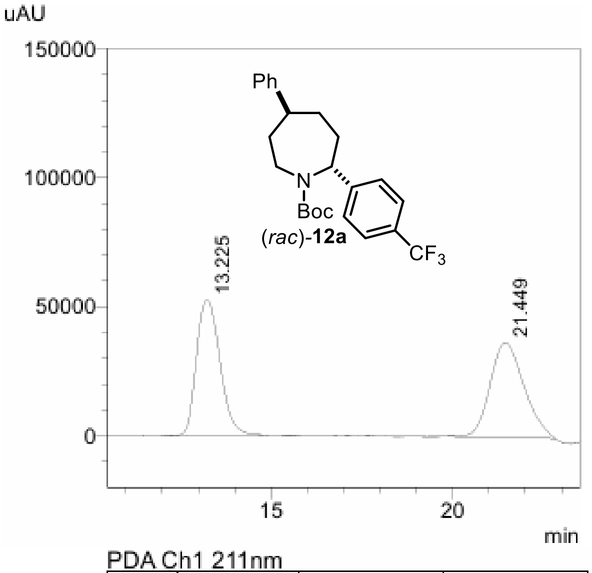

PDA Ch1 211nm

Peak#	Ret. Time	Area	Area%
1	13.225	2360860	49.779
2	21.449	2381857	50.221
Total		4742717	100.000

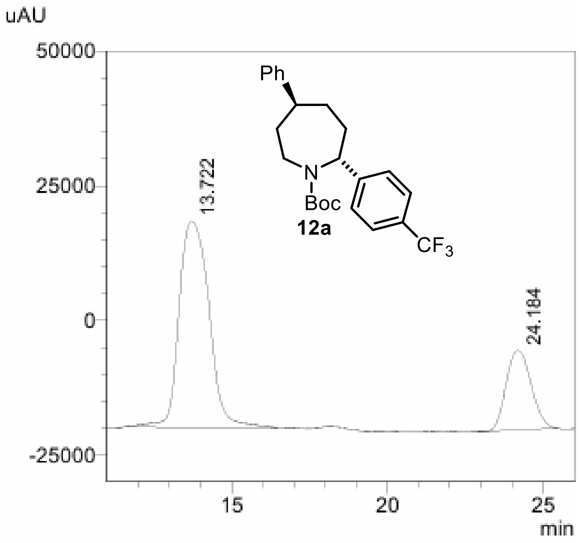

PDA Ch1 211nm

Peak#	Ret. Time	Area	Area%
1	13.722	2561495	75.593
2	24.184	827054	24.407
Total		3388549	100.000

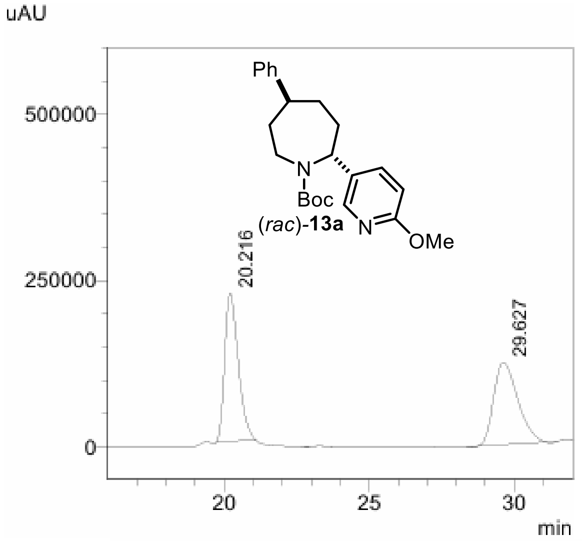

PDA Ch1 213nm

Peak#	Ret. Time	Area	Area%
1	20.216	7236517	50.478
2	29.627	7099439	49.522
Total		14335956	100.000

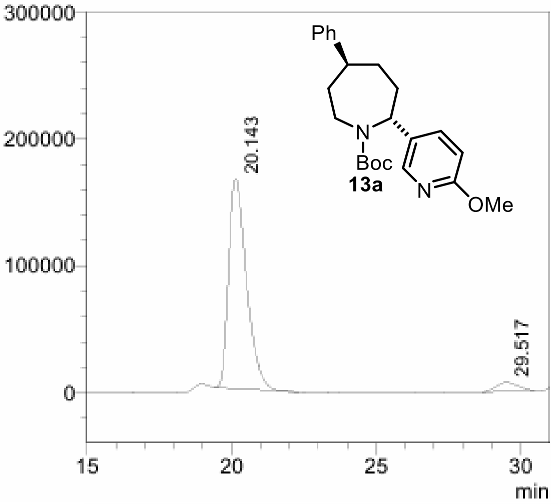

PDA Ch1 213nm

Peak#	Ret. Time	Area	Area%
1	20.143	7104122	95.039
2	29.517	370799	4.961
Total		7474922	100.000

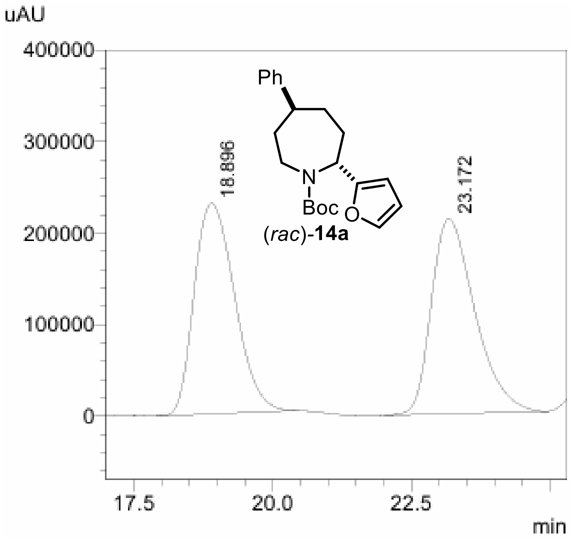

PDA Ch1 208nm

Peak#	Ret. Time	Area	Area%
1	18.896	11700157	50.153
2	23.172	11628945	49.847
Total		23329102	100.000

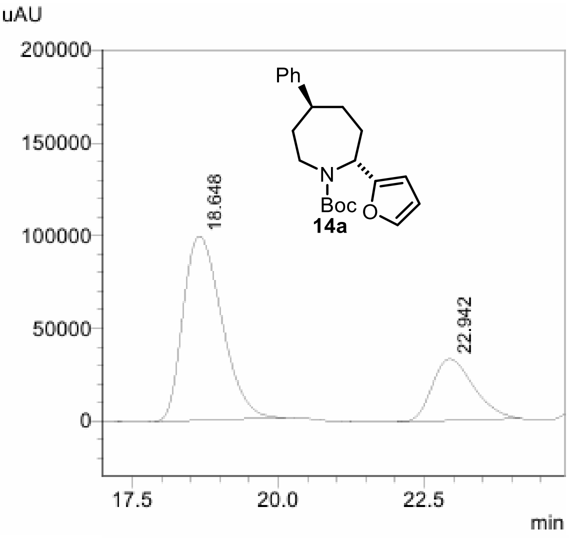

PDA Ch1 229nm

Peak#	Ret. Time	Area	Area%
1	18.648	4583828	73.693
2	22.942	1636327	26.307
Total		6220155	100.000

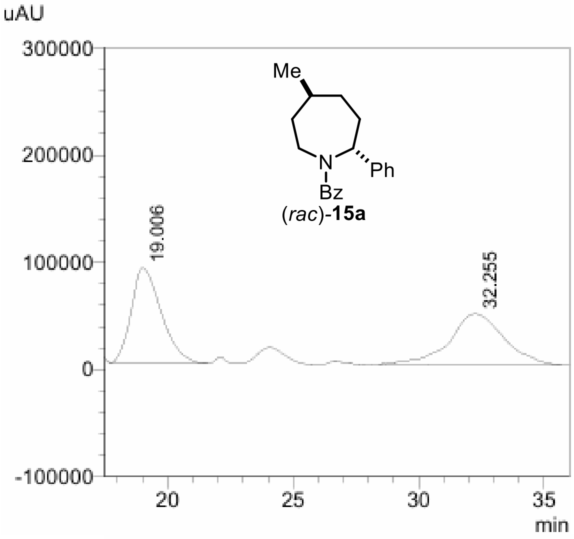

PDA Ch1 211nm

Peak#	Ret. Time	Area	Area%
1	19.006	7314116	50.794
2	32.255	7085582	49.206
Total		14399698	100.000

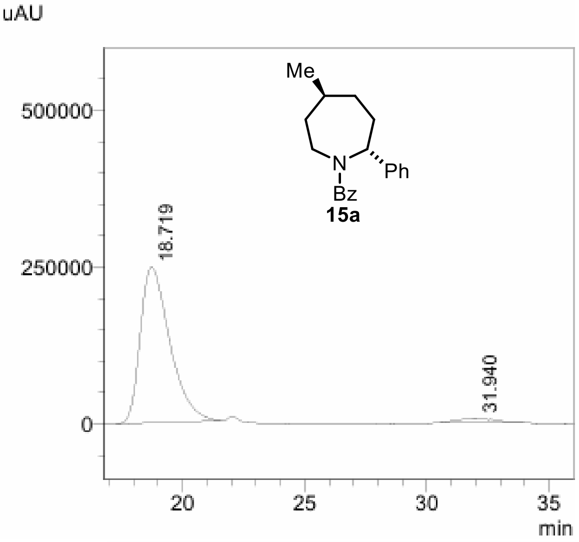

PDA Ch1 211nm

Peak#	Ret. Time	Area	Area%
1	18.719	20952206	96.150
2	31.940	838935	3.850
Total		21791141	100.000

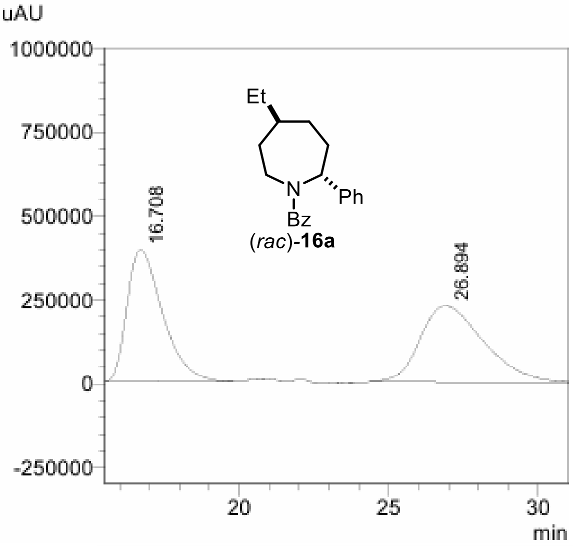

PDA Ch1 204nm

Peak#	Ret. Time	Area	Area%
1	16.708	32240765	49.027
2	26.894	33520848	50.973
Total		65761614	100.000

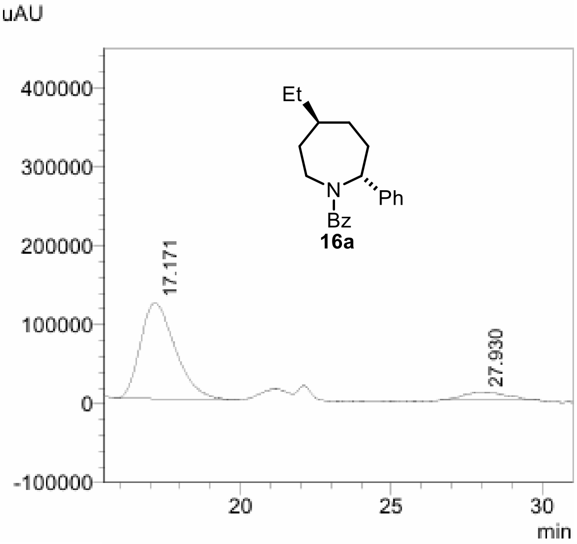

PDA Ch1 204nm

Peak#	Ret. Time	Area	Area%
1	17.171	9803390	90.592
2	27.930	1018050	9.408
Total		10821440	100.000

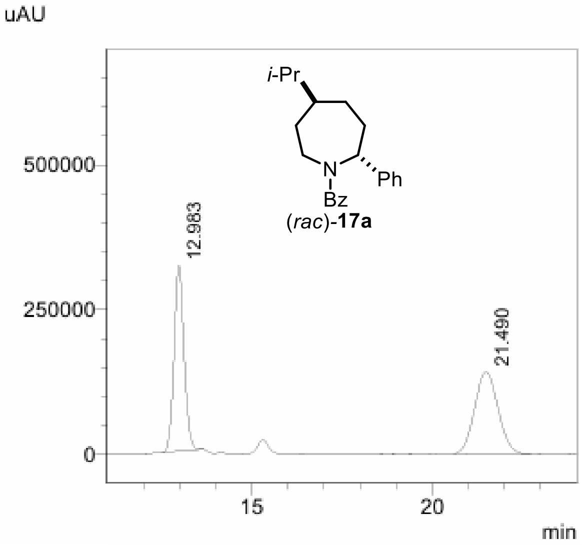

PDA Ch1 209nm

Peak#	Ret. Time	Area	Area%
1	12.983	6180538	49.242
2	21.490	6370875	50.758
Total		12551413	100.000

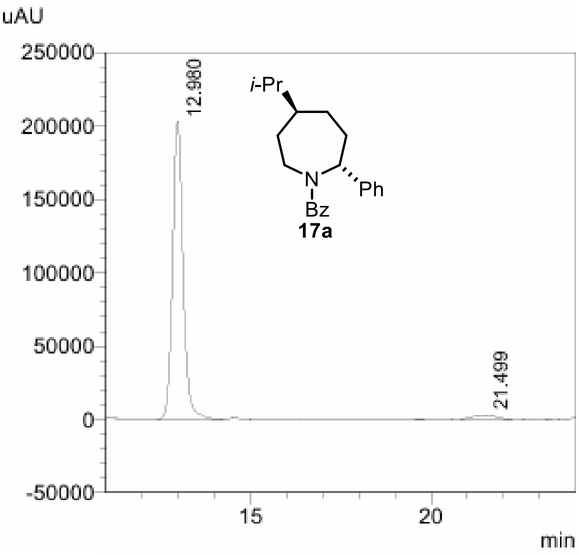

PDA Ch1 209nm

Peak#	Ret. Time	Area	Area%
1	12.980	4075425	96.949
2	21.499	128240	3.051
Total		4203666	100.000

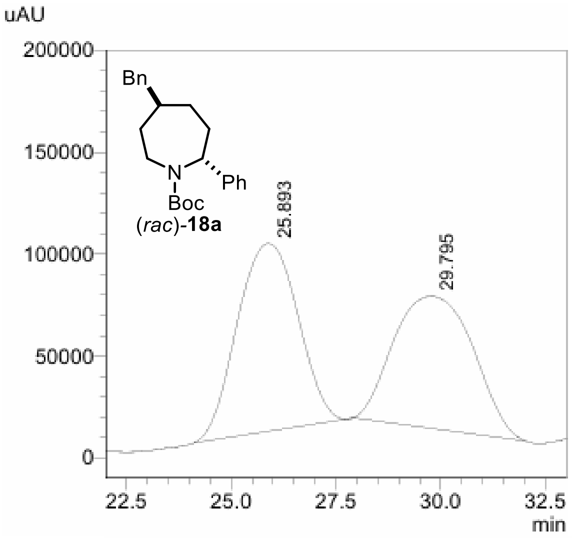

PDA Ch1 207nm

Peak#	Ret. Time	Area	Area%
1	25.893	9297248	51.940
2	29.795	8602826	48.060
Total		17900074	100.000

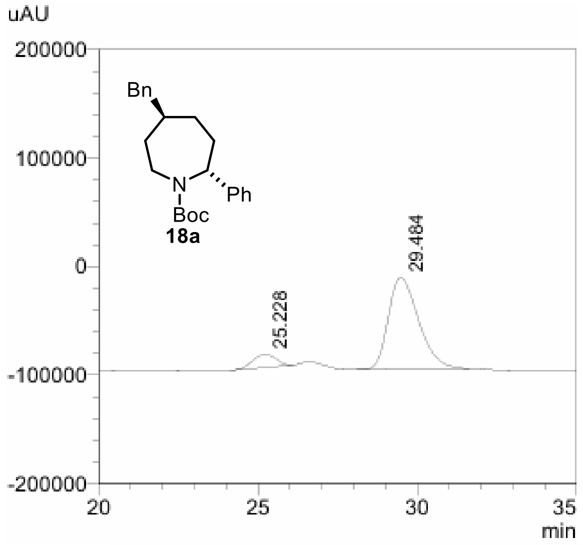

PDA Ch1 217nm

Peak#	Ret. Time	Area	Area%
1	25.228	606098	9.764
2	29.484	5601411	90.236
Total		6207509	100.000

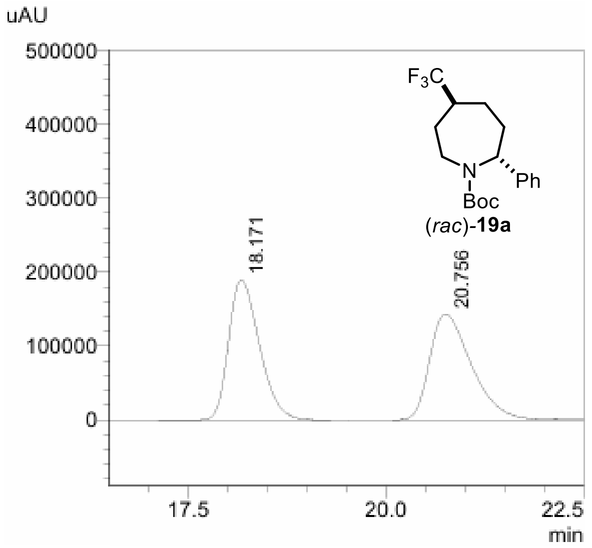

PDA Ch1 208nm

Peak#	Ret. Time	Area	Area%
1	18.171	5155282	49.517
2	20.756	5255895	50.483
Total		10411177	100.000

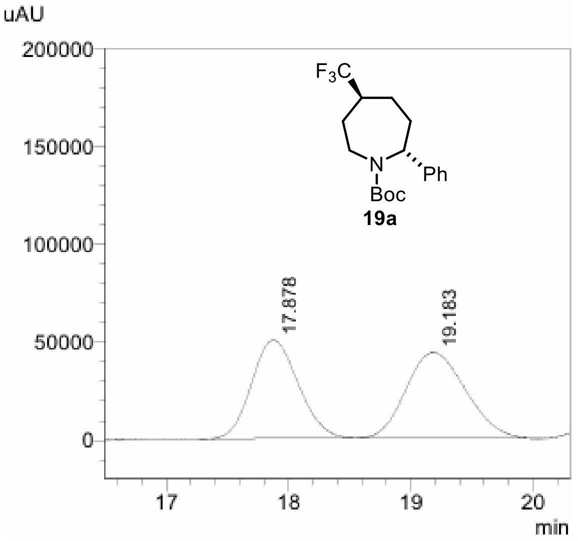

PDA Ch1 207nm

Peak#	Ret. Time	Area	Area%
1	17.878	1334645	47.276
2	19.183	1488437	52.724
Total		2823082	100.000

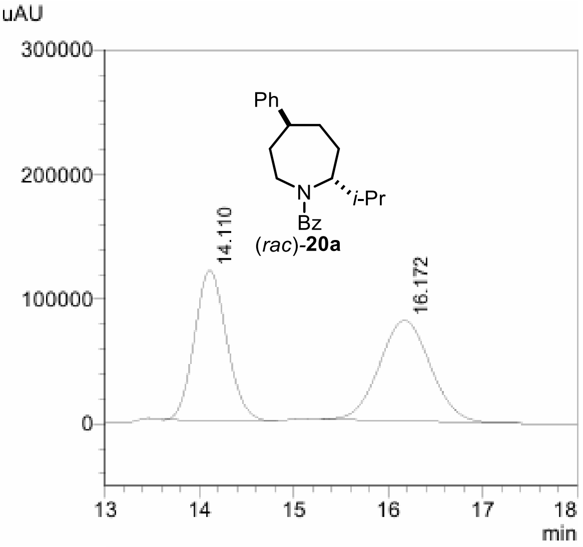

PDA Ch1 211nm

Peak#	Ret. Time	Area	Area%
1	14.110	2861697	48.384
2	16.172	3052894	51.616
Total		5914590	100.000

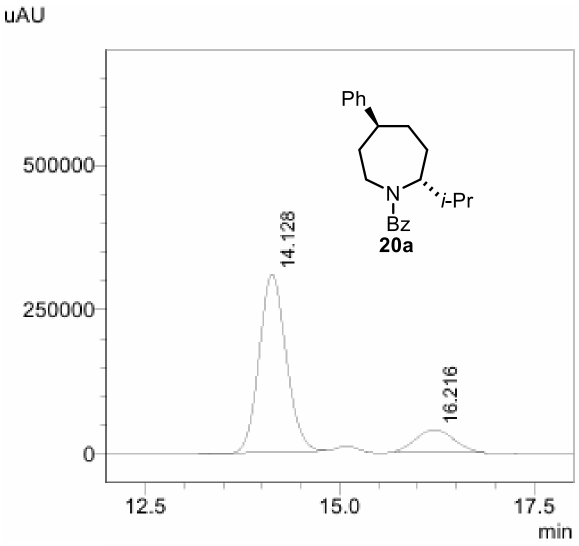

PDA Ch1 211nm

Peak#	Ret. Time	Area	Area%
1	14.128	7433279	85.024
2	16.216	1309304	14.976
Total		8742583	100.000

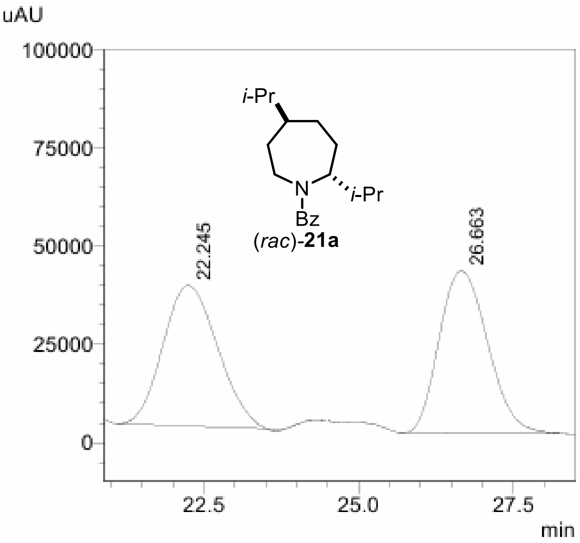

PDA Ch1 216nm

Peak#	Ret. Time	Area	Area%
1	22.245	2193289	49.522
2	26.663	2235630	50.478
Total		4428919	100.000

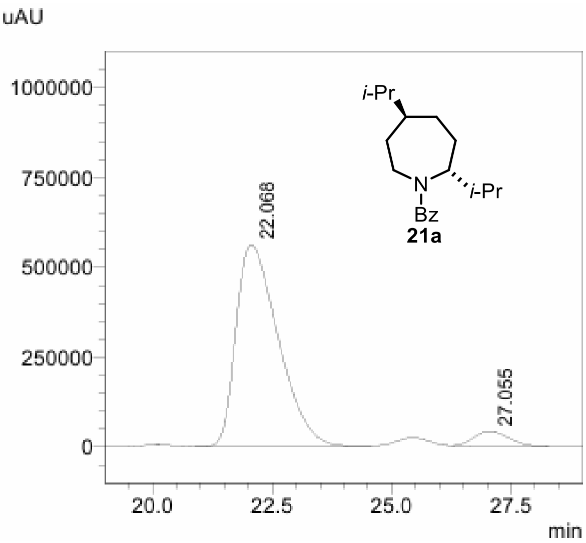

PDA Ch1 216nm

Peak#	Ret. Time	Area	Area%
1	22.068	34482832	94.324
2	27.055	2075038	5.676
Total		36557870	100.000

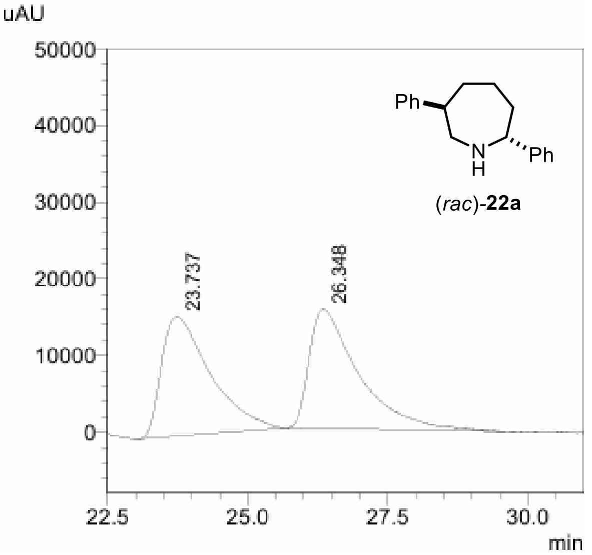

PDA Ch1 210nm

Peak#	Ret. Time	Area	Area%
1	23.737	945687	49.742
2	26.348	955491	50.258
Total		1901178	100.000

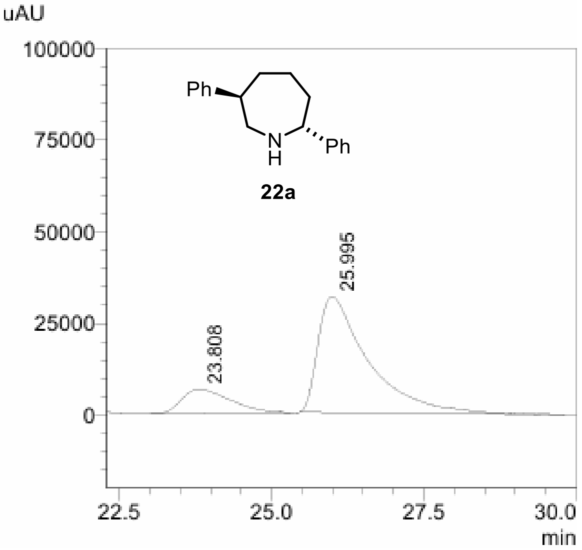

PDA Ch1 210nm

Peak#	Ret. Time	Area	Area%
1	23.808	362628	16.643
2	25.995	1816296	83.357
Total		2178925	100.000

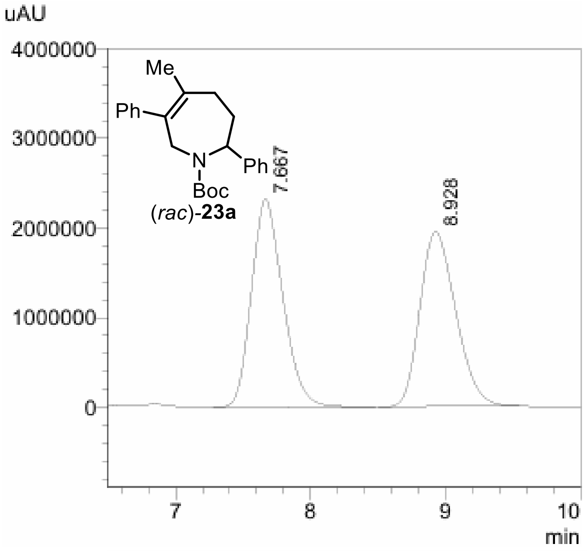

PDA Ch1 232nm

Peak#	Ret. Time	Area	Area%
1	7.667	37471090	50.973
2	8.928	36040856	49.027
Total		73511946	100.000

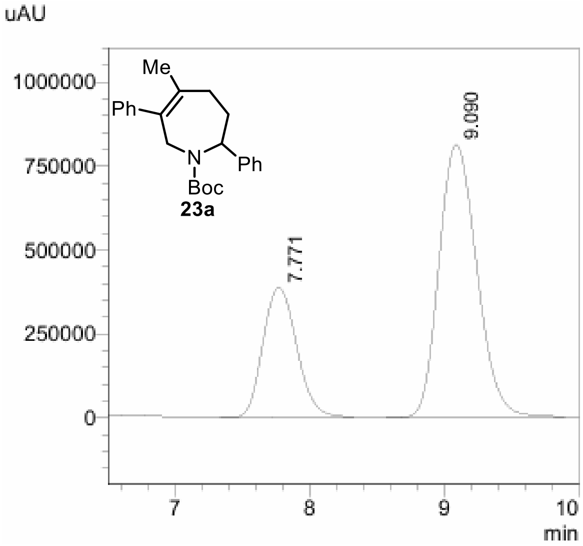

PDA Ch1 232nm

Peak#	Ret. Time	Area	Area%
1	7.771	6729071	29.769
2	9.090	15875122	70.231
Total		22604193	100.000

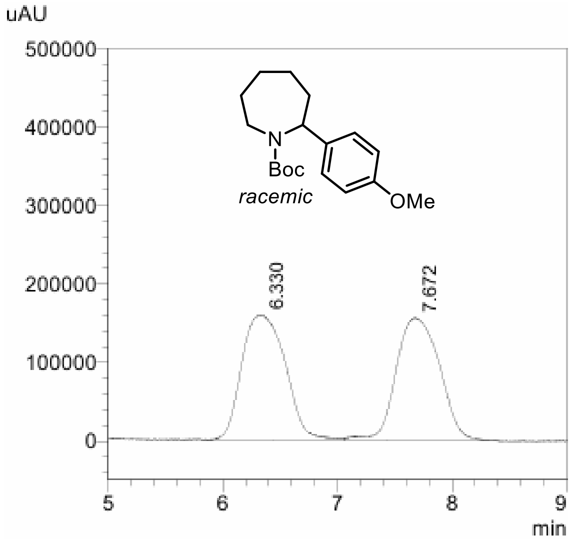

PDA Ch1 203nm

Peak#	Ret. Time	Area	Area%
1	6.330	4194603	50.430
2	7.672	4123100	49.570
Total		8317704	100.000

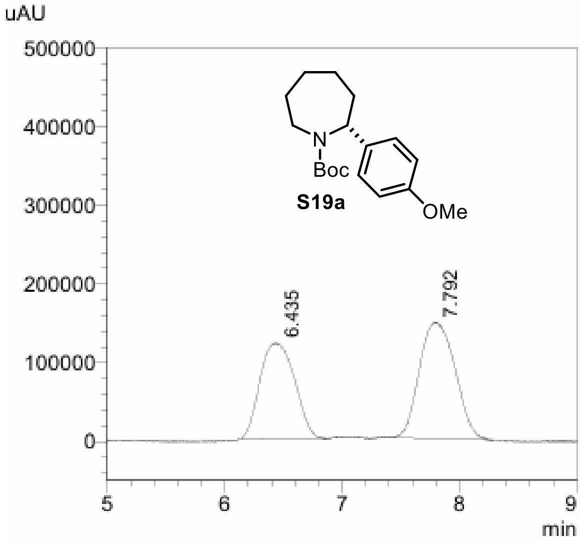

PDA Ch1 203nm

Peak#	Ret. Time	Area	Area%
1	6.435	2473777	44.132
2	7.792	3131572	55.868
Total		5605349	100.000

12 NMR Spectra

S4 – ^{1}H NMR (600 MHz, $CDCl_3$)

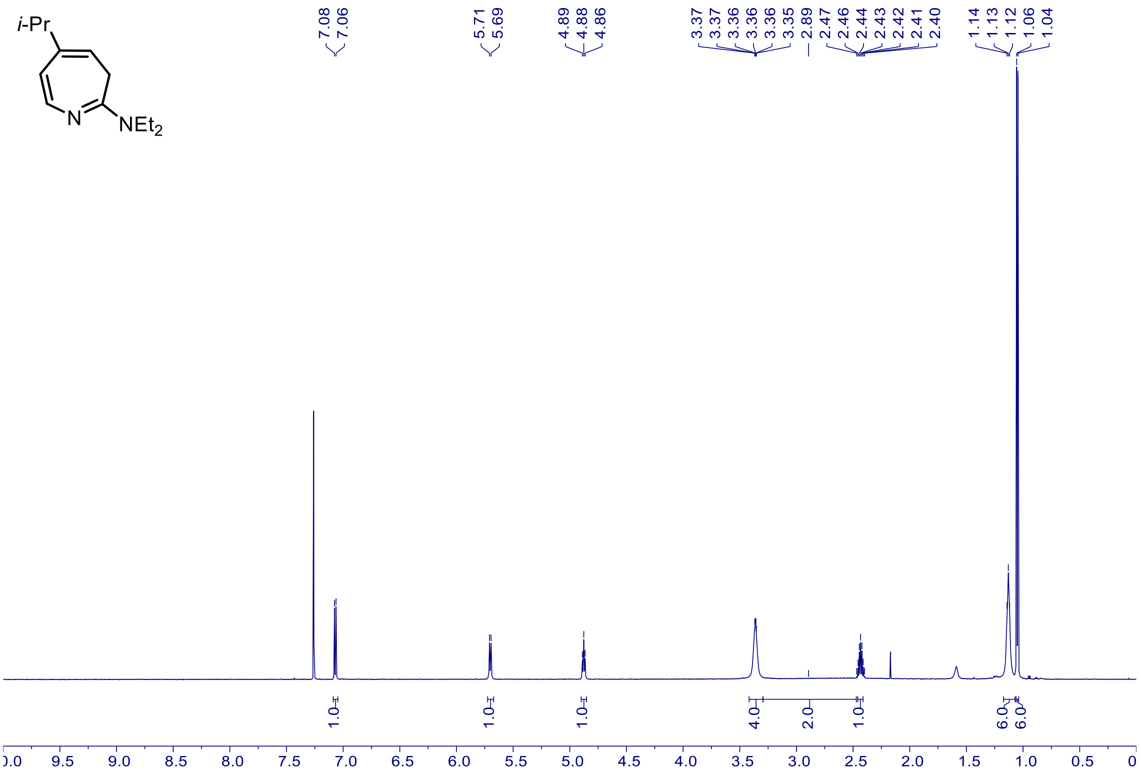

S4 – ^{13}C NMR (151 MHz, $CDCl_3$)

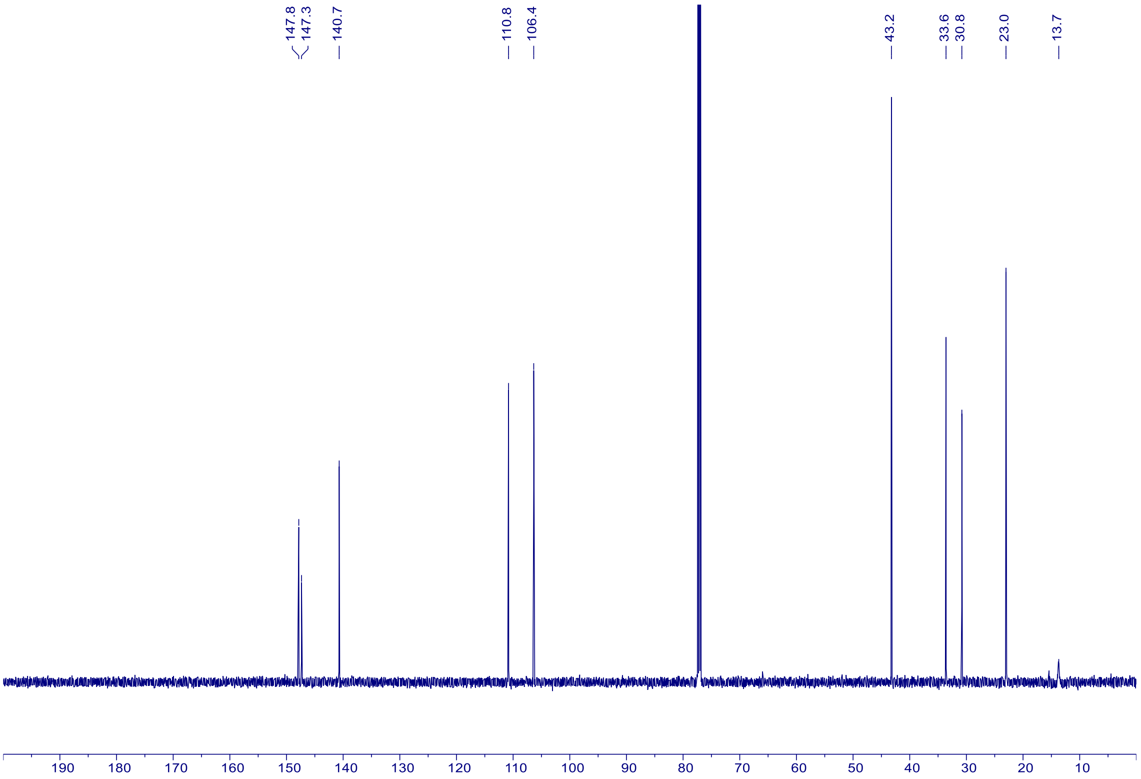

S11 – ^{1}H NMR (600 MHz, $CDCl_3$)

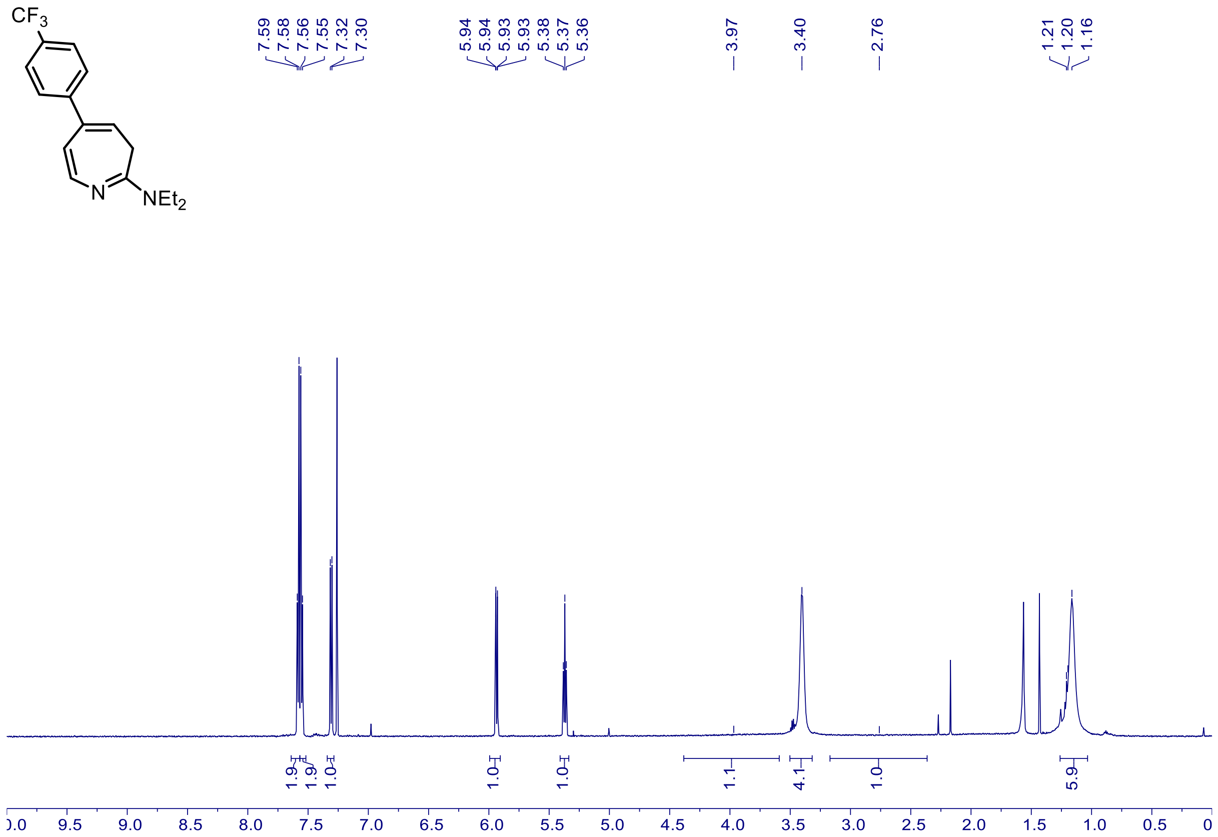

S11 – ^{13}C NMR (151 MHz, $CDCl_3$)

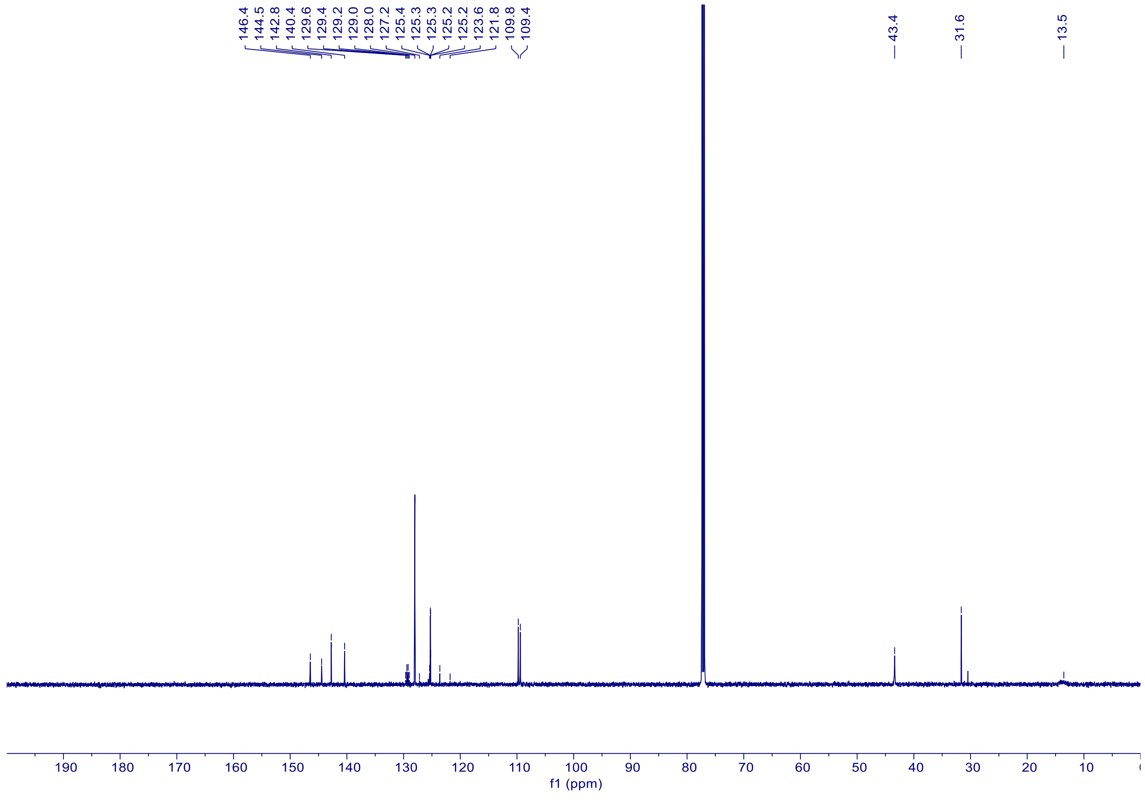

S11 – ^{19}F NMR (564 MHz, $CDCl_3$)

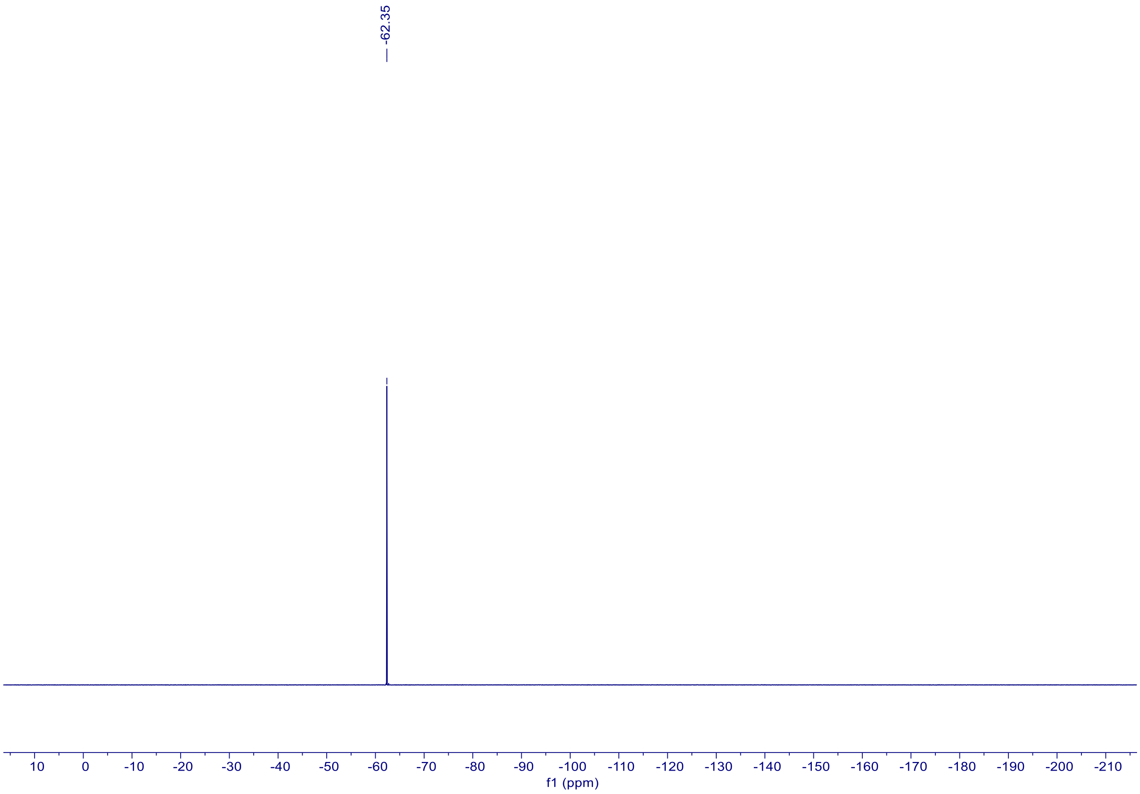

S12 – ^{1}H NMR (600 MHz, $CDCl_3$)

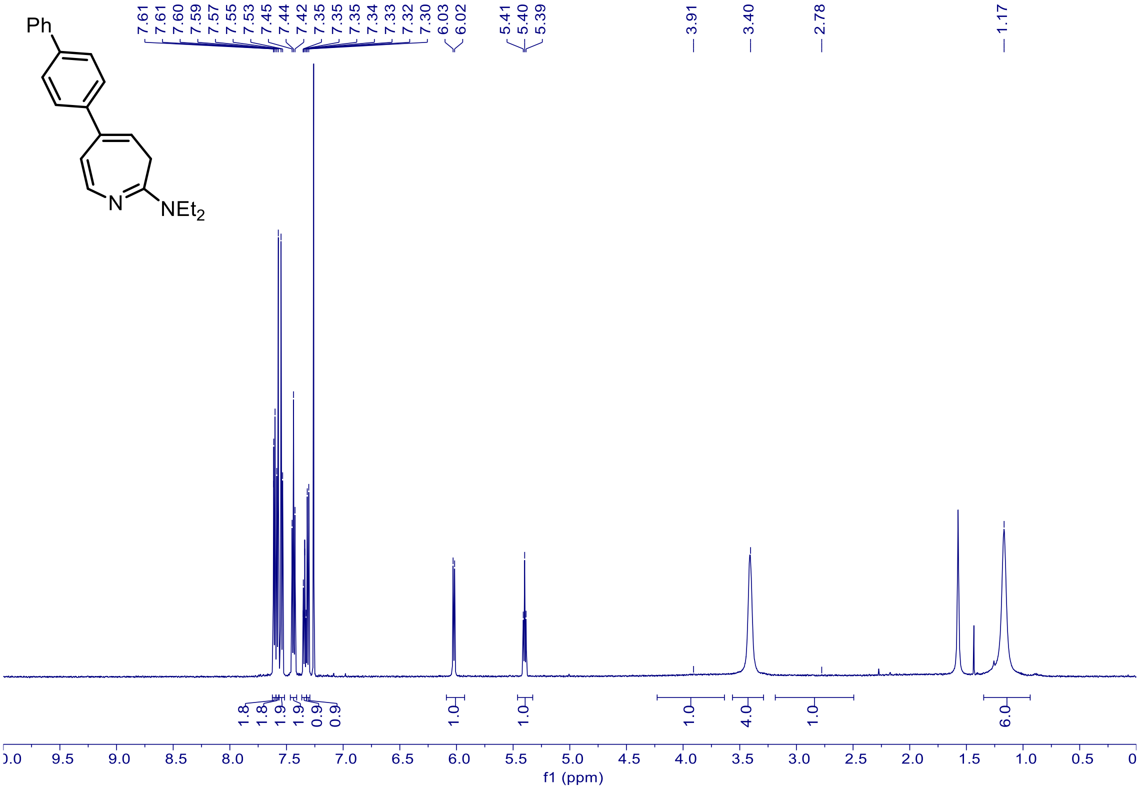

S12 – ^{13}C NMR (151 MHz, $CDCl_3$)

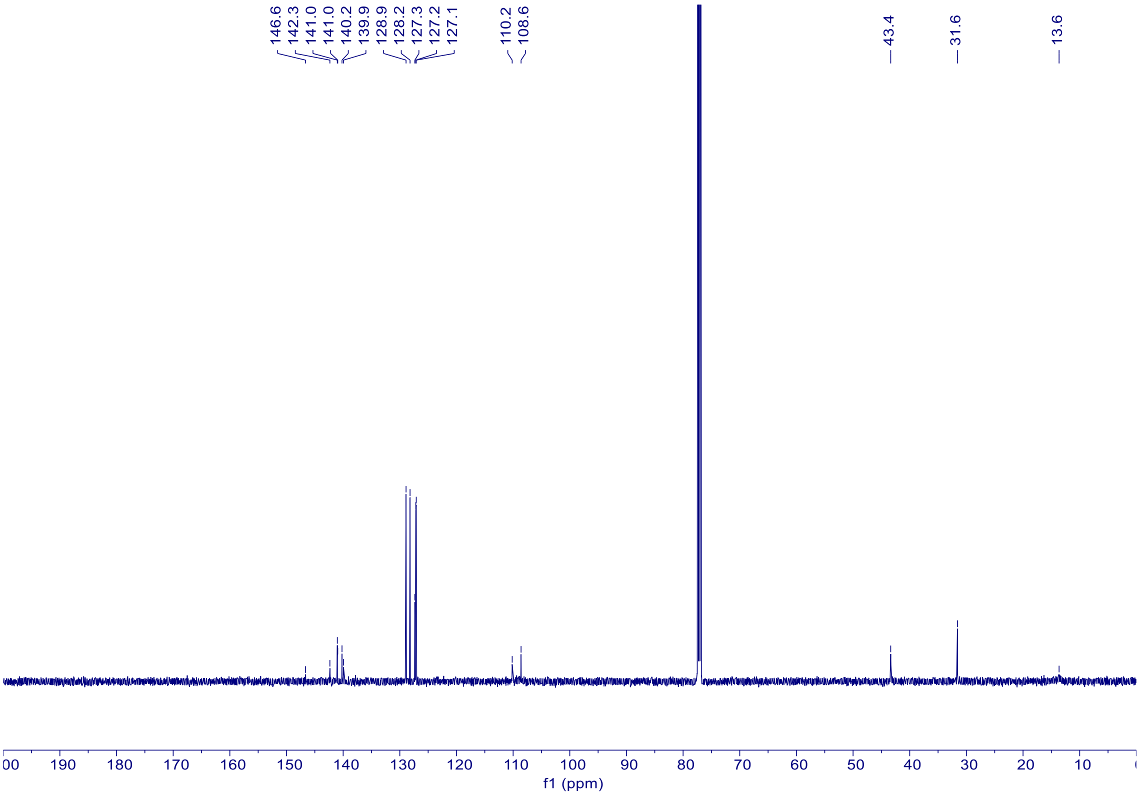

S13 – ^{1}H NMR (600 MHz, $CDCl_3$)

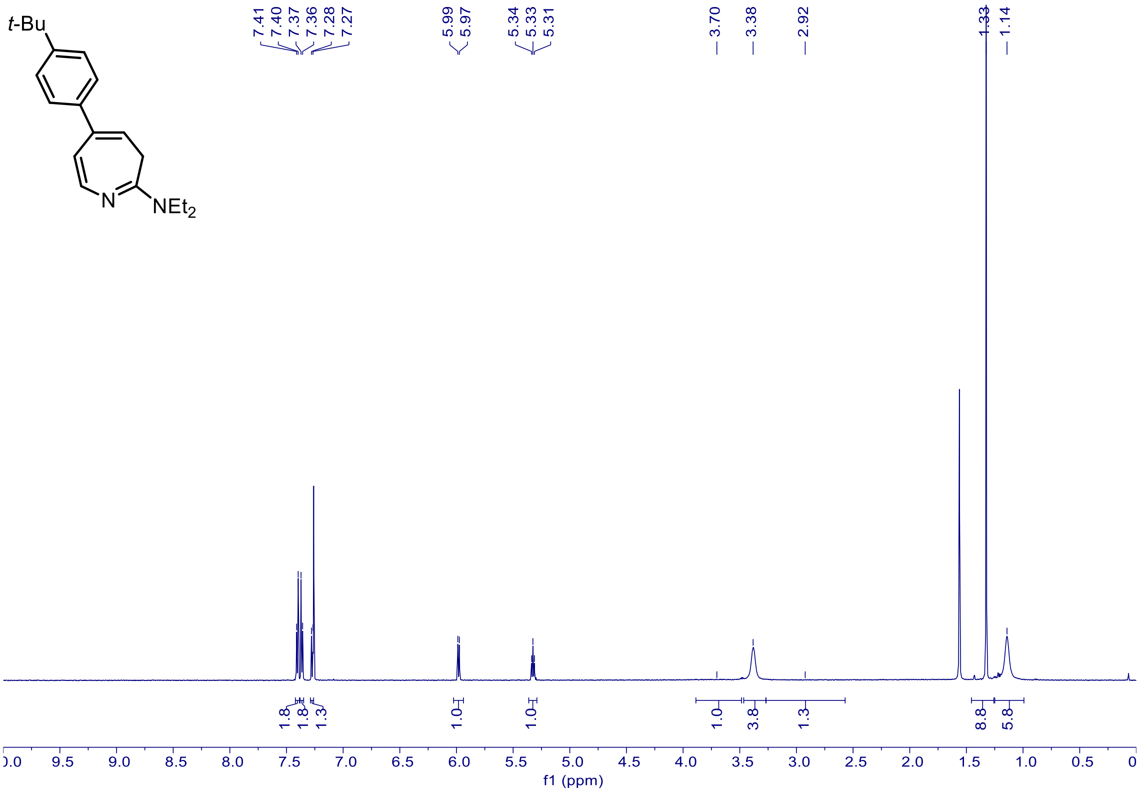

S13 – ^{13}C NMR (151 MHz, $CDCl_3$)

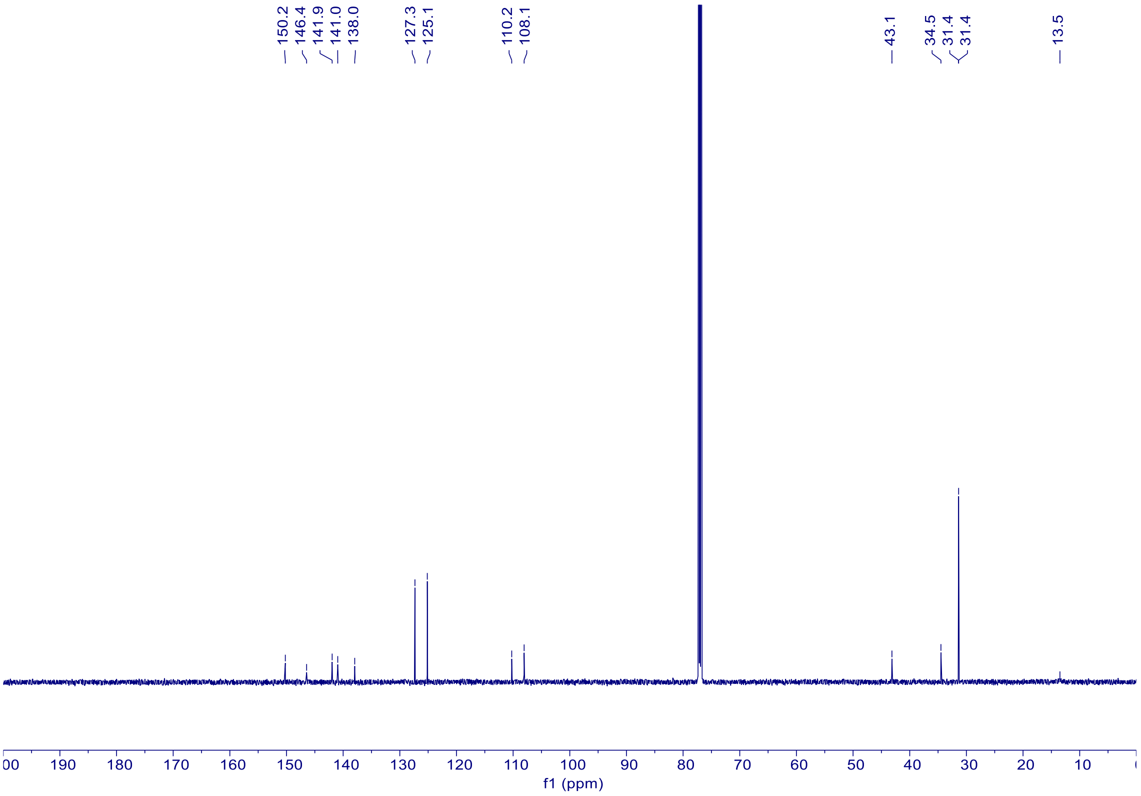

S15 – ¹H NMR (600 MHz, CDCl$_3$)

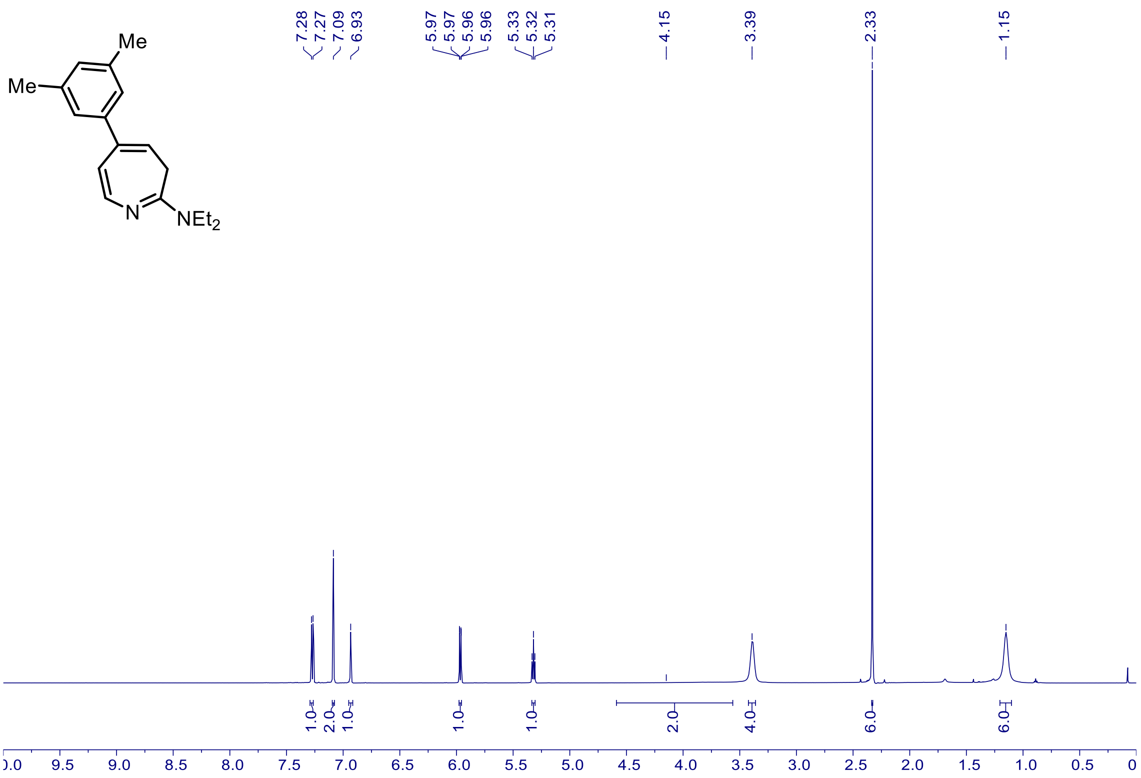

S15 – ¹³C NMR (151 MHz, CDCl$_3$)

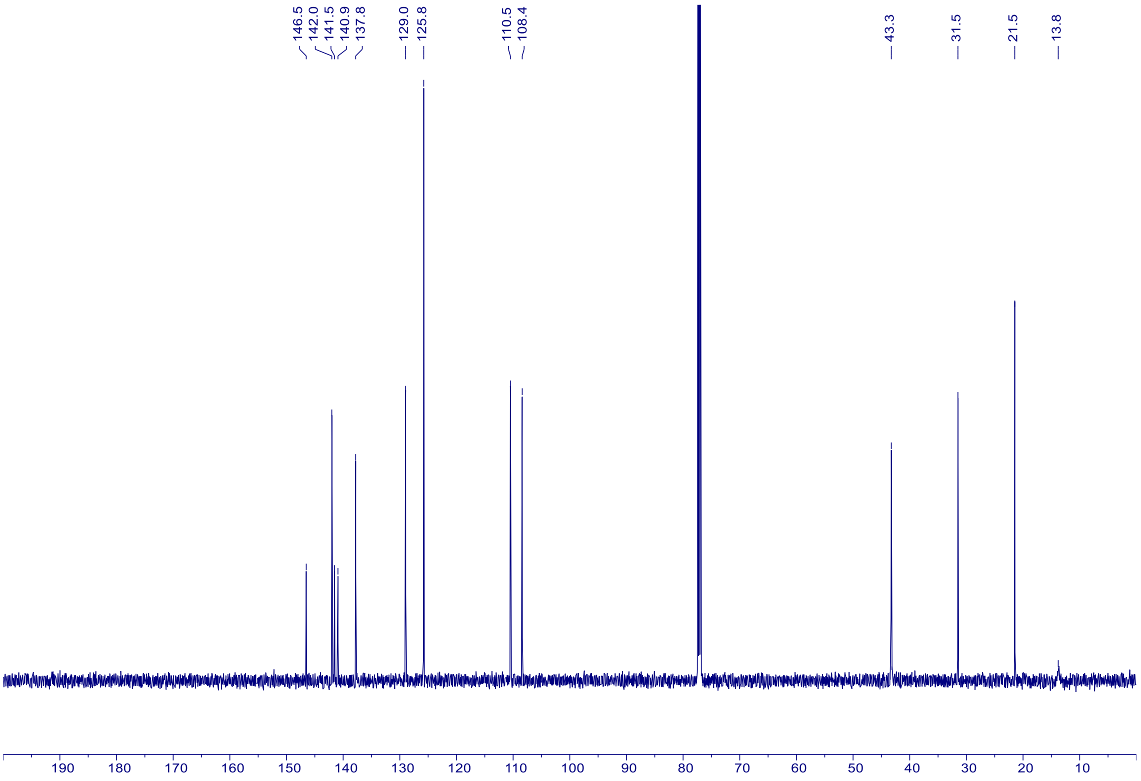

S16 – ^{1}H NMR (600 MHz, $CDCl_3$)

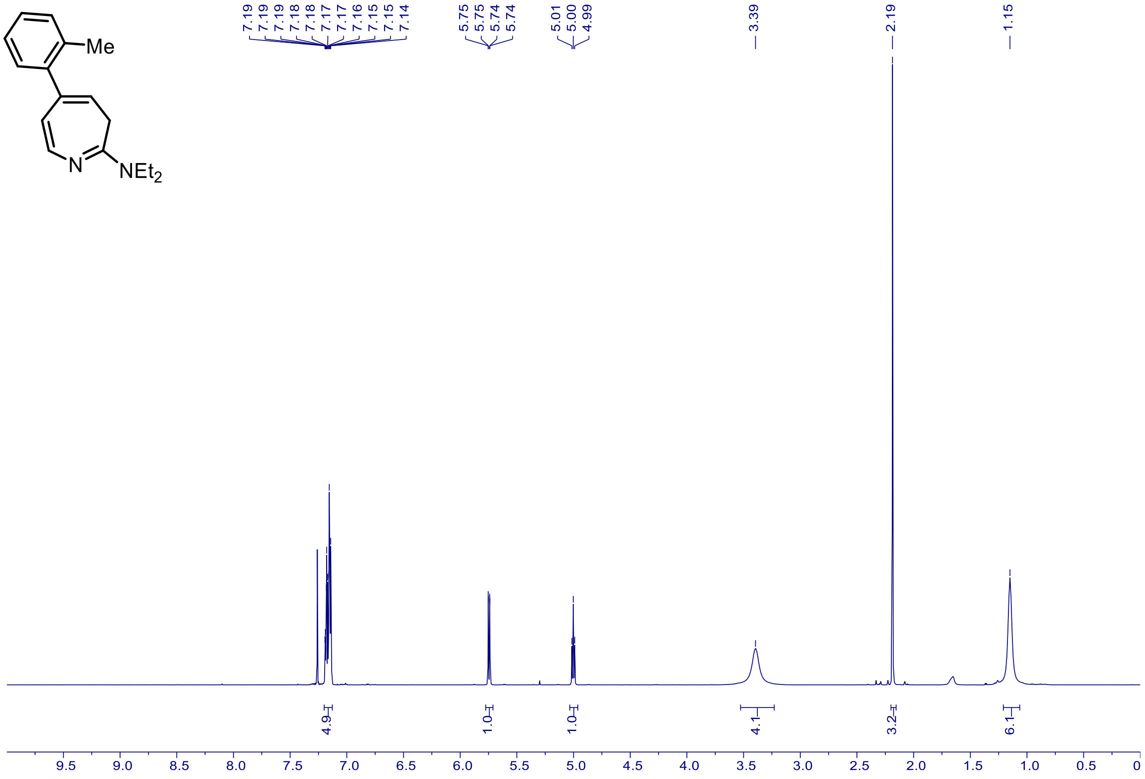

S16 – ^{13}C NMR (151 MHz, $CDCl_3$)

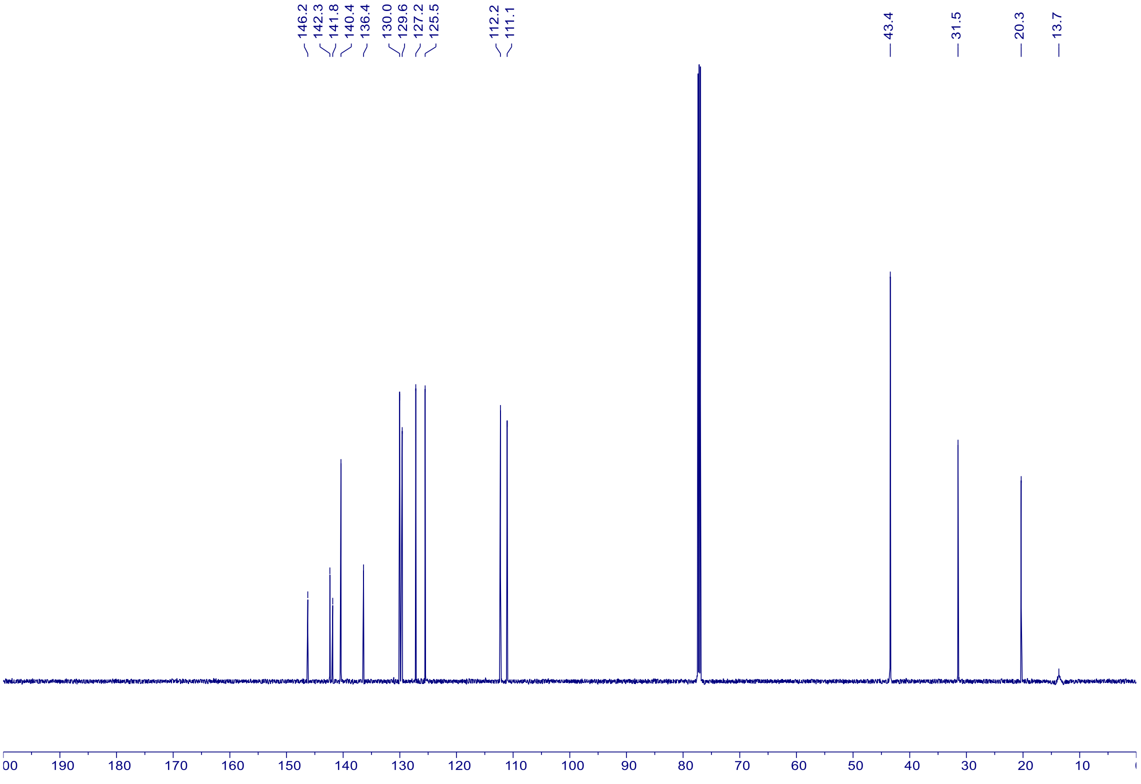

S17 – ¹H NMR (600 MHz, $CDCl_3$)

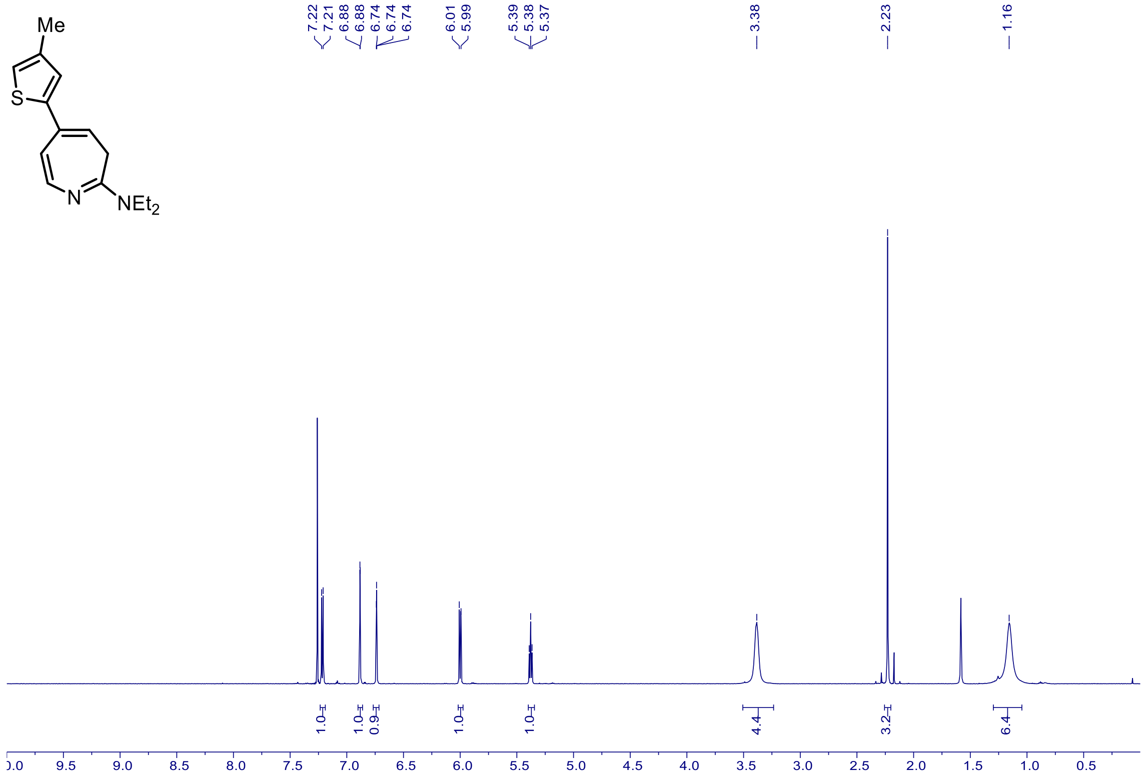

S17 – ¹³C NMR (151 MHz, $CDCl_3$)

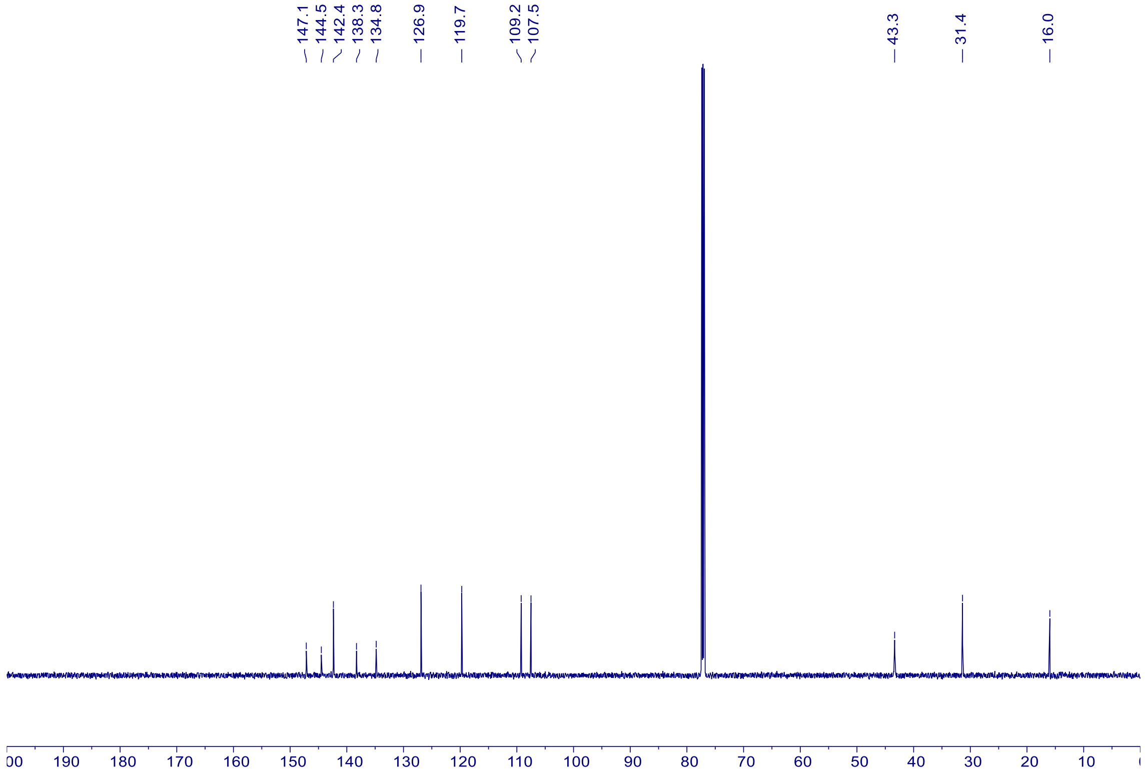

1– ^{1}H NMR (600 MHz, CDCl$_3$)

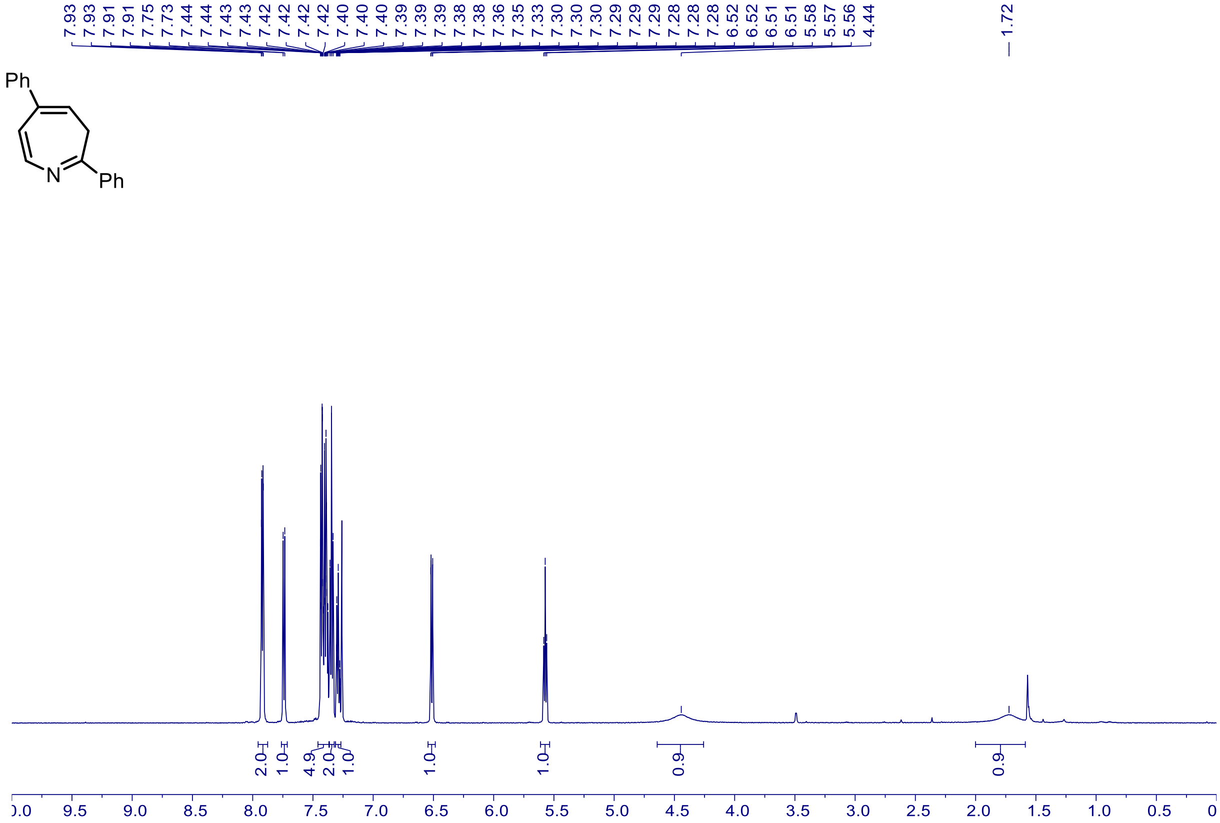

1 – ^{13}C NMR (151 MHz, CDCl$_3$)

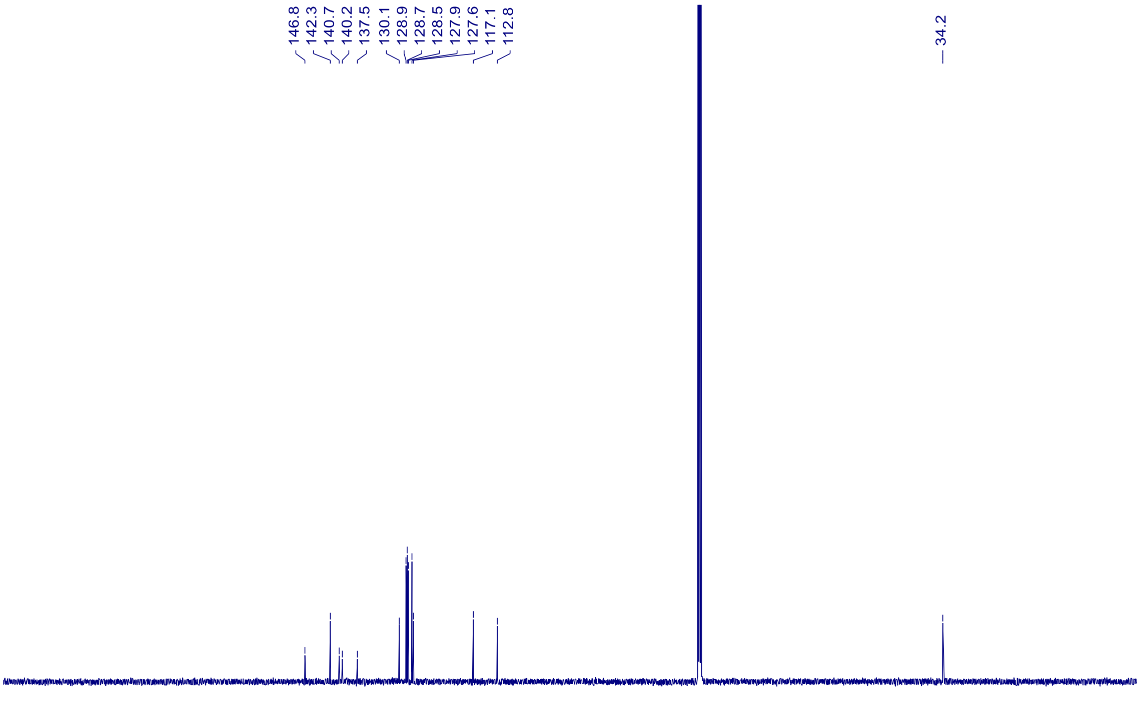

2 – ^{1}H NMR (600 MHz, CDCl$_3$)

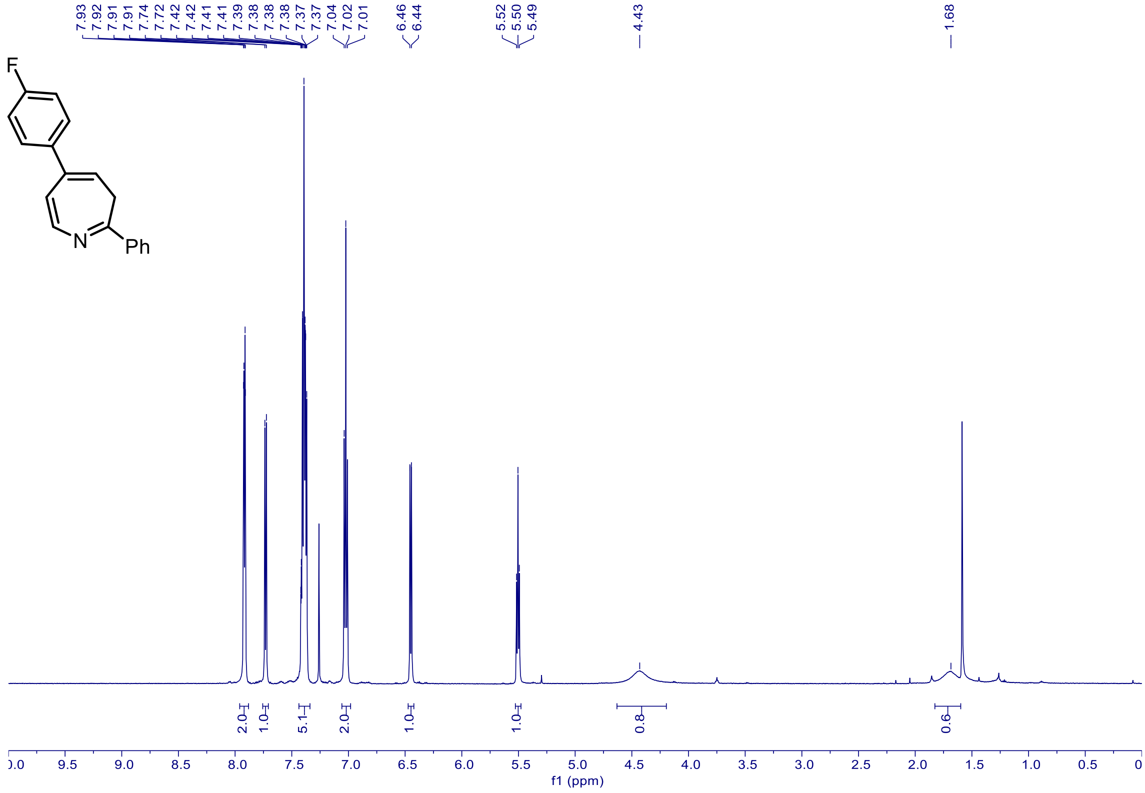

2 – ^{13}C NMR (151 MHz, CDCl$_3$)

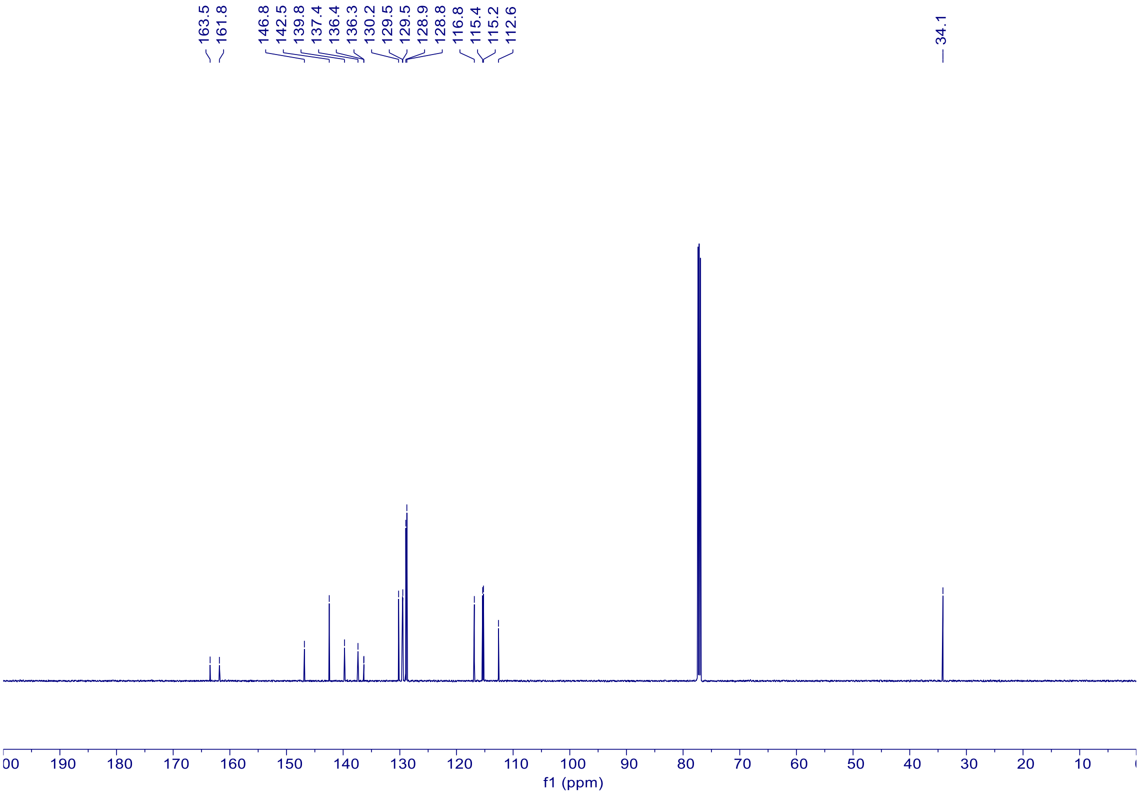

2 – ^{19}F NMR (564 MHz, $CDCl_3$)

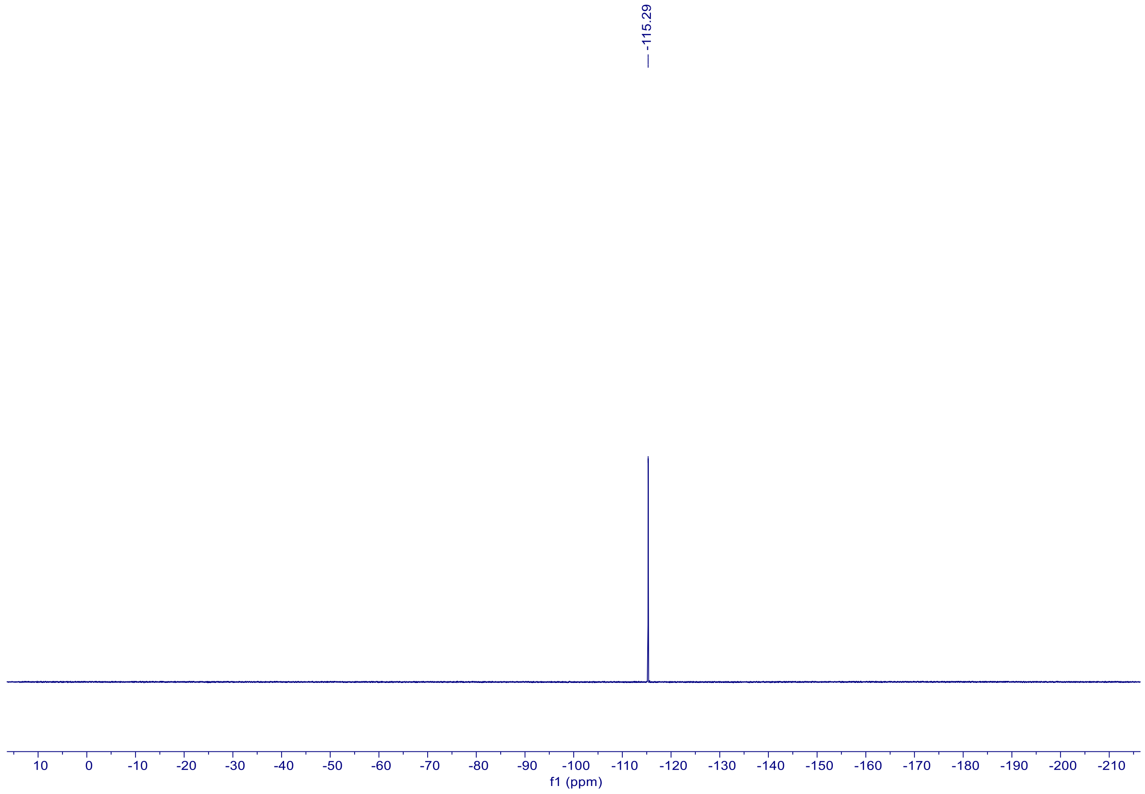

3 – ^{1}H NMR (600 MHz, $CDCl_3$)

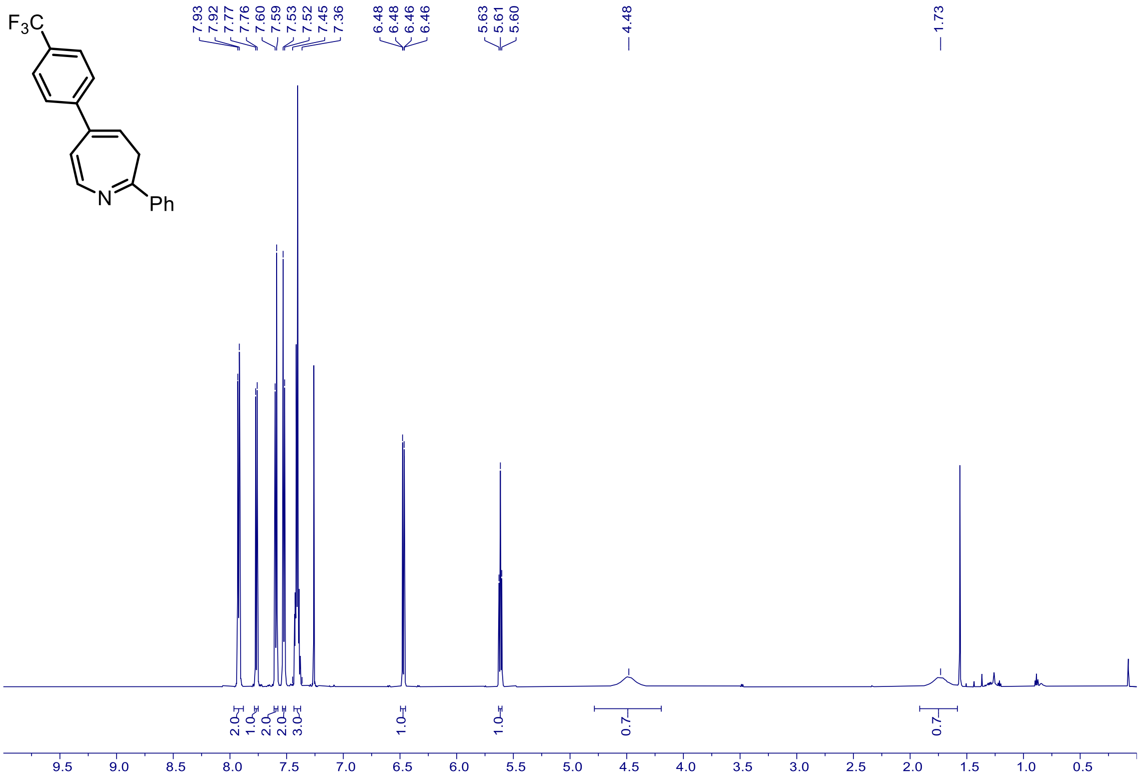

3 – ^{13}C NMR (151 MHz, $CDCl_3$)

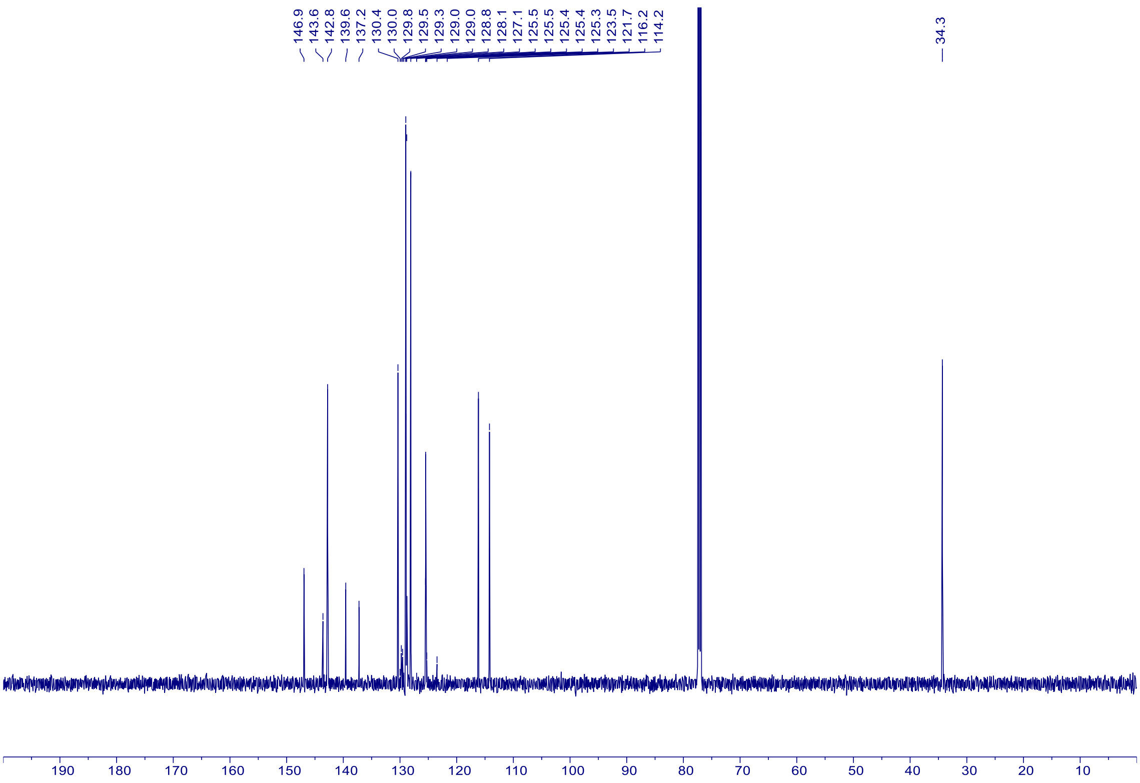

3 – ^{19}F NMR (564 MHz, $CDCl_3$)

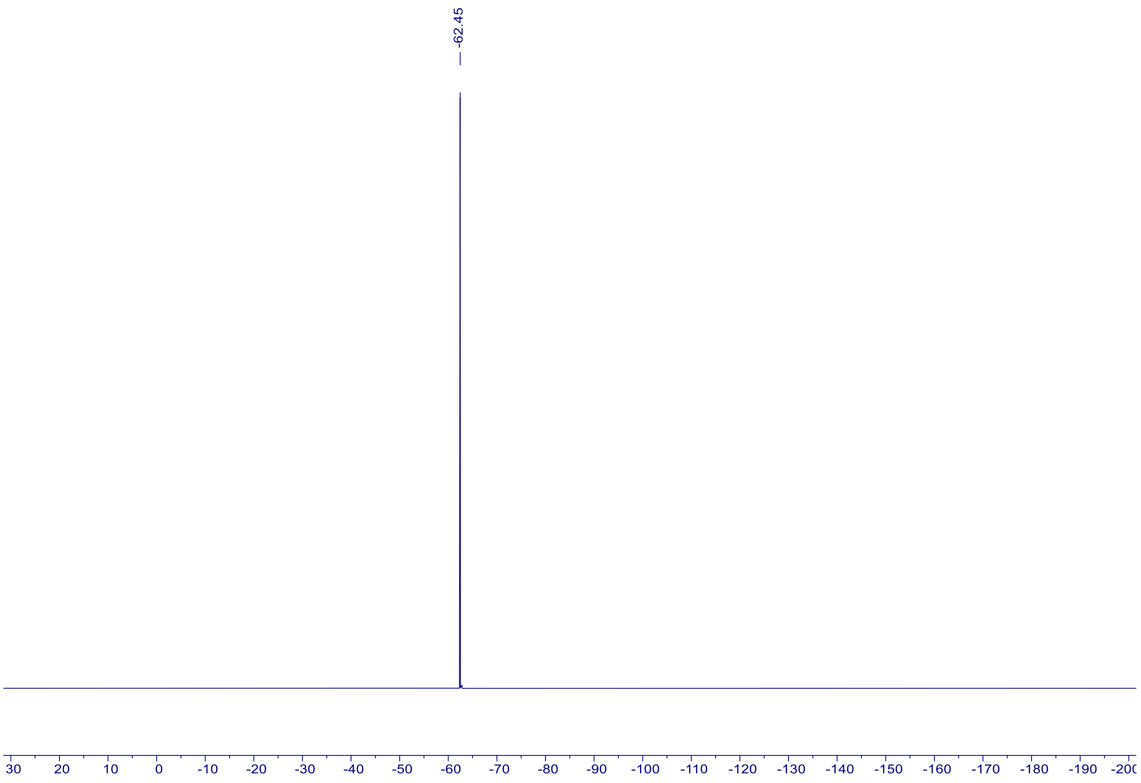

4 – ^{1}H NMR (600 MHz, CDCl$_3$)

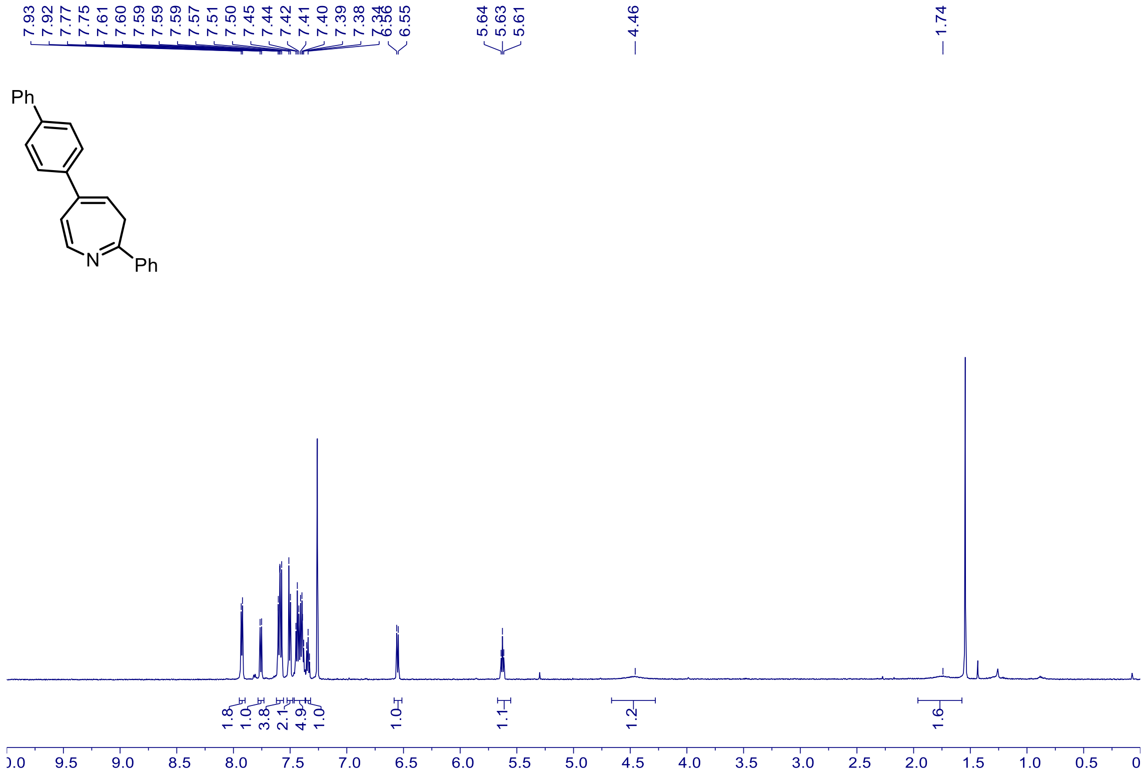

4 – ^{13}C NMR (151 MHz, CDCl$_3$)

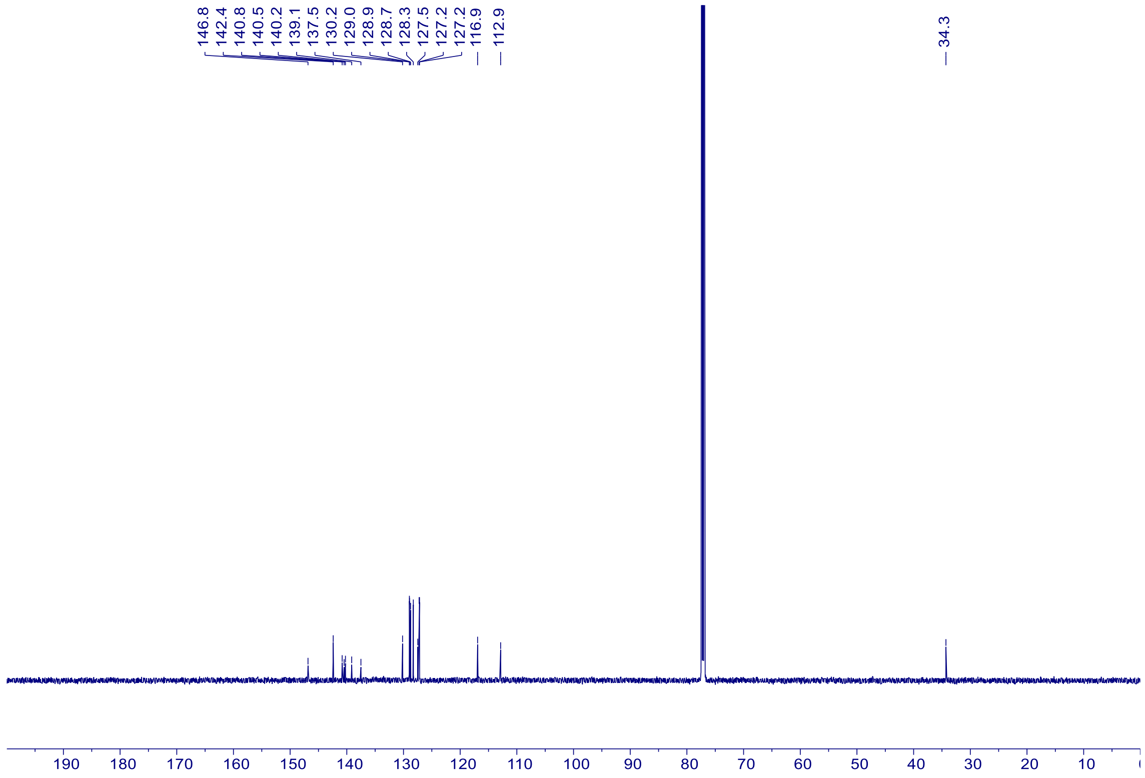

5 – ^{1}H NMR (600 MHz, $CDCl_3$)

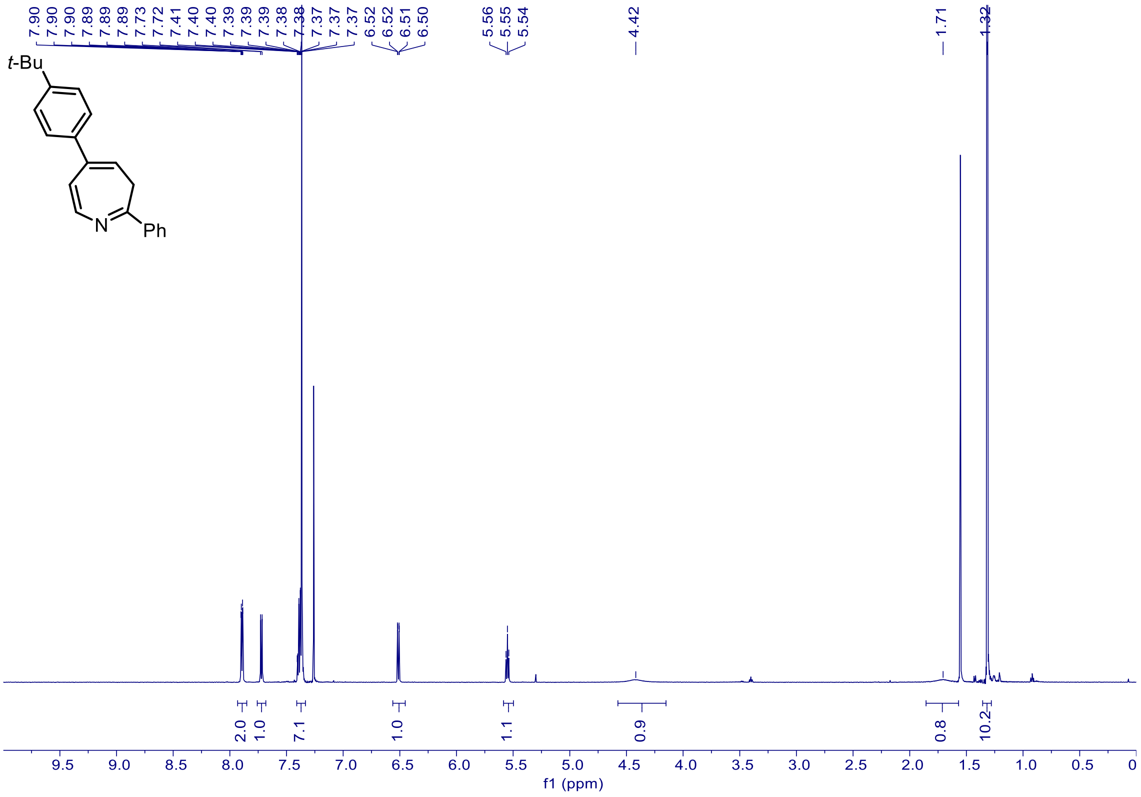

5 – ^{13}C NMR (151 MHz, $CDCl_3$)

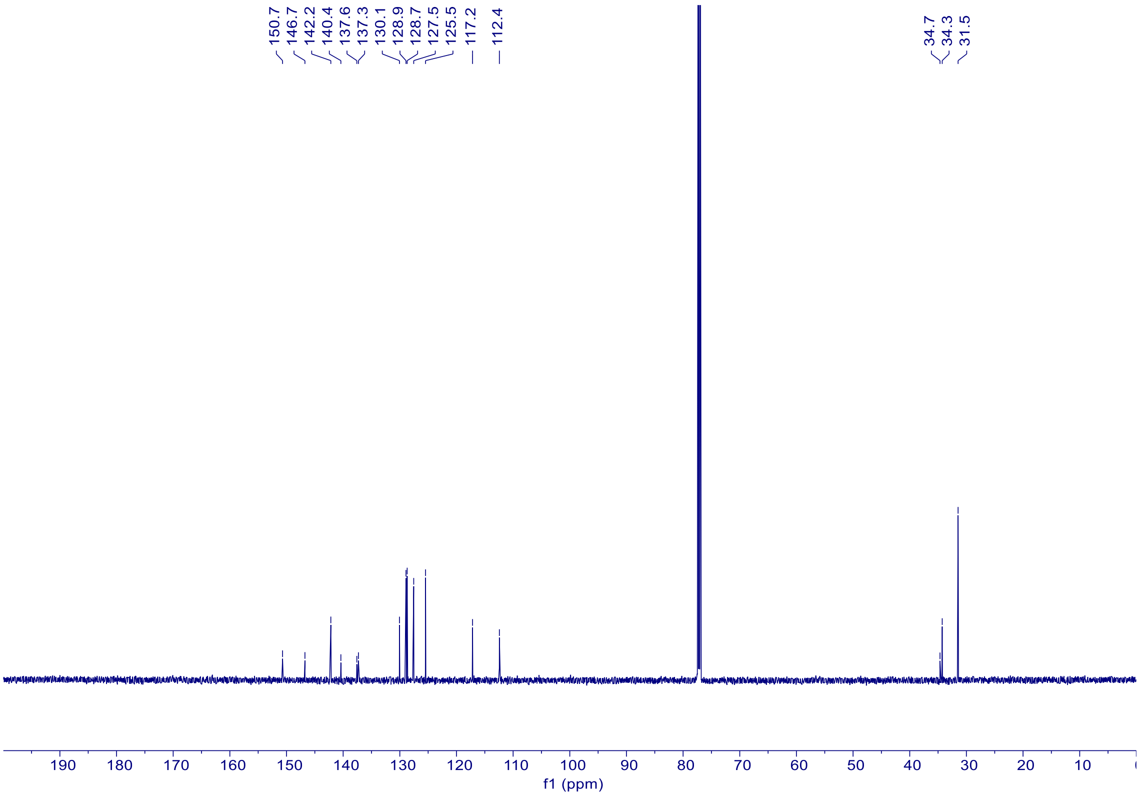

6 – ^{1}H NMR (600 MHz, $CDCl_3$)

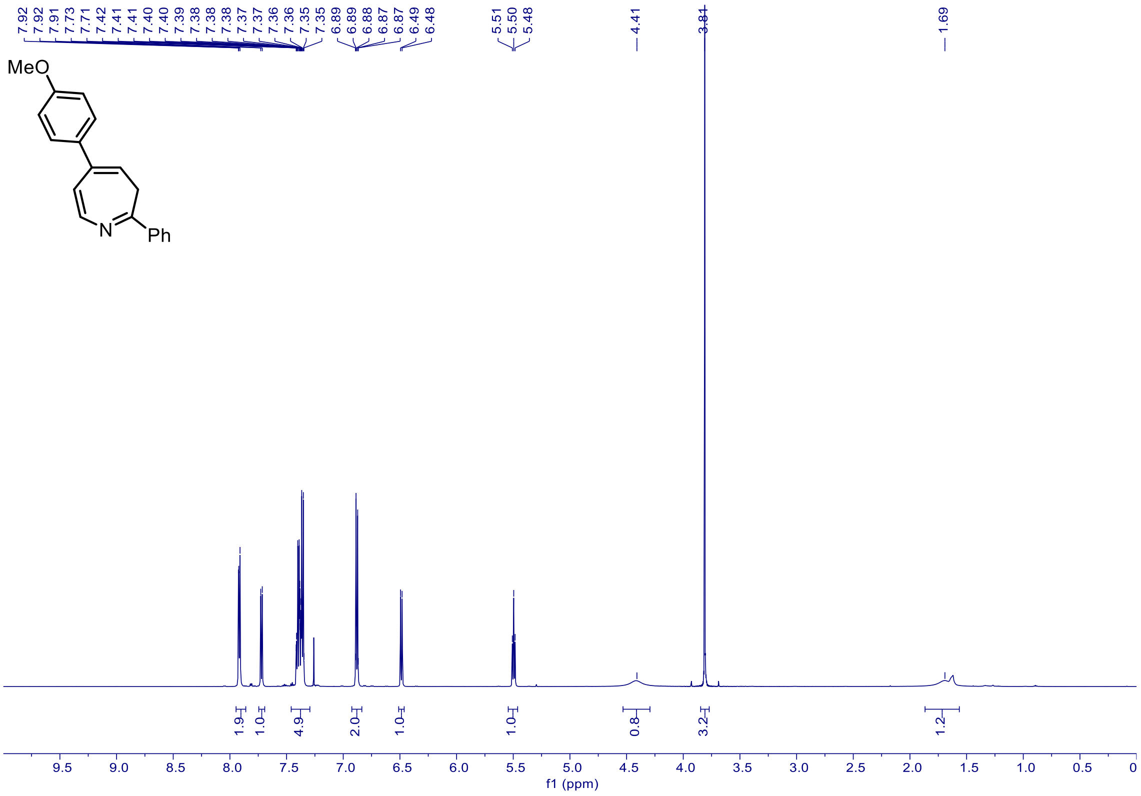

6 – ^{13}C NMR (151 MHz, $CDCl_3$)

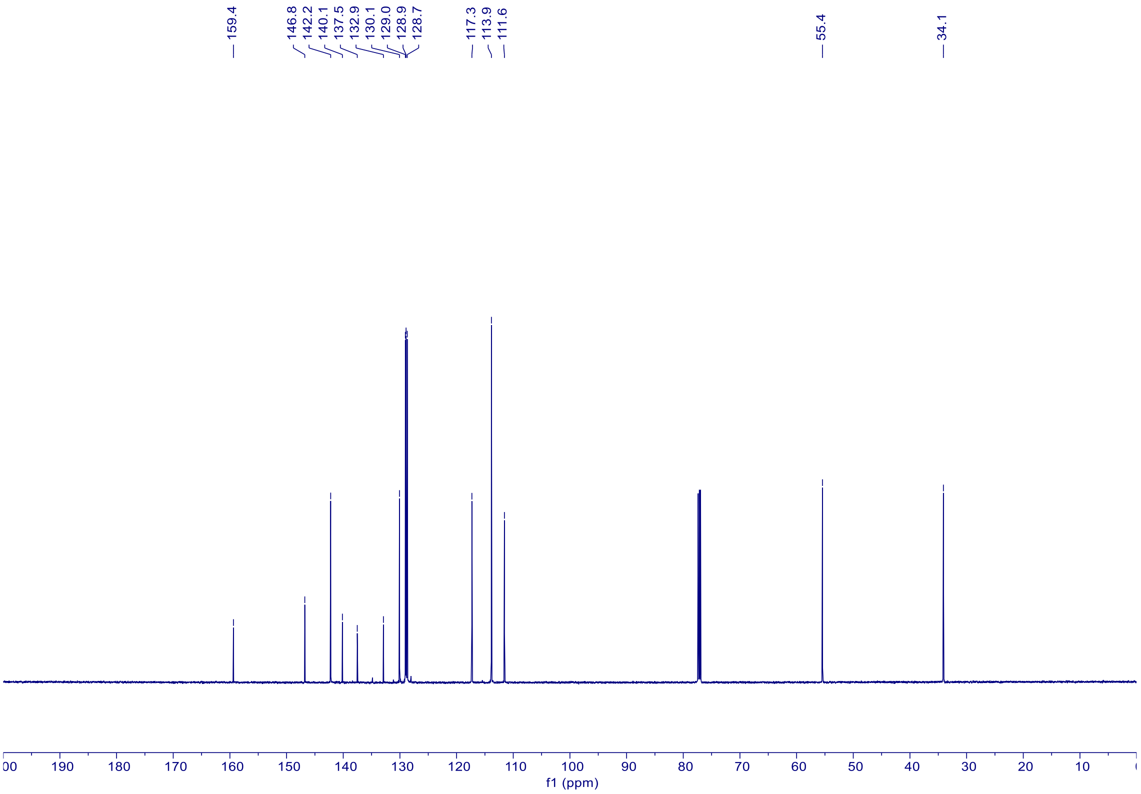

7 – ^{1}H NMR (600 MHz, $CDCl_3$)

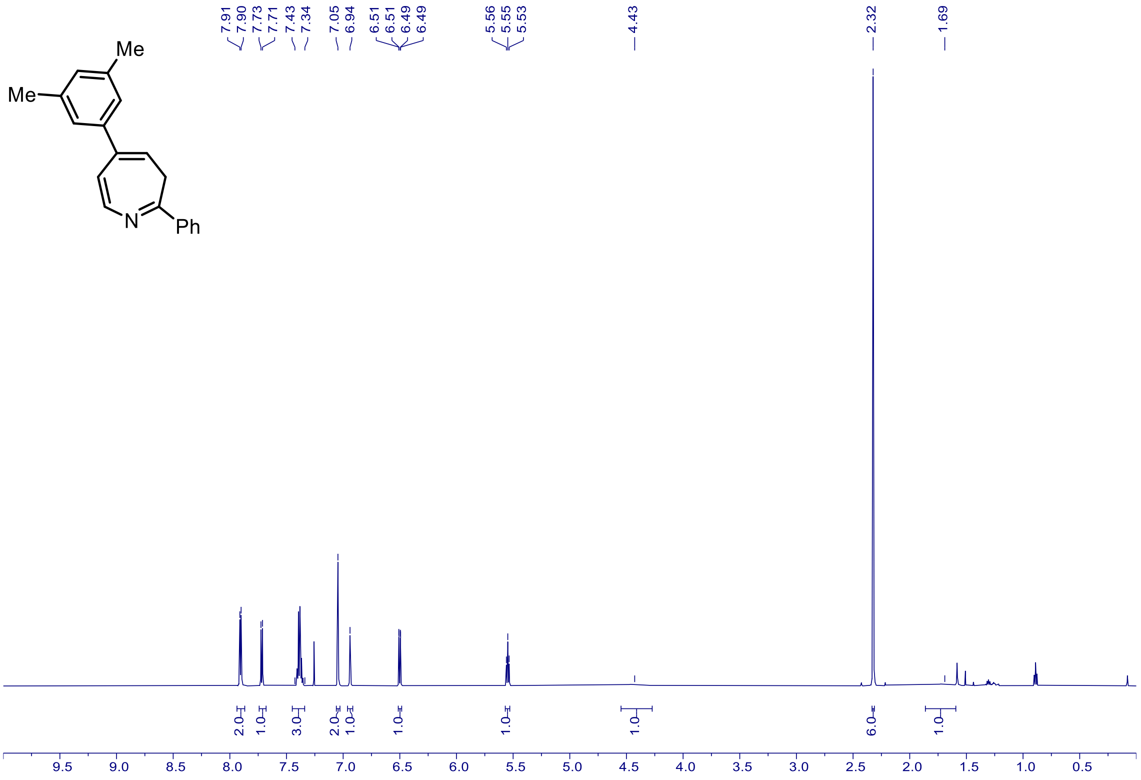

7 – ^{13}C NMR (151 MHz, $CDCl_3$)

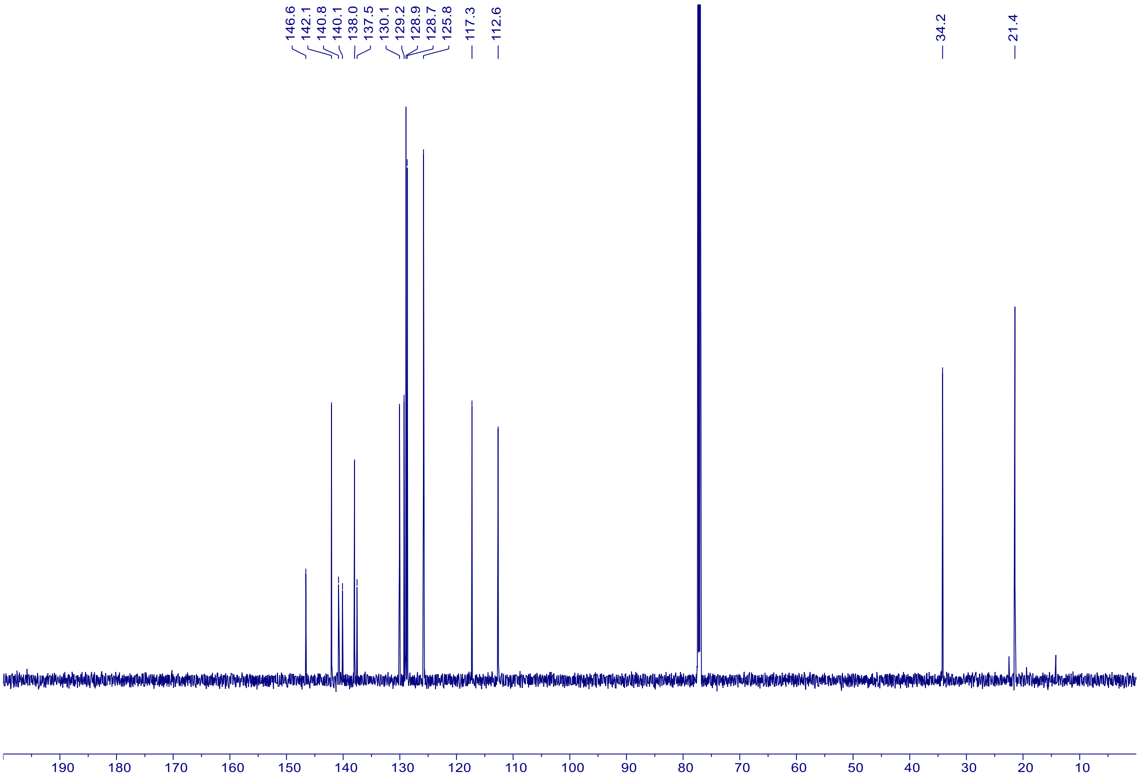

8 – ^{1}H NMR (600 MHz, $CDCl_3$)

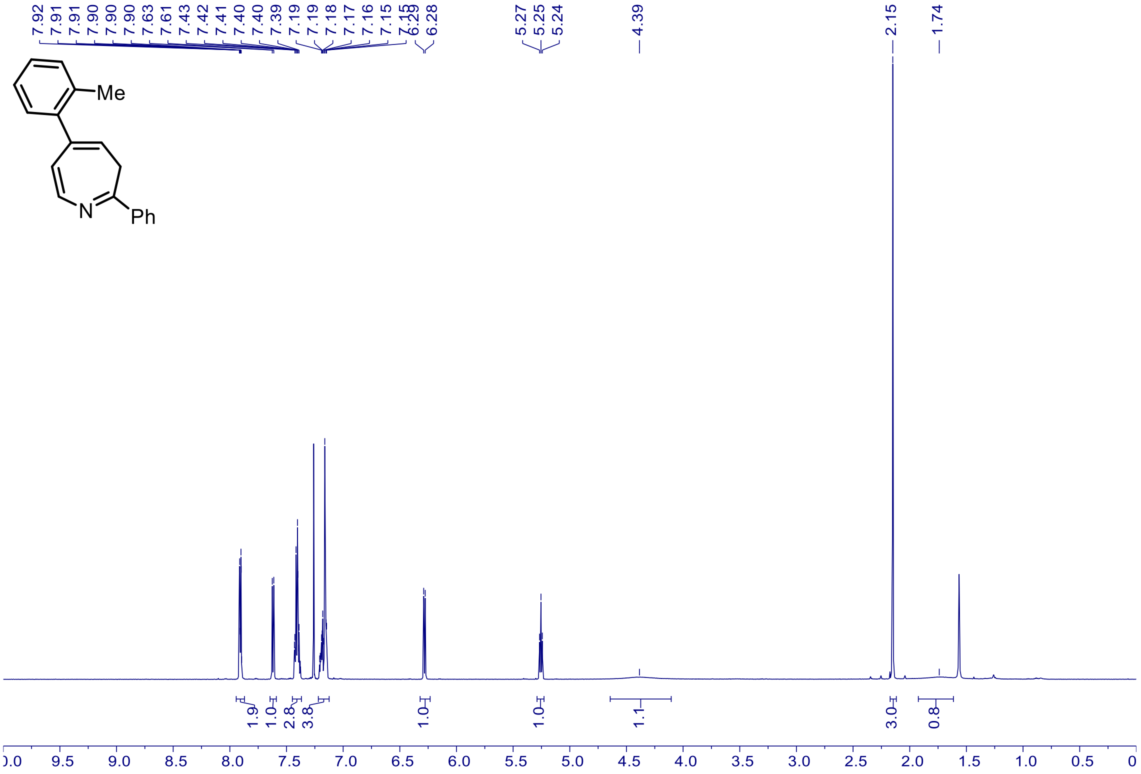

8 – ^{13}C NMR (151 MHz, $CDCl_3$)

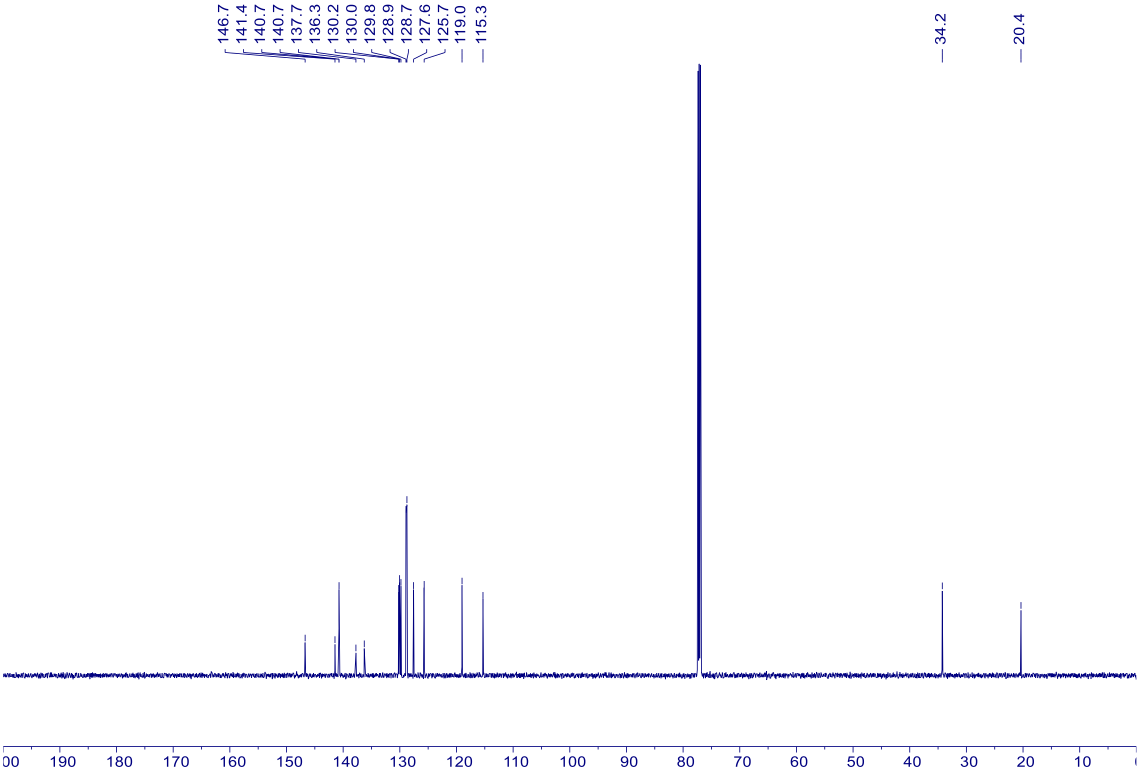

9 – ^{1}H NMR (600 MHz, CDCl$_3$)

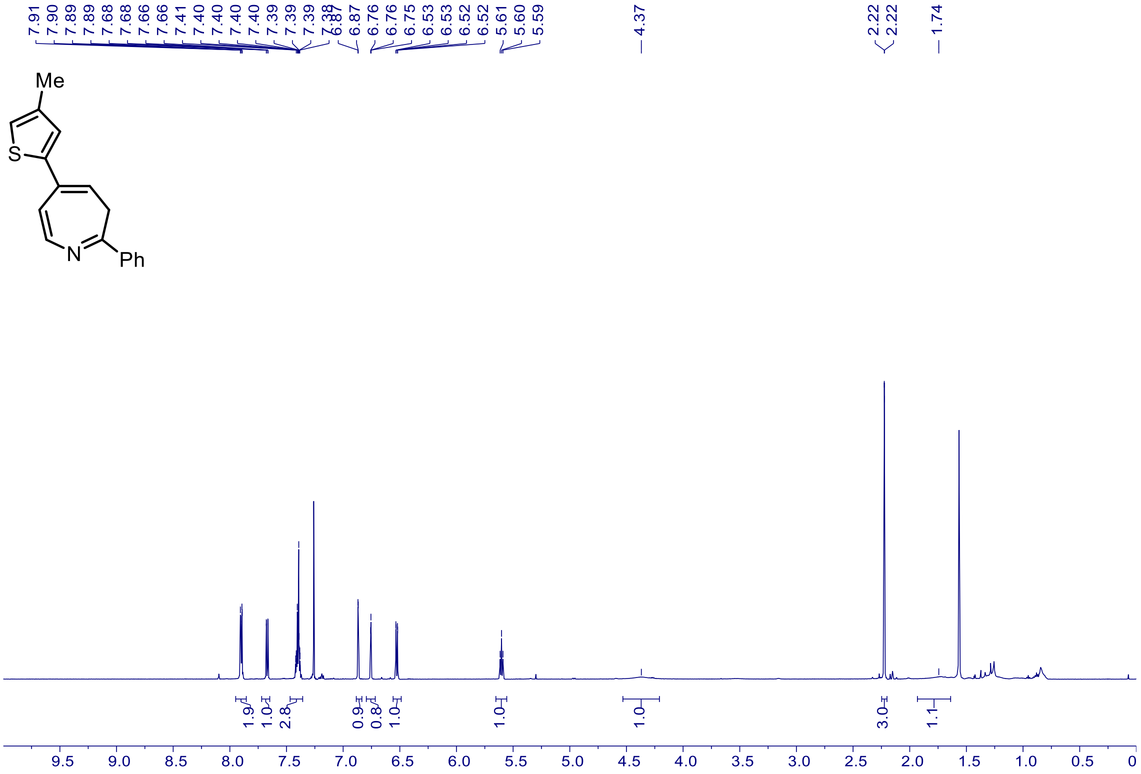

9 – ^{1}H NMR (600 MHz, CDCl$_3$)

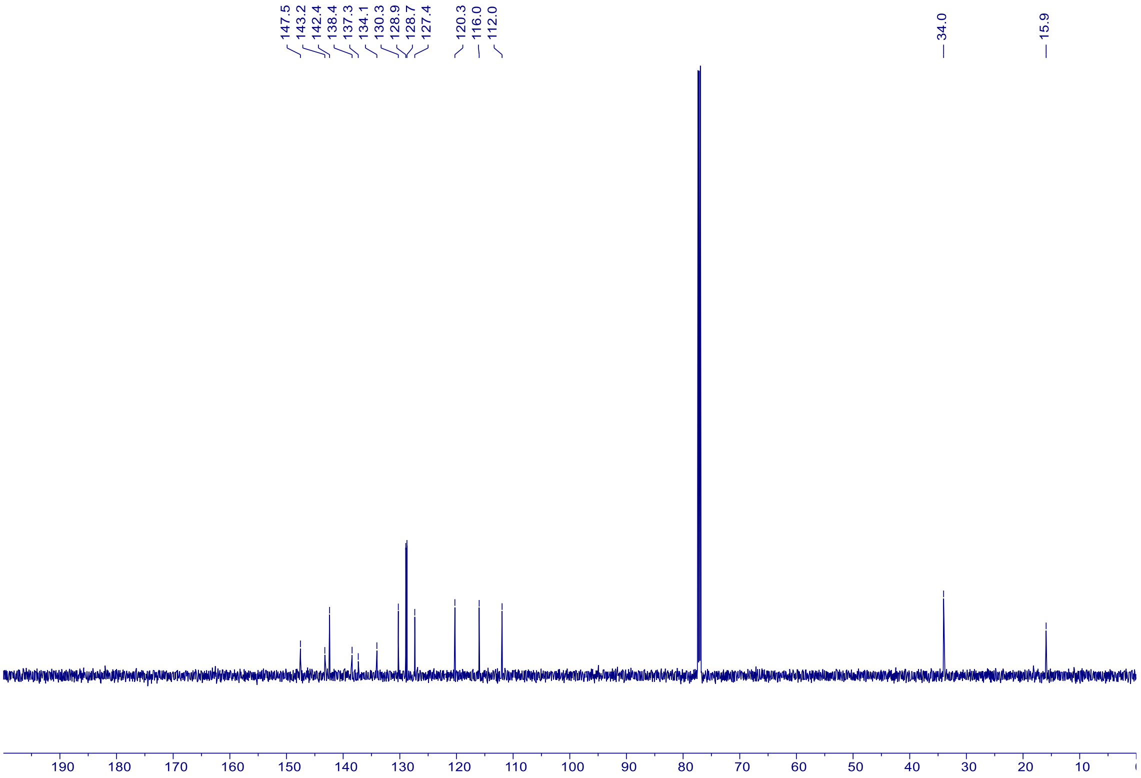

10 – ^{1}H NMR (400 MHz, CDCl$_3$)

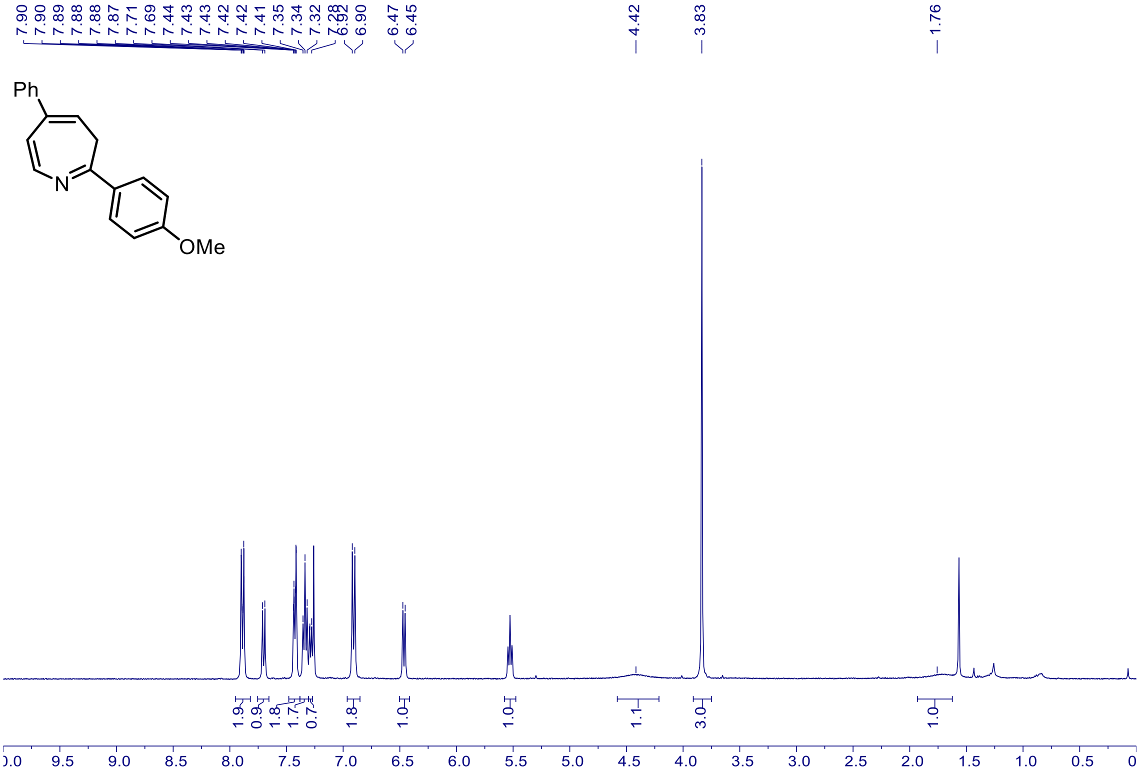

10 – ^{13}C NMR (151 MHz, CDCl$_3$)

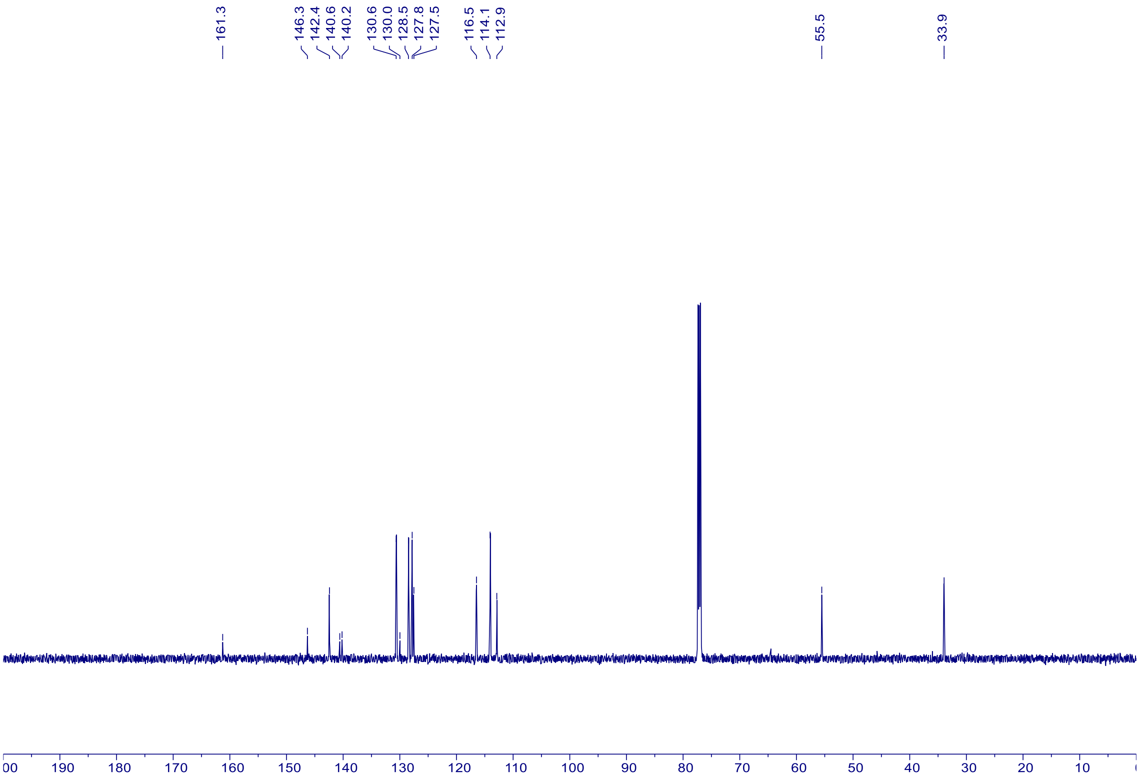

11 – ^{1}H NMR (600 MHz, $CDCl_3$)

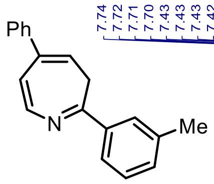

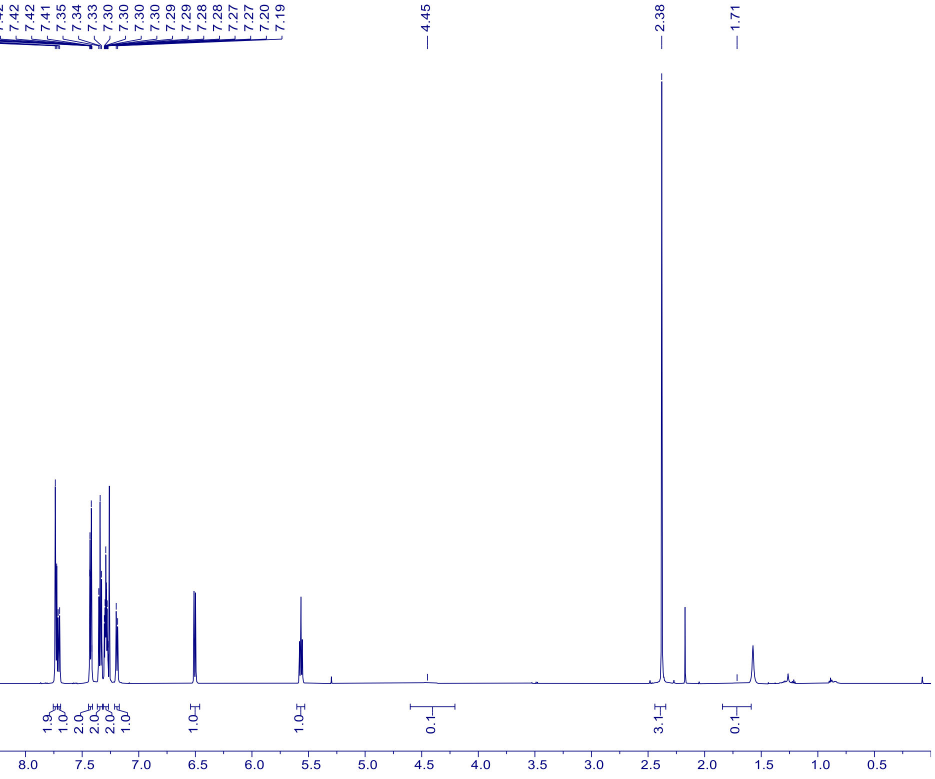

11 – ^{13}C NMR (151 MHz, $CDCl_3$)

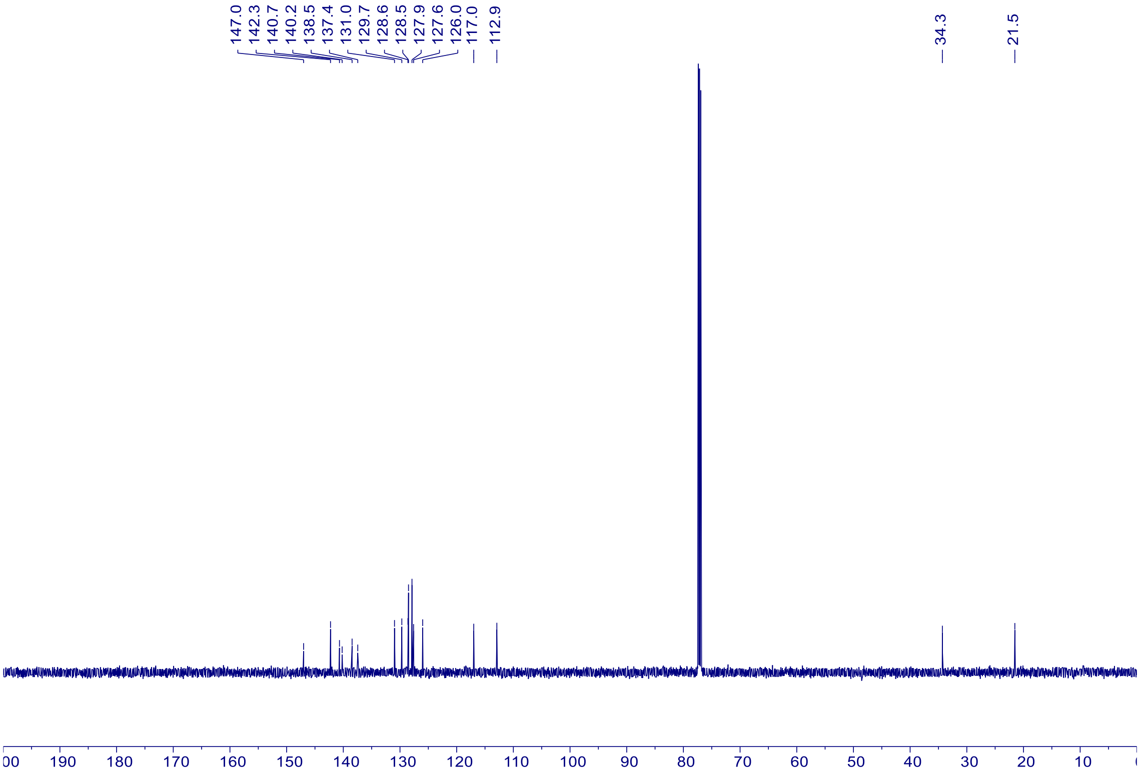

12 – ^{1}H NMR (600 MHz, $CDCl_3$)

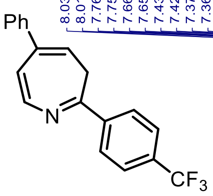

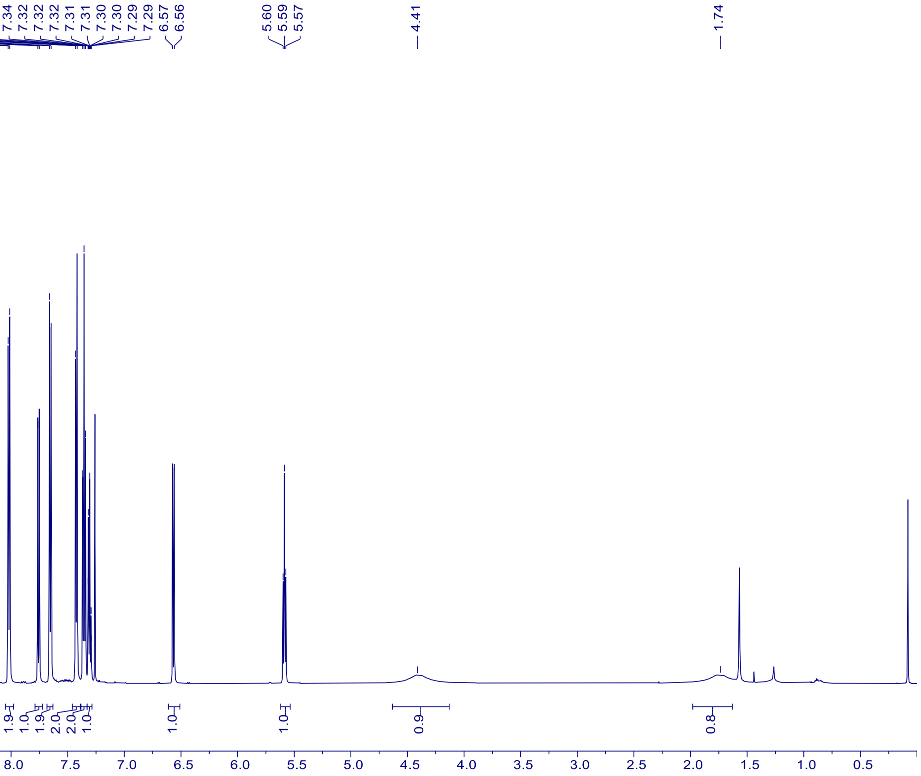

12 – ^{13}C NMR (151 MHz, $CDCl_3$)

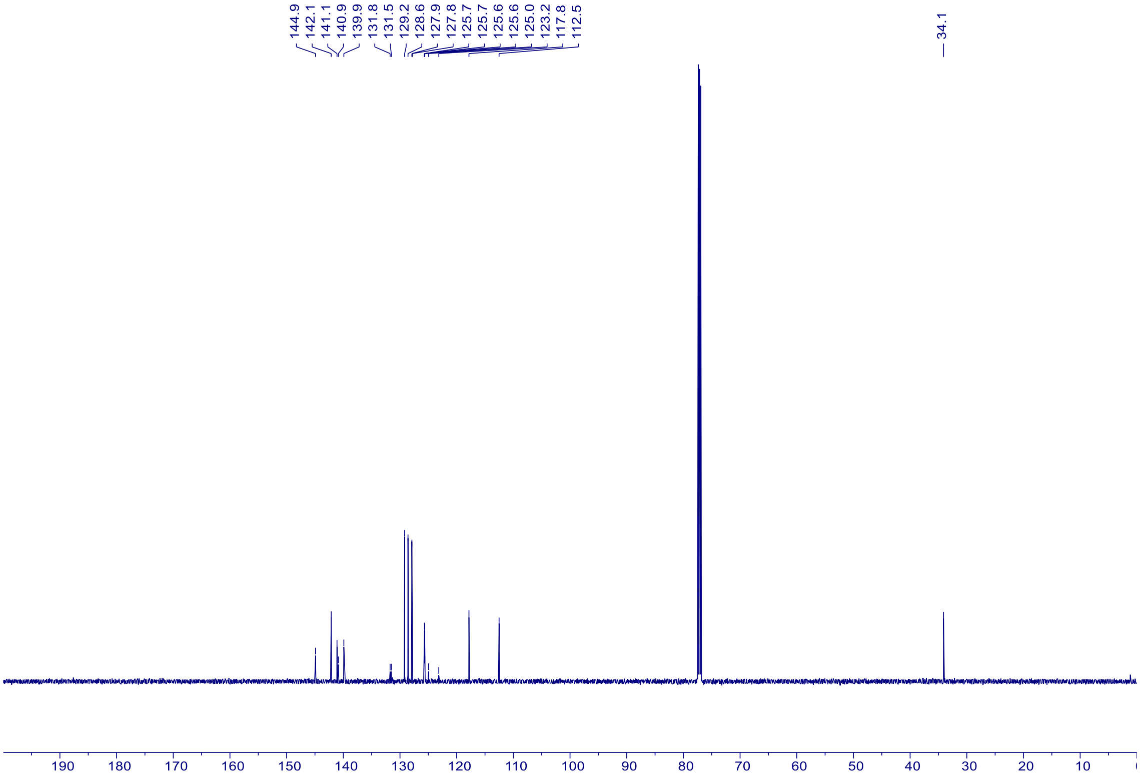

12 – ^{19}F NMR (565 MHz, $CDCl_3$)

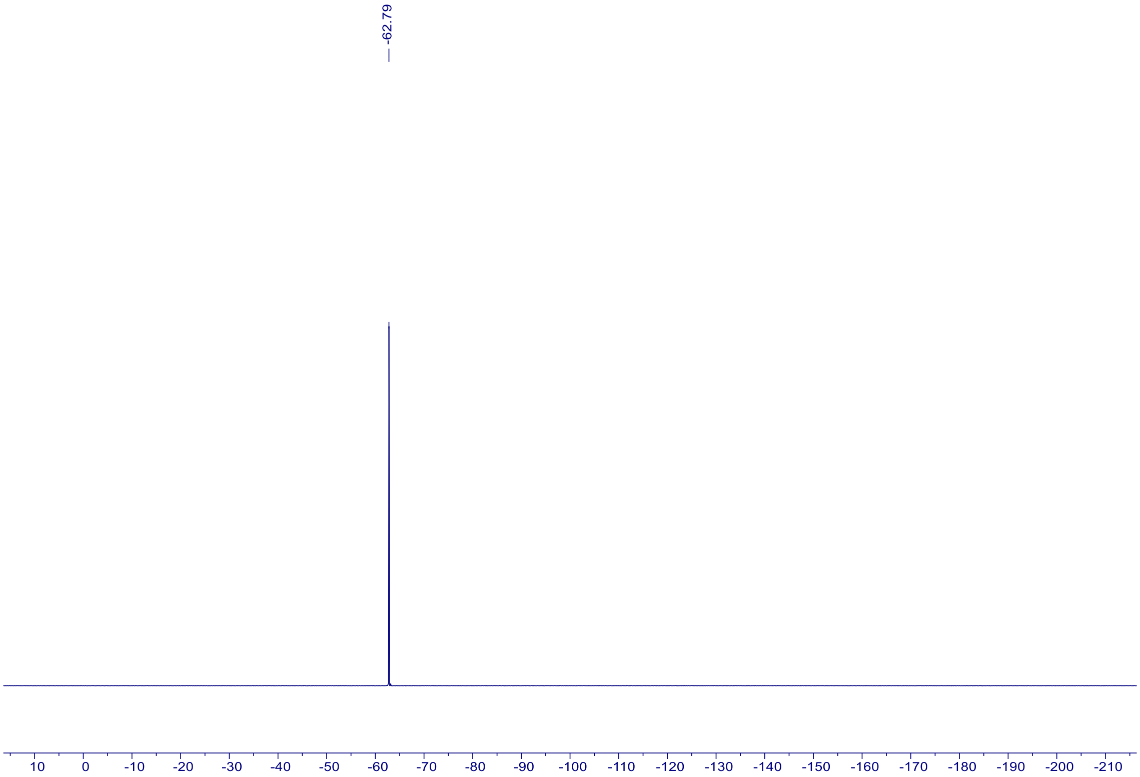

13 – ^{1}H NMR (400 MHz, CDCl$_3$)

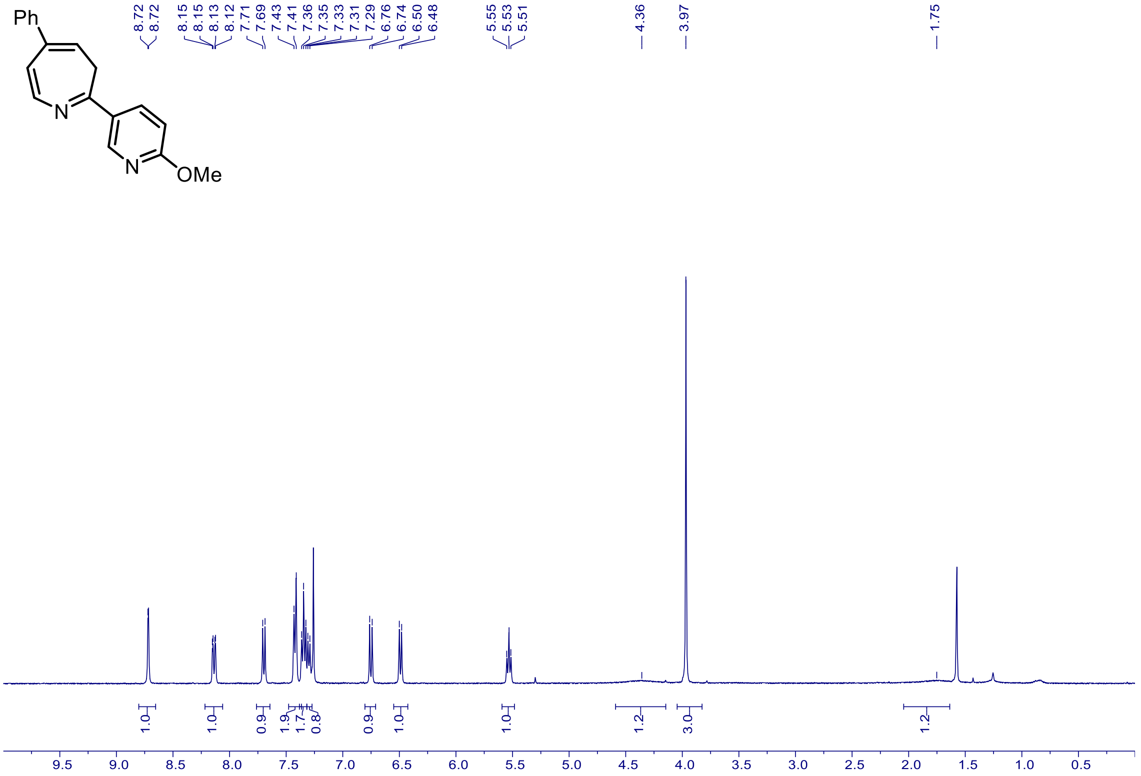

13 – ^{13}C NMR (151 MHz, CDCl$_3$)

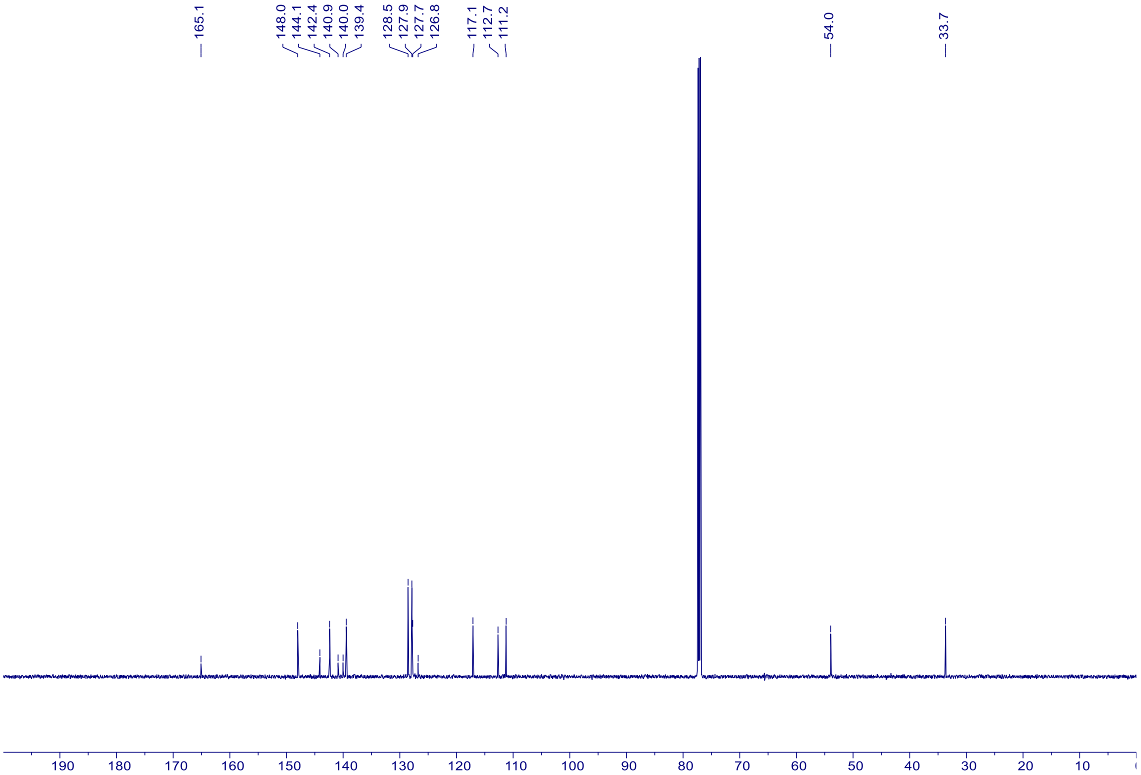

14 – ^{1}H NMR (600 MHz, $CDCl_3$)

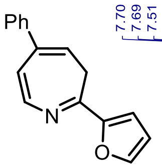

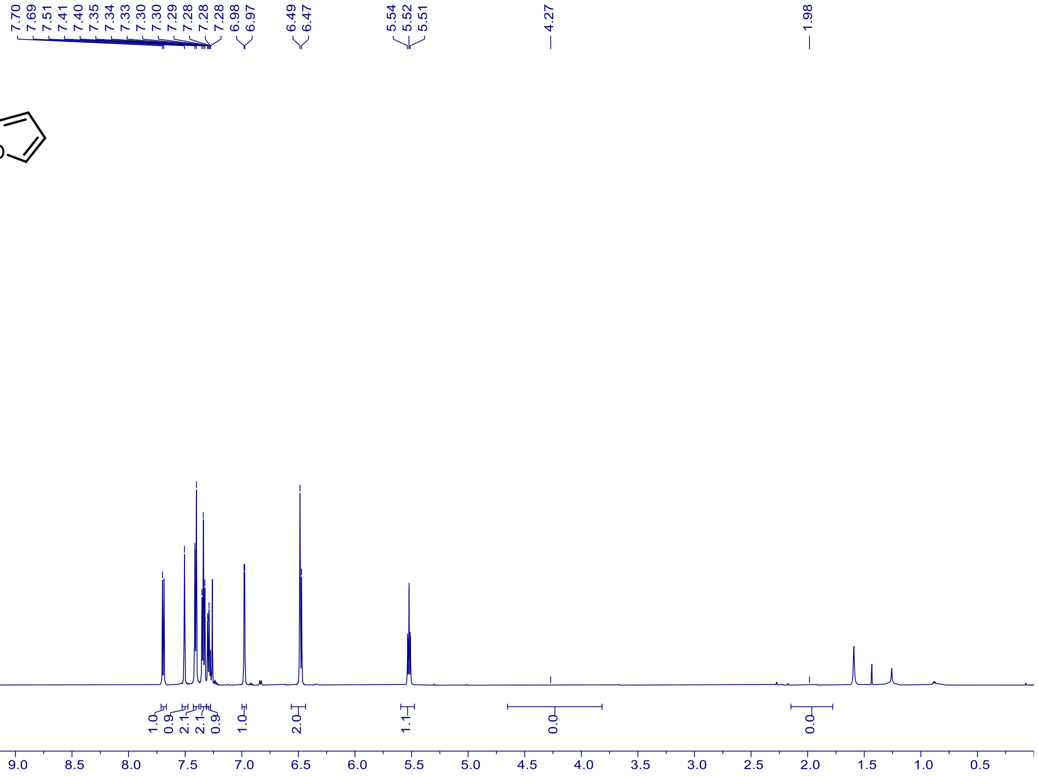

14 – ^{13}C NMR (151 MHz, $CDCl_3$)

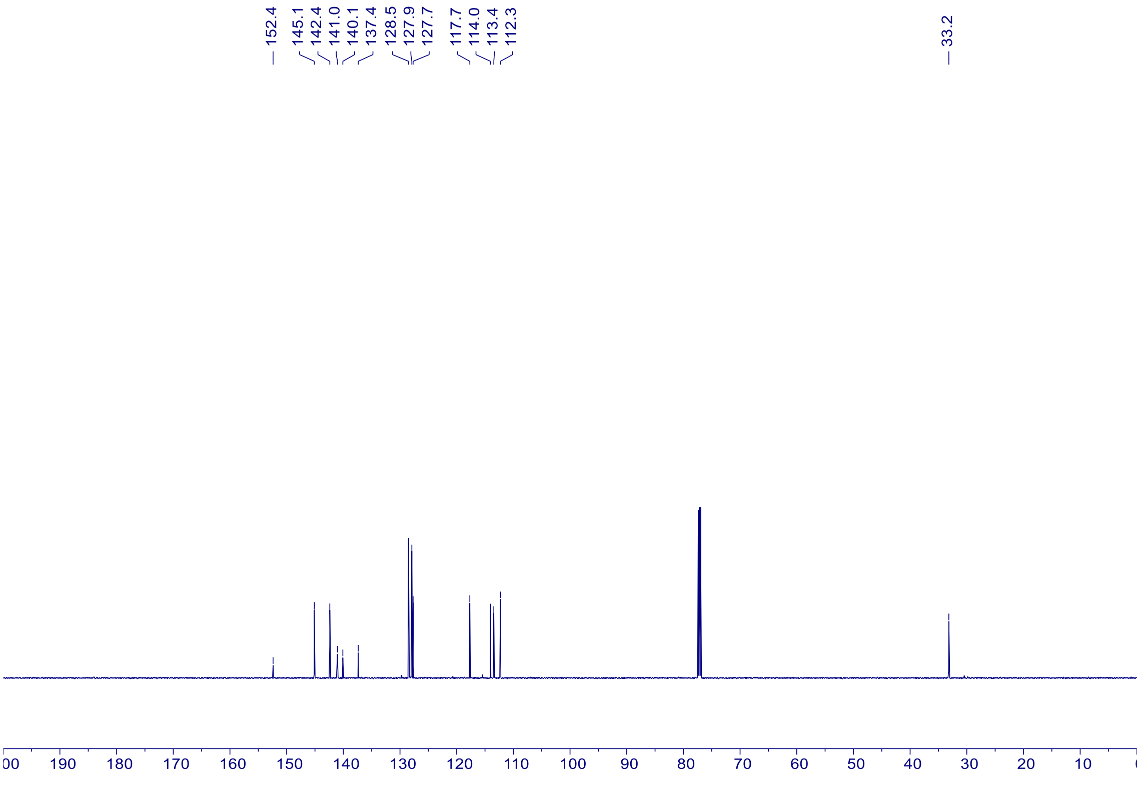

15 – ^{1}H NMR (600 MHz, $CDCl_3$)

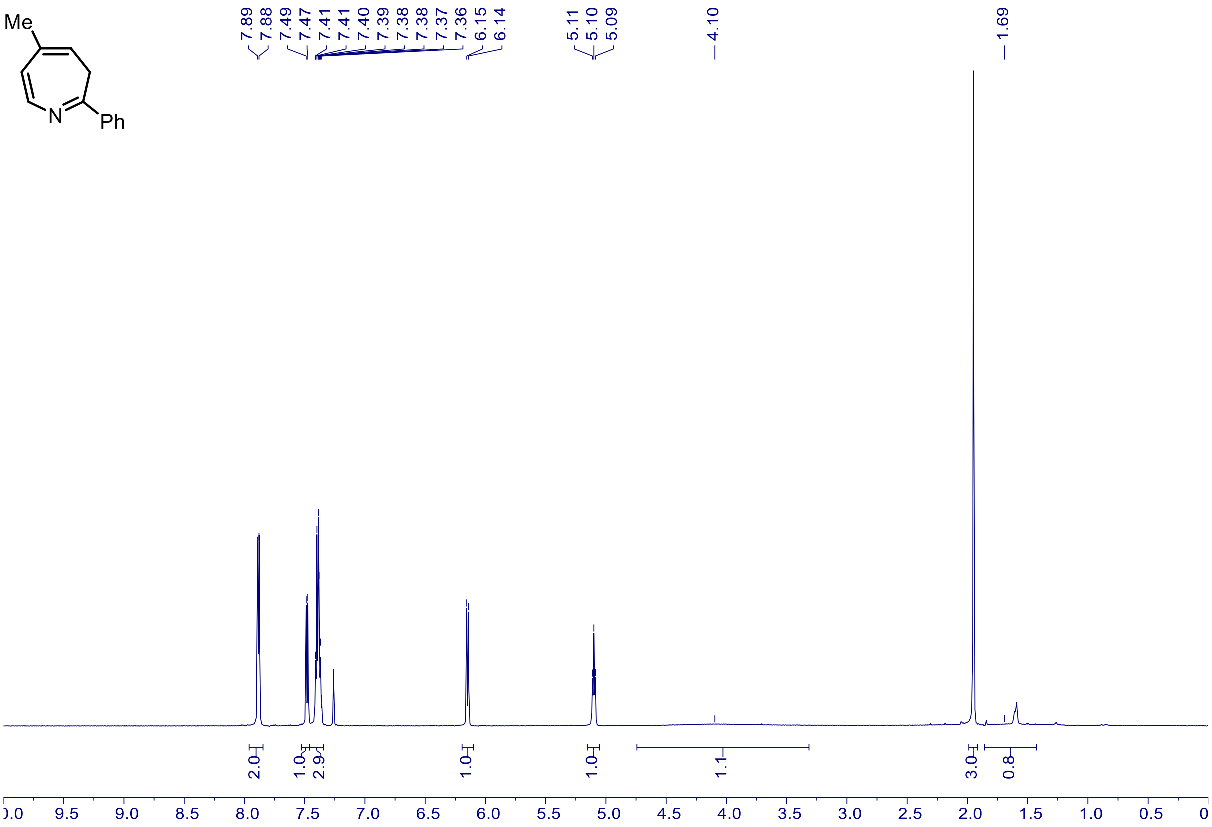

15 – ^{13}C NMR (151 MHz, $CDCl_3$)

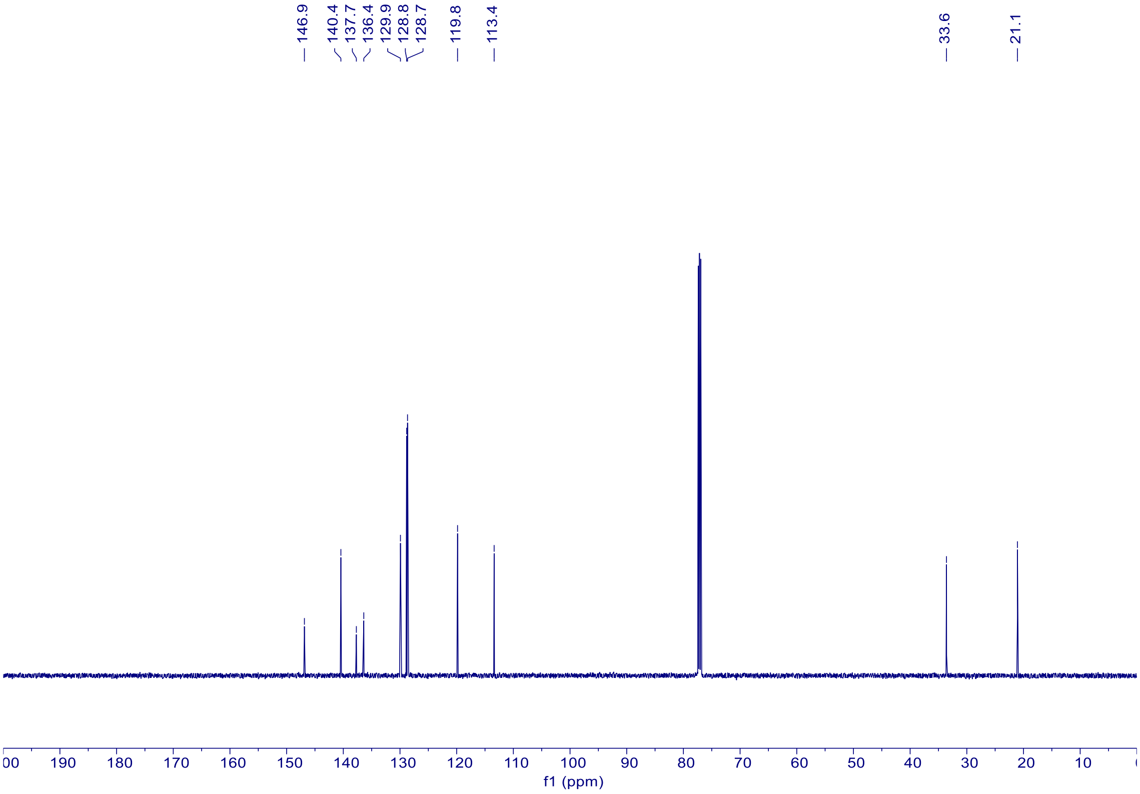

16 – ^{1}H NMR (600 MHz, $CDCl_3$)

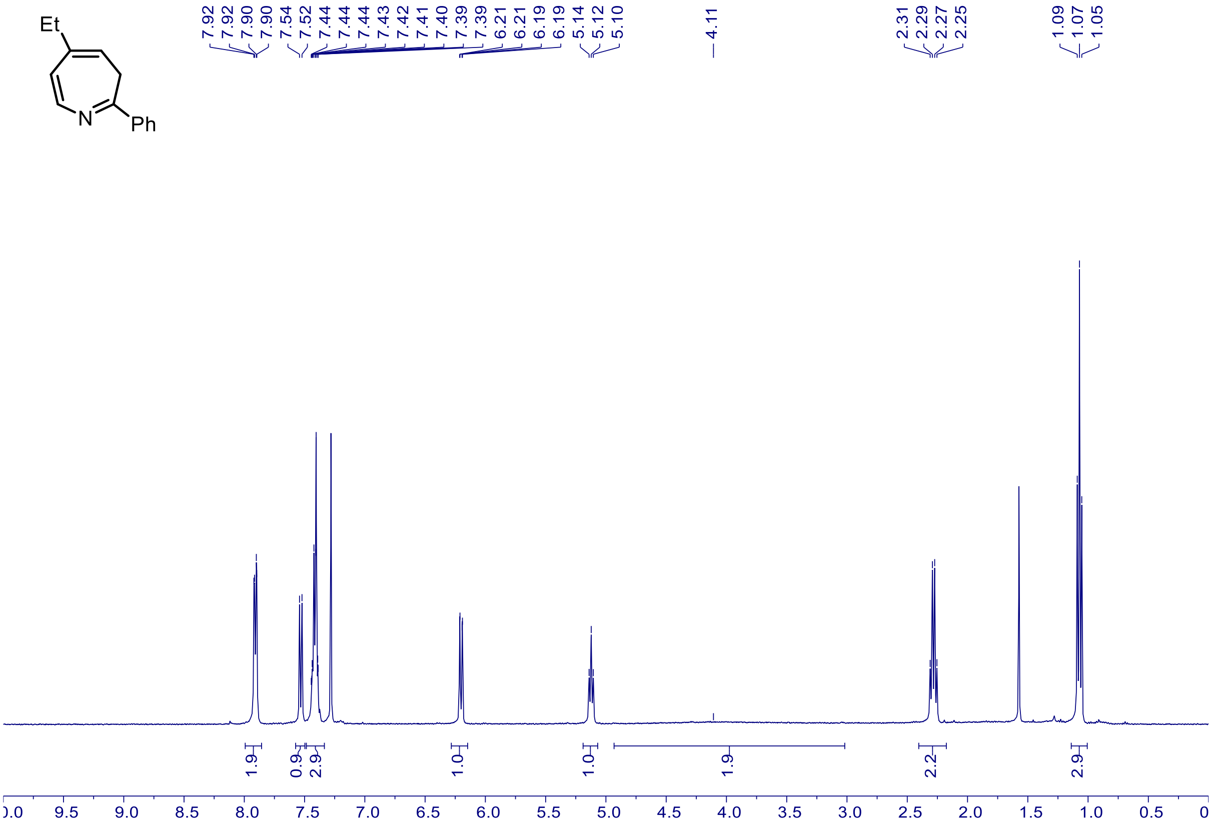

16 – ^{13}C NMR (101 MHz, $CDCl_3$)

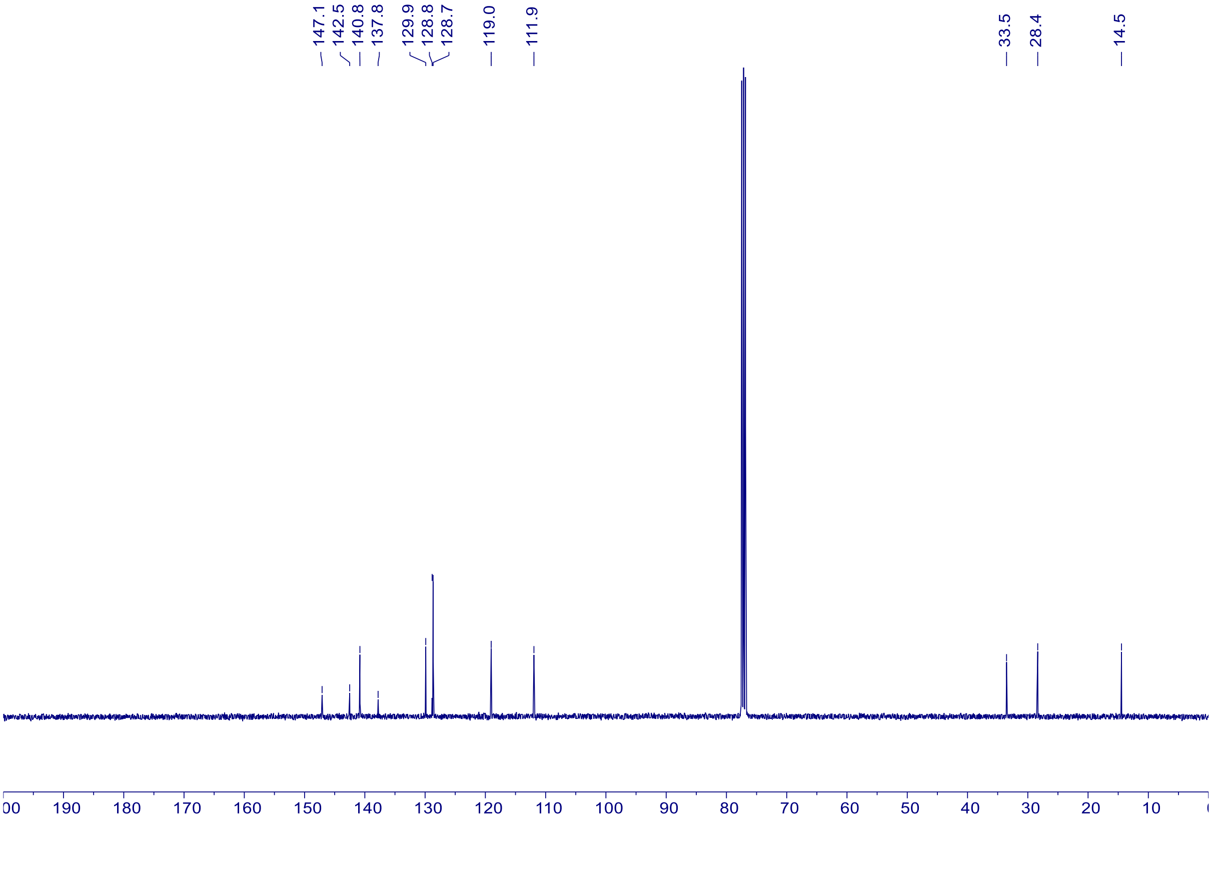

17 – ^{1}H NMR (600 MHz, CDCl3)

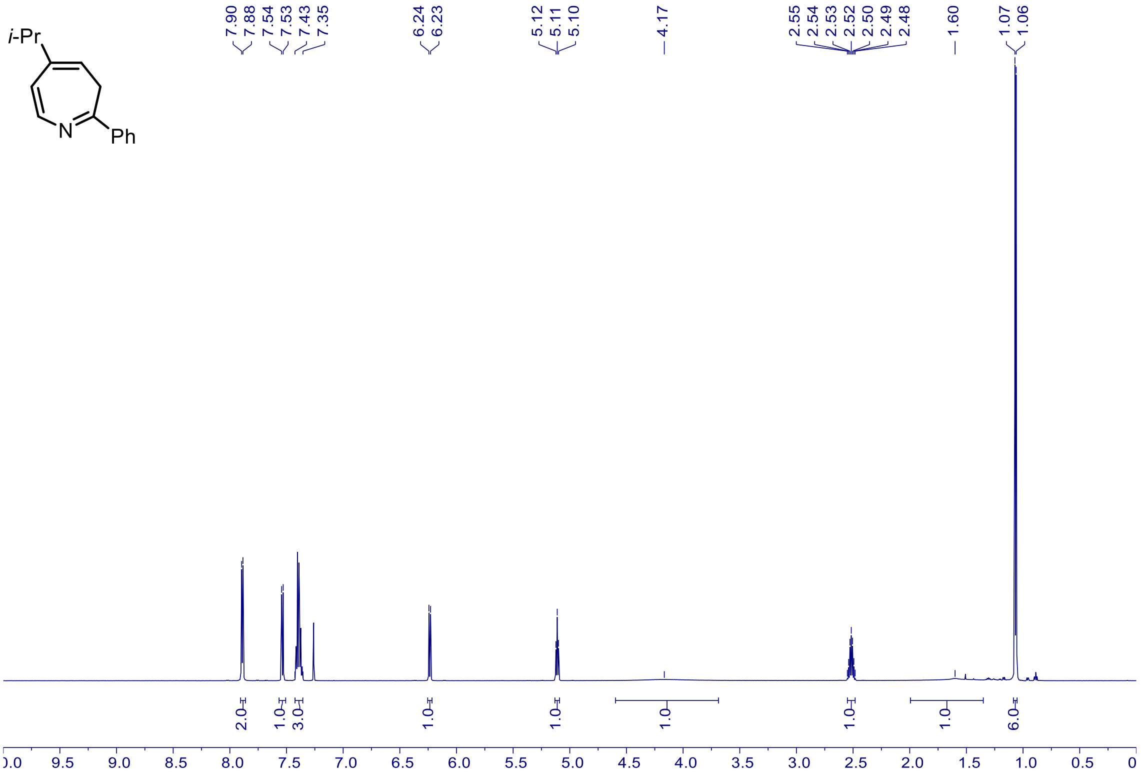

17 – ^{13}C NMR (151 MHz, CDCl3)

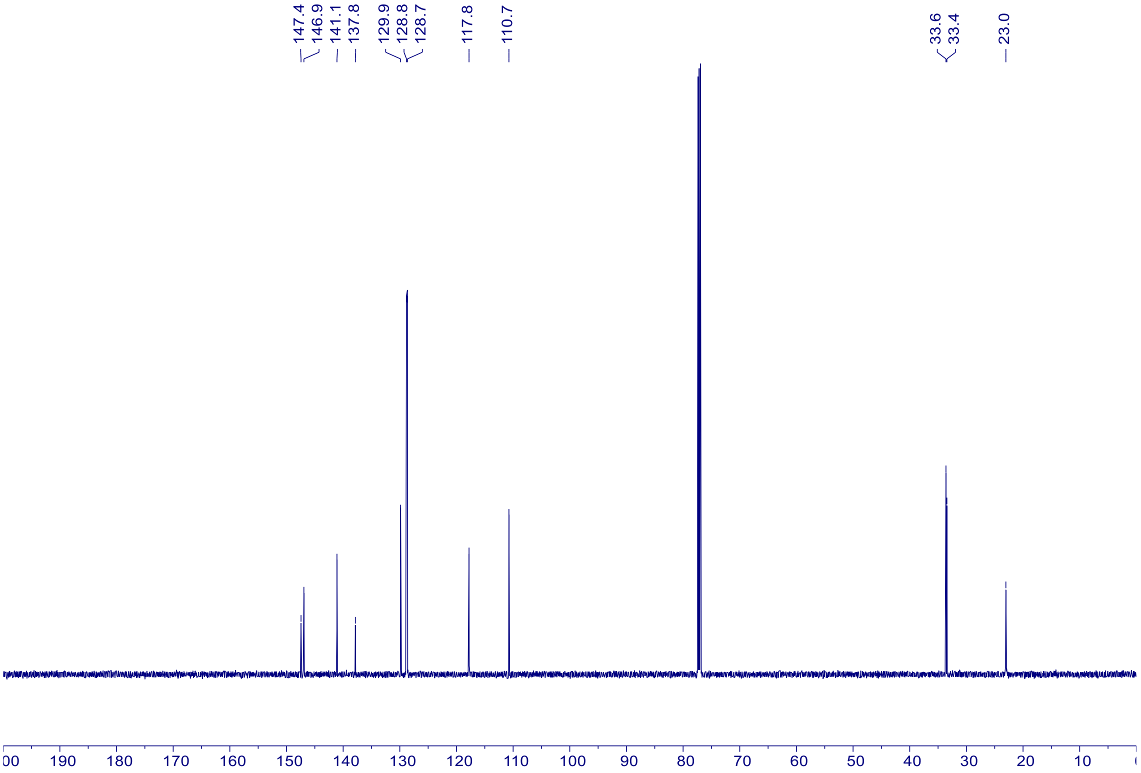

20 – ^{1}H NMR (600 MHz, CDCl$_3$)

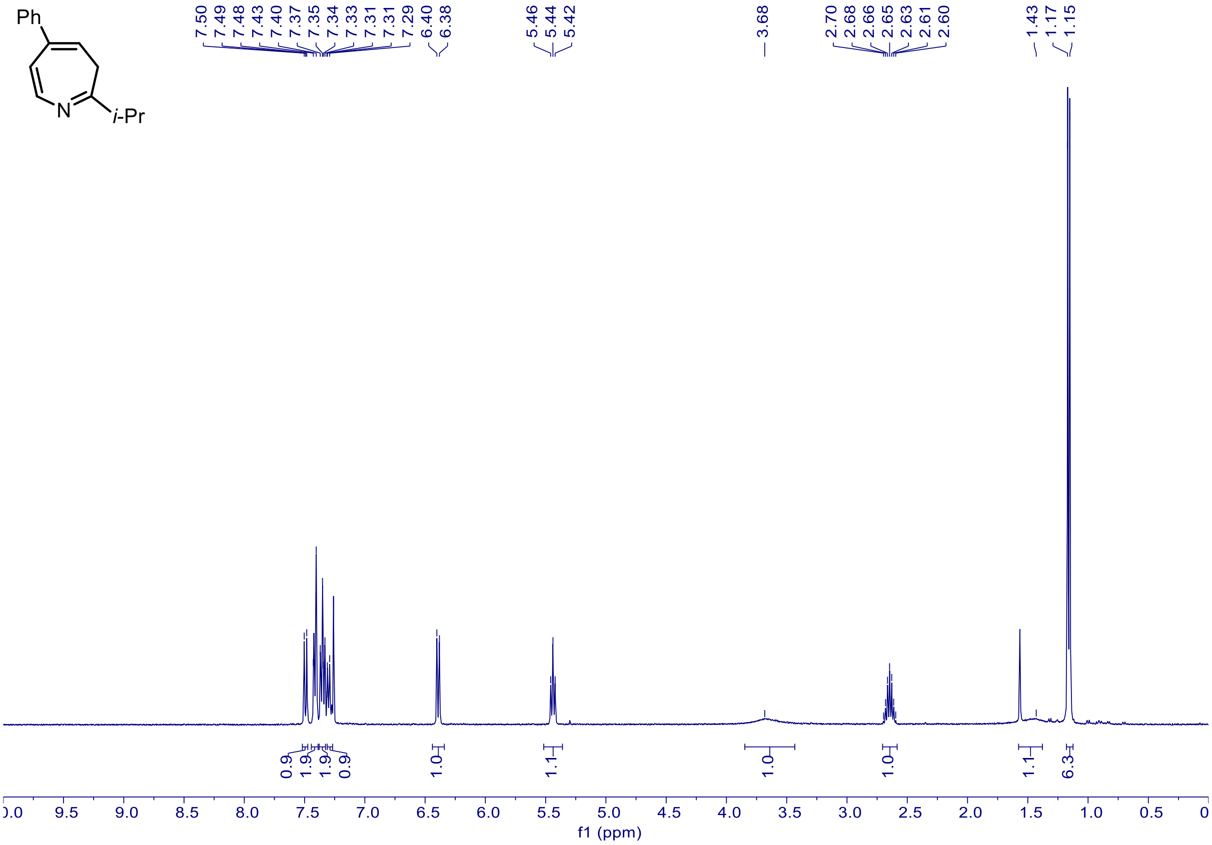

20 – ^{13}C NMR (151 MHz, CDCl$_3$)

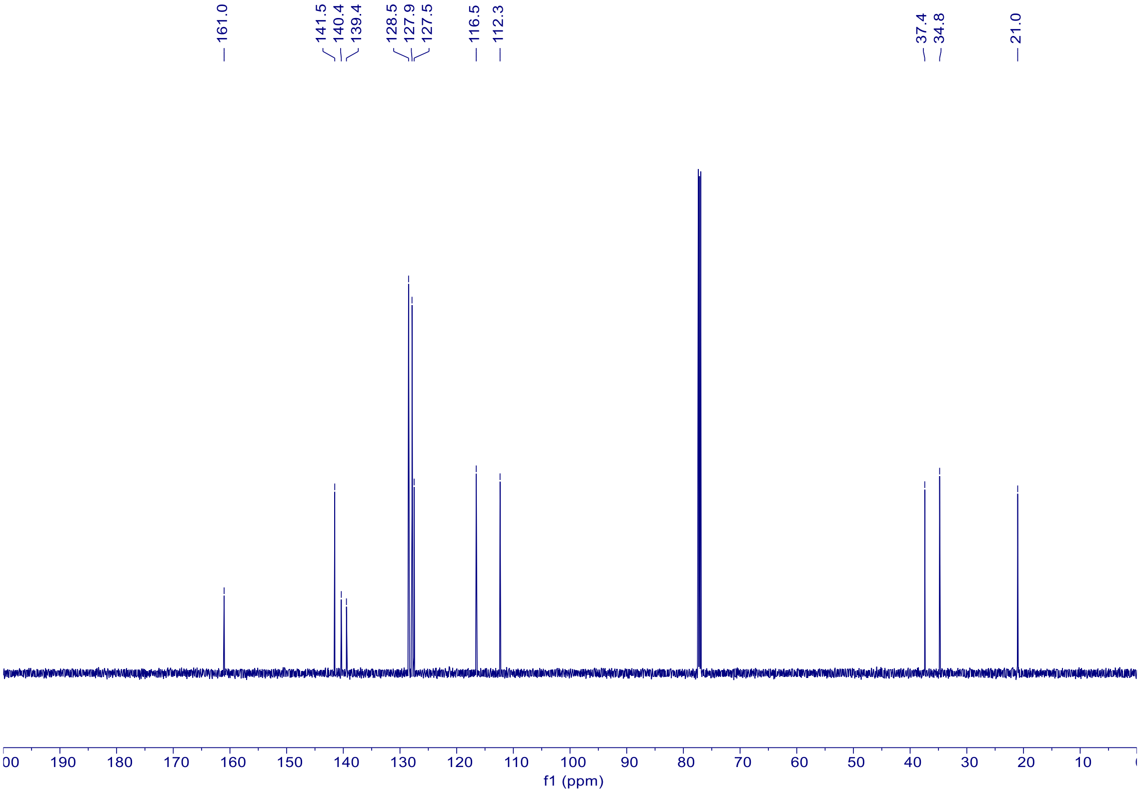

21 – ^{1}H NMR (600 MHz, $CDCl_3$)

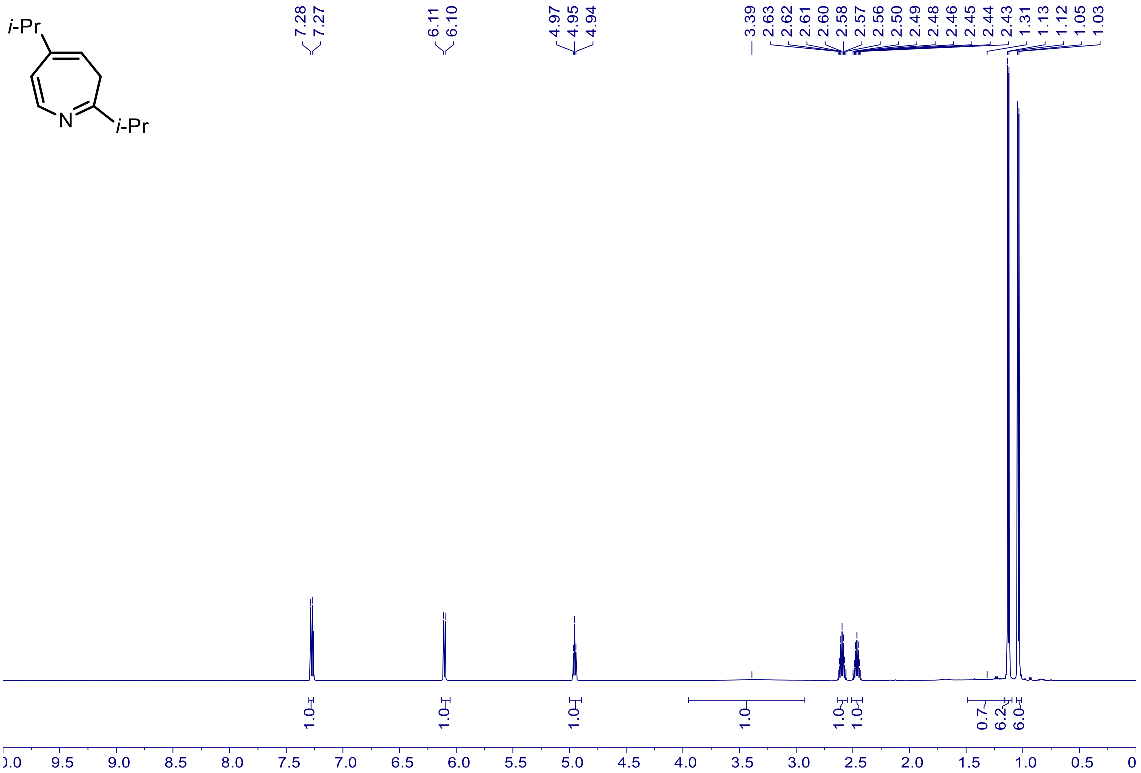

21 – ^{13}C NMR (151 MHz, $CDCl_3$)

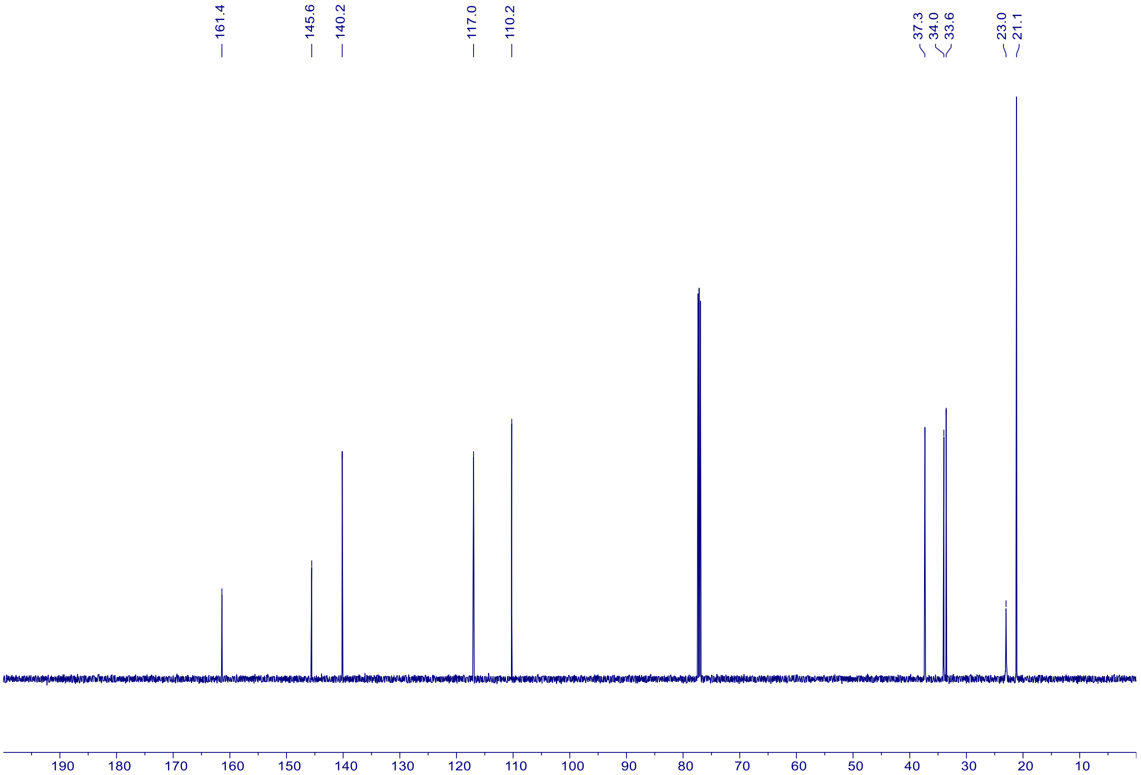

23 – ^{1}H NMR (600 MHz, $CDCl_3$)

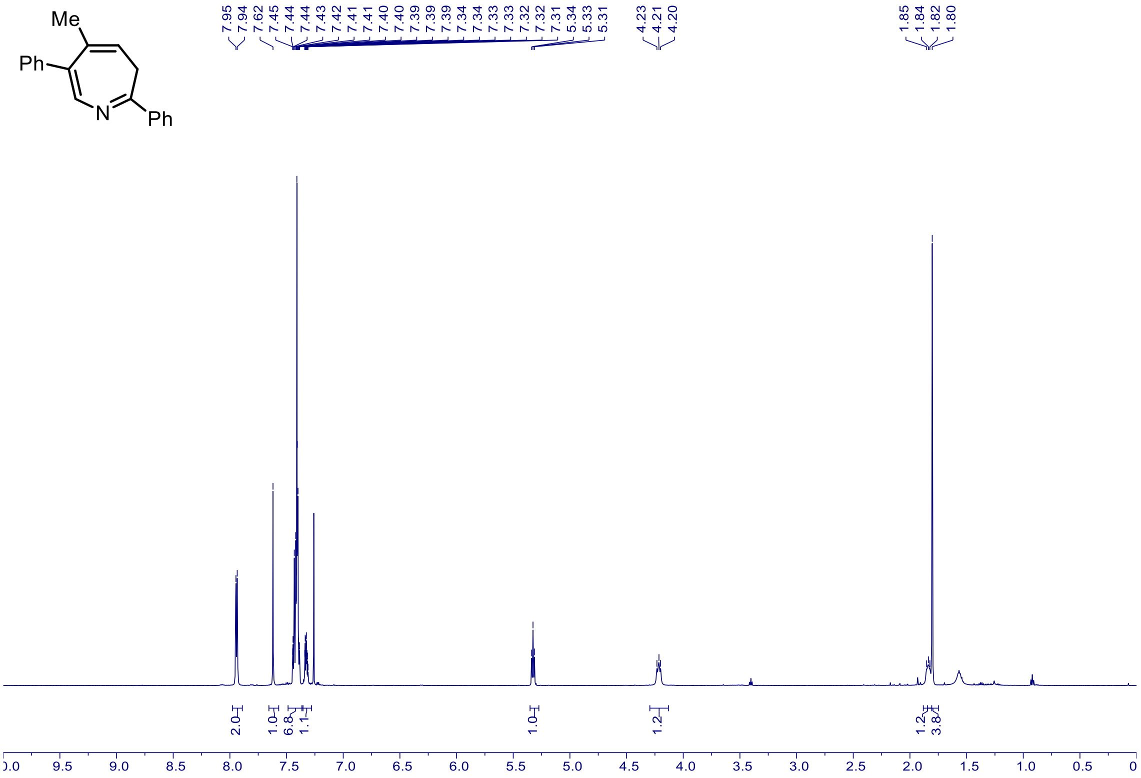

23 – ^{13}C NMR (151 MHz, $CDCl_3$)

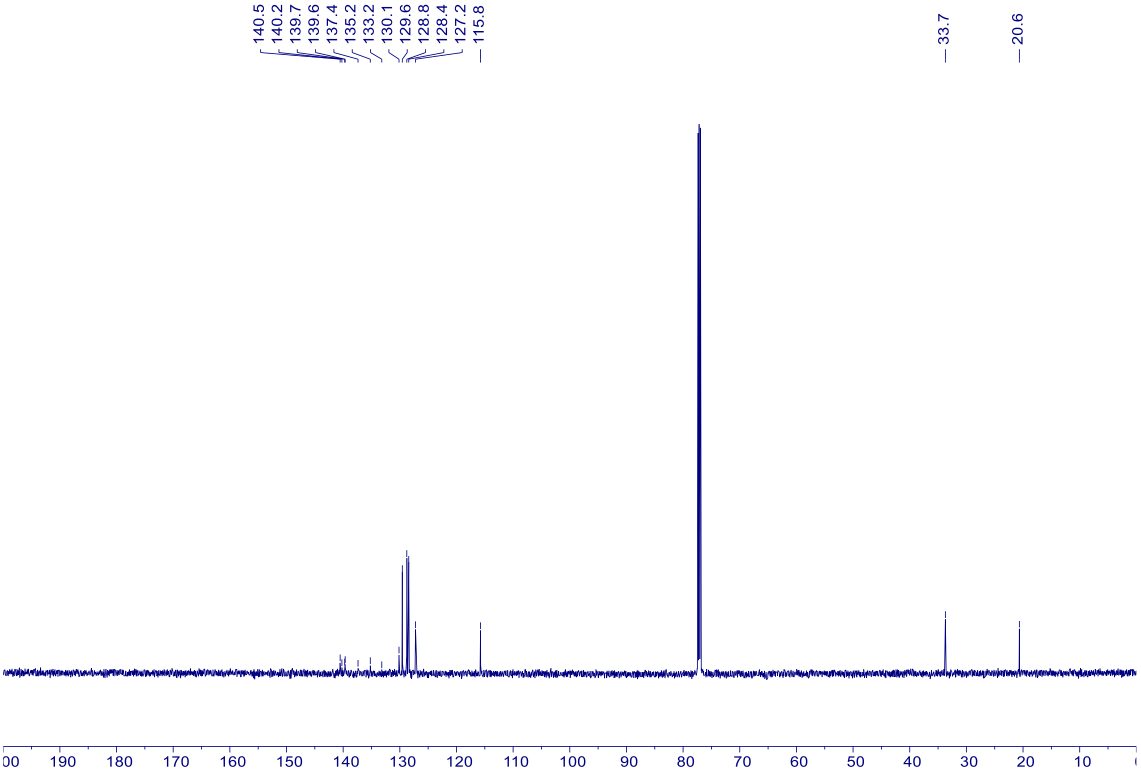

S19 – ^{1}H NMR (600 MHz, $CDCl_3$)

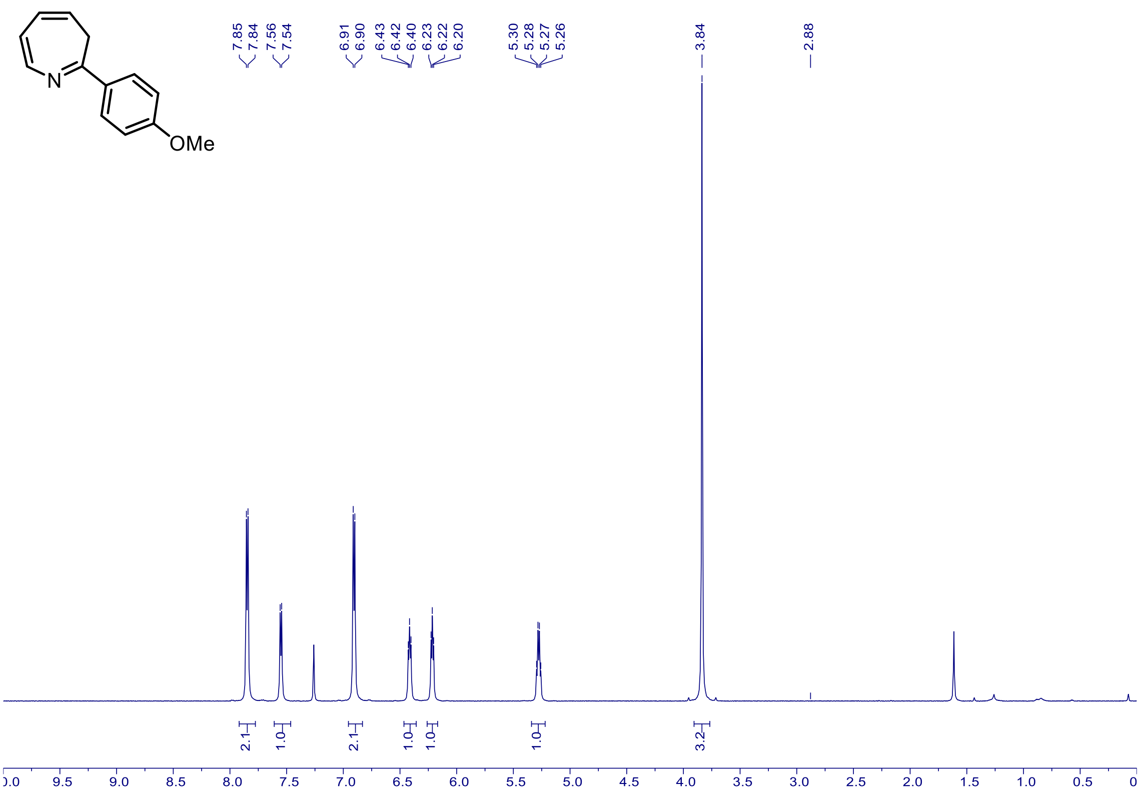

S19 – ^{13}C NMR (151 MHz, $CDCl_3$)

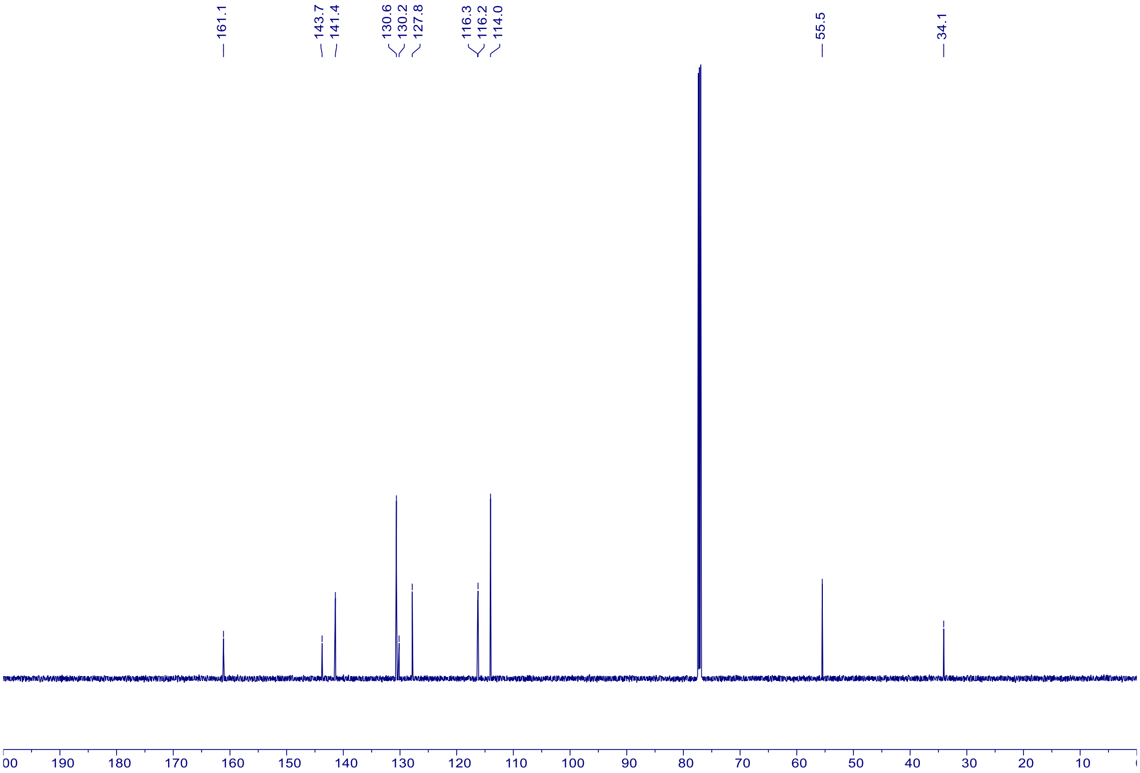

Ox-L2 – ^{1}H NMR (600 MHz, $CDCl_3$)

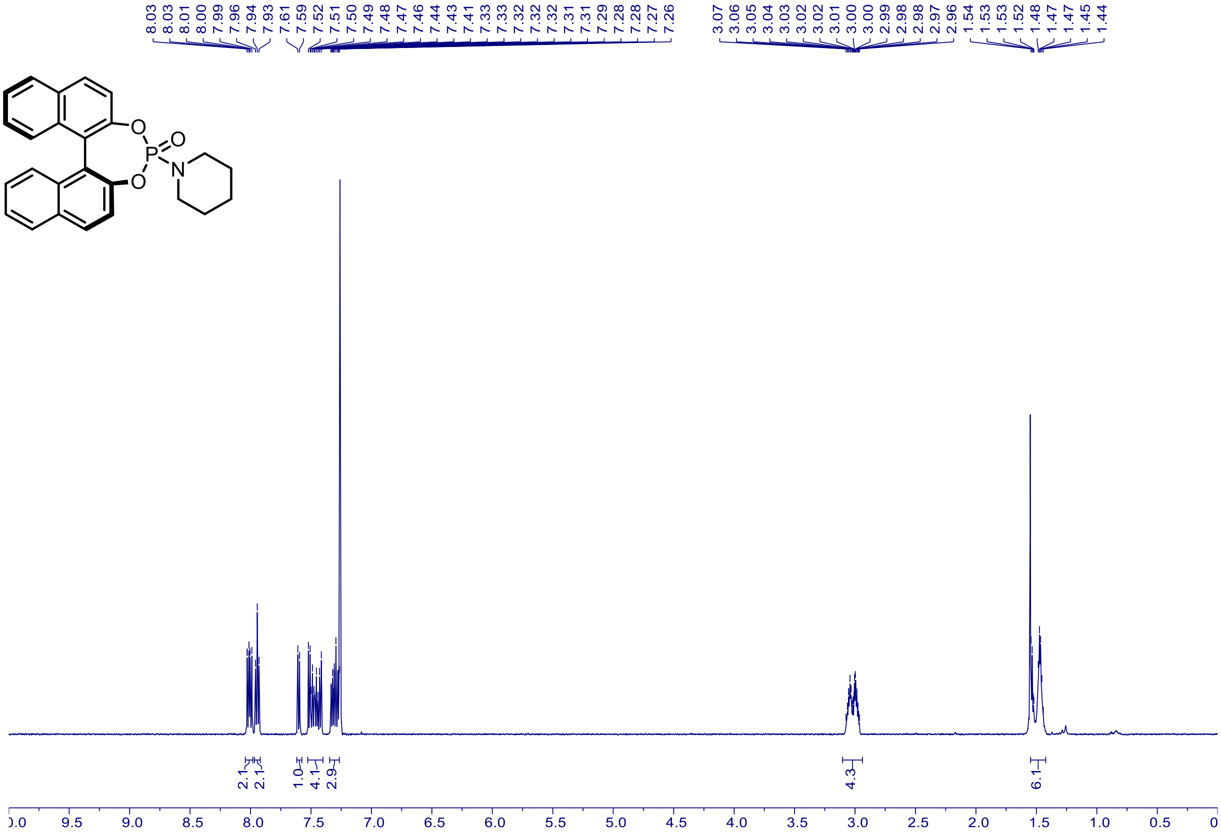

Ox-L2 – ^{13}C NMR (151 MHz, $CDCl_3$)

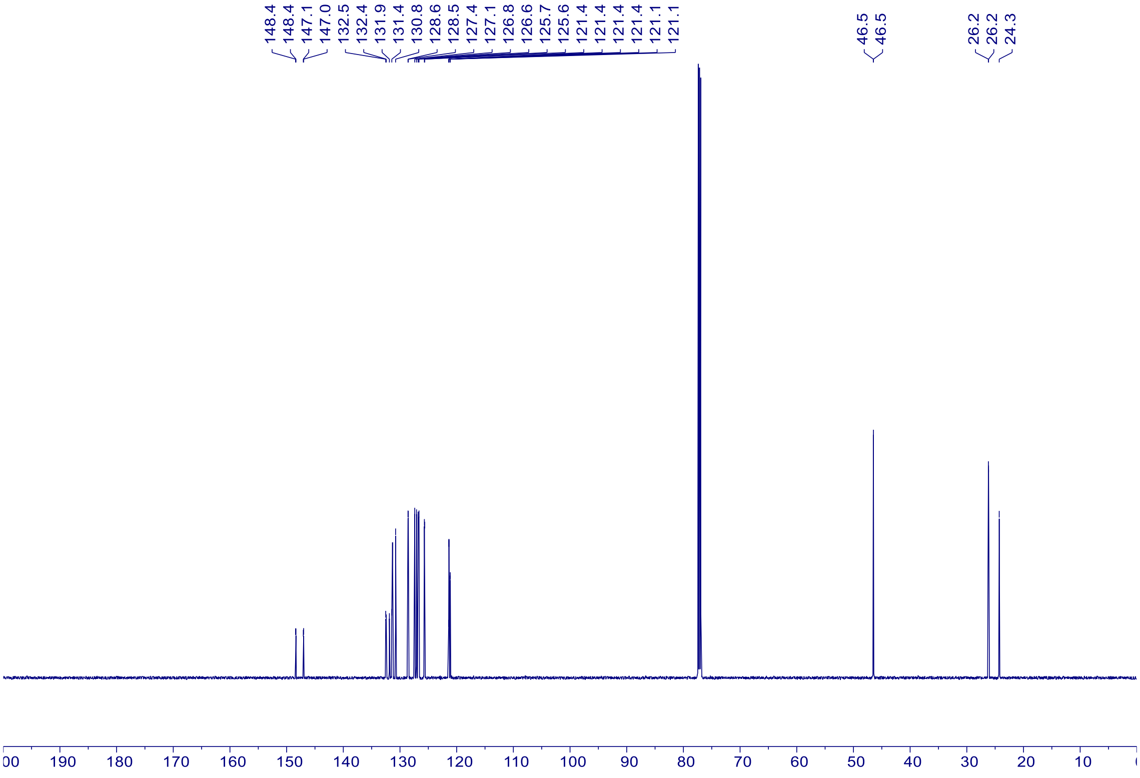

Ox-L2 – ^{31}P NMR (243 MHz, $CDCl_3$)

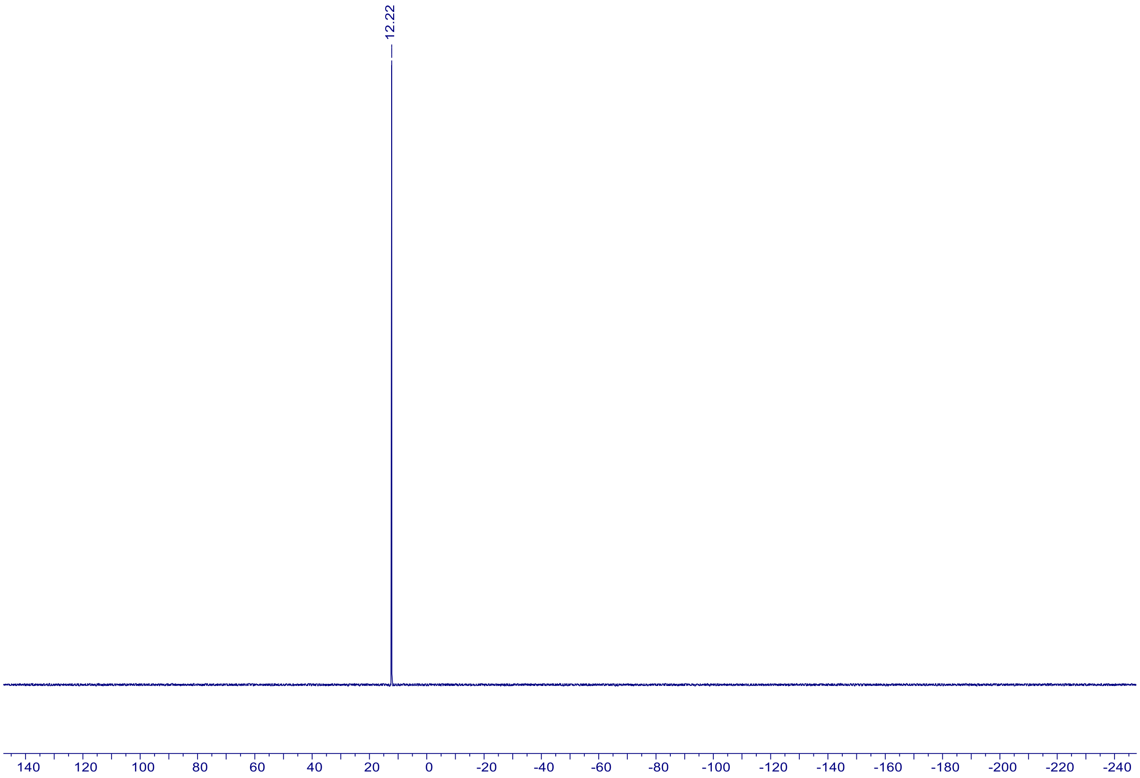

1a – ^{1}H NMR (600 MHz, $CDCl_3$)

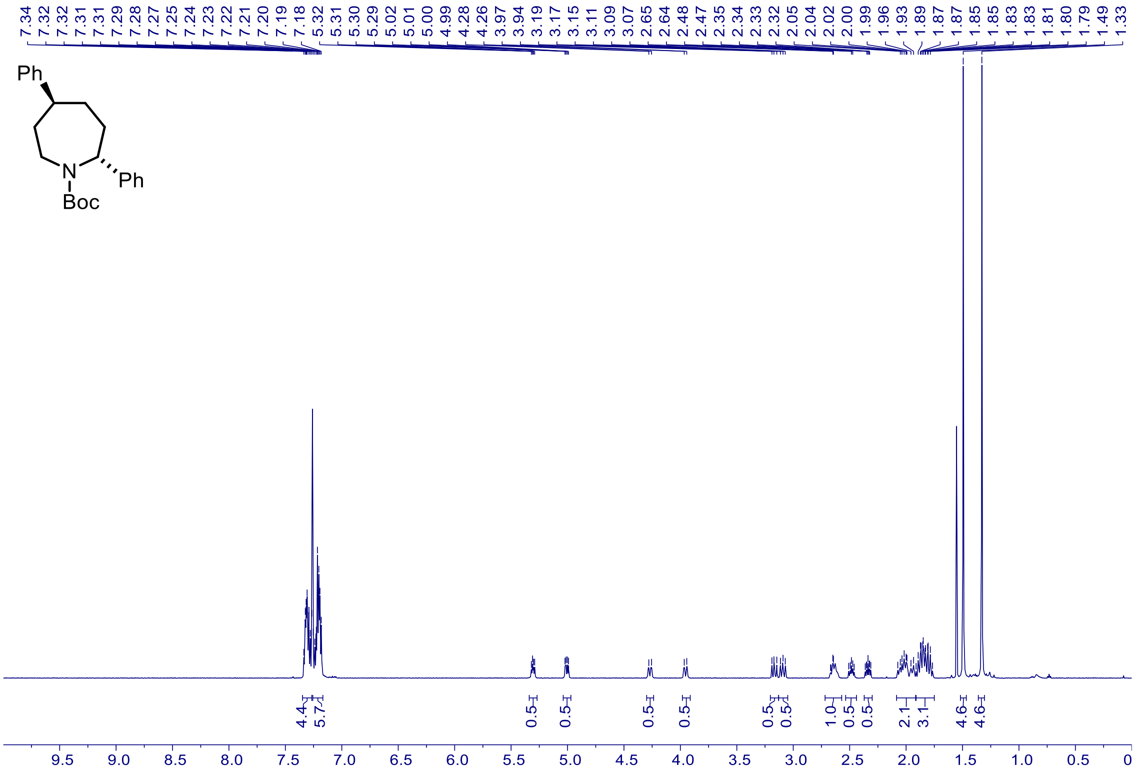

1a – ^{13}C NMR (151 MHz, $CDCl_3$)

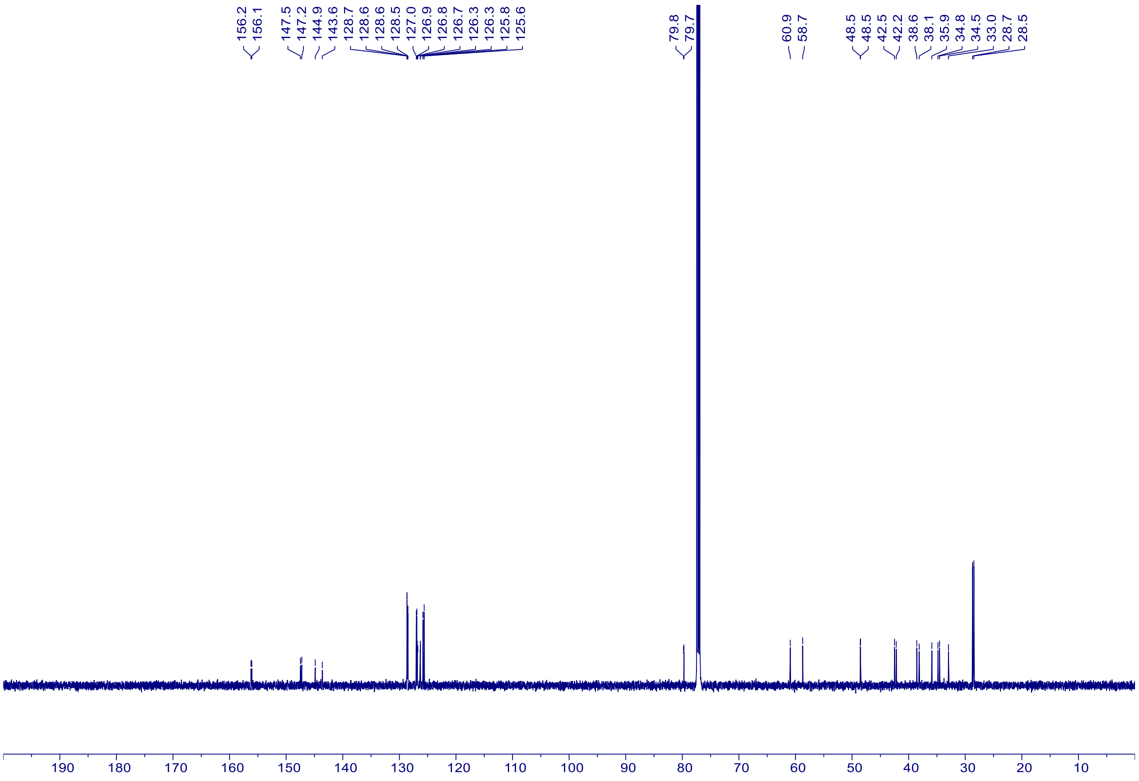

1a – ^{1}H-^{1}H NOESY (600 MHz, $CDCl_3$)

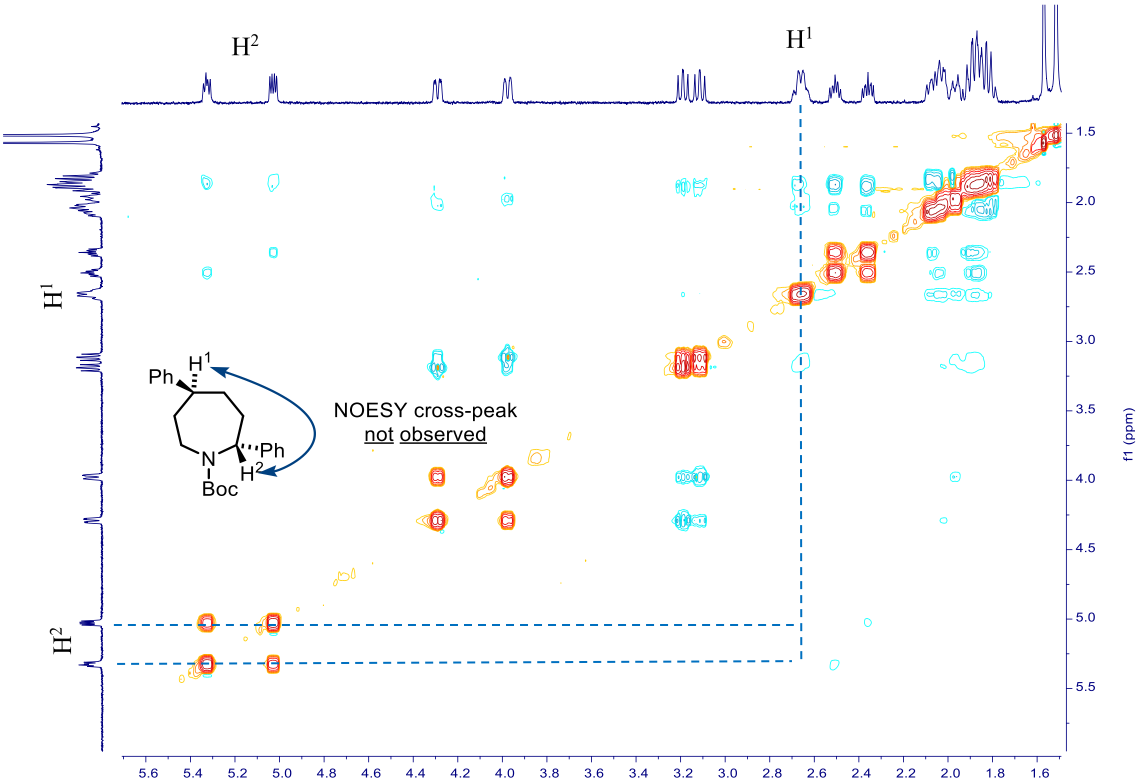

Int-1 – ^{1}H NMR (600 MHz, CDCl$_3$)

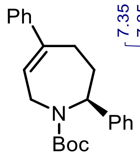

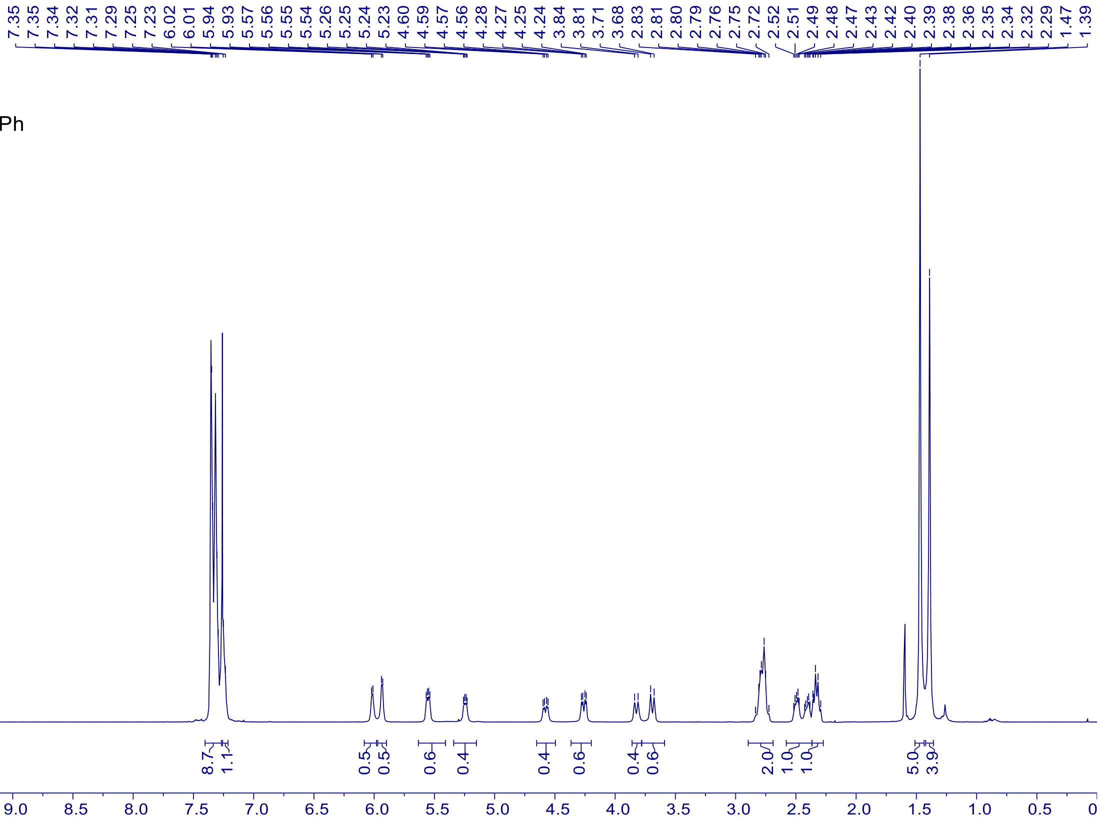

Int-1 – ^{13}C NMR (151 MHz, CDCl$_3$)

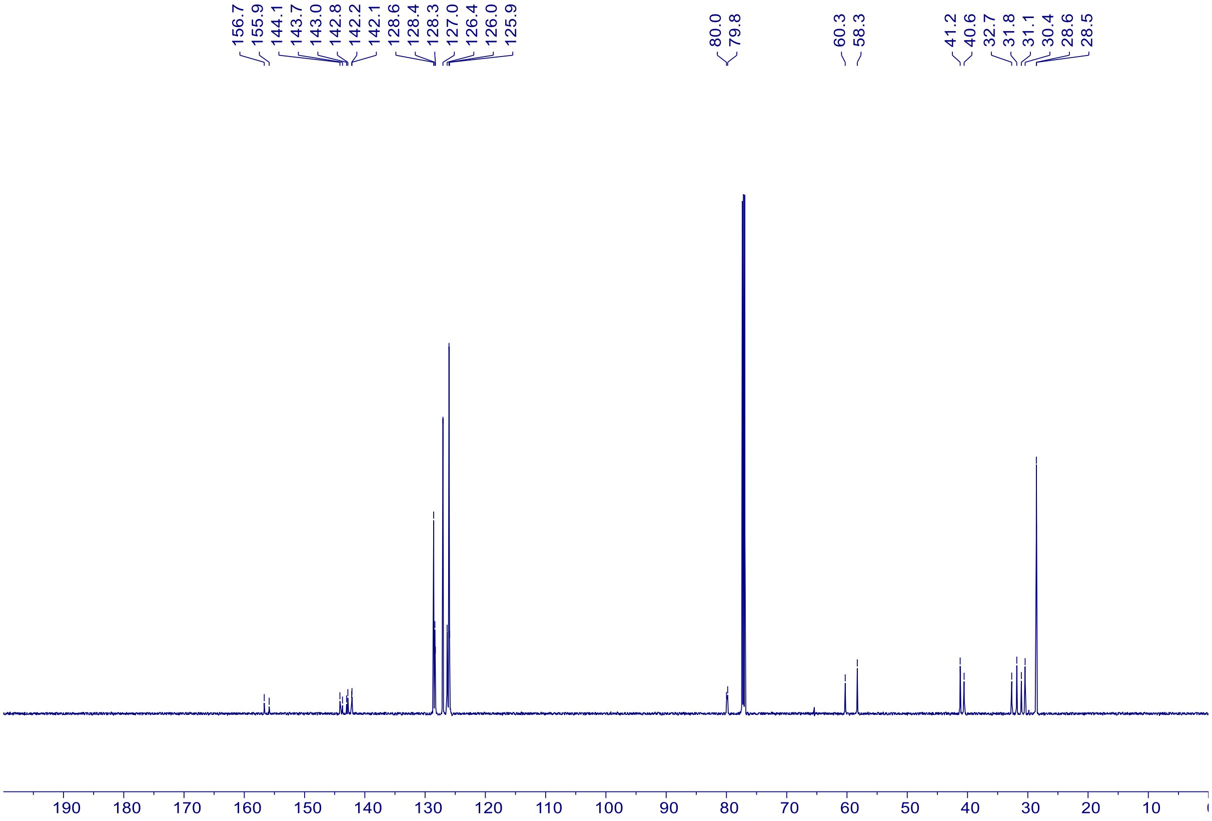

2a – ^{1}H NMR (600 MHz, $CDCl_3$)

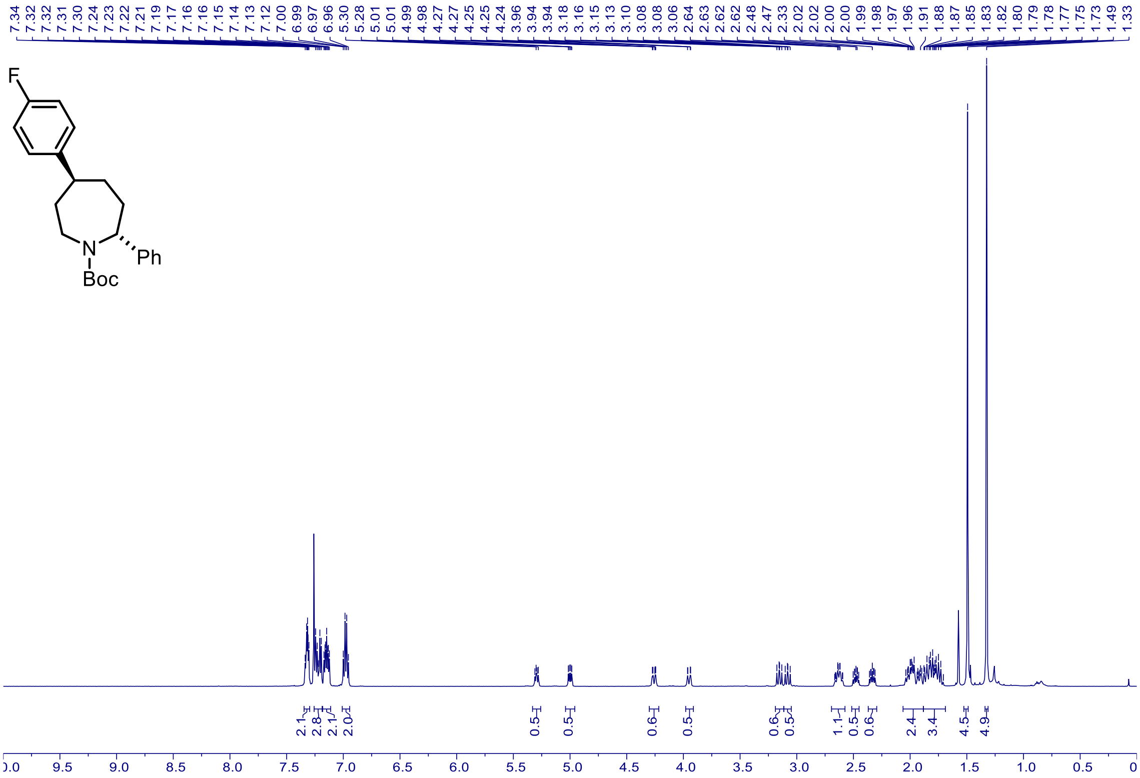

2a – ^{13}C NMR (151 MHz, $CDCl_3$)

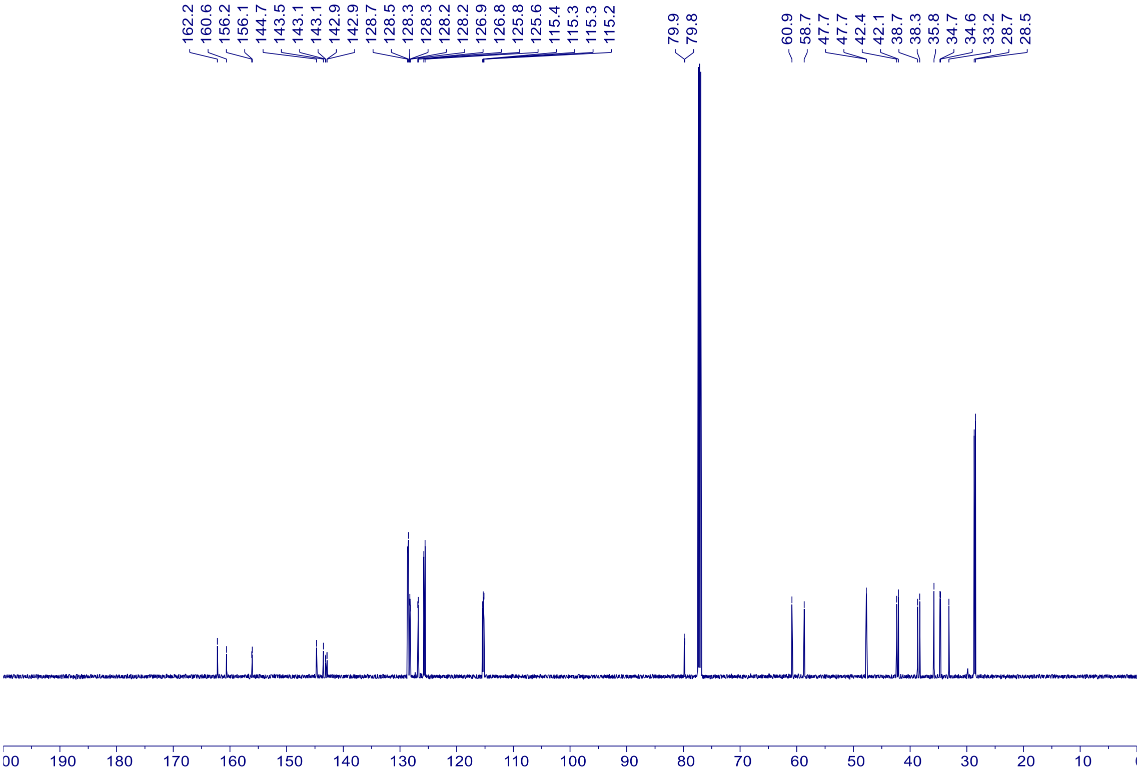

2a – ^{19}F NMR (564 MHz, $CDCl_3$)

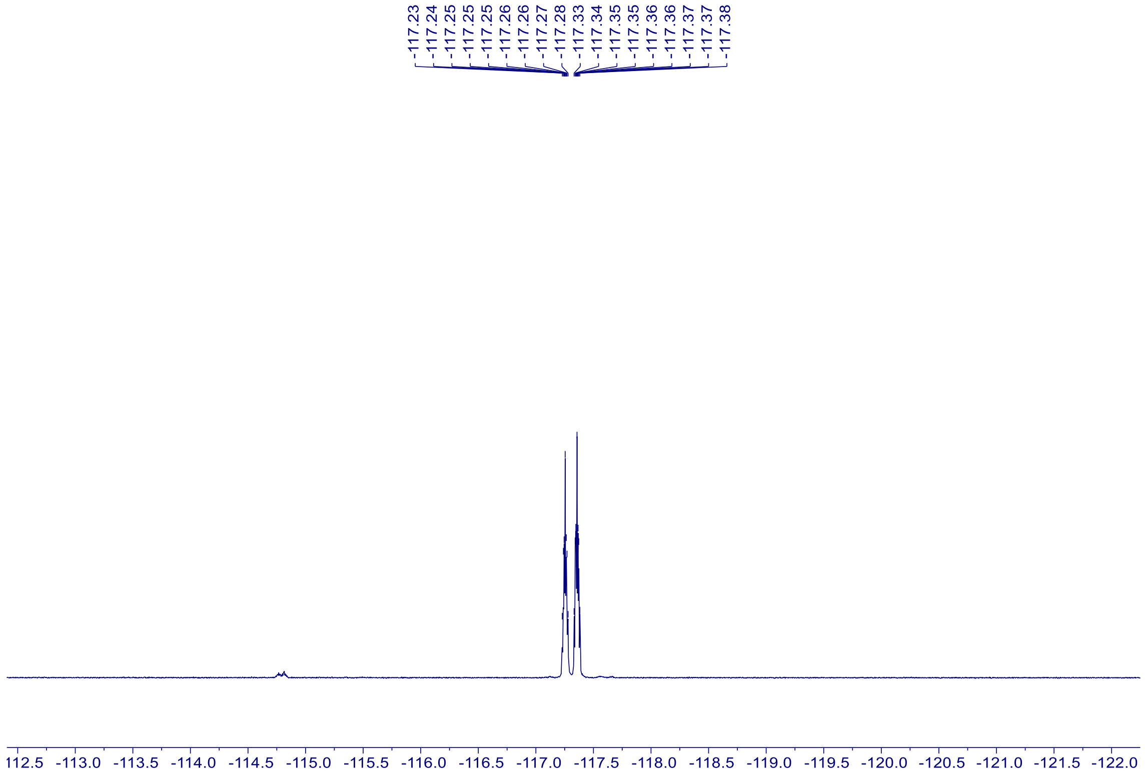

3a – ^{1}H NMR (600 MHz, $CDCl_3$)

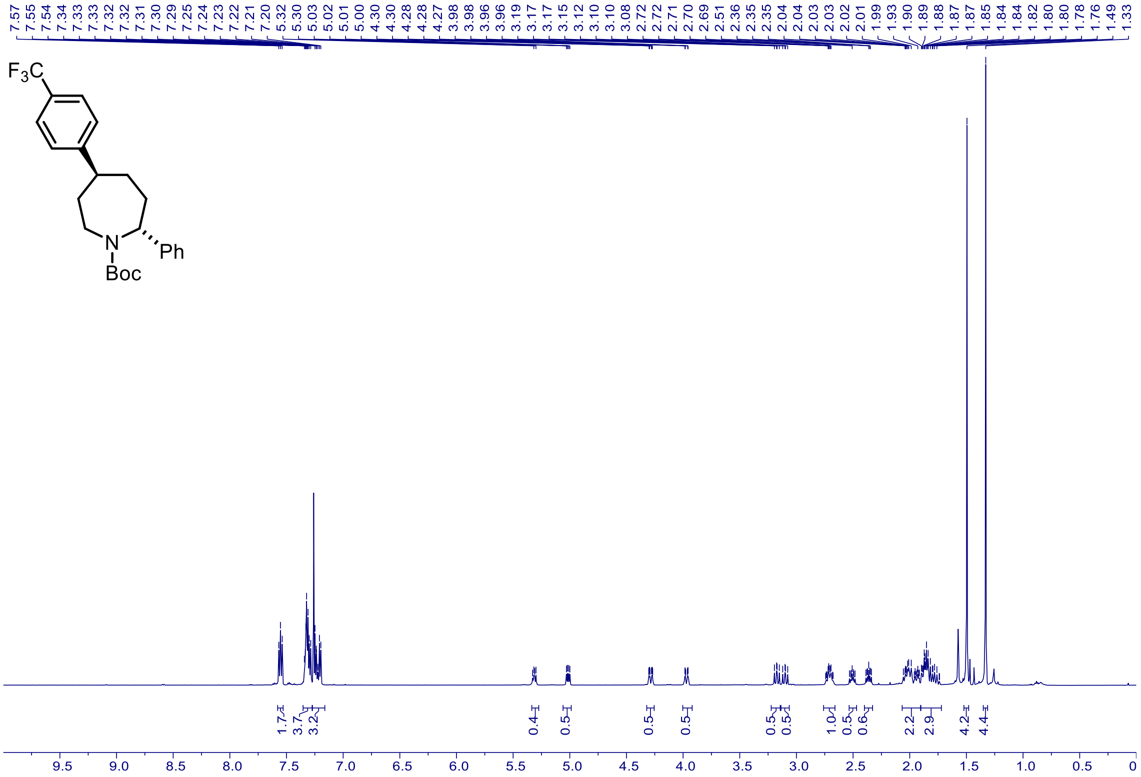

3a – ^{13}C NMR (151 MHz, $CDCl_3$)

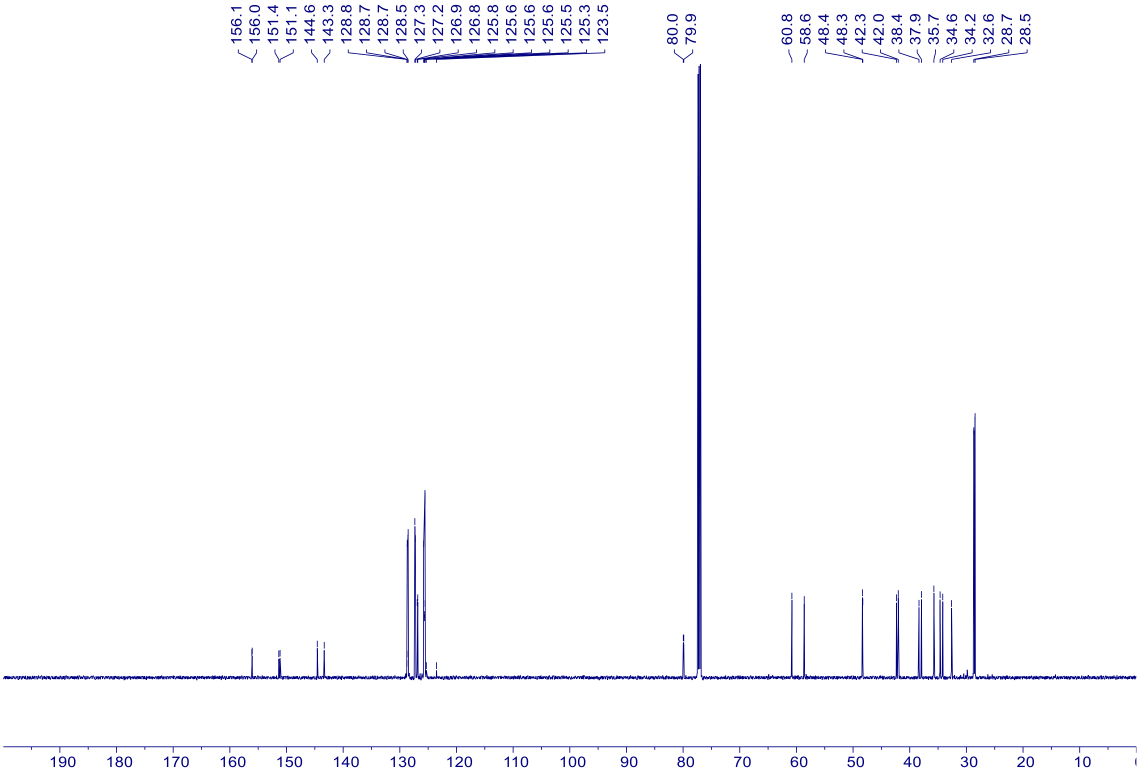

3a – ^{19}F NMR (564 MHz, $CDCl_3$)

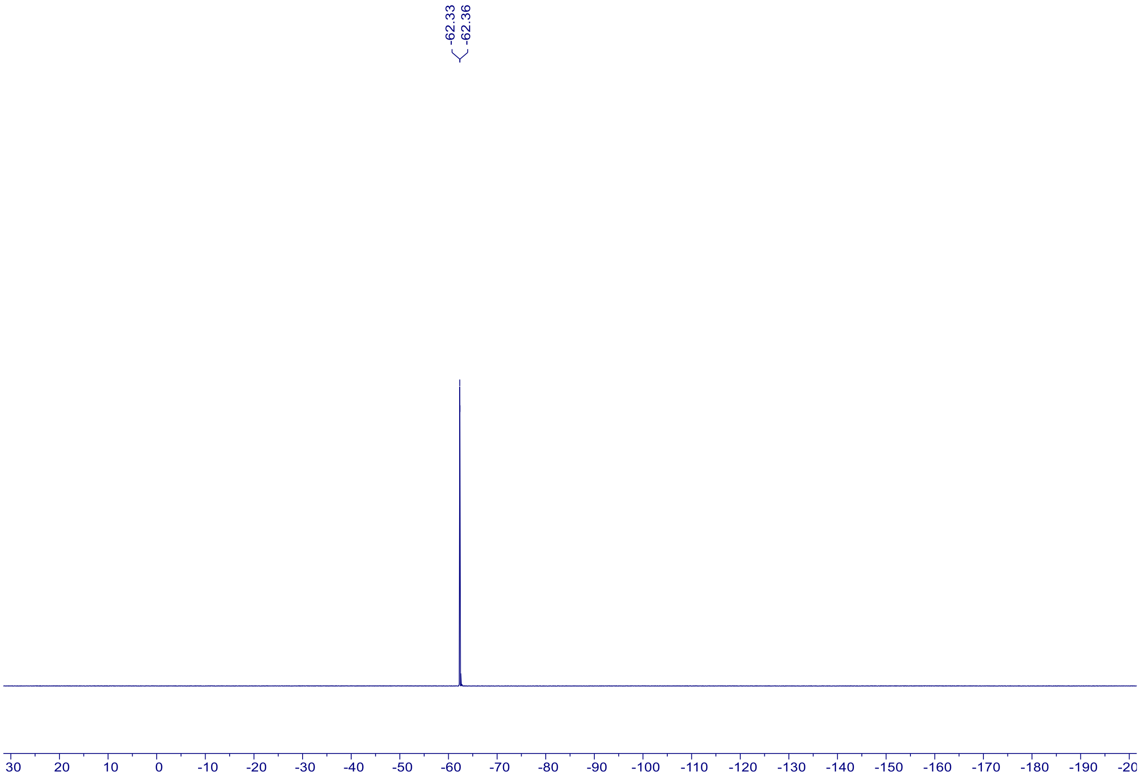

4a – ^{1}H NMR (600 MHz, $CDCl_3$)

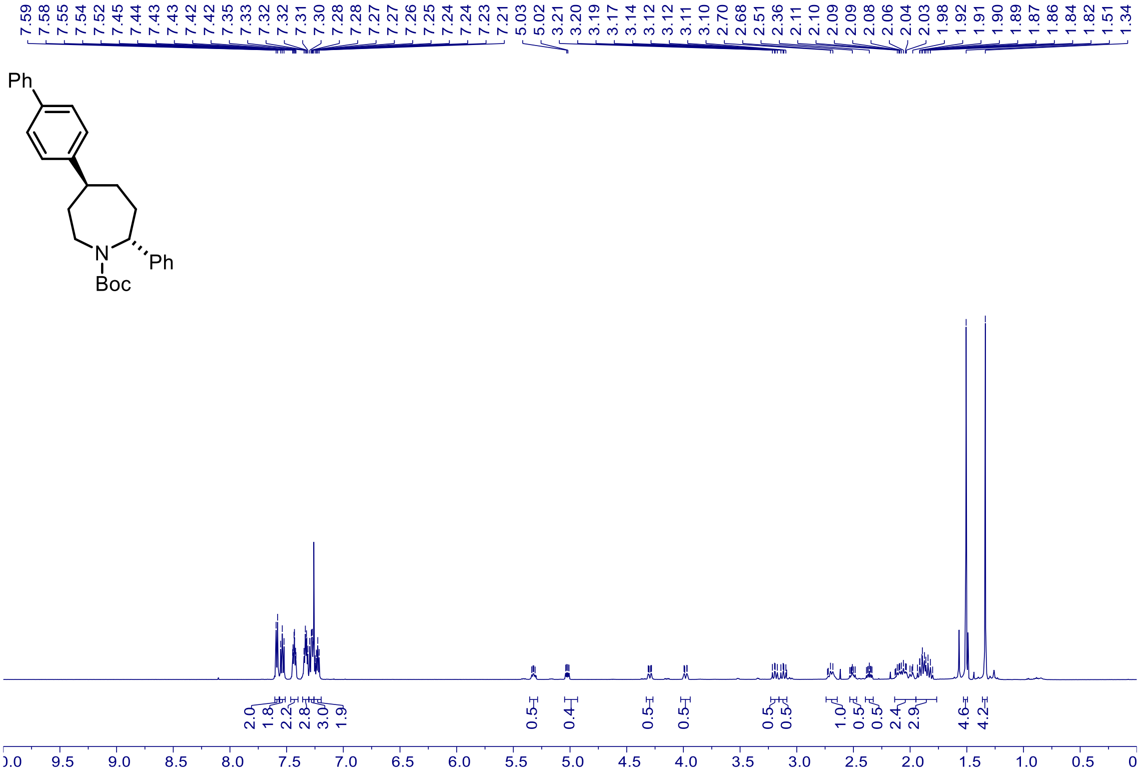

4a – ^{13}C NMR (151 MHz, $CDCl_3$)

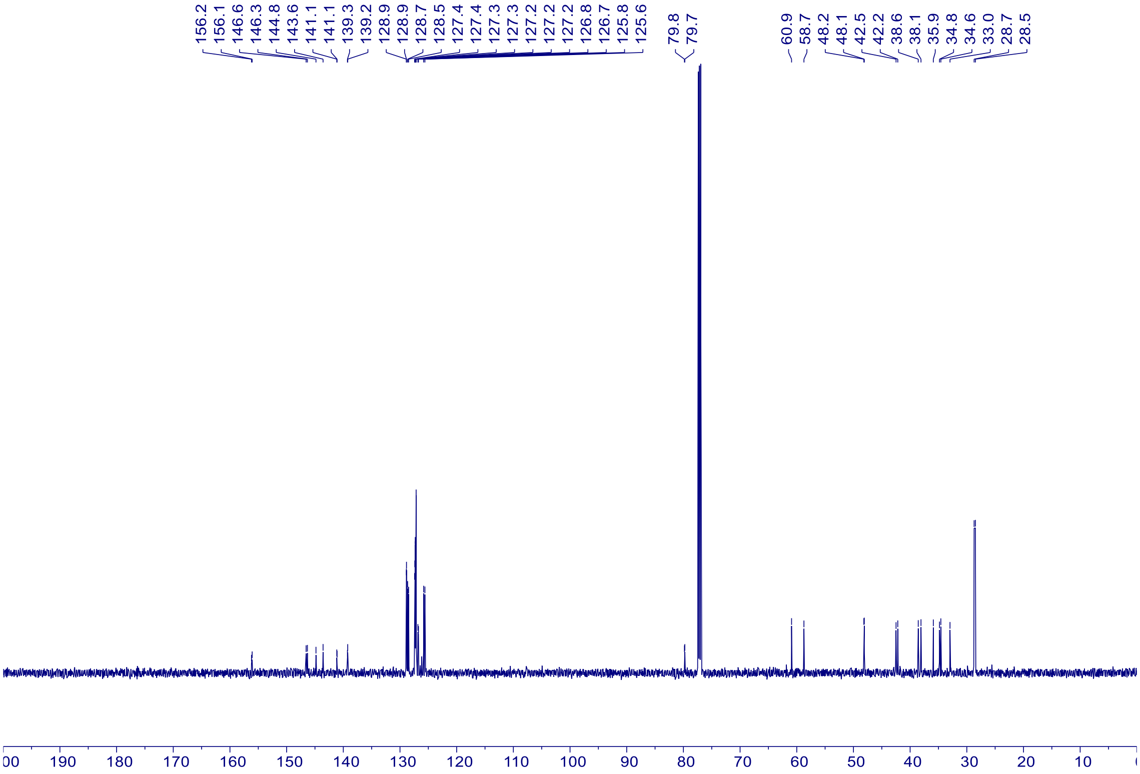

5a – ^{1}H NMR (600 MHz, $CDCl_3$)

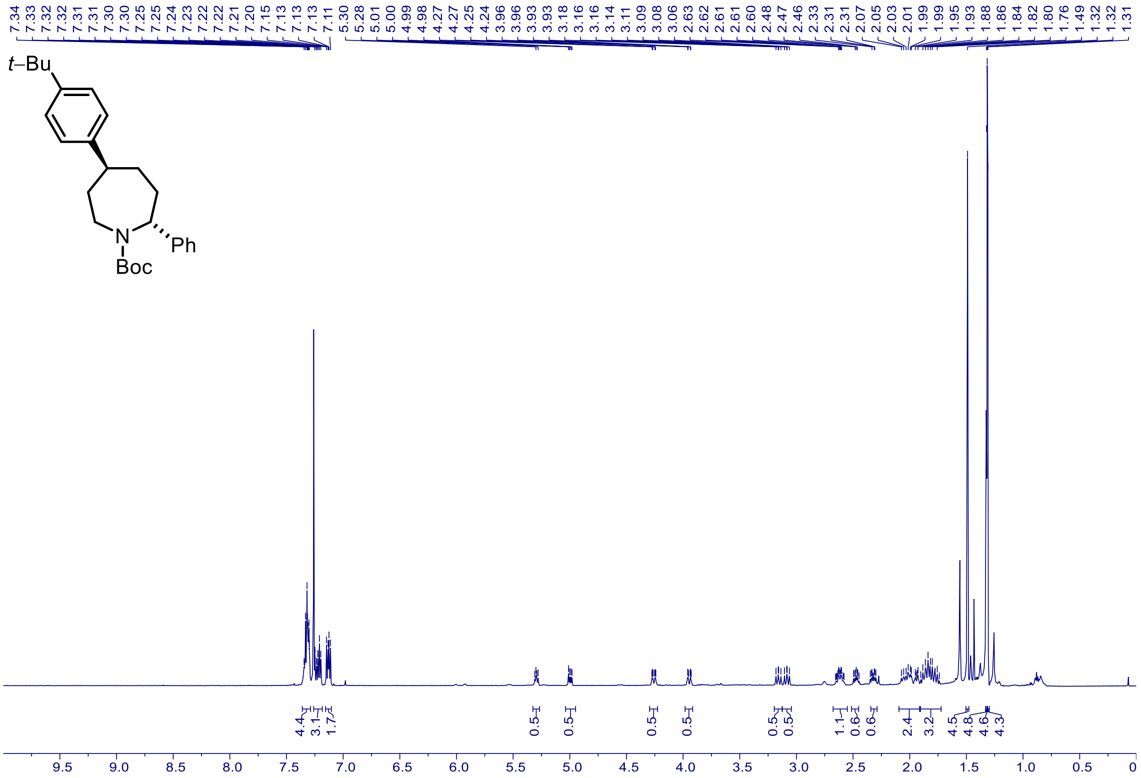

5a – ^{13}C NMR (151 MHz, $CDCl_3$)

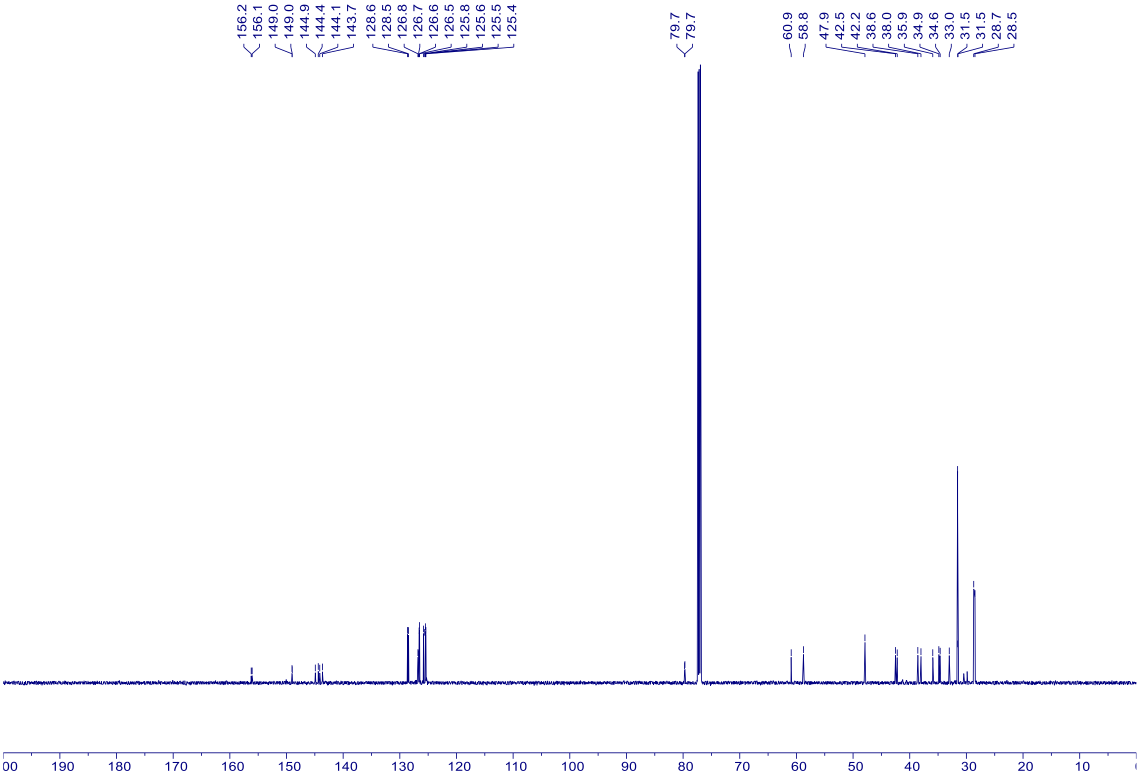

6a – ^{1}H NMR (600 MHz, $CDCl_3$)

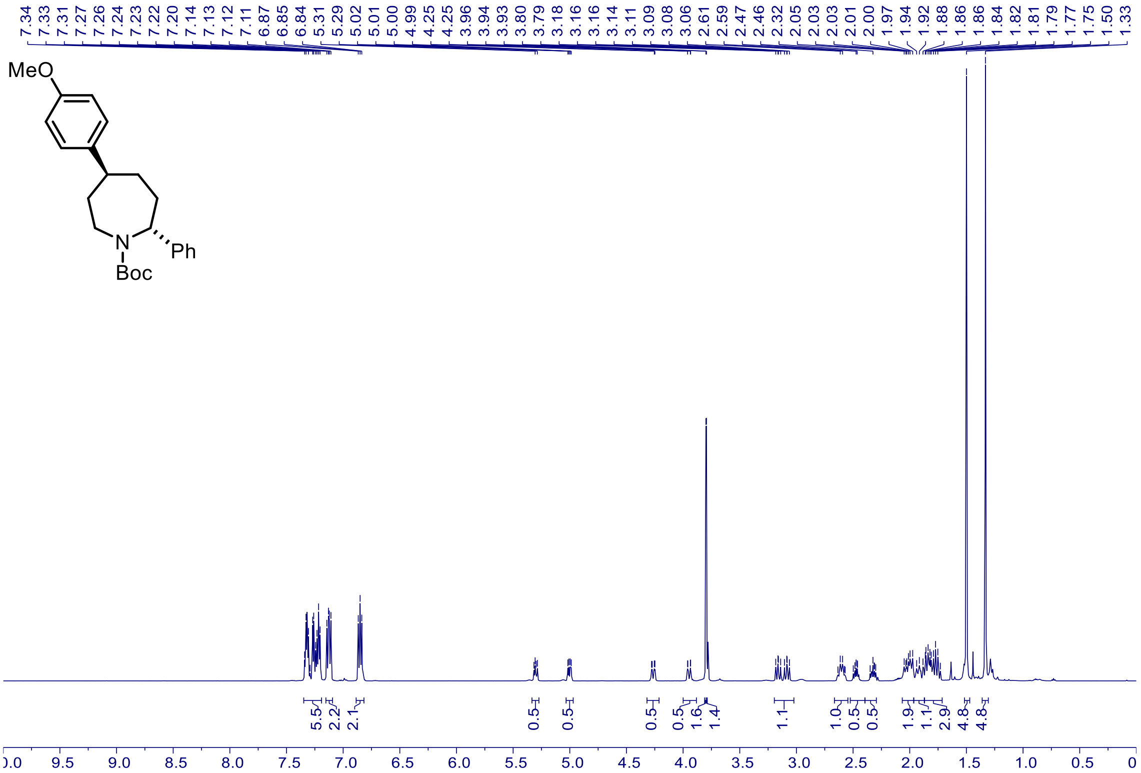

6a – ^{13}C NMR (151 MHz, $CDCl_3$)

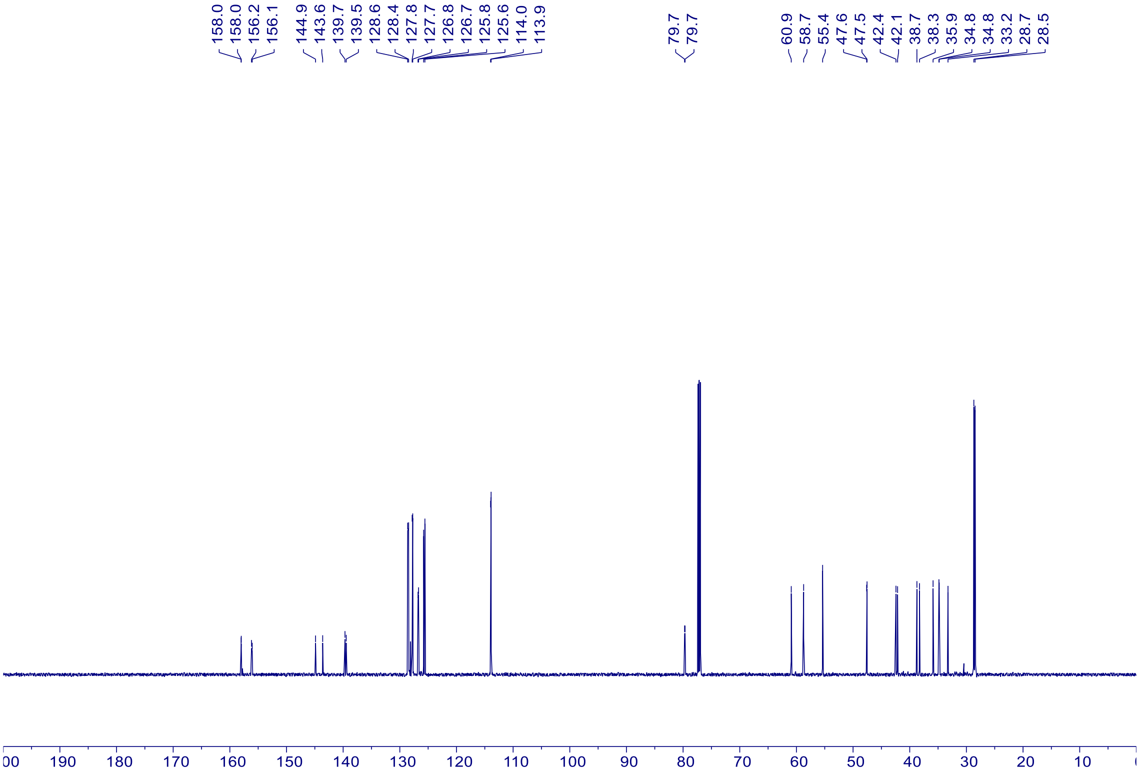

7a – ^{1}H NMR (600 MHz, $CDCl_3$)

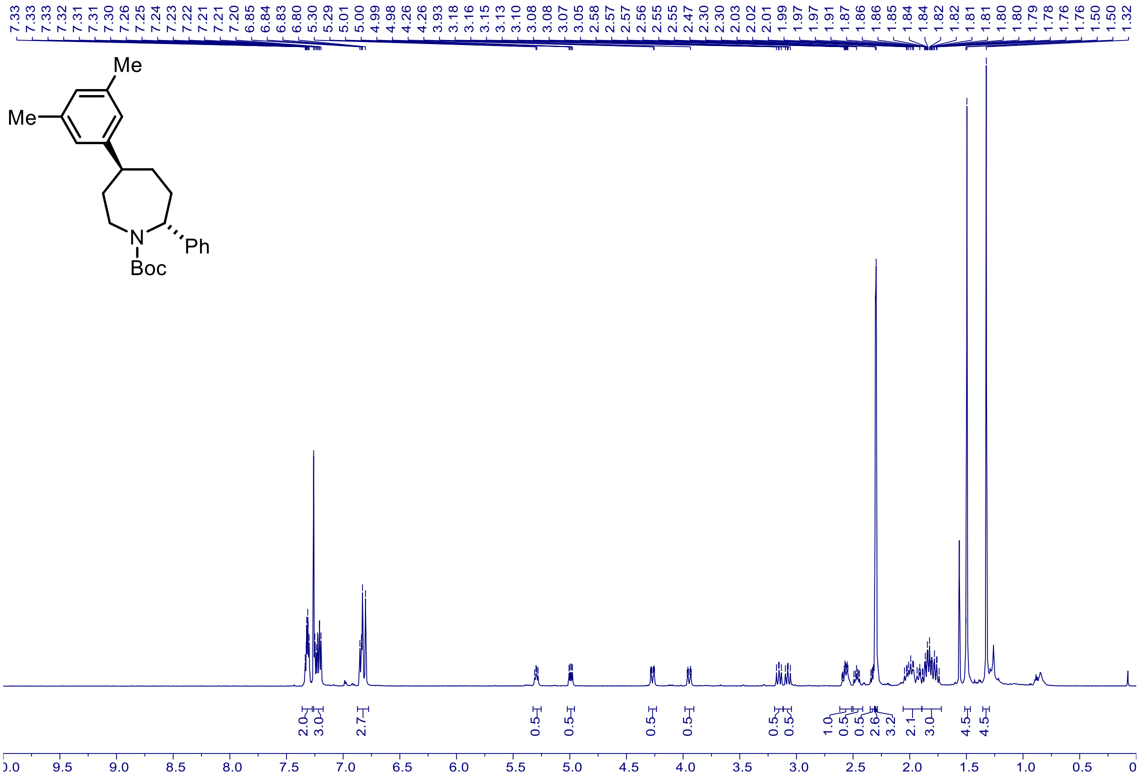

7a – ^{13}C NMR (151 MHz, $CDCl_3$)

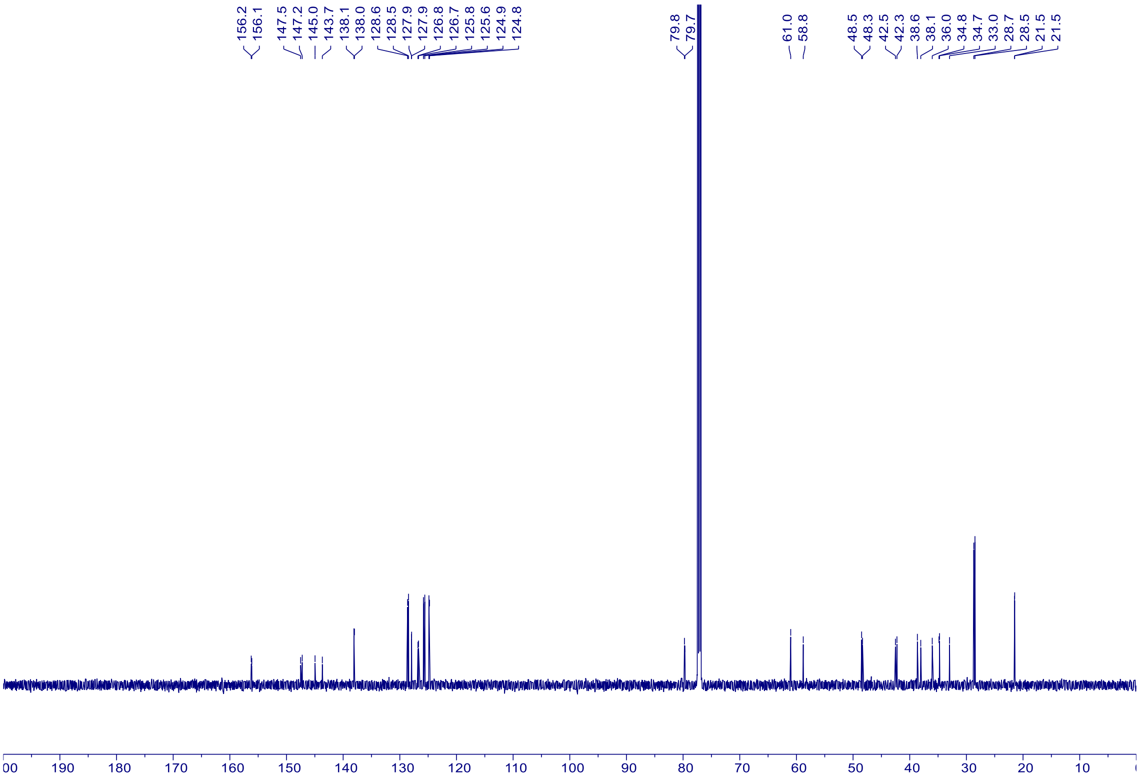

8a – ^{1}H NMR (600 MHz, $CDCl_3$)

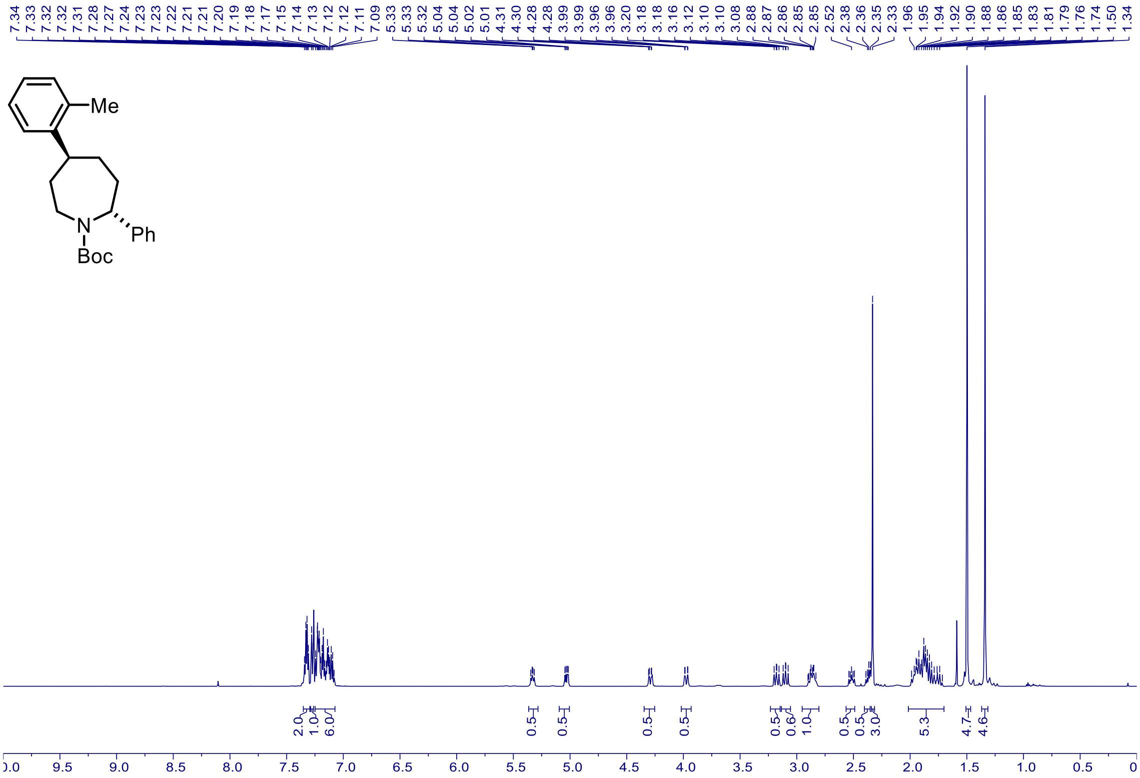

8a – ^{13}C NMR (151 MHz, $CDCl_3$)

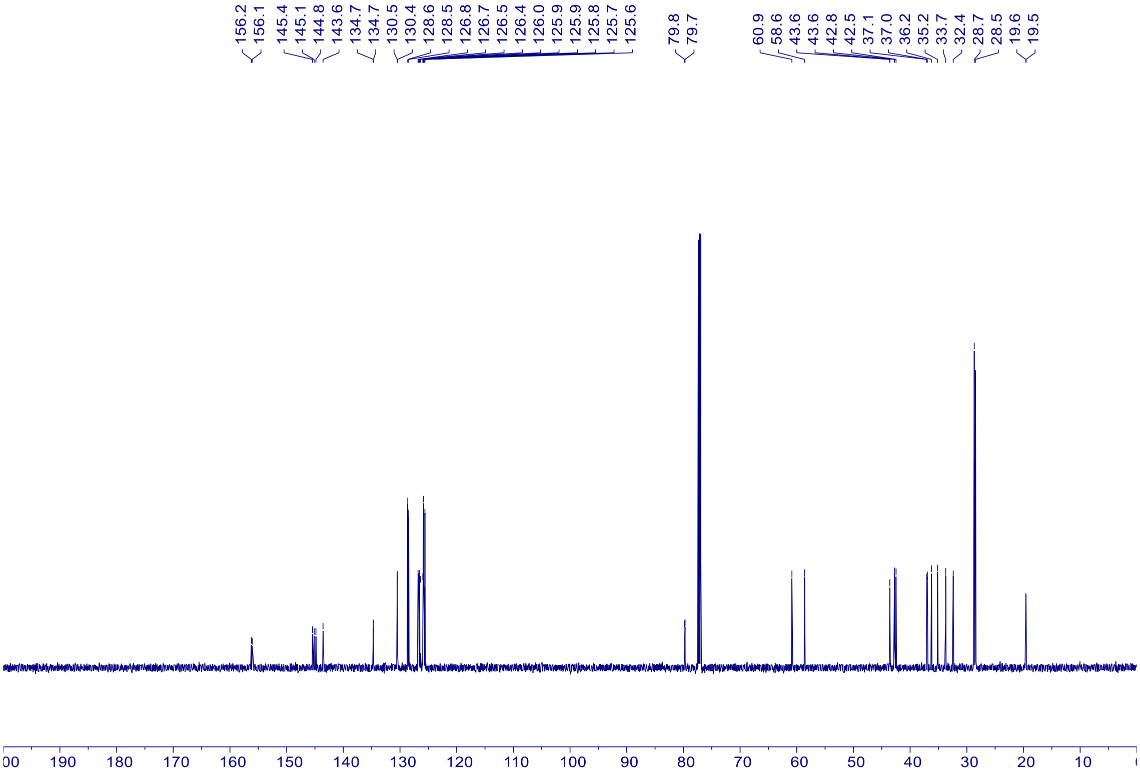

9a – ^{1}H NMR (600 MHz, $CDCl_3$)

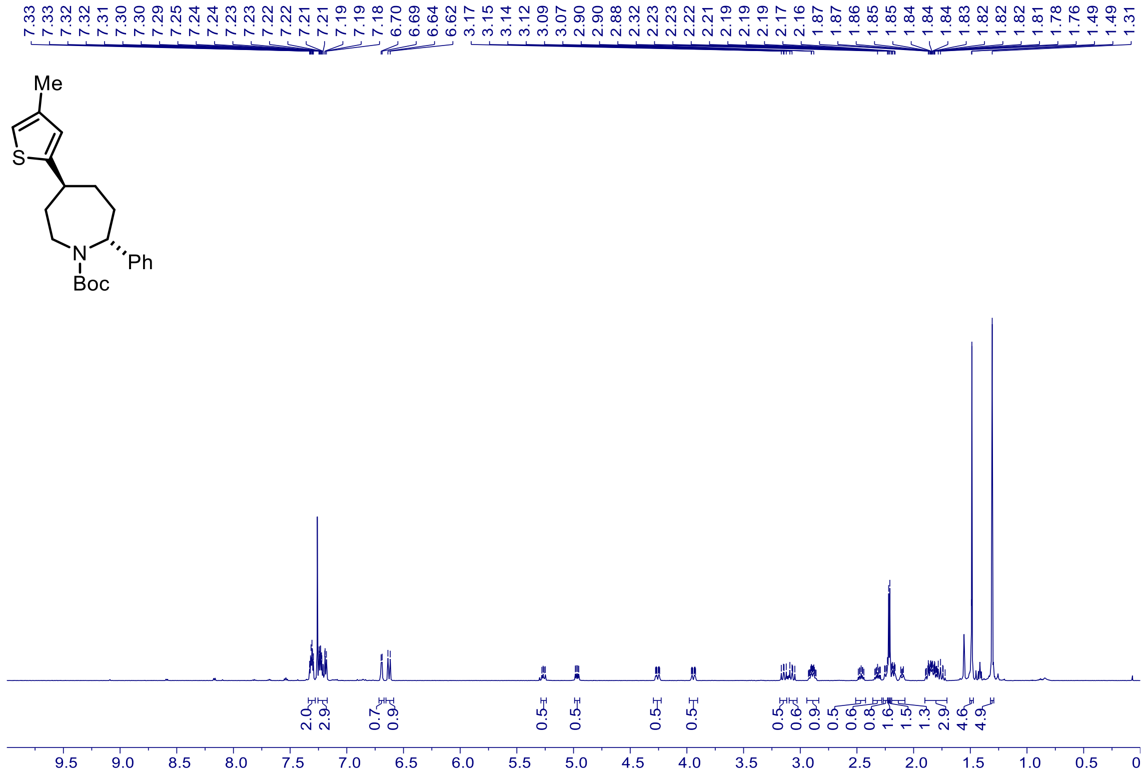

9a – ^{13}C NMR (151 MHz, $CDCl_3$)

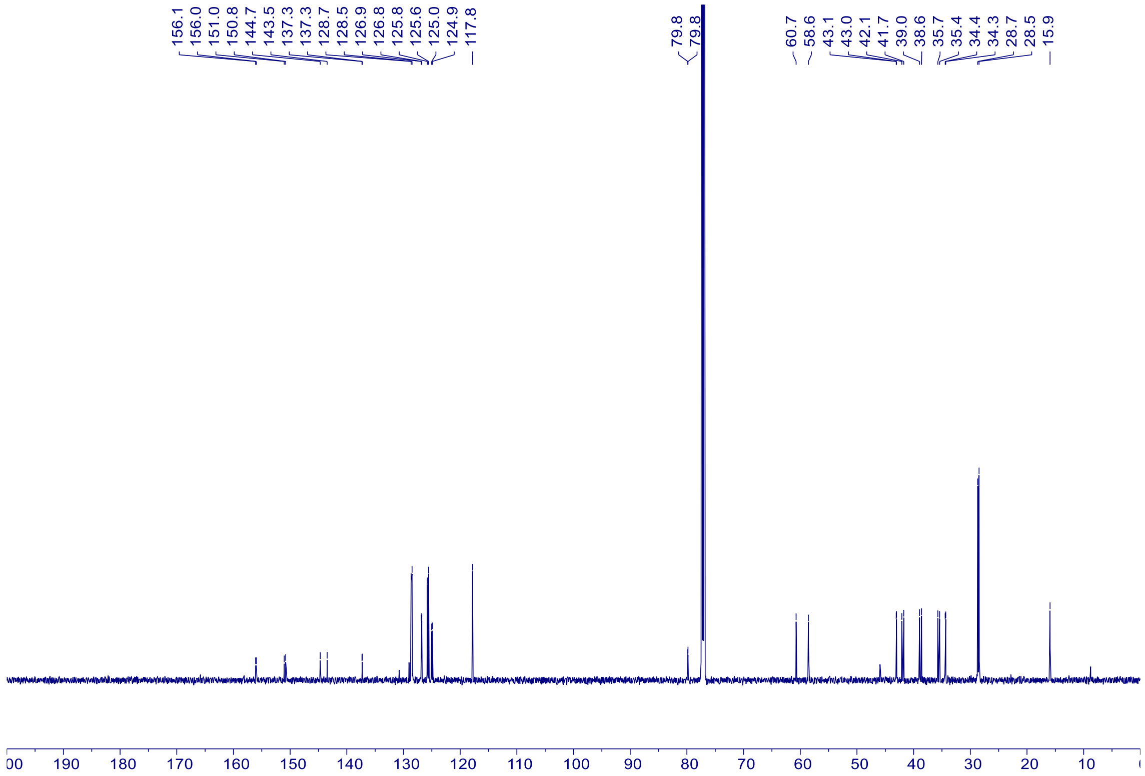

10a – ^{1}H NMR (600 MHz, $CDCl_3$)

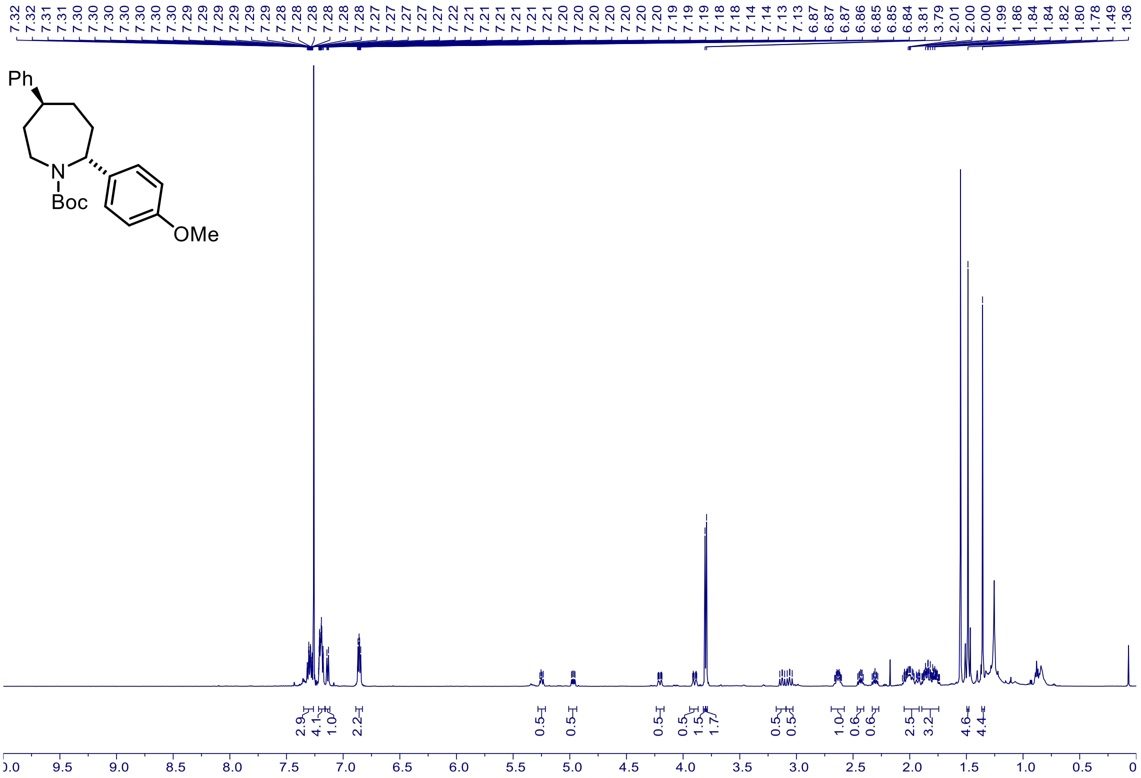

10a – ^{13}C NMR (151 MHz, $CDCl_3$)

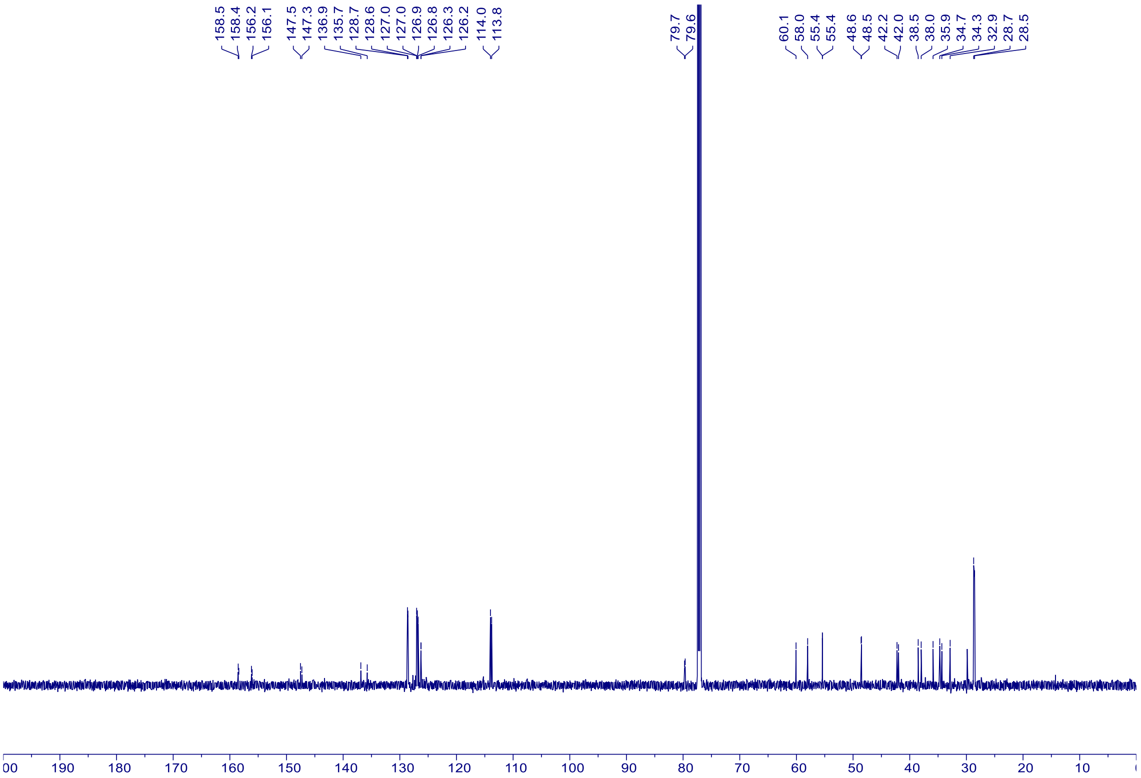

11a – ¹H NMR (600 MHz, CDCl$_3$)

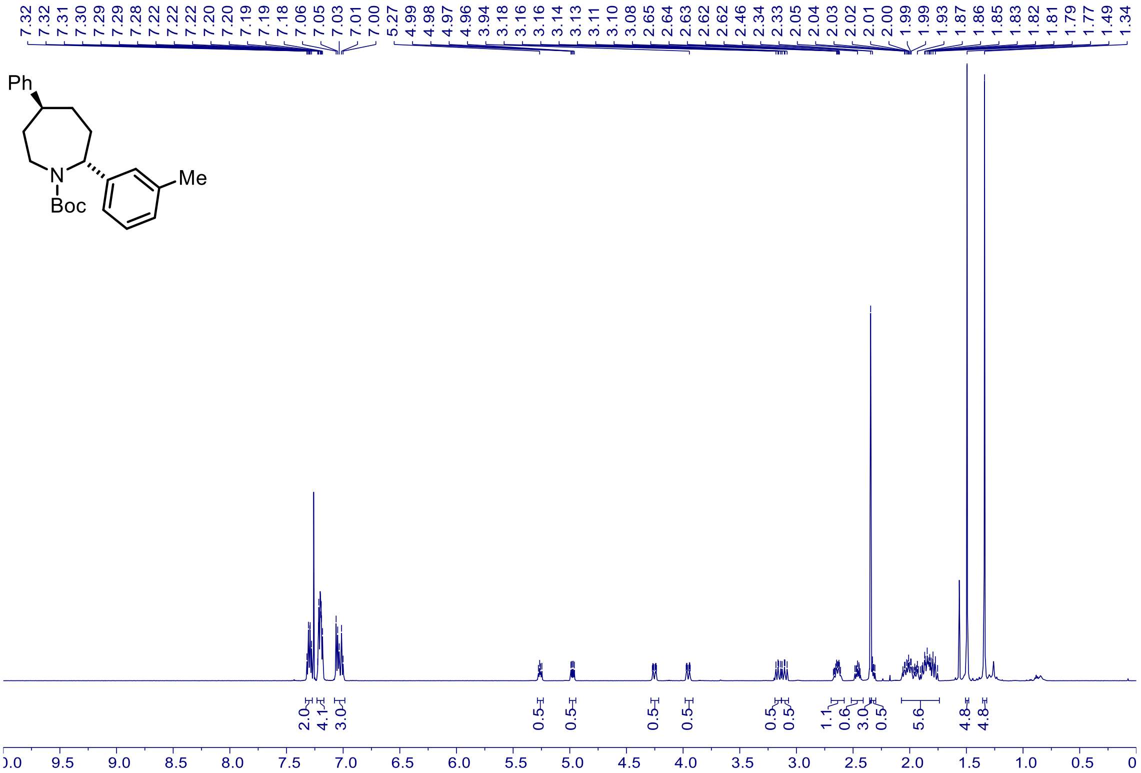

11a – ¹³C NMR (151 MHz, CDCl$_3$)

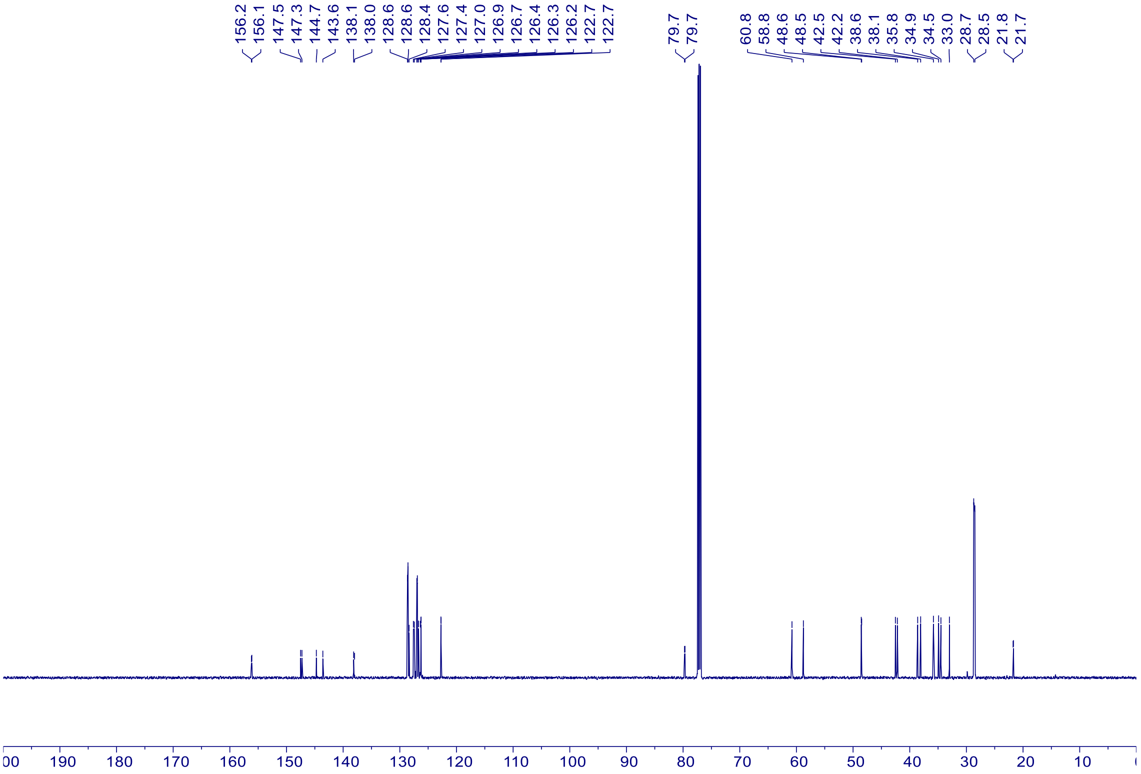

12a – ^{1}H NMR (600 MHz, CDCl$_3$)

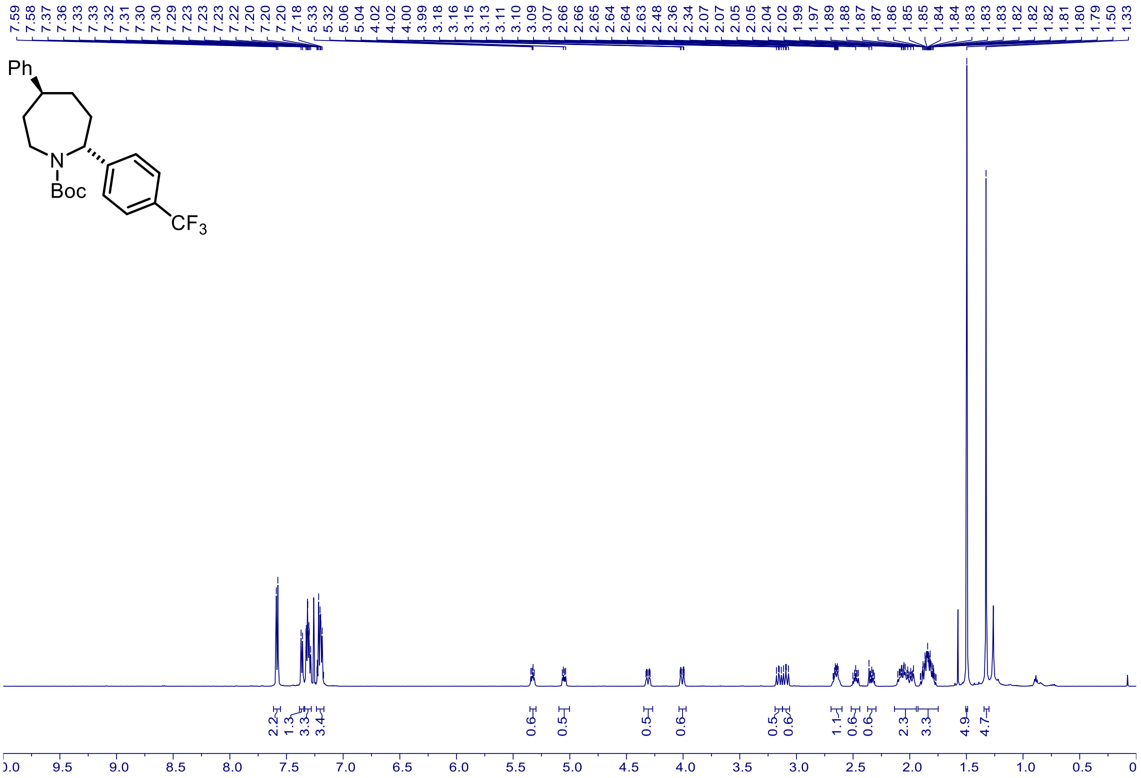

12a – ^{13}C NMR (151 MHz, CDCl$_3$)

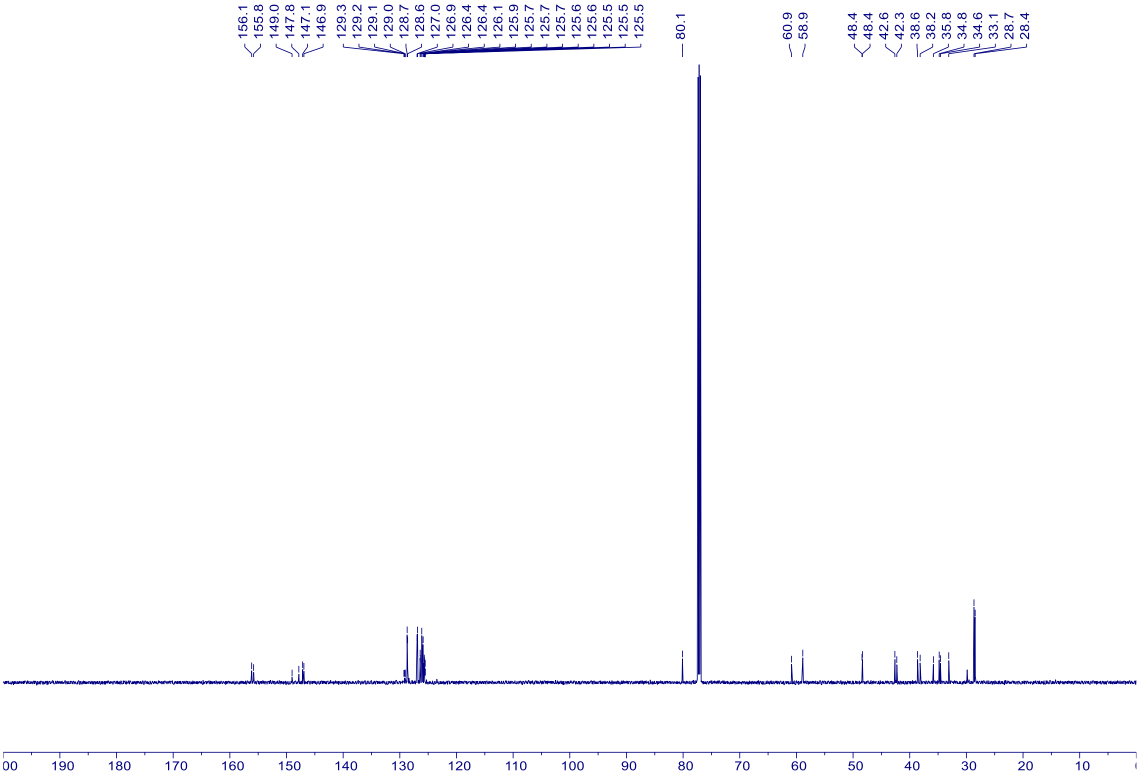

12a – ^{19}F NMR (565 MHz, $CDCl_3$)

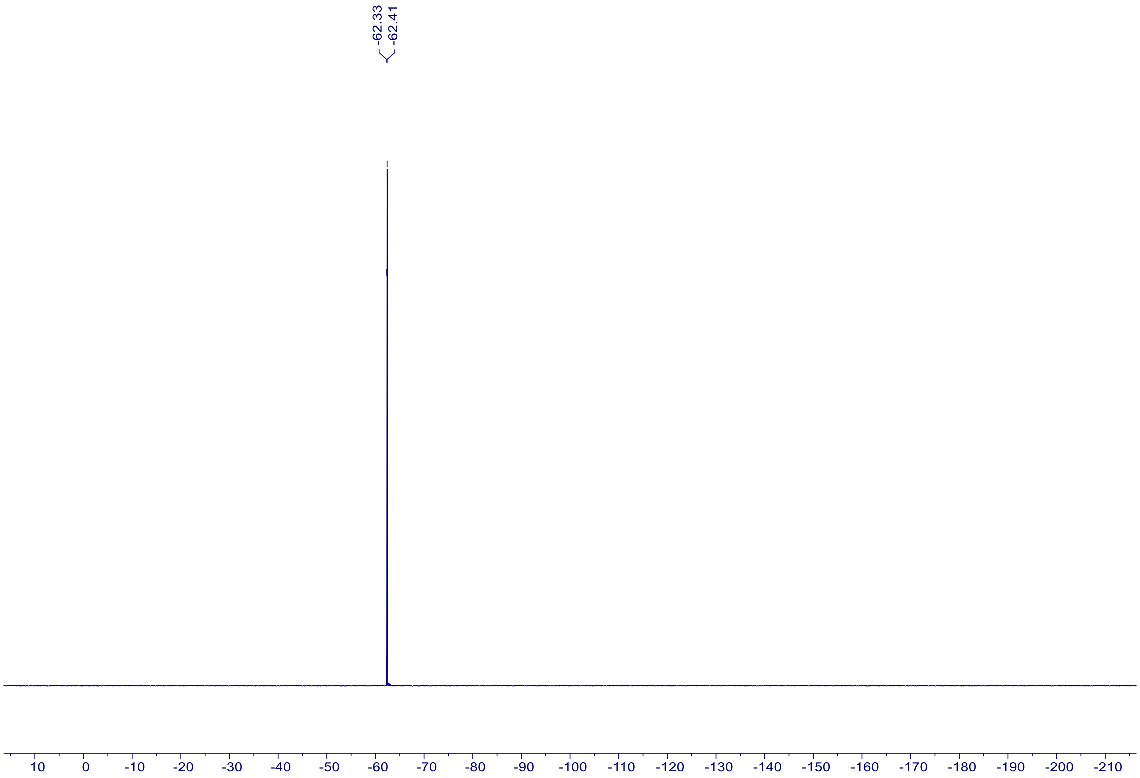

13a – ^{1}H NMR (600 MHz, CDCl$_3$)

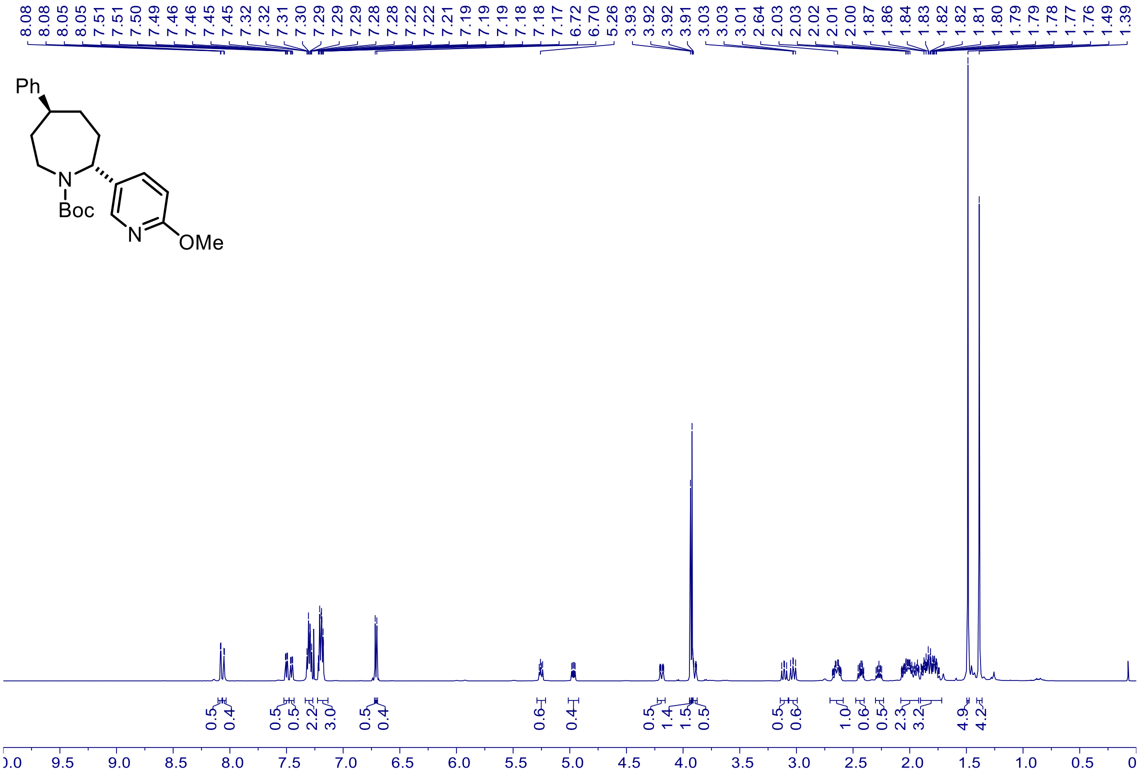

13a – ^{13}C NMR (151 MHz, CDCl$_3$)

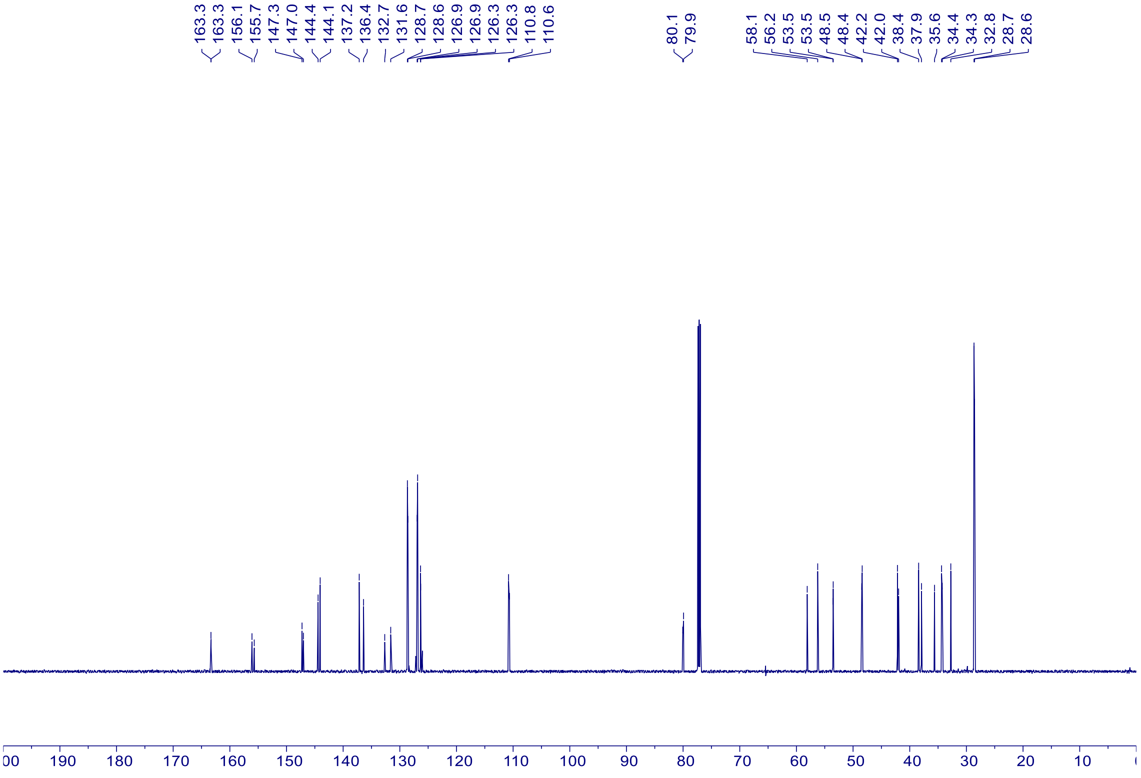

14a – ^{1}H NMR (600 MHz, $CDCl_3$)

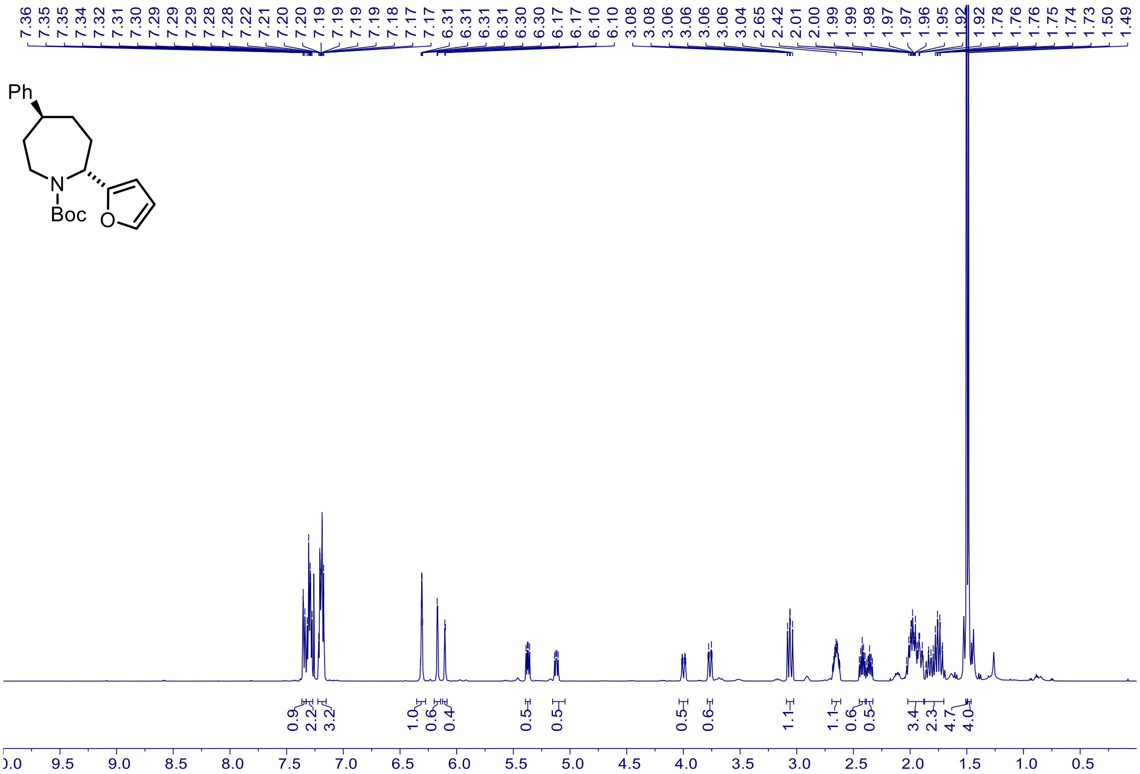

14a – ^{13}C NMR (151 MHz, $CDCl_3$)

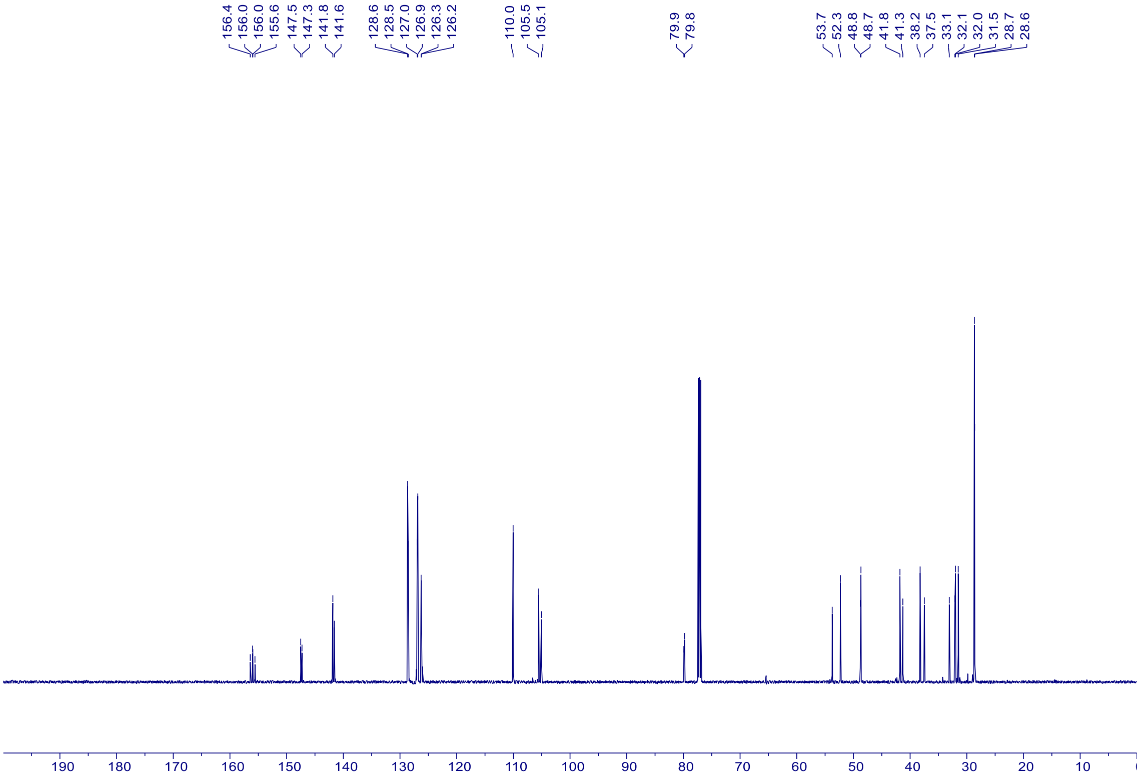

15a – ^{1}H NMR (600 MHz, $CDCl_3$)

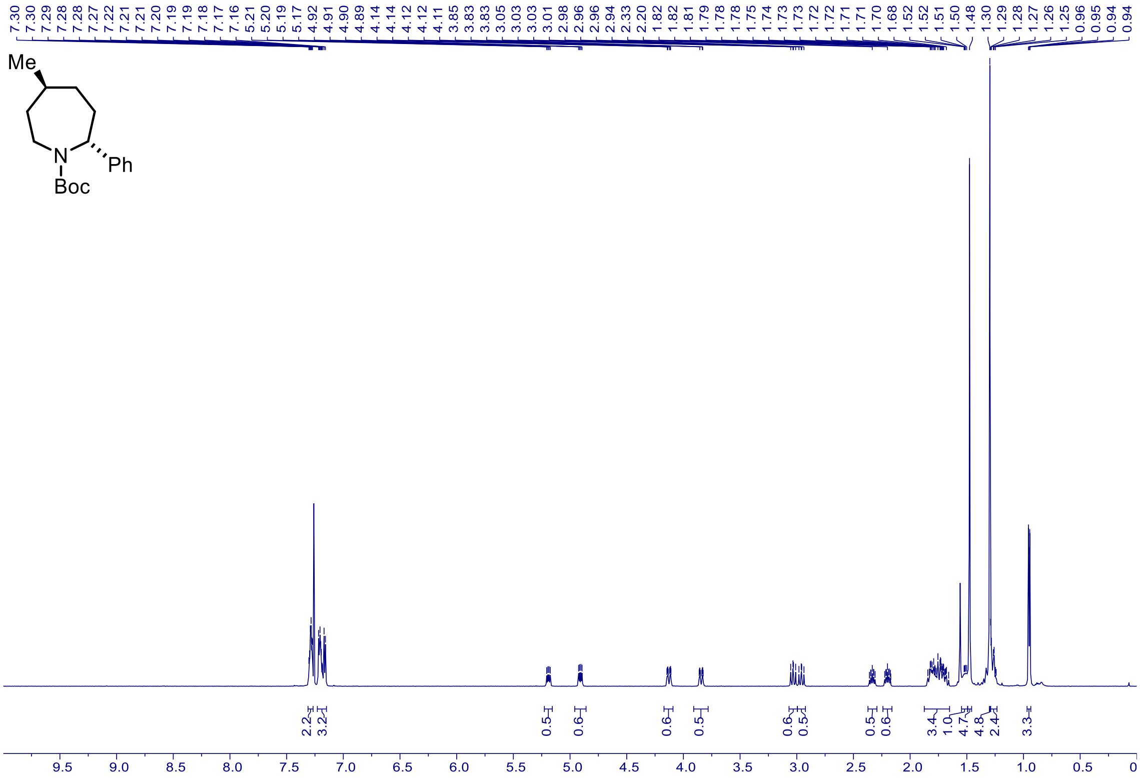

15a – ^{13}C NMR (151 MHz, $CDCl_3$)

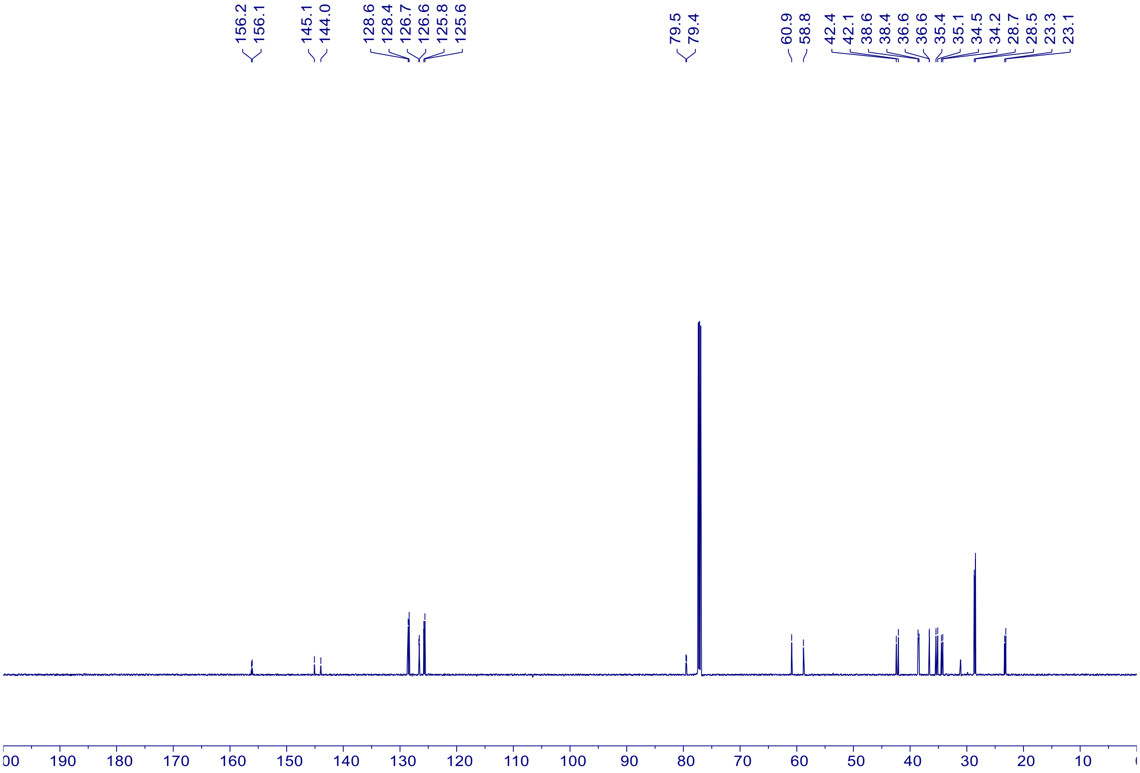

16a – ^{1}H NMR (600 MHz, CDCl$_3$)

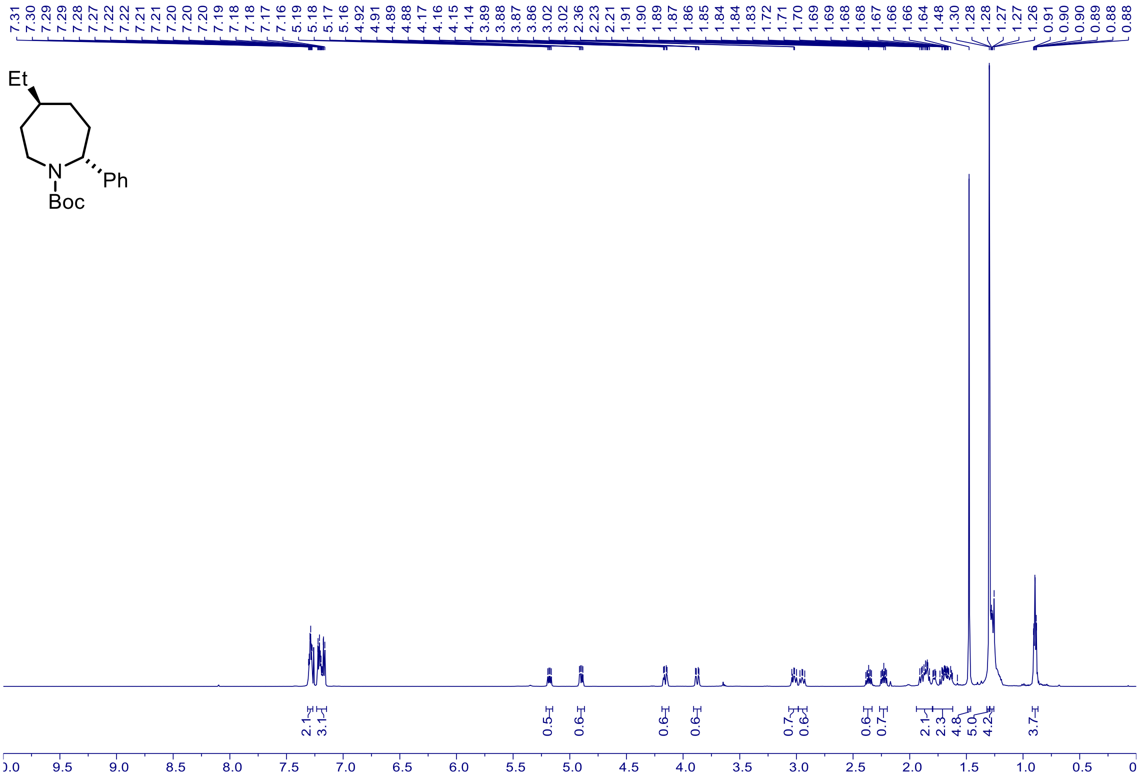

16a – ^{13}C NMR (151 MHz, CDCl$_3$)

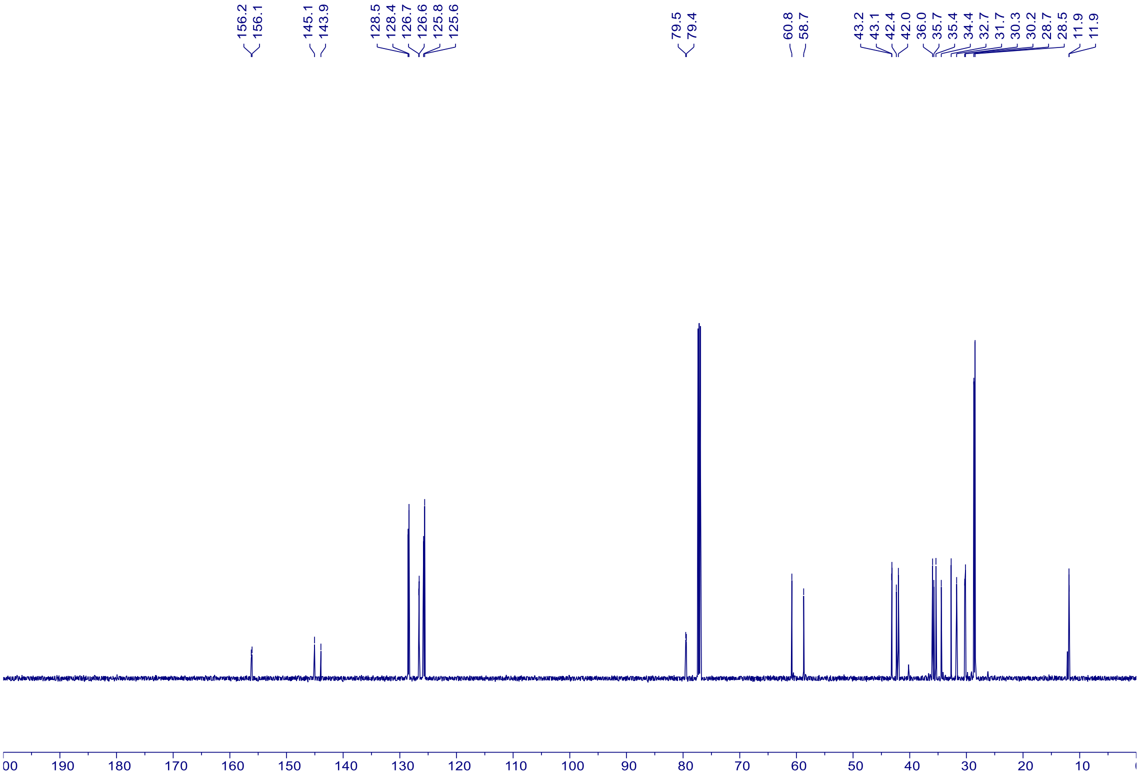

17a – ^{1}H NMR (600 MHz, $CDCl_3$)

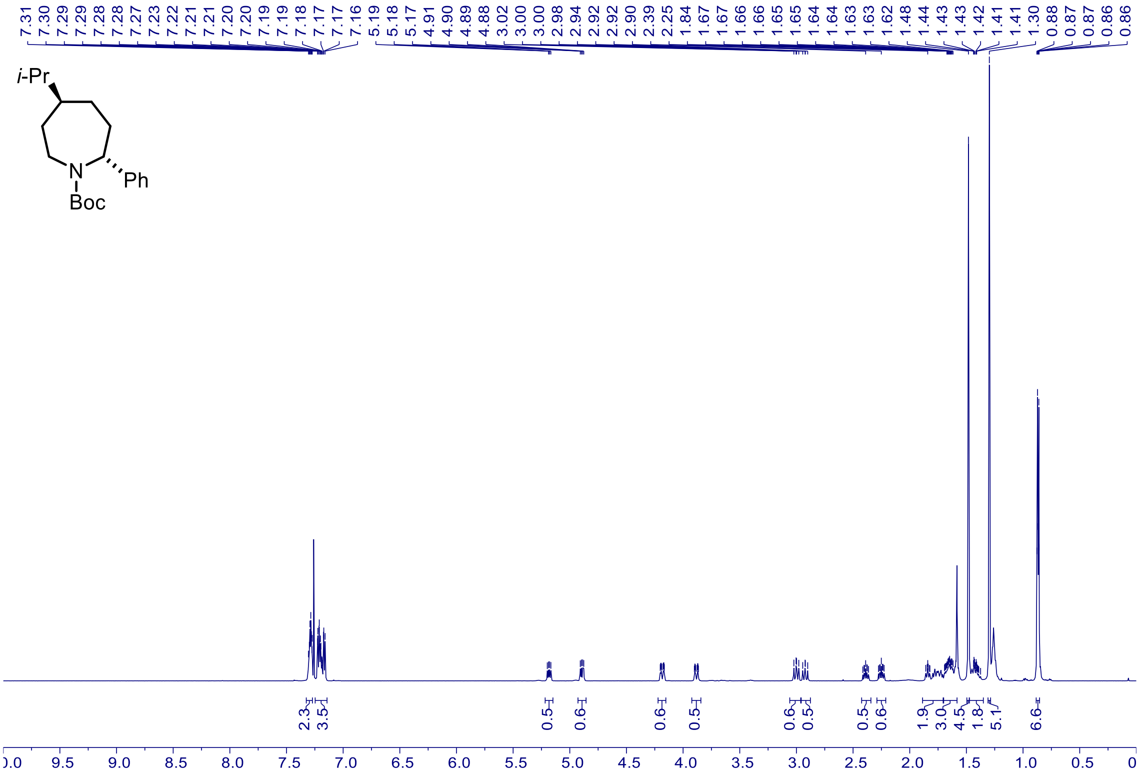

17a – ^{13}C NMR (151 MHz, $CDCl_3$)

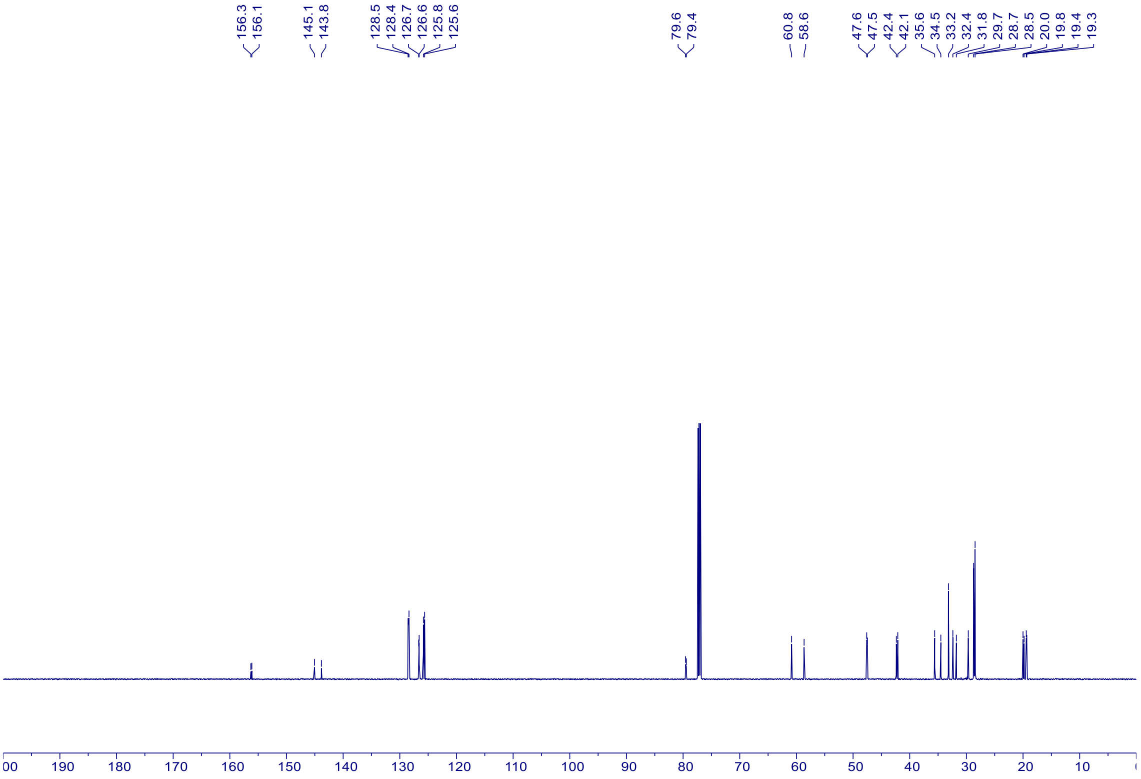

17a – ^{1}H-^{1}H NOESY (600 MHz, $CDCl_3$)

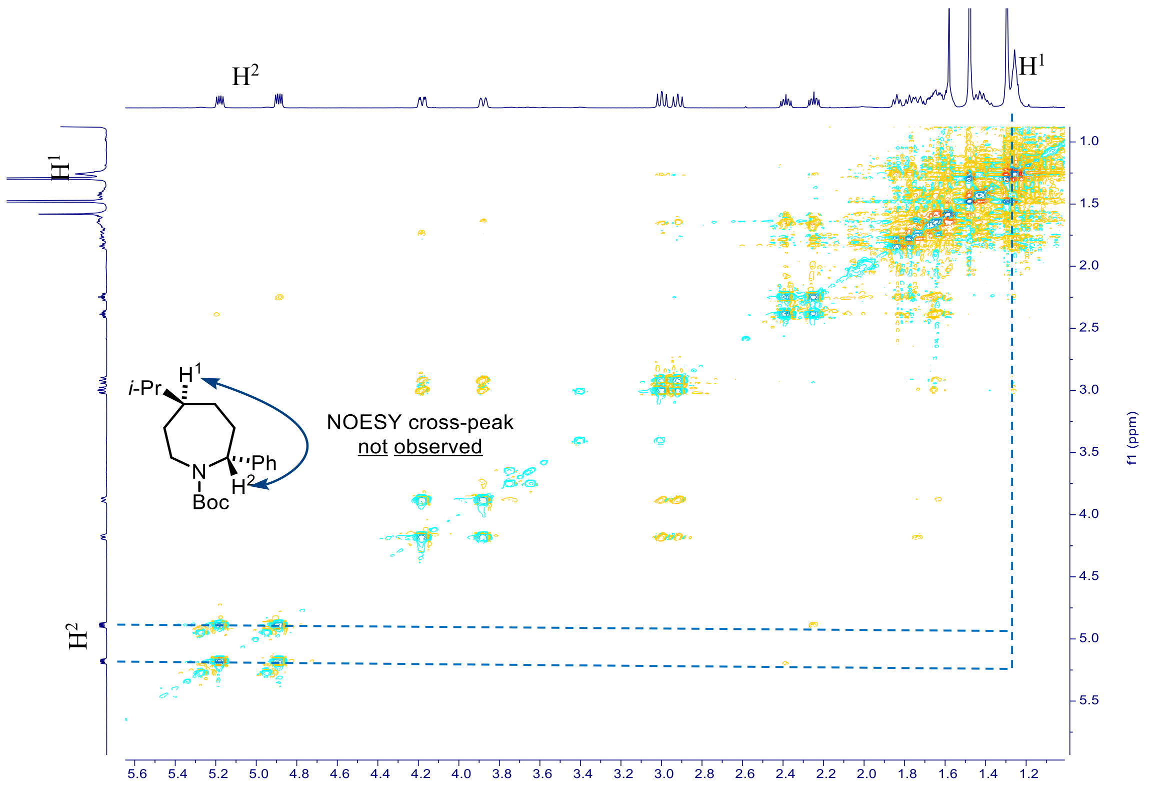

18a – ^{1}H NMR (600 MHz, $CDCl_3$)

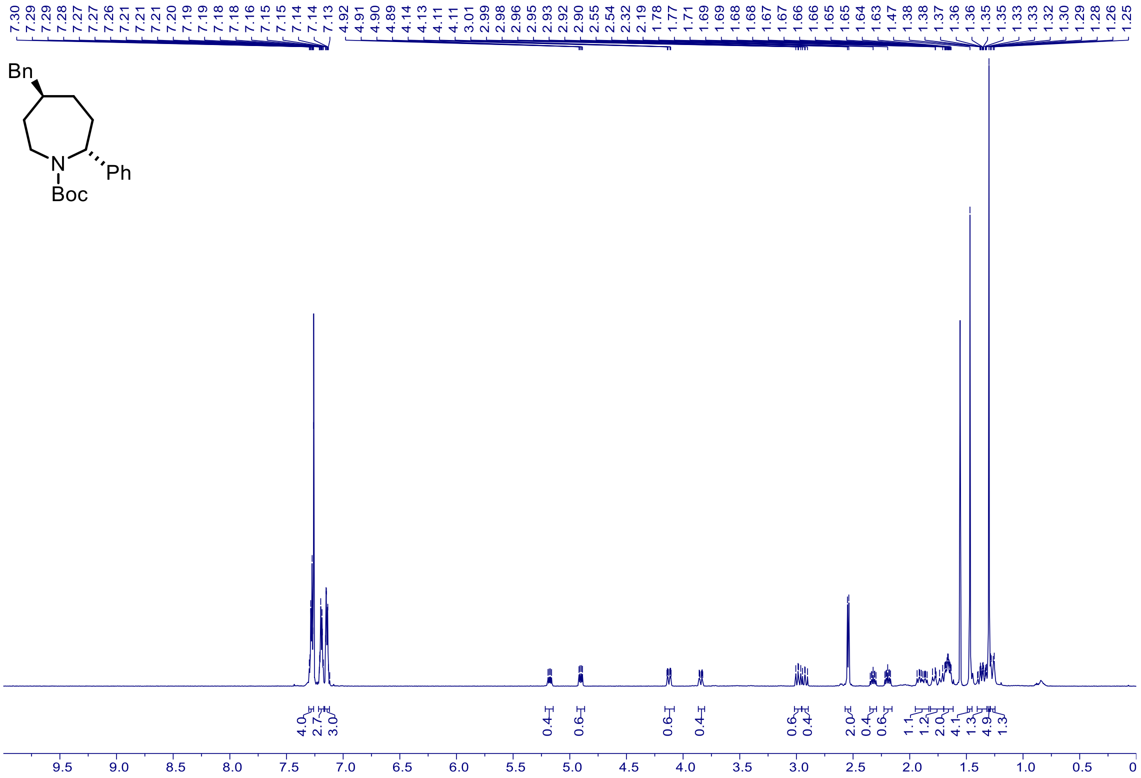

18a – ^{13}C NMR (151 MHz, $CDCl_3$)

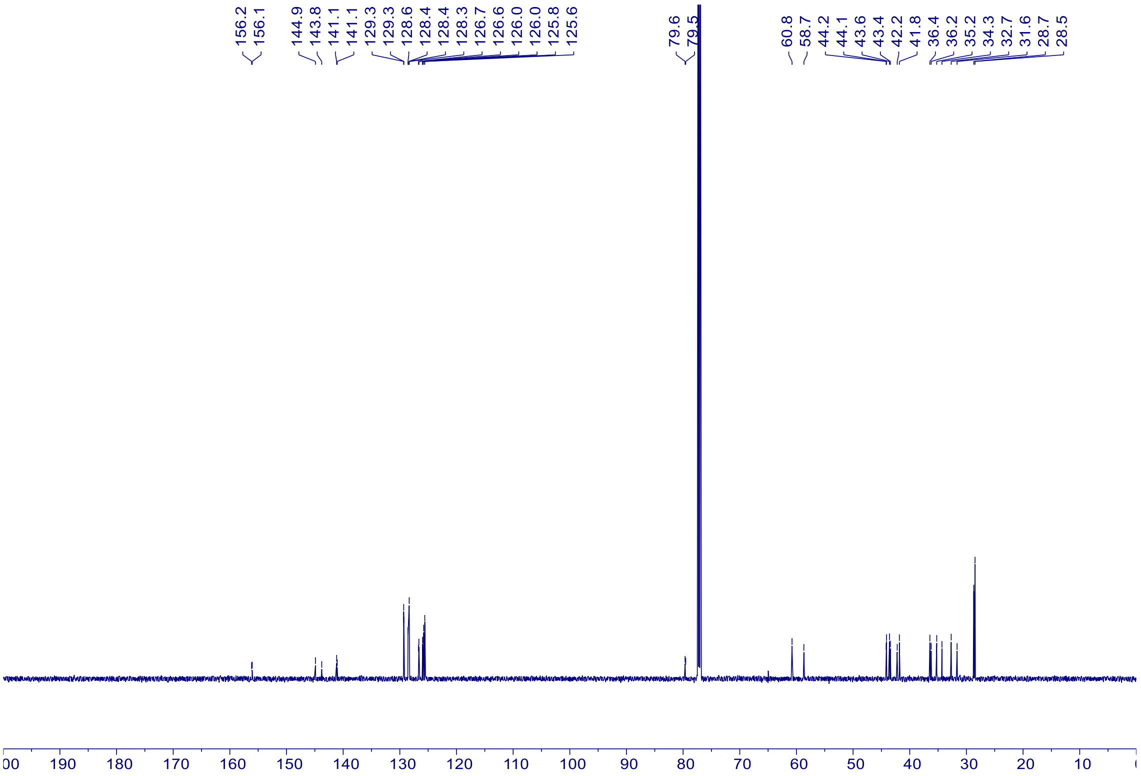

19a – ¹H NMR (600 MHz, $CDCl_3$)

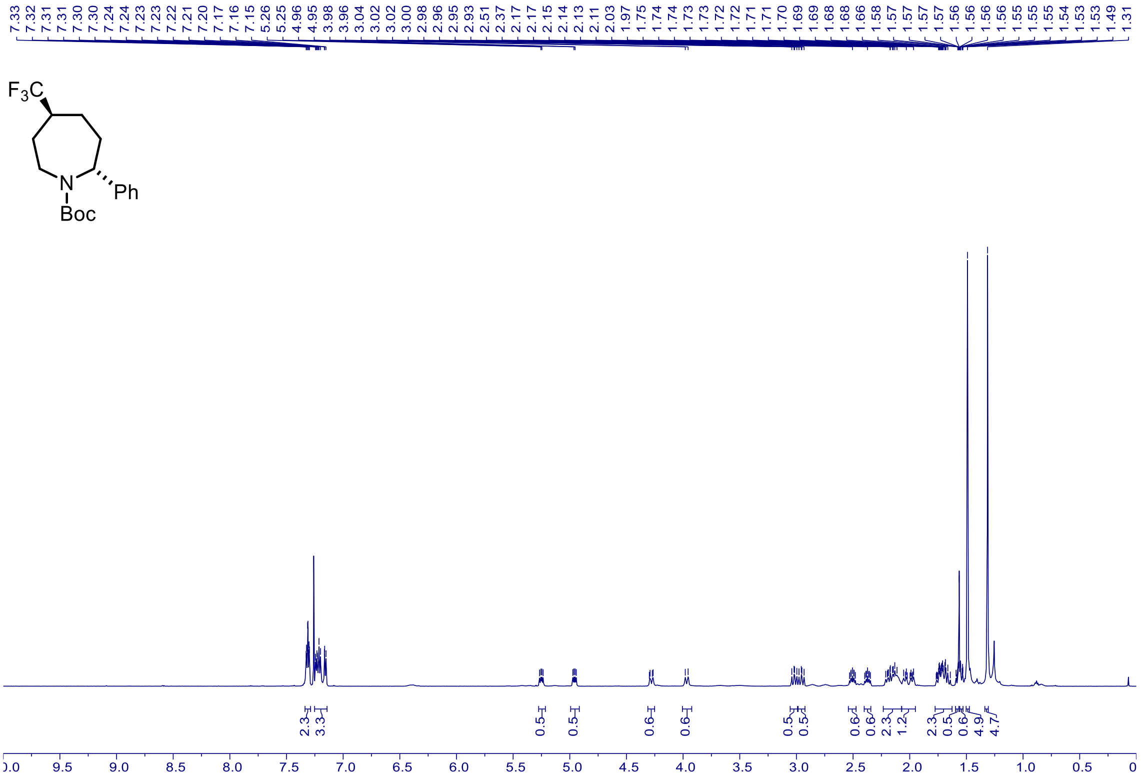

19a – ¹³C NMR (151 MHz, $CDCl_3$)

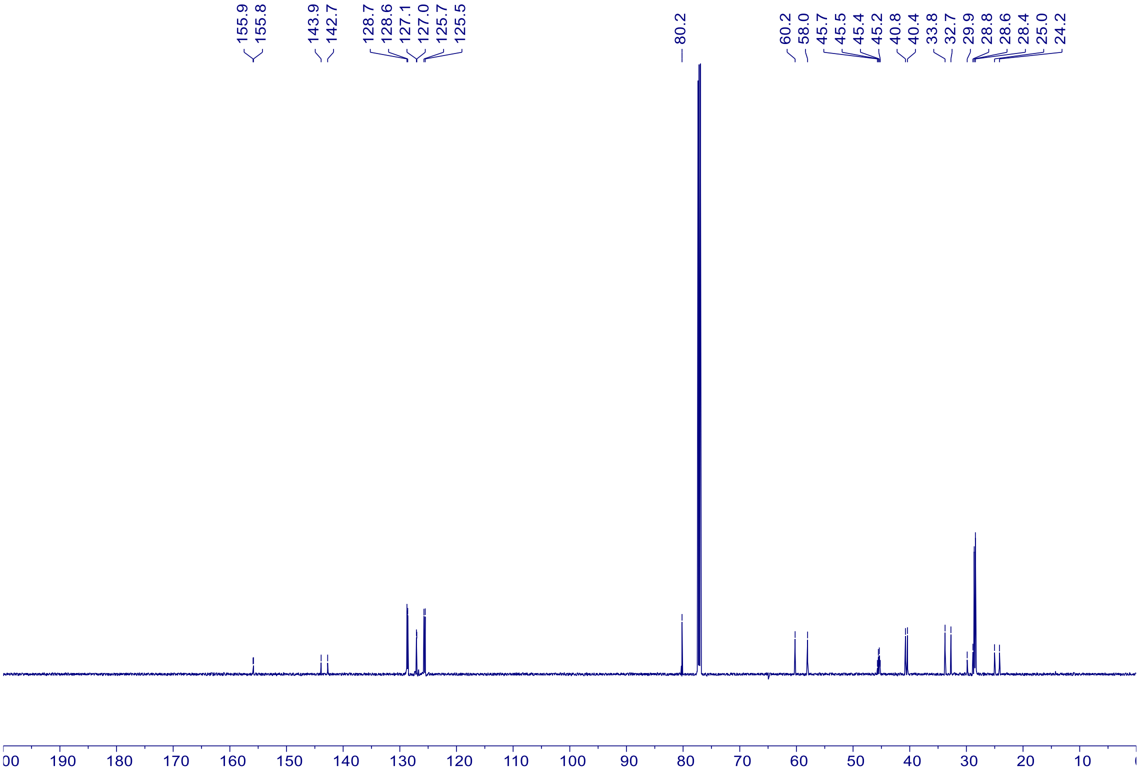

19a – ^{19}F NMR (564 MHz, $CDCl_3$)

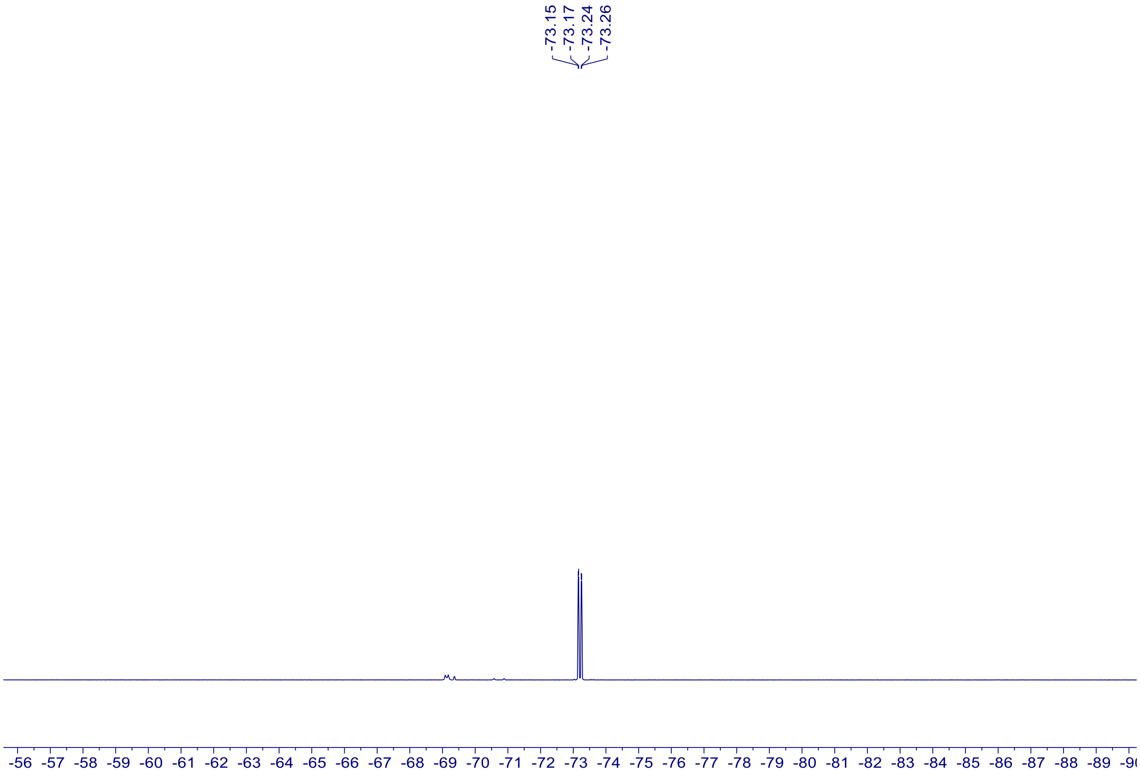

20a – ^{1}H NMR (600 MHz, $CDCl_3$)

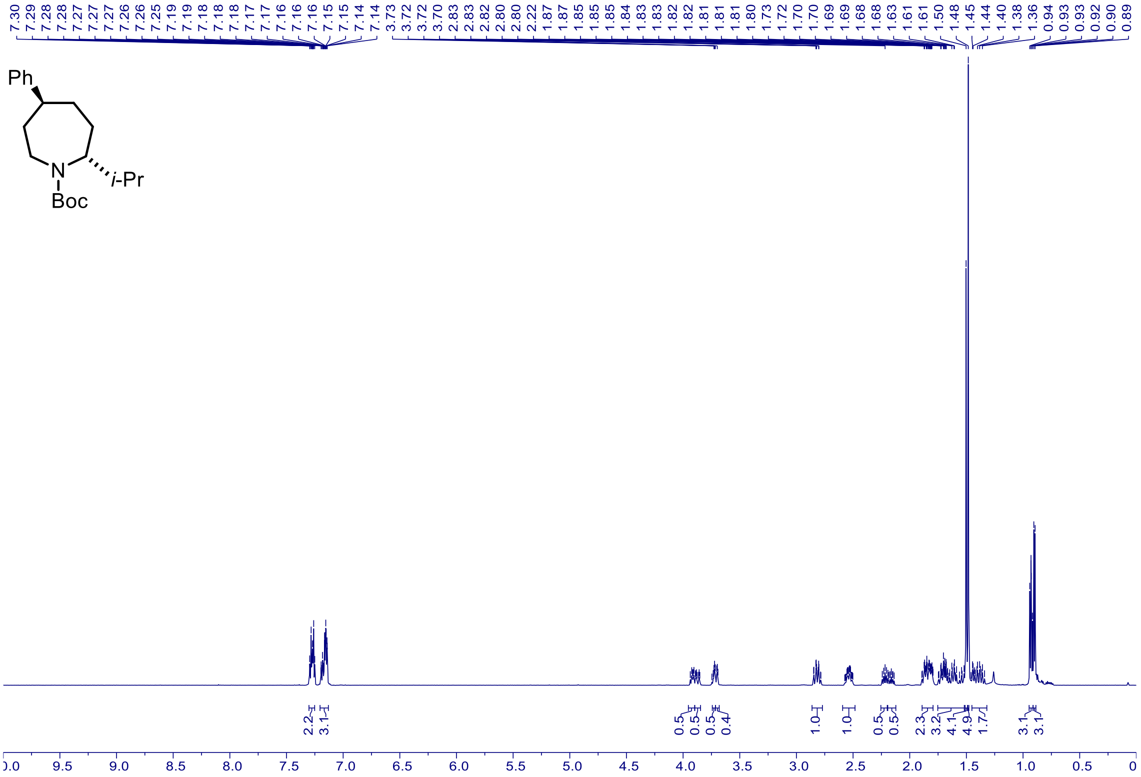

20a – ^{13}C NMR (151 MHz, $CDCl_3$)

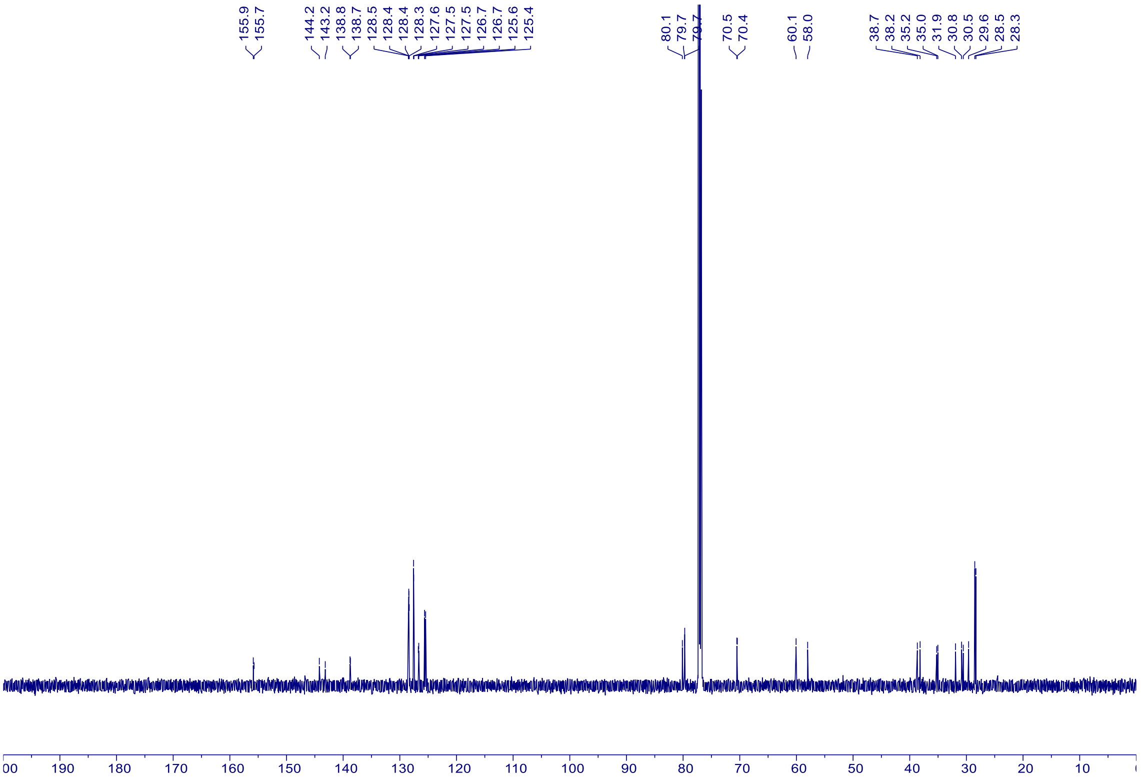

20a – ^{1}H-^{1}H NOESY (600 MHz, $CDCl_3$)

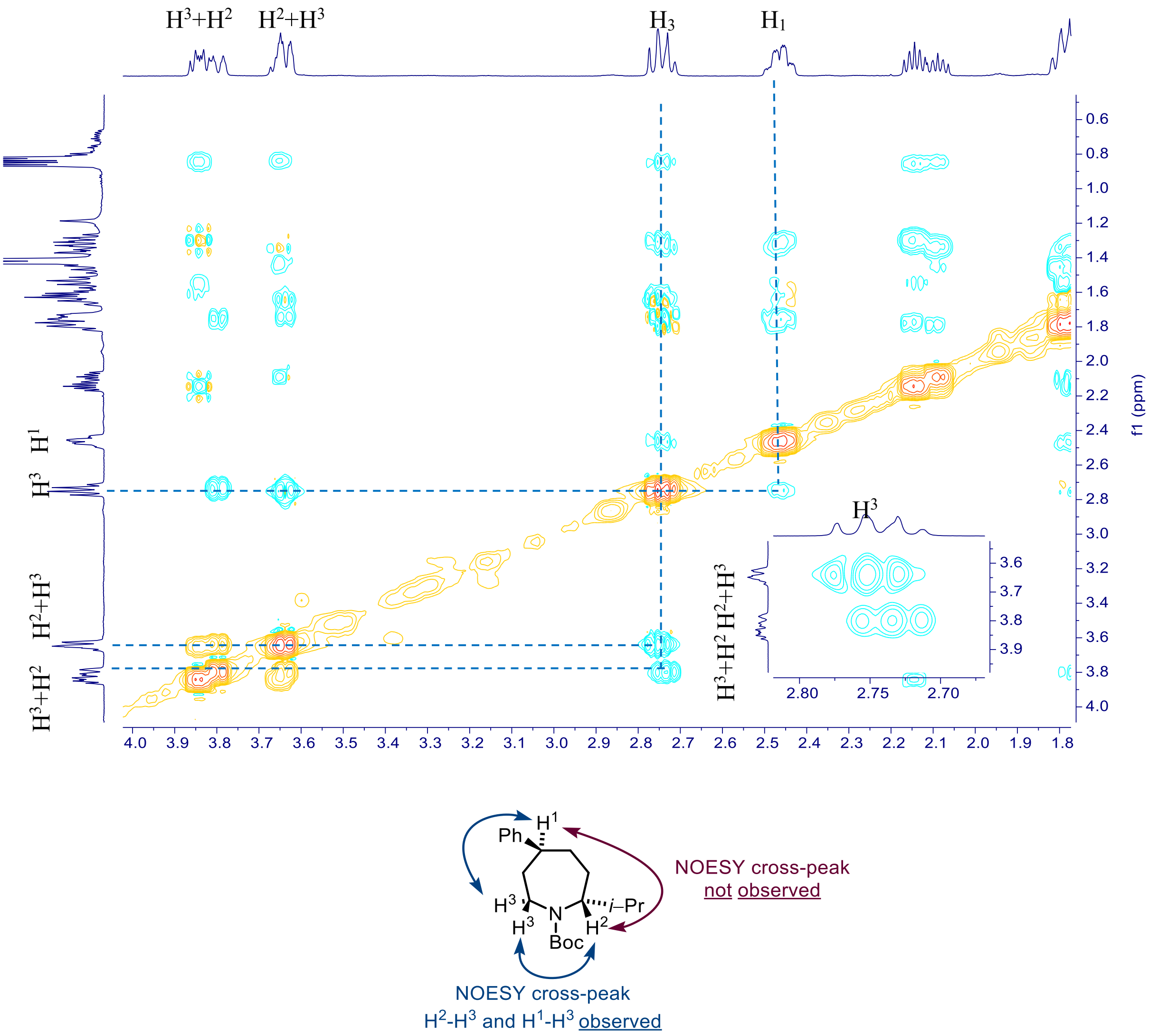

21a – ¹H NMR (600 MHz, CDCl₃)

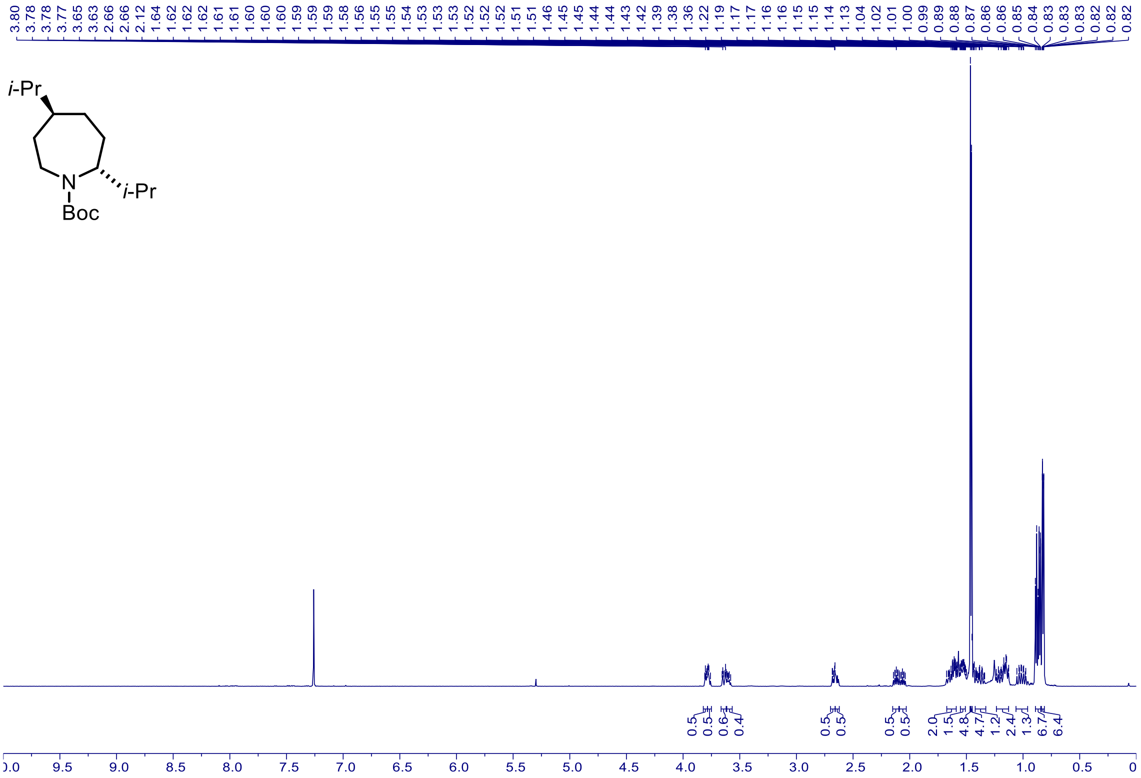

21a – ¹³C NMR (151 MHz, CDCl₃)

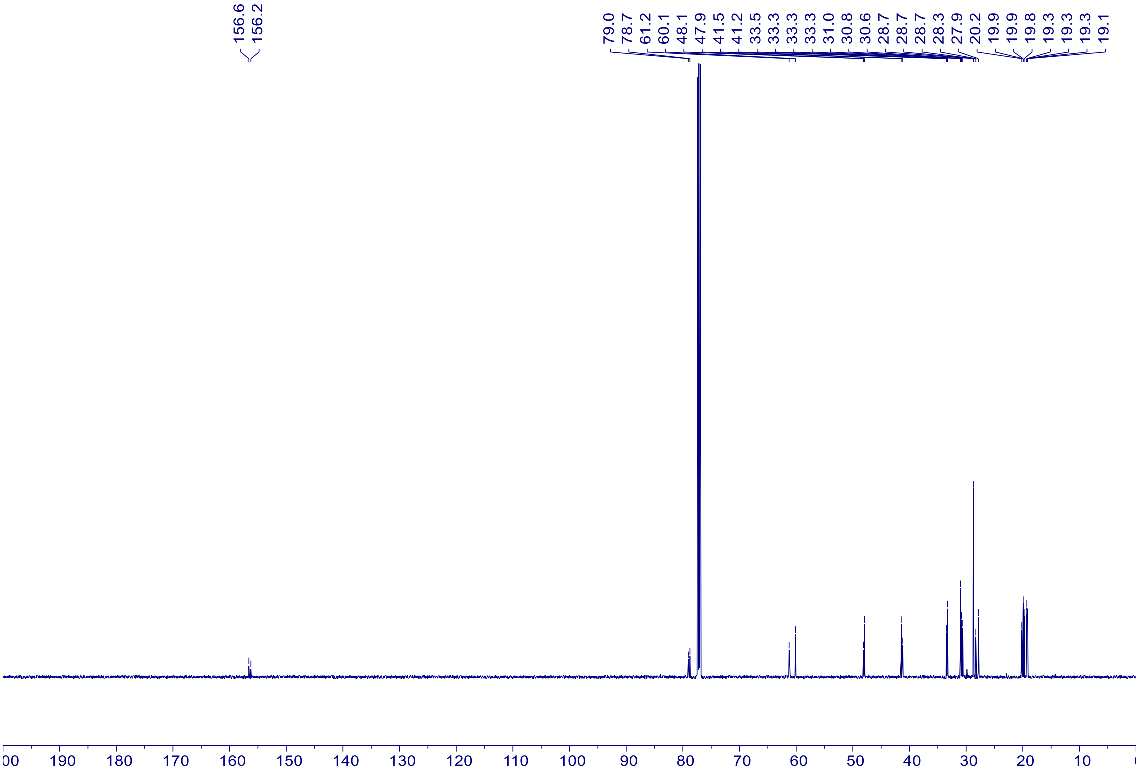

22a – ^{1}H NMR (600 MHz, $CDCl_3$)

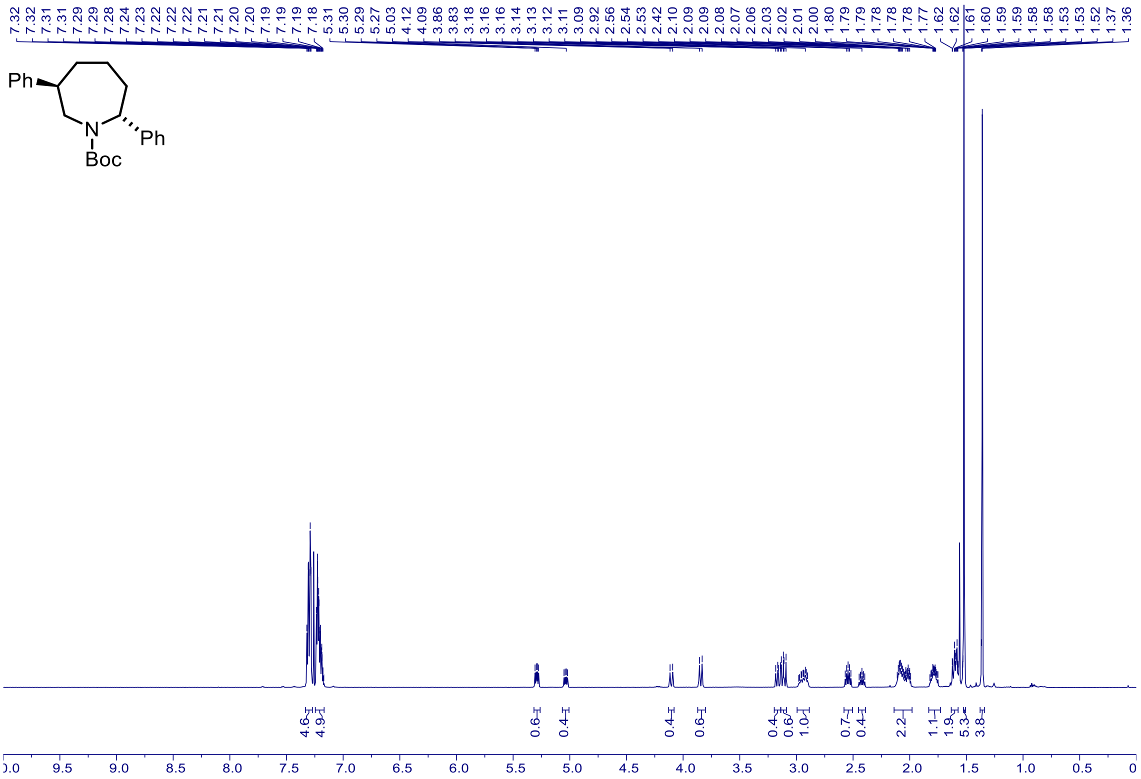

22a – ^{13}C NMR (151 MHz, $CDCl_3$)

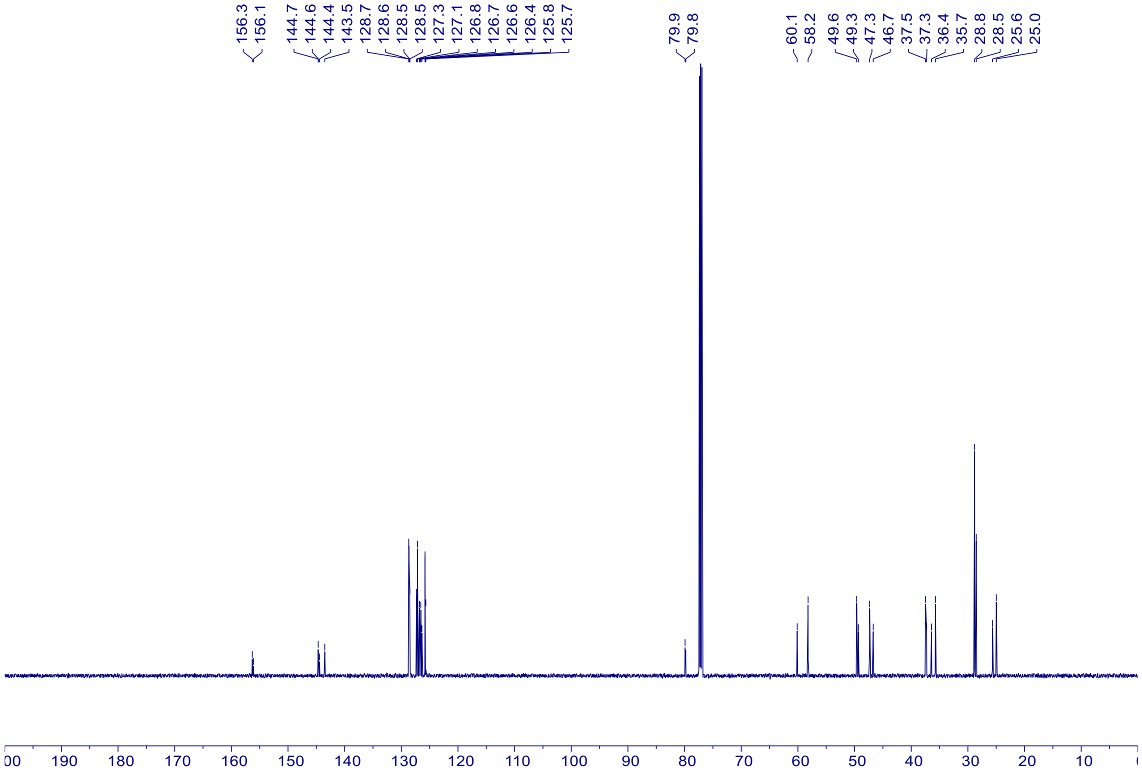

22a – ^{1}H-^{1}H NOESY (600 MHz, CDCl$_3$)

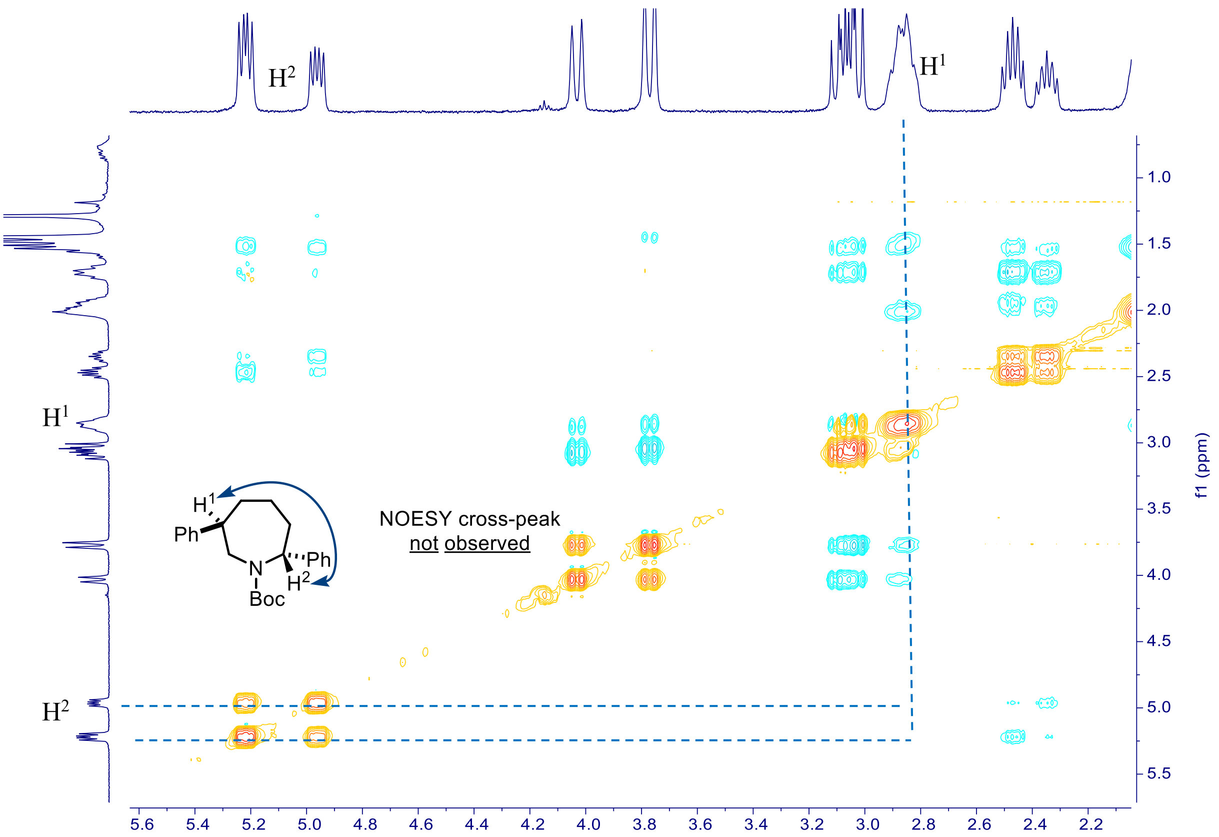

23a – ^{1}H NMR (600 MHz, CDCl$_3$)

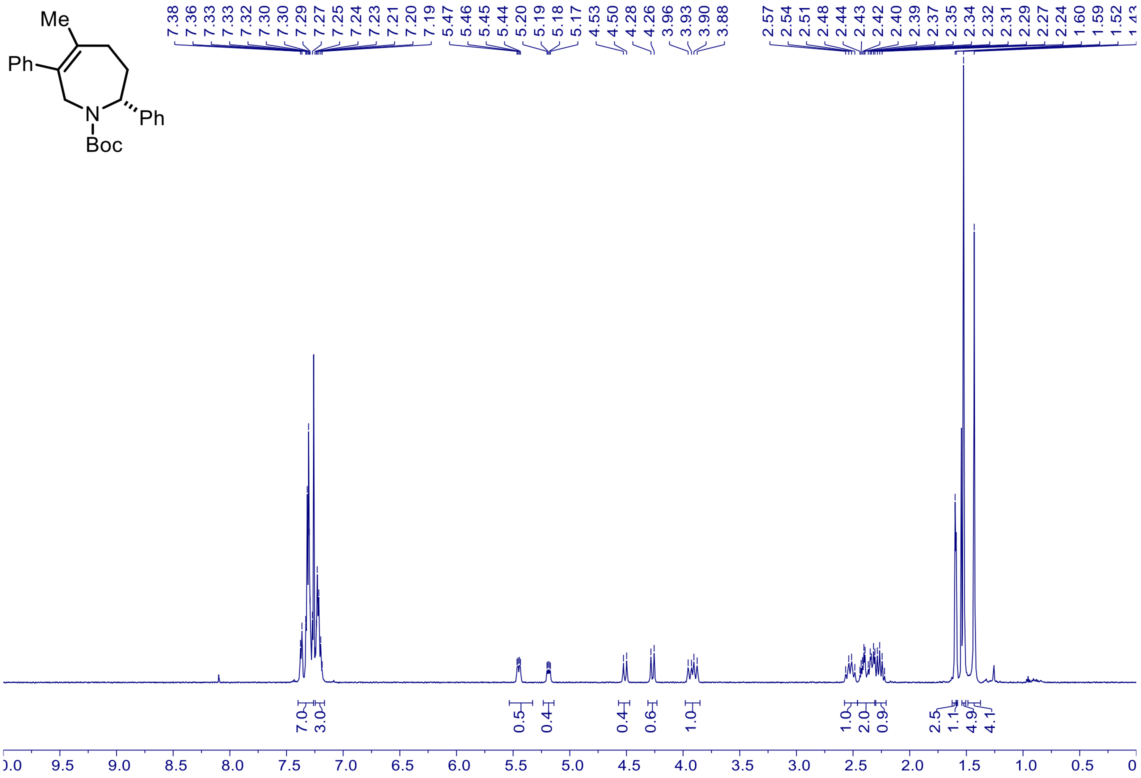

23a – ^{13}C NMR (151 MHz, CDCl$_3$)

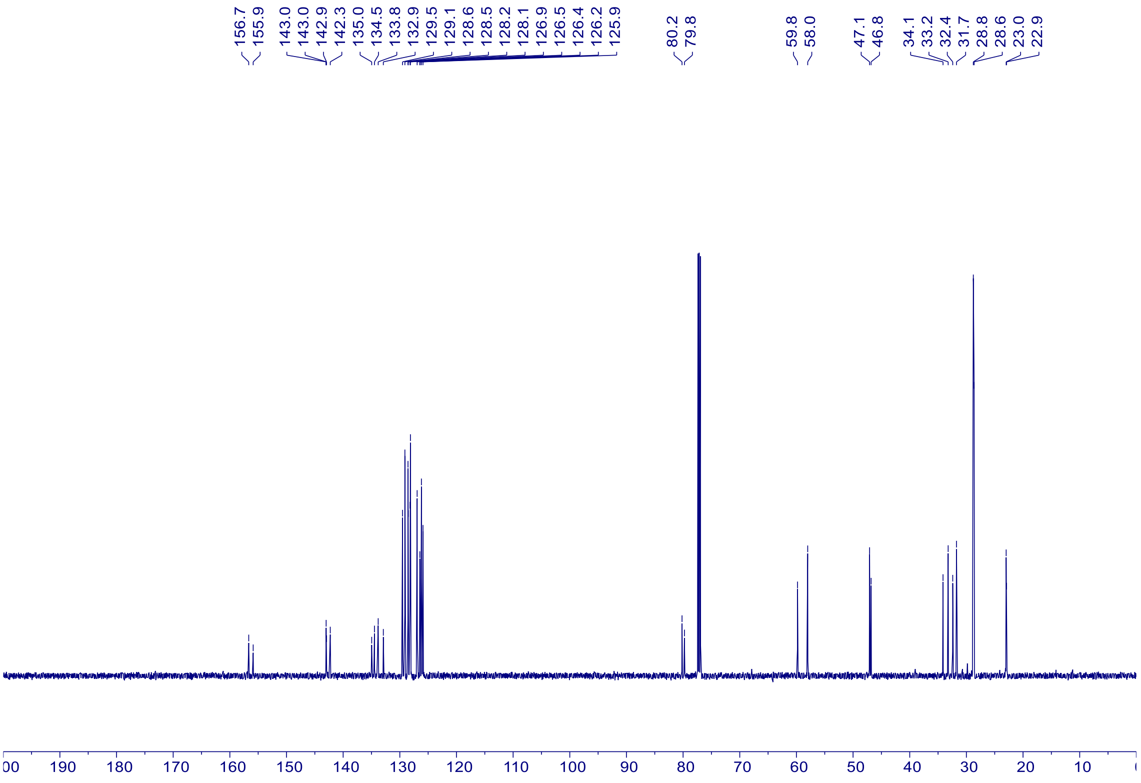

S19a – ^{1}H NMR (600 MHz, $CDCl_3$)

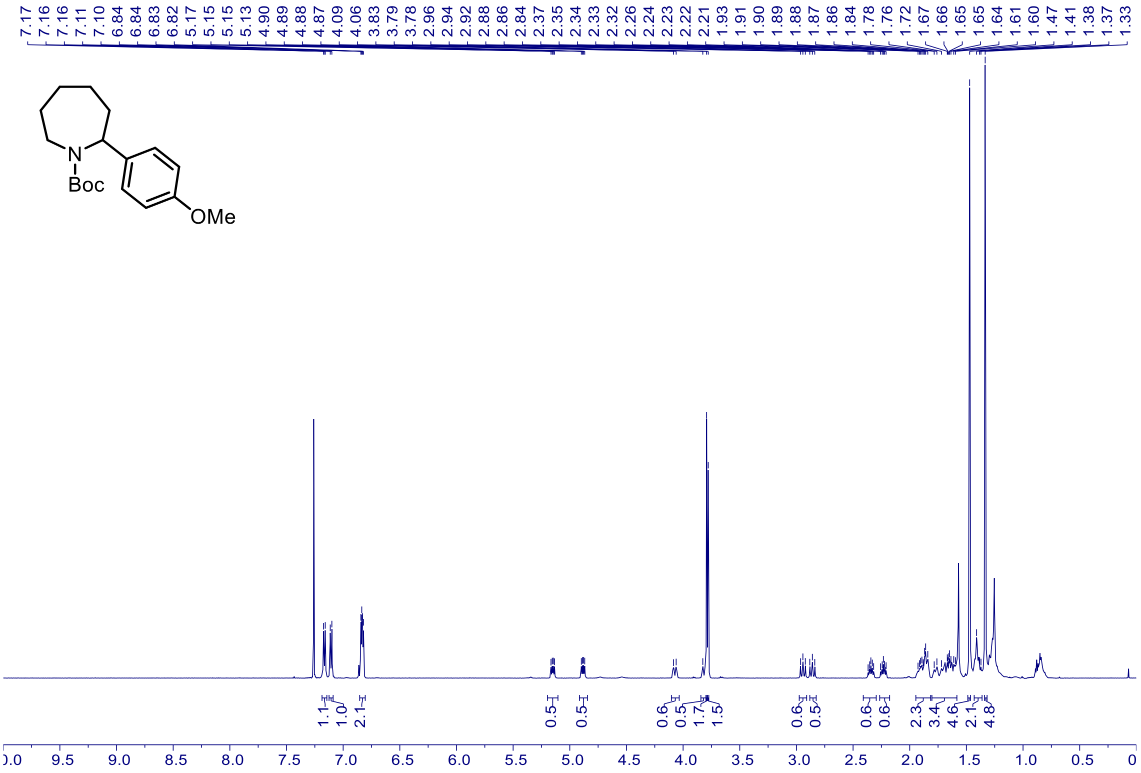

S19a – ^{13}C NMR (151 MHz, $CDCl_3$)

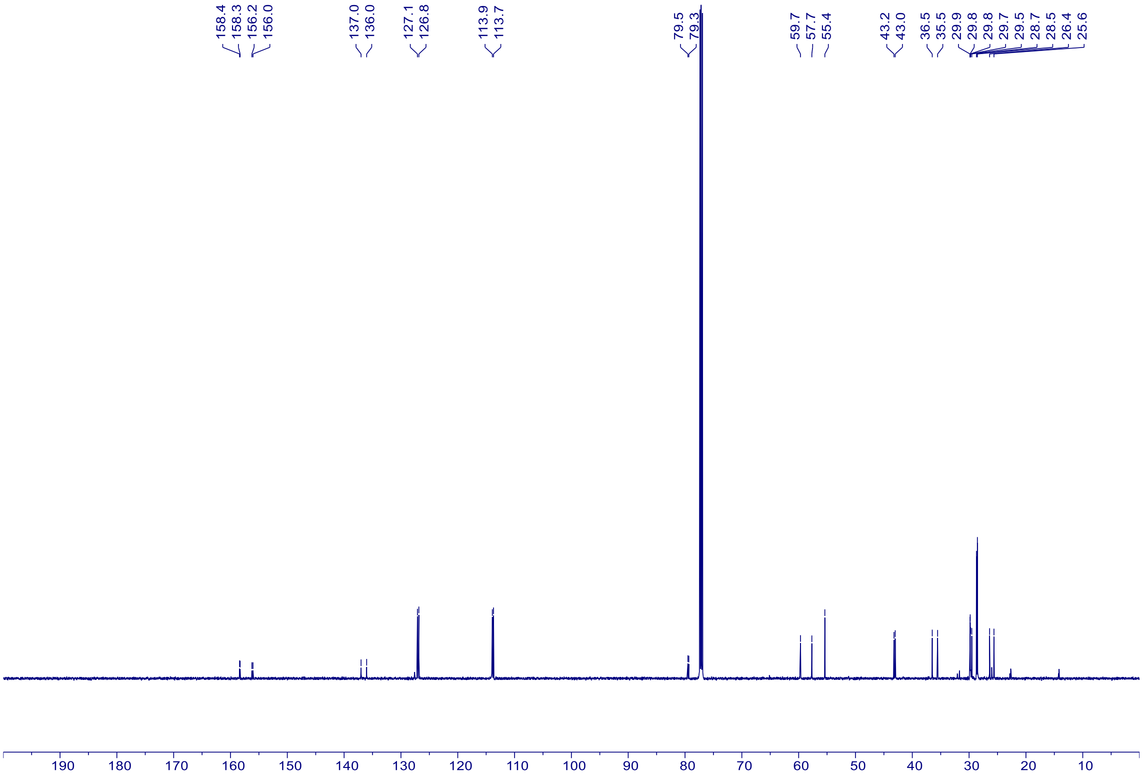

13 References

(1) Li, B.; Ruffoni, A.; Leonori, D. A Photochemical Strategy for Ortho-Aminophenol Synthesis via Dearomative-Rearomative Coupling Between Aryl Azides and Alcohols. *Angew. Chem. Int. Ed.* **2023**, *62* (52), e202310540.

(2) Li, Y. Zhuo.; Kirby, J. P.; George, M. W.; Poliakoff, Martyn.; Schuster, G. B. 1,2-Didehydroazepines from the Photolysis of Substituted Aryl Azides: Analysis of Their Chemical and Physical Properties by Time-Resolved Spectroscopic Methods. *J. Am. Chem. Soc.* **1988**, *110* (24), 8092–8098.

(3) Sundberg, R. J.; Suter, S. R.; Brenner, M. Photolysis of 0-Substituted Aryl Azides in Diethylamine. Formation and Autoxidation of 2-Diethylamino-1H-Azepine Intermediates. *J. Am. Chem. Soc.* **1972**, *94* (2), 513–520.

(4) Matador, E.; Tilby, M. J.; Saridakis, I.; Pedrón, M.; Tomczak, D.; Llaveria, J.; Atodiresei, I.; Merino, P.; Ruffoni, A.; Leonori, D. A Photochemical Strategy for the Conversion of Nitroarenes into Rigidified Pyrrolidine Analogues. *J. Am. Chem. Soc.* **2023**, *145* (50), 27810–27820.

(5) Mykura, R.; Sánchez-Bento, R.; Matador, E.; Duong, V. K.; Varela, A.; Angelini, L.; Carbajo, R. J.; Llaveria, J.; Ruffoni, A.; Leonori, D. Synthesis of Polysubstituted Azepanes by Dearomative Ring Expansion of Nitroarenes. *Nat. Chem.* **2024**, *16* (5), 771–779.

(6) Li, G.; Lavagnino, M. N.; Ali, S. Z.; Hu, S.; Radosevich, A. T. Tandem C/N-Difunctionalization of Nitroarenes: Reductive Amination and Annulation by a Ring Expansion/Contraction Sequence. *J. Am. Chem. Soc.* **2023**, *145* (1), 41–46.

(7) Kellogg, R. M.; Van Bergen T. J. Ring Expansion of a 1,2-Dihydropyridine to an Azepine. *J. Org. Chem.* **1971**, *36* (7), 978–983.

(8) Krasovskiy, A.; Knochel, P. Convenient Titration Method for Organometallic Zinc, Magnesium, and Lanthanide- Reagents. *Synthesis* **2006**, *2006* (05), 0890–0891.

(9) Arnold, L. A.; Imbos, R.; Mandoli, A.; De Vries, A. H. M.; Naasz, R.; Feringa, B. L. Enantioselective Catalytic Conjugate Addition of Dialkylzinc Reagents Using Copper–Phosphoramidite Complexes; Ligand Variation and Non-Linear Effects. *Tetrahedron* **2000**, *56* (18), 2865–2878.

(10) Smith, C. R.; Mans, D. J.; RajanBabu, T. V. (R)-2,2'-BINAPHTHOYL-(S,S)-DI(1-PHENYLETHYL) AMINOPHOSPHINE. SCALABLE PROTOCOLS FOR THE SYNTHESES OF PHOSPHORAMIDITE (FERINGA) LIGANDS. *Org. Synth.* **2008**, *85*, 238.

(11) Lefort, L.; Boogers, J. A. F.; de Vries, J. G.; de Vries, A. H. M. Under Pressure: Rapid Development of Scalable Asymmetric Hydrogenation Catalysts. *Top. Catal.* **2010**, *53* (15), 1081–1086.

(12) Bernsmann, H.; van den Berg, M.; Hoen, R.; Minnaard, A. J.; Mehler, G.; Reetz, M. T.; De Vries, J. G.; Feringa, B. L. PipPhos and MorfPhos: Privileged Monodentate Phosphoramidite Ligands for Rhodium-Catalyzed Asymmetric Hydrogenation. *J. Org. Chem.* **2005**, *70* (3), 943–951.

(13) Harada, H.; Thalji, R. K.; Bergman, R. G.; Ellman, J. A. Enantioselective Intramolecular Hydroarylation of Alkenes via Directed C−H Bond Activation. *J. Org. Chem.* **2008**, *73* (17), 6772–6779.

(14) Du, H.; Yuan, W.; Zhao, B.; Shi, Y. Catalytic Asymmetric Diamination of Conjugated Dienes and Triene. *J. Am. Chem. Soc.* **2007**, *129* (38), 11688–11689.

(15) Albat, D.; Köcher, A.; Witt, J.; Schmalz, H.-G. On the Asymmetric Iridium-Catalyzed N-Allylation of Amino Acid Esters: Improved Selectivities through Structural Variation of the Chiral Phosphoramidite Ligand. *Eur. J. Org. Chem.* **2022**, *2022* (12), e202200188.

(16) Pizzuti, M. G.; Minnaard, A. J.; Feringa, B. L. Catalytic Enantioselective Addition of Organometallic Reagents to *N* -Formylimines Using Monodentate Phosphoramidite Ligands. *J. Org. Chem.* **2008**, *73* (3), 940–947.
(17) Farrugia, L. J. WinGX and ORTEP for Windows: An Update. *J. Appl. Crystallogr.* **2012**, *45* (4), 849–854.
(18) Bruker D8 Adventure Diffractometer – Purchased with Financial Support from DFG, German Research Foundation (Grant No. 527802652), Ministerium Für Kultur Und Wissenschaft Des Landes Nordrhein-Westfalen (MKW, Ministry of Culture and Science of the State of North Rhine-Westphalia) and RWTH Aachen University.
(19) Sheldrick, G. M. SHELXT – Integrated Space-Group and Crystal-Structure Determination. *Acta Crystallogr. Sect. Found. Adv.* **2015**, *71* (1), 3–8.
(20) Sheldrick, G. M. Crystal Structure Refinement with SHELXL. *Acta Crystallogr. Sect. C Struct. Chem.* **2015**, *71* (1), 3–8.
(21) Dolomanov, O. V.; Bourhis, L. J.; Gildea, R. J.; Howard, J. a. K.; Puschmann, H. OLEX2: A Complete Structure Solution, Refinement and Analysis Program. *J. Appl. Crystallogr.* **2009**, *42* (2), 339–341.
(22) Parsons, S.; Flack, H. D.; Wagner, T. Use of Intensity Quotients and Differences in Absolute Structure Refinement. *Acta Crystallogr. Sect. B Struct. Sci. Cryst. Eng. Mater.* **2013**, *69* (3), 249–259.
(23) Hooft, R. W. W.; Straver, L. H.; Spek, A. L. Determination of Absolute Structure Using Bayesian Statistics on Bijvoet Differences. *J. Appl. Crystallogr.* **2008**, *41* (1), 96–103.
(24) SADABS-2016/2 - Bruker AXS Area Detector Scaling and Absorption Correction.
(25) Krause, L.; Herbst-Irmer, R.; Sheldrick, G. M.; Stalke, D. Comparison of Silver and Molybdenum Microfocus X-Ray Sources for Single-Crystal Structure Determination. *J. Appl. Crystallogr.* **2015**, *48* (1), 3–10.
(26) S, A. B.; Bhattacharjee, R.; Gupta, S.; Ahammad, S.; Datta, A.; Kundu, S. Deoxygenation of Nitrosoarene by N-Heterocyclic Carbene (NHC): An Elusive Breslow-Type Intermediate Bridging Carbene and Nitrene. *Chem. Commun.* **2020**, *56* (81), 12166–12169.
(27) Greenhalgh, M. D.; Taylor, J. E.; Smith, A. D. Best Practice Considerations for Using the Selectivity Factor, s, as a Metric for the Efficiency of Kinetic Resolutions. *Tetrahedron* **2018**, *74* (38), 5554–5560.
(28) Monti, C.; Gennari, C.; Piarulli, U.; de Vries, J. G.; de Vries, A. H. M.; Lefort, L. Rh-Catalyzed Asymmetric Hydrogenation of Prochiral Olefins with a Dynamic Library of Chiral TROPOS Phosphorus Ligands. *Chem. – Eur. J.* **2005**, *11* (22), 6701–6717.
(29) Boogers, J. A. F.; Felfer, U.; Kotthaus, M.; Lefort, L.; Steinbauer, G.; de Vries, A. H. M.; de Vries, J. G. A Mixed-Ligand Approach Enables the Asymmetric Hydrogenation of an α-Isopropylcinnamic Acid En Route to the Renin Inhibitor Aliskiren. *Org. Process Res. Dev.* **2007**, *11* (3), 585–591.
(30) Renom-Carrasco, M.; Gajewski, P.; Pignataro, L.; de Vries, J. G.; Piarulli, U.; Gennari, C.; Lefort, L. A Mixed Ligand Approach for the Asymmetric Hydrogenation of 2-Substituted Pyridinium Salts. *Adv. Synth. Catal.* **2016**, *358* (16), 2589–2593.
(31) Mršić, N.; Lefort, L.; Boogers, J. A. F.; Minnaard, A. J.; Feringa, B. L.; de Vries, J. G. Asymmetric Hydrogenation of Quinolines Catalyzed by Iridium Complexes of Monodentate BINOL-Derived Phosphoramidites. *Adv. Synth. Catal.* **2008**, *350* (7–8), 1081–1089.
(32) Wales, D. J.; Doye, J. P. K. Global Optimization by Basin-Hopping and the Lowest Energy Structures of Lennard-Jones Clusters Containing up to 110 Atoms. *J. Phys. Chem. A* **1997**, *101* (28), 5111–5116.

(33) Goedecker, S. Minima Hopping: An Efficient Search Method for the Global Minimum of the Potential Energy Surface of Complex Molecular Systems. *J. Chem. Phys.* **2004**, *120* (21), 9911–9917.
(34) Neese, F. The ORCA Program System. *WIREs Comput. Mol. Sci.* **2012**, *2* (1), 73–78.
(35) Neese, F. Software Update: The ORCA Program System, Version 4.0. *WIREs Comput. Mol. Sci.* **2018**, *8* (1), e1327.
(36) Neese, F.; Wennmohs, F.; Becker, U.; Riplinger, C. The ORCA Quantum Chemistry Program Package. *J. Chem. Phys.* **2020**, *152* (22), 224108.
(37) *Extended tight-binding quantum chemistry methods - Bannwarth - 2021 - WIREs Computational Molecular Science - Wiley Online Library*.
(38) Hohenberg, P.; Kohn, W. Inhomogeneous Electron Gas. *Phys. Rev.* **1964**, *136* (3B), B864–B871.
(39) Kohn, W. Self-Consistent Equations Including Exchange and Correlation Effects. *Phys. Rev.* **1965**, *140* (4A), A1133–A1138.
(40) Parr, R. G. Density Functional Theory of Atoms and Molecules. In *Horizons of Quantum Chemistry*; Fukui, K., Pullman, B., Eds.; Springer Netherlands: Dordrecht, 1980; 5–15.
(41) Comstock, M. J. The Challenge of d and f Electrons. In *The Challenge of d and f Electrons*; M. Joan Comstock, Ed.; American Chemical Society, 1989; *Vol.,394*.
(42) Chai, J.-D.; Head-Gordon, M. Long-Range Corrected Hybrid Density Functionals with Damped Atom–Atom Dispersion Corrections. *Phys. Chem. Chem. Phys.* **2008**, *10* (44), 6615–6620.
(43) Dunning, T. H., Jr. Gaussian Basis Sets for Use in Correlated Molecular Calculations. I. The Atoms Boron through Neon and Hydrogen. *J. Chem. Phys.* **1989**, *90* (2), 1007–1023.
(44) Kendall, R. A.; Dunning, T. H., Jr.; Harrison, R. J. Electron Affinities of the First-row Atoms Revisited. Systematic Basis Sets and Wave Functions. *J. Chem. Phys.* **1992**, *96* (9), 6796–6806.
(45) Woon, D. E.; Dunning, T. H., Jr. Gaussian Basis Sets for Use in Correlated Molecular Calculations. III. The Atoms Aluminum through Argon. *J. Chem. Phys.* **1993**, *98* (2), 1358–1371.
(46) Peterson, K. A.; Woon, D. E.; Dunning, T. H., Jr. Benchmark Calculations with Correlated Molecular Wave Functions. IV. The Classical Barrier Height of the H+H2→H2+H Reaction. *J. Chem. Phys.* **1994**, *100* (10), 7410–7415.
(47) Wilson, A. K.; van Mourik, T.; Dunning, T. H. Gaussian Basis Sets for Use in Correlated Molecular Calculations. VI. Sextuple Zeta Correlation Consistent Basis Sets for Boron through Neon. *J. Mol. Struct. THEOCHEM* **1996**, *388*, 339–349.
(48) Gaussian 16, Revision C.01, M. J. Frisch, G. W. Trucks, H. B. Schlegel, G. E. Scuseria, M. A. Robb, J. R. Cheeseman, G. Scalmani, V. Barone, G. A. Petersson, H. Nakatsuji, X. Li, M. Caricato, A. V. Marenich, J. Bloino, B. G. Janesko, R. Gomperts, B. Mennucci, H. P. Hratchian, J. V. Ortiz, A. F. Izmaylov, J. L. Sonnenberg, D. Williams-Young, F. Ding, F. Lipparini, F. Egidi, J. Goings, B. Peng, A. Petrone, T. Henderson, D. Ranasinghe, V. G. Zakrzewski, J. Gao, N. Rega, G. Zheng, W. Liang, M. Hada, M. Ehara, K. Toyota, R. Fukuda, J. Hasegawa, M. Ishida, T. Nakajima, Y. Honda, O. Kitao, H. Nakai, T. Vreven, K. Throssell, J. A. Montgomery, Jr., J. E. Peralta, F. Ogliaro, M. J. Bearpark, J. J. Heyd, E. N. Brothers, K. N. Kudin, V. N. Staroverov, T. A. Keith, R. Kobayashi, J. Normand, K. Raghavachari, A. P. Rendell, J. C. Burant, S. S. Iyengar, J. Tomasi, M. Cossi, J. M. Millam, M. Klene, C. Adamo, R. Cammi, J. W. Ochterski, R. L. Martin, K. Morokuma, O. Farkas, J. B. Foresman, and D. J. Fox. Gaussian, Inc., Wallingford CT. 2016.
(49) Grimme, S. Supramolecular Binding Thermodynamics by Dispersion-Corrected Density Functional Theory. *Chem. – Eur. J.* **2012**, *18* (32), 9955–9964.

(50) Luchini, G.; Alegre-Requena, J. V.; Funes-Ardoiz, I.; Paton, R. S. GoodVibes: Automated Thermochemistry for Heterogeneous Computational Chemistry Data. F1000Research April 24, 2020.
(51) Ditchfield, R.; Hehre, W. J.; Pople, J. A. Self-Consistent Molecular-Orbital Methods. IX. An Extended Gaussian-Type Basis for Molecular-Orbital Studies of Organic Molecules. *J. Chem. Phys.* **1971**, *54* (2), 724–728.
(52) Hehre, W. J.; Ditchfield, R.; Pople, J. A. Self—Consistent Molecular Orbital Methods. XII. Further Extensions of Gaussian—Type Basis Sets for Use in Molecular Orbital Studies of Organic Molecules. *J. Chem. Phys.* **1972**, *56* (5), 2257–2261.
(53) Hariharan, P. C.; Pople, J. A. The Influence of Polarization Functions on Molecular Orbital Hydrogenation Energies. *Theor. Chim. Acta* **1973**, *28* (3), 213–222.
(54) Hariharan, P. C.; Pople, J. A. Accuracy of AH n Equilibrium Geometries by Single Determinant Molecular Orbital Theory. *Mol. Phys.* **1974**, *27* (1), 209–214.
(55) Gordon, M. S. The Isomers of Silacyclopropane. *Chem. Phys. Lett.* **1980**, *76* (1), 163–168.
(56) Francl, M. M.; Pietro, W. J.; Hehre, W. J.; Binkley, J. S.; Gordon, M. S.; DeFrees, D. J.; Pople, J. A. Self-consistent Molecular Orbital Methods. XXIII. A Polarization-type Basis Set for Second-row Elements. *J. Chem. Phys.* **1982**, *77* (7), 3654–3665.
(57) Binning Jr., R. C.; Curtiss, L. A. Compact Contracted Basis Sets for Third-Row Atoms: Ga–Kr. *J. Comput. Chem.* **1990**, *11* (10), 1206–1216.
(58) Blaudeau, J.-P.; McGrath, M. P.; Curtiss, L. A.; Radom, L. Extension of Gaussian-2 (G2) Theory to Molecules Containing Third-Row Atoms K and Ca. *J. Chem. Phys.* **1997**, *107* (13), 5016–5021.
(59) Rassolov, V. A.; Ratner, M. A.; Pople, J. A.; Redfern, P. C.; Curtiss, L. A. 6-31G* Basis Set for Third-Row Atoms. *J. Comput. Chem.* **2001**, *22* (9), 976–984.
(60) Rassolov, V. A.; Pople, J. A.; Ratner, M. A.; Windus, T. L. 6-31G* Basis Set for Atoms K through Zn. *J. Chem. Phys.* **1998**, *109* (4), 1223–1229.
(61) Wadt, W. R.; Hay, P. J. Ab Initio Effective Core Potentials for Molecular Calculations. Potentials for Main Group Elements Na to Bi. *J. Chem. Phys.* **1985**, *82* (1), 284–298.
(62) Hay, P. J.; Wadt, W. R. Ab Initio Effective Core Potentials for Molecular Calculations. Potentials for the Transition Metal Atoms Sc to Hg. *J. Chem. Phys.* **1985**, *82* (1), 270–283.
(63) Hay, P. J.; Wadt, W. R. Ab Initio Effective Core Potentials for Molecular Calculations. Potentials for K to Au Including the Outermost Core Orbitals. *J. Chem. Phys.* **1985**, *82* (1), 299–310.
(64) *T. H. Dunning Jr. and P. J. Hay, In H. F. Schaefer III, Ed., Methods of Electronic Structure Theory, Vol. 2, Plenum Press, 1977. - References - Scientific Research Publishing.*
(65) Ehlers, A. W.; Böhme, M.; Dapprich, S.; Gobbi, A.; Höllwarth, A.; Jonas, V.; Köhler, K. F.; Stegmann, R.; Veldkamp, A.; Frenking, G. A Set of F-Polarization Functions for Pseudo-Potential Basis Sets of the Transition Metals Sc□Cu, Y□Ag and La□Au. *Chem. Phys. Lett.* **1993**, *208* (1), 111–114.
(66) Weigend, F.; Ahlrichs, R. Balanced Basis Sets of Split Valence, Triple Zeta Valence and Quadruple Zeta Valence Quality for H to Rn: Design and Assessment of Accuracy. *Phys. Chem. Chem. Phys.* **2005**, *7* (18), 3297–3305.
(67) Marenich, A. V.; Cramer, C. J.; Truhlar, D. G. Universal Solvation Model Based on Solute Electron Density and on a Continuum Model of the Solvent Defined by the Bulk Dielectric Constant and Atomic Surface Tensions. *J. Phys. Chem. B* **2009**, *113* (18), 6378–6396.

(68) Hopmann, K. H.; Bayer, A. On the Mechanism of Iridium-Catalyzed Asymmetric Hydrogenation of Imines and Alkenes: A Theoretical Study. *Organometallics* **2011**, *30* (9), 2483–2497.
